# New genotypes of *Helicobacter Pylori* VacA d-region identified from global strains

**DOI:** 10.1186/s12860-020-00338-2

**Published:** 2021-01-07

**Authors:** Djaleel Muhammad Soyfoo, Yussriya Hanaa Doomah, Dong Xu, Chao Zhang, Huai-Ming Sang, Yan-Yan Liu, Guo-Xin Zhang, Jian-Xia Jiang, Shun-Fu Xu

**Affiliations:** 1 Department of Gastroenterology, The First Affiliated Hospital of Nanjing Medical University, Nanjing, China; 2 Department of Obstetrics and Gynecology, The Second Affiliated Hospital of Nanjing Medical University, Nanjing, China; 3 Department of Electrical Engineering and Computer Science, Bond Life Sciences Center, University of Missouri, Columbia, MO USA; 4 Institute for Computational Biomedicine, Weill Cornell Medicine, New York, NY 10021 USA; 5 Division of Hematology and Medical Oncology, Department of Medicine, Weill Cornell Medicine, New York, NY 10021 USA

**Keywords:** Helicobacter pylori, Vacuolating toxin a, Bioinformatics, Polymorphism, Intrinsically disordered proteins

## Abstract

**Background:**

Pathogenesis of *Helicobacter Pylori* (HP) vacuolating toxin A (vacA) depends on polymorphic diversity within the signal (s), middle (m), intermediate (i), deletion (d) and c-regions. These regions show distinct allelic diversity. The s-region, m-region and the c-region (a 15 bp deletion at the 3′-end region of the p55 domain of the vacA gene) exist as 2 types (s1, s2, m1, m2, c1 and c2), while the i–region has 3 allelic types (i1, i2 and i3). The locus of d-region of the vacA gene has also been classified into 2 genotypes, namely d1 and d2. We investigated the “d-region”/“loop region” through bioinformatics, to predict its properties and relation to disease. One thousand two hundred fifty-nine strains from the NCBI nucleotide database and the dryad database with complete vacA sequences were included in the study. The sequences were aligned using BioEdit and analyzed using Lasergene and BLAST. The secondary structure and physicochemical properties of the region were predicted using PredictProtein.

**Results:**

We identified 31 highly polymorphic genotypes in the “d-region”, with a mean length of 34 amino acids (9 ~ 55 amino acids). We further classified the 31 genotypes into 3 main types, namely K-type (strains starting with the KDKP motif in the “d-region”), Q-type (strains starting with the KNQT motif), and E-type (strains starting with the ESKT motif) respectively. The most common type, K-type, is more prevalent in cancer patients (80.87%) and is associated with the s1i1m1c1 genotypes (*P* < .01). Incidentally, a new region expressing sequence diversity (2 aa deletion) at the C-terminus of the p55 domain of vacA was identified during bioinformatics analysis.

**Conclusions:**

Prediction of secondary structures shows that the “d-region” adopts a loop conformation and is a disordered region.

## Background

Since its discovery in 1983, the causal relationship between *Helicobacter pylori* and gastric diseases has been irrefutable [1]. In 1994, the International Agency for Research on Cancer categorized *Helicobacter pylori* (HP) as a Class 1 carcinogen due to its strong correlation with gastric adenocarcinoma and mucosa-associated lymphoid tissue (MALT) lymphoma [2]. According to Yamaoka [3], the pathogenesis of HP has been linked to its virulence genes, namely the cytotoxin-associated gene A (*cag*A), outer-membrane proteins [4] and vacuolating toxin A (*vacA*) [5].

VacA is an intracellular-acting exotoxin that was initially described as a proteinaceous component with the ability to cause vacuolation in eukaryotic cells [6]. All HP strains contain a single chromosomal vacA gene, which encodes a protein about 140-kDa in mass [7]. After translation and proteolysis, two domains namely p33 and p55 are secreted, both of which are required for efficient binding of the toxin to the plasma membrane of cells [4, 8–13]. Although the *vacA* gene is found in all of the isolated *H. pylori* strains, only about 50% of the isolates have vacuolating activity. This is because vacA expresses allelic and genetic diversity within the *vacA* gene [4, 14]. The variation between vacA strains is mainly attributed to the vacA gene polymorphisms within the signal (s), middle (m), intermediate (i) [15] and more recently the deletion (d) and c-regions (a 15 bp deletion located at the 3′ end of the p55 domain of the vac A gene) [16]. The d-region, first described by Ogiwara et al. in 2009 was defined as an 81 base pair deletion between the i- and m-regions. The authors speculated that it might be responsible for binding of vacA to host gastric cells and vacuolating activity. The d-region was categorised as 2 genotypes namely d1 (no deletion or short deletions ranging from 9 to 23 bp) and d2 (with 81 bp deletion), but their roles in disease are unclear and available evidence is conflicting [17].

Another study by Telford et al. in 1994 described a loop region in the vacA toxin. In their study of the vacA toxin, Telford et al. found that the vacA precursor toxin undergoes proteolytic cleavage to produce a 37-kDa amino-terminal fragment and a 58-kDa carboxy-terminal fragment. The authors mentioned that this protein-specific cleavage occurred at a highly hydrophilic region made up of a short repeat of 8 amino acids (AKNDKXES), which connect the two subunits of the vacA toxin. Further secondary structure predictions indicated that this region formed a flexible exposed loop in the protein [10]. Burroni et al. hypothesized that the hydrophilic loop forms a hinge region between the two subunits and proteolysis at this site may be important for activity of the toxin. However, the results from their study concluded otherwise [18]. Another study by Tombola et al. showed that the loop region influences average channel conductance and the propensity of the toxin to enter artificial lipid bilayers [19].

The aim of our study is to investigate the “d-region”/“loop region” through bioinformatics and its relation to clinical outcomes. Recently, cryo-EM studies have reported that there is a missing region at residues 300–334 between p33 and p55 domains (HP strain 60,190), which manifests as a weak and discontinuous density in the center of all vacA oligomers on a 3D map. The authors suggest that this region represents a highly flexible loop found between the p33 and p55 domains [20, 21]. Therefore, we also performed predictions about the secondary structure and physicochemical properties of this particular region. Additionally, we theorized that the short deletions of 9–23 bp, currently denoted as d1 type should each have their own genotypic classification.

## Results

### VacA genotypes amongst HP strains

**Fig. 1:**
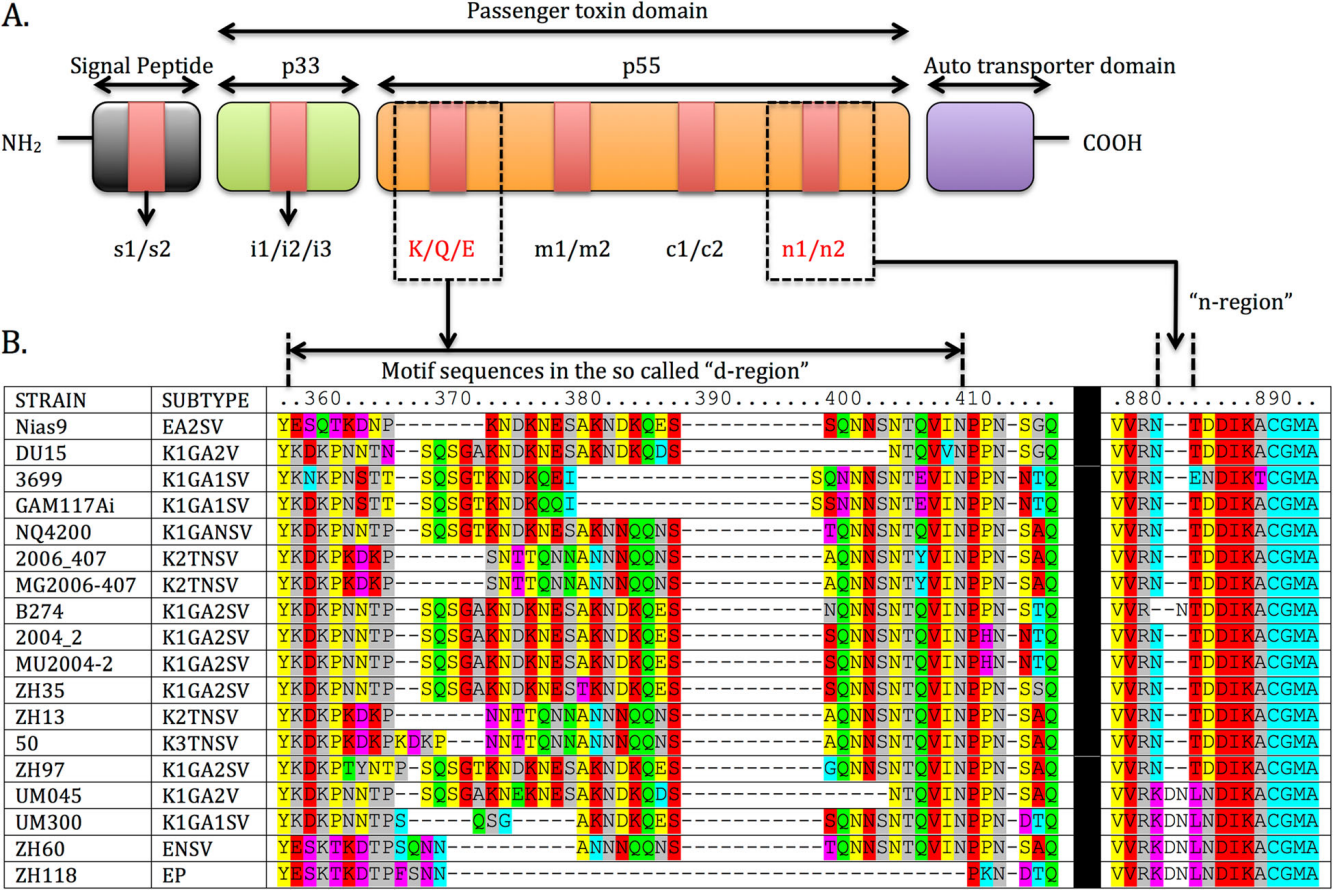
**a** The location of the different genotypes in the vacA protoxin: signal region(s) and intermediate region (i) on p33 domain. On the p55 domain, we can identify the middle region (m), c region (c) and the incidentally identified “tail-region” (n). This n-region exists as 2 genotypes: n1 (2 amino acid residue deletion) and n2 (without deletion). Between the i- and m- region (currently defined as “d-region”), we found 3 common occurring genotypes, which are denoted by K (K-type), Q (Q-type) and E (E-type). These genotypes are further divided into subtypes due to a high degree of polymorphism. **b**: Multiple sequence alignment from BioEdit of 18 isolates showing the highly polymorphic (so-called “d-region”) and the newly identified n-region. The high degree of polymorphism indicates that categorizing this region as d1 or d2 could be inadequate. This polymorphic region has a mean length of 34 amino acids. 10 motifs can be identified within these 18 isolates: KDKP, ESKT, KNQT, SQNN, NTQV, ANNN, ANDK, PKND, NTQV, and NNTP. The sequence of amino acid residues in the 3′ end of the p55 domain, denoted by the “n-region” shows two distinct genotypes with n1 (2 amino acid residue deletion) and n2 (without deletion). The most common occurring genotype across the studied 1259 strains is the n1 genotype

Alignment of the 1259 vacA sequences using the BioEdit software revealed the distinct genotypes of the well-documented s-, i-, m- and c- regions. We incidentally detected a new region expressing sequence diversity at the C-terminal of the p55 domain. We defined this region as the “n-region” and identified 2 genotypes: n1 genotype (with 2 aa deletion) and n2 genotype (without deletion). (Fig. 1a). Furthermore, within the “d-region”, complex variations in amino acids amongst strains could be found. BLAST analysis showed that this region expresses a high degree of polymorphism across the 1259 strains. (Fig. 1b and Supplementary Table 1) The length of this variable region ranges from 9 to 55 amino acids and has a mean length of 34 amino acids. In the *Helicobacter pylori* strain 60,190, it is located between amino acids 332–367.

**Fig. 2:**
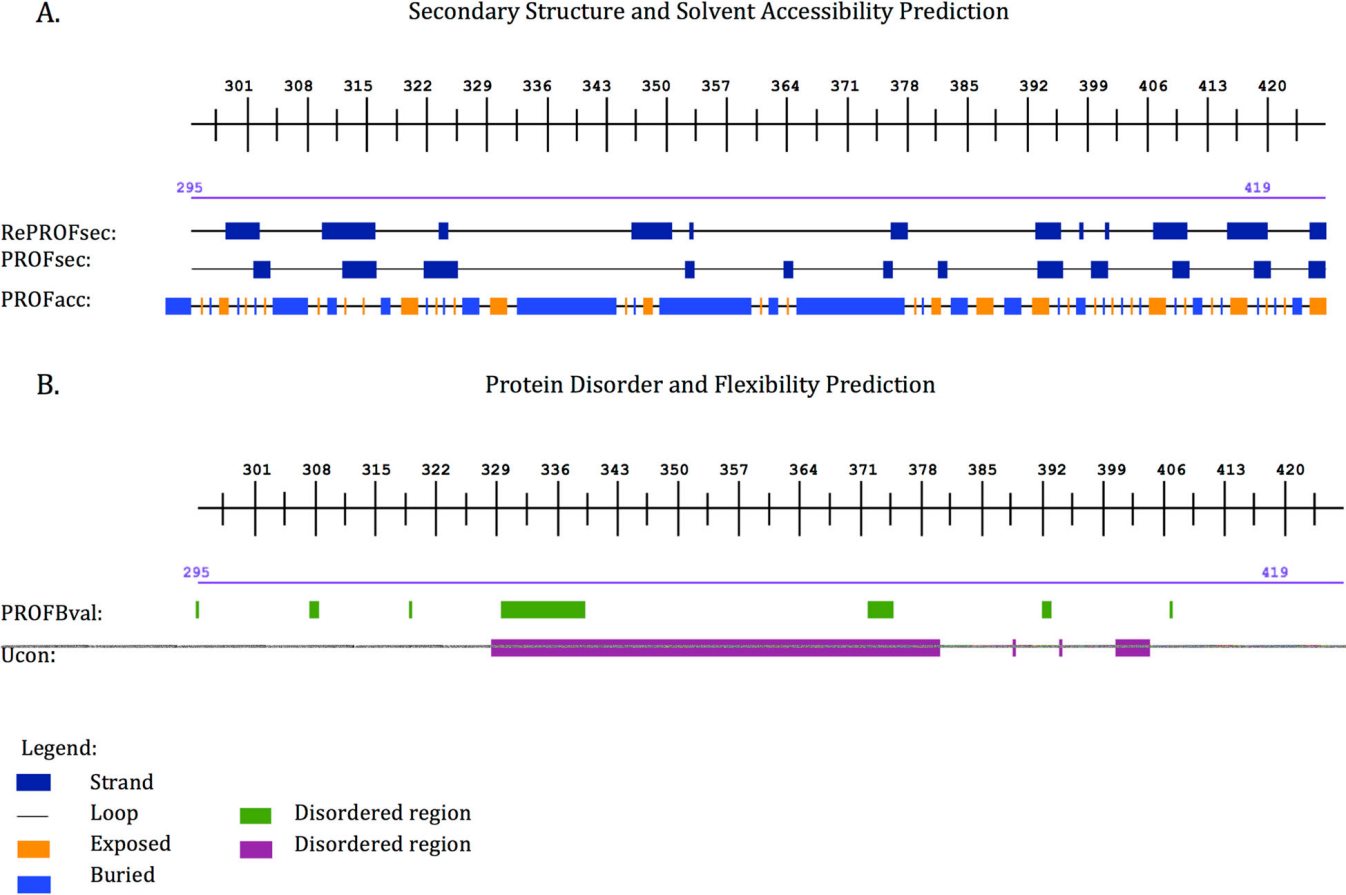
**a** The secondary structure and solvent accessibility information generated by PredictProtein server (https://predictprotein.org/). RePROFsec and PROFsec generate the predicted secondary structures. Two states of secondary structure are predicted in this region (295aa-419aa): strand and loop. PROFAcc generates the prediction of solvent accessibility of the residues: Exposed residues within this region (light blue) were higher. **b** Protein disorder through Ucon (orange) reveals presence of an intrinsic disorder region (approximately 50 amino acids long)

### Identification of motifs within the region currently defined as the “d-region”

Sequence analysis using BLAST and Lasergene showed the presence of 10 possible amino acid motifs (KDKP, ANDK, ESKT, KNQT, SNTT, NNTP, SQNN, ANNN, NTQV and PKND) within the region as shown in Table 1. We simplified the motifs using single letter codes (K, A, E, Q, T, G, S, N, V, and P). In depth analysis showed that this region consists of a combination of motifs across the 1259 strains. (Details about all the strains included are available in Supplementary Table 2). The combinations of motifs form subtypes that may start with K (K-type), Q (Q-type), A (A-type), T (T-type) or E (E-type). Table 2 shows the 31 identified combinations (31 subtypes) in this particular region among strains. The number of subtypes that start with K-,Q-, A-, T- and E- are 19, 5, 1,1, and 5, respectively. The most common subtypes were EP, K1GA1SV, K1GA2SV, K1GA2V, K2TNSV, and QGA2SV occuring at frequencies of 26.23, 14.02, 17.28, 20.21, 9.89 and 5.50%, respectively. Since the A-type and T-type occurred only once respectively, we did not include them in statistical analyses.

**Table 1:**
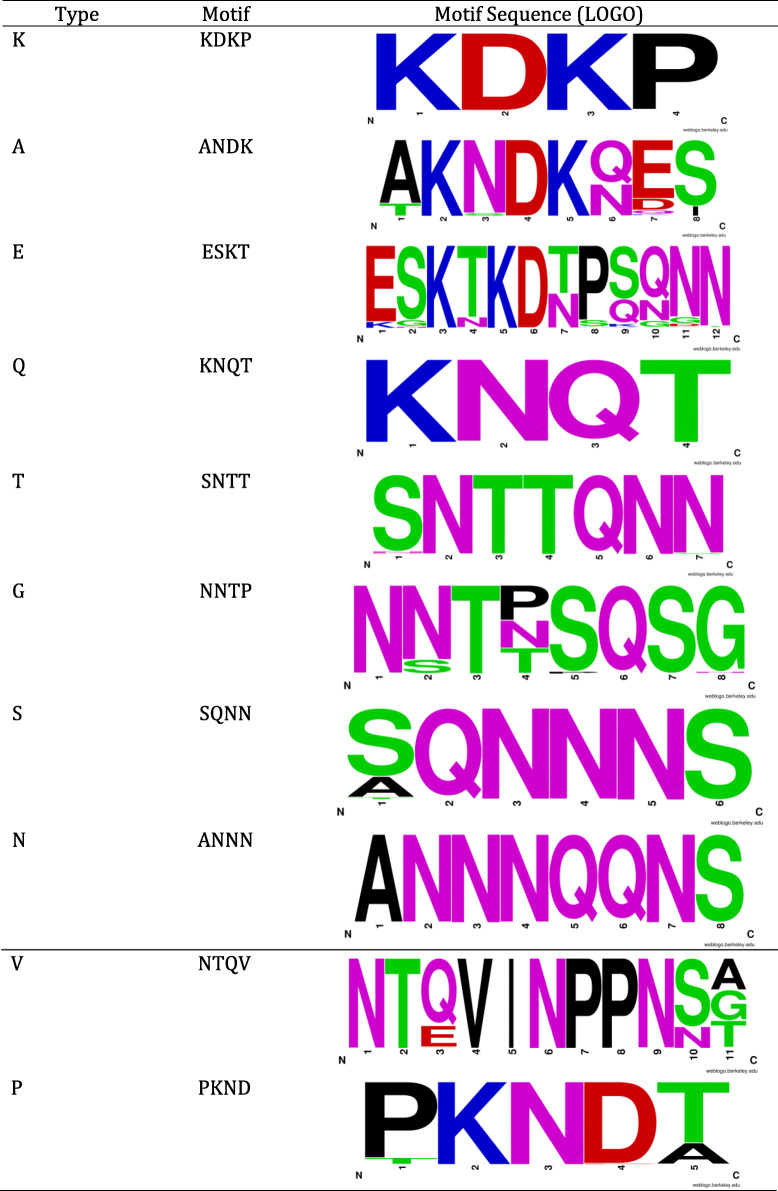
The 10 identified motifs within the current “d-region”

The motif logos show the variation of amino acids that have been identified among the 1259 strains. Each logo consists of stacks of symbols, one stack for each position in the sequence. The overall height of the stack indicates the sequence conservation at that position while the height of symbols within the stack indicates the relative frequency of each amino acid at that position. The width of the stack is proportional to the fraction of valid symbols in that position. In order to simplify the motifs present, a single letter code was assigned to each of them (K,A,E,Q,T,G,S,N,V and P). Each strain has a combination of the above motifs, which usually start with K, Q or E. Only 2 strains have been identified that start with motifs A and T.

**Table 2:** The prevalence of vacA genotypes

VacA	VacA Alleles	Strain Type		CagA (motif-C)				CagA (motif-D)		
		East Asian(%)	Western(%)	0	1	2	3	0	1	2
s-region	s1	159 (97.54)	792 (79.2)	21	470	74	8	21	199	5
	s2	4 (2.46)	208 (20.8)	2	7	0	0	2	0	0
i-region	i1	142 (87.12)	711 (71.1)	15	421	72	6	15	194	5
	i2	21 (12.88)	289 (28.9)	8	56	2	2	8	5	0
m-region	m1	130 (79.75)	596 (59.6)	10	370	63	5	10	146	5
	m2	33 (20.25)	404 (40.4)	13	107	11	3	13	53	0
c-region	c1	129 (79.14)	564 (56.4)	10	359	62	4	10	136	4
	c2	34 (20.86)	436 (43.6)	13	118	12	4	13	63	1
n-region	n1	132 (80.98)	590 (59)	11	363	64	4	11	148	5
	n2	31 (19.02)	410 (41)	12	114	10	4	12	51	0
**“d-region”**	**K-type**	**142 (87.12)**	**629 (62.9)**	**12**	**370**	**63**	**6**	**12**	**193**	**5**
	K1A1SV	0	2							
	K1A2SV	0	3							
	K1A2V	0	1							
	K1GA1SV	0	163							
	K1GA1V	1	3							
	K1GA2	0	1							
	K1GA2SV	63	138							
	K1GA2V	75	160							
	K1GA3SV	1	3							
	K1GA3V	1	1							
	K1GANSV	0	1							
	K1GP	0	12							
	K1N2SV2	0	1							
	K1TA1SV	0	3							
	K1TA2SV	0	1							
	K1TNSV	0	6							
	K2TA1SV	0	13							
	K2TNSV	1	114							
	K3TNSV	0	3							
	**E-type**	**20 (12.27)**	**300 (30.0)**	**10**	**60**	**2**	**2**	**10**	**5**	**0**
	E	0	4							
	EA1SV	0	1							
	EA2SV	0	9							
	ENSV	0	1							
	EP	20	285							
	Q-type	1 (0.61)	69 (6.9)	0	47	9	0	0	1	0
	QA2SV	0	1							
	QGA1SV	0	2							
	QGA2SV	1	63							
	QGA2SV2	0	1							
	QGA2V	0	2							
	**A-type** (A2SV)	0	1 (0.001)	0	0	0	0	0	0	0
	**T-type** (TNS)	0	1 (0.001)	0	0	0	0	0	0	0

### Correlation between east Asian and Western strain and vacA genotypes

From the 1259 strains, 163 were East Asian isolates (China, Japan and Korea) and 1000 were from Western countries (North America, Europe, Australia and other countries). Among the identified 163 East-Asian strains, 20/163 (12.3%) were of E-type, 142/163 (87.1%) were of K-type and 1/163 (1.59%) of Q-type. In contrast, among the Western strains, 300/1000 (30%) were E-type, 630/1000 (63%) were K-type while 66/1000 (6.6%) expressed the Q-type respectively. Table 2 shows the frequency of s-, i-, m-, c- and n- regions according to their strains. The frequency of the vacA K-type, s1, i1, m1, c1 and n1 genotypes was higher among East Asian isolates (87.12, 97.54%, 87,12, 79.75, 79.14 and 80.98% respectively) than Western type isolates (63.0, 72.2, 71.1, 59.6, 56.4 and 59%, respectively) (*P* < .01). These genotypes were more associated with East Asian type strains. The odds ratio was 0.25 (99% confidence interval, CI, 0.1565–0.4053; *P* < .0001) for K-type, 10.44 (99% confidence interval, CI, 3.8254–28.4885; *P* < .0001) for s1, 2.75 (99% confidence interval, CI, 1.7038–4.4338; *P* < .0001) for i1, 2.67 (99% confidence interval, CI, 1.7857–3.9932; *P* < .0001) for m1, 2.93 (99% confidence interval, CI, 1.9701–4.3667; *P* < .0001) for c1 and 2.96 (99% confidence interval, CI, 1.9618–4.4630; *P* < .0001) for n1. Moreover, the frequency of the E-type and Q-type among Western strains (30, 6.9%) was higher than East-Asian type (12.27, 0.61%) isolates. The vacA E- and Q-types were more associated with Western strains. The odds ratio was 3.06 (99% confidence interval, CI, 1.8828–4.9872, *P* < .0001) for E-type and 12.01 (99% confidence interval, CI, 1.6558–87.0600, *P* = .0018) for Q-type.

**Table 3:** Frequency of vacA E-, K- and Q- types in CA and NCA patients

VacA	Type	CA (%)	NCA (%)	Total	*P*-value
‘d’-region	E	14 (12.17)	160 (27.40)	174	0.0007
	K	93 (80.87)	367 (62.84)	460	
	Q	8 (6.96)	57 (9.76)	65	
s-i-m-c-n-‘E/K’	s1i1m1c1n1E	0 (0)	0 (0)	0	0.0009
combinations	s1i1m1c1n1K	74 (64.35)	293 (50.0)	367	
	s2i2m2c2n2E	10 (8.70)	94 (16.04)	104	
	s2i2m2c2n2K	0 (0)	2 (0.34)	2	
	Others^a^	31 (26.96)	197 (33.62)	228	

*CA* Cancer and MALT patients, *NCA* Atrophic gastritis, intestinal metaplasia, chronic gastritis, functional dyspepsia, gastric ulcer, duodenal ulcer and volunteers. The table also shows the frequencies of E and K types when combined with vacA s, i m, c and n genotypes. Others^a^ are various combinations that were found other than the ones mentioned in the above table

### Prevalence of HP vacA genotypes in CA (gastric adenocarcinoma) vs. NCA (non gastric cancer) groups

The frequency of the vacA K-type was higher in CA (80.87%) than NCA cases (62.84%). Conversely, the incidence of vacA E-type and Q-type was higher in NCA patients (27.40 and 9.76%) compared to CA patients (12.17 and 6.96%) (*P* < .01, Table 3). An analysis of the relationship between the E-type or K-type in combination with the other genotypes (s-, i-, m-, c-, n-) of vacA shows that the K-type is more associated with s1i1m1c1n1 genotypes (367/759, 48.35%) rather than s2i2m2c2n2 genotypes (2/206, 0.97%) (*P* < .01). Additionally, the combined s1i1m1c1n1-K genotype is more frequent among cancer patients (74/115, 64.35%) than non-cancer patients (293/586, 50%) (*P* < .01). However, the difference between the s2i2m2c2n2-K/E genotypes and disease outcomes was not statistically significant. (*P* > .01).

### Prevalence of HP vacA genotypes among disease groups. (CA vs. DU; CA vs. CG; CG vs. AG)

The occurrence of the vacA K-type, and i1 genotype among CA patients (86.92 and 87.83%) was significantly higher than DU patients (69.23 and 69.81%) (*P* < .01). The odds ratio (OR) was 2.95 (99% confidence interval, CI, 1.3082–6.6629; *P* = .0075) for K-type and 0.32 (99% CI, 0.1426–0.7207; *P* = .0046) for i1. There was no significant difference between the frequencies of vacA s1/−s2, m1/−m2, c1/−c2 and n1/−n2 genotypes in CA patients compared to DU patients (*P* > .01, Table 4).

Comparison between the CA and CG patient groups revealed that the frequency of occurrence of vacA K-type, s1, i1, m1, c1 and n1 genotypes in CA (86.92, 90.43, 87.83, 75.65, 71.30 and 73.91%, respectively) was higher than in CG (67.35, 79.17, 70.28, 57.22, 54.72 and 58.61%, respectively) (*P* < .01). A simple logistic regression analysis showed that these genotypes were significantly associated with the risk of CA; the odds ratio (OR) was 3.22 (99% confidence interval, CI, 1.7568–5.9016; *P* < .0001) for K-type, 0.40 (99% CI, 0.2054–0.7866; *P* = .0063) for s1, 0.33 (99% CI, 0.1793–0.5989; *P* = .0002) for i1, 0.49 (99% CI, 0.3088–0.7660; *P* = .0017) for c1, 0.43 (99% CI, 0.2679–0.6918; *P* = .0004) for m1, and 0.50 (99% CI, 0.3136–0.7966; *P* = .0032) for n1. (Table 4).

**Table 4:** Association between vacA genotypes and different diseases

VacA Geno-type	Control	Disease 1		Disease 2		Control	Disease	
	CG (%)	CA (%)	*P*-value	AG (%)	*P*-value	DU (%)	CA (%)	*P*-value
E	111 (32.65)	**14 (13.08)**	**< 0.0001**	18 (22.22)	0.0674	**16 (30.77)**	**14 (13.08)**	**0.0075**
K	229 (67.35)	**93 (86.92)**		63 (77.78)		**36 (69.23)**	**93 (86.92)**	
s1	285 (79.17)	**104 (90.43)**	**0.0063**	101 (87.83)	0.0383	49 (92.45)	104 (90.43)	0.7778
s2	75 (20.83)	**11 (9.57)**		14 (12.17)		4 (7.55)	11 (9.57)	
i1	**253 (70.28)**	**101 (87.83)**	**0.0002**	**96 (83.48)**	**0.0052**	**37 (69.81)**	**101 (87.83)**	**0.0046**
i2	**107 (29.72)**	**14 (12.17)**		**19 (16.52)**		**16 (30.19)**	**14 (12.17)**	
m1	**206 (57.22)**	**87 (75.65)**	**0.0004**	**92 (80)**	**< 0.0001**	33 (62.26)	87 (75.65)	0.0743
m2	**154 (42.78)**	**28 (24.35)**		**23 (20)**		20 (37.74)	28 (24.35)	
c1	**197 (54.72)**	**82 (71.30)**	**0.0017**	**89 (77.39)**	**< 0.0001**	33 (62.26)	82 (71.30)	0.2413
c2	**163 (45.28)**	**33 (28.70)**		**26 (22.61)**		20 (37.74)	33 (28.70)	
n1	**211 (58.61)**	**85 (73.91)**	**0.0032**	**92 (80)**	**< 0.0001**	34 (64.15)	85 (73.91)	0.1958
n2	**149 (41.39)**	**30 (26.09)**		**23 (20)**		19 (35.85)	30 (26.09)	

Significant values are shown in bold

**Table 5:** Association between number of KDKP repeats and disease outcomes

nKDKP	DU (%)	CA (%)	*P* value
0	17 (32.08)	22 (19.13)	0.0008
1	35 (66.04)	69 (60)	
2	1 (1.89)	23 (20)	
3	0 (−)	1 (0.87)	

*nKDKP* Number of KDKP repeats within the region, *DU* Duodenal ulcer, *CA* Gastric Cancer and MALT

Similar investigations between AG and CG patient groups indicated that the frequency of the vacA i1, m1, c1 and n1 genotypes in AG patients (83.48, 77.39, 80 and 80%, respectively) was significantly higher than in CG patients (70.28, 54.72, 57.22 and 58.61%, respectively) (*P* < .01). These genotypes were significantly associated with an increased risk of AG; the odds ratio (OR) was 0.47 (99% confidence interval, CI, 0.2723–0.8043; *P* = 0.0052) for i1, 0.33 (99% CI, 0.2024–0.5527; *P* < .0001) for m1, 0.35 (99% CI, 0.2177–0.5727; *P* < .0001) for c1, and 0.35 (99% CI, 0.2141–0.5853; *P* < .0001) for n1. There was no significant difference between the frequencies of vacA K−/E- types and s1/−s2 genotypes in isolates from AG and CG (*P* > .01, Table 4).

### Number of KDKP motif repeats and disease outcome

Our findings suggest that when the KDKP motif repeats twice, it occurs more frequently in CA (20%, 23/115) compared to DU (1.89%, 1/53) (*P* < .01). On the other hand, strains without any KDKP (i.e. strains expressing either ESKT or KNQT) motif repeat are more frequent in DU patients (32.08%, 17/56) than in CA patients (19.13%, 22/115) (*P* < .01). (Table 5).

### Relationship of vacA E−/K−/Q- type with cagA EPIYA-C/−D motifs

From the 1259 strains, 782 strains were cagA-EPIYA positive. The analysis of the distribution of the EPIYA motifs C or D patterns among the 782 strains showed that the vacA K-type was more frequent among cagA EPIYA-D motifs (198/204, 97.05%) compared to the cag A EPIYA–C (439/559, 78.53%) motifs (*P* < .01). On the other hand, E- and Q-types were more prevalent among cagA EPIYA-C motifs (64/559, 11.45% and 56/559, 10.02%, respectively) compared to cag A EPIYA-D motifs (5/204, 2.45% and 1/204, 0.05%) (*P* < .01).

### Secondary structure and physicochemical properties of the “d-region”

Fasta sequences obtained from BioEdit were used in the software PredictProtein to obtain the prediction of the physicochemical properties. Secondary structure prediction using the PROFsec method showed that this region lacks a regular secondary structure and tends to adopt a loop conformation. Further software predictions by PredictProtein showed that the amino acid residues within the region have a relatively exposed solvent accessibility rather than buried. This property could contribute to the hydrophilicity of the region. Additionally, protein disorder was predicted using the Meta-Disorder predictor method. The software revealed that the “d-region” expresses protein disorder and is flexible as shown in Fig. 2. A comparison of the amino acids within the full sequence of vacA showed that the ratio of “disorder promoting residues” (lysine, glutamine, glutamate, aspartate, arginine, serine and proline) in the “d-region” (4.53%) was higher than that of “order promoting residues” (1.91%) (leucine, threonine, valine, phenylalanine, isoleucine, tyrosine, asparagine and histidine). Further evaluation of amino acid composition within the “d-region” revealed that the proportion of “order promoting residues” was 39.22% whereas the proportion of “disorder promoting residues” was 60.78%. These features suggest that the “d-region” could possibly be an intrinsic disorder region (IDR).

## Discussion

Results obtained from the NCBI public database and Perl programming show that there is a highly polymorphic region located between amino acids 332–367 in the HP strain 60,190. Our study included a large pool of strains reported worldwide and revealed several subtypes within this region. We therefore suggest that the region found between the i- and m- regions is not limited to the 2 genotypes as currently defined by the d-region.

This polymorphic region located between the C-terminus of the p33 and the N-terminus of the p55 domains is on average 34 amino acids long, hydrophilic, flexible and expresses protein disorder. Its atypical secondary structure shows a remarkable resemblance to the “protease-sensitive loop” defined by Telford et al. in 1994 [10]. Further mention of this loop region surfaced in 1998 when Burroni et al. decided to investigate its role in toxin activity [18]. The authors stated that the proportion of heptamers and hexamers formed by vacA appears to be related to the length of the loop connecting the p37 and p58 domains. In their study, the loops of strains 60,190 and 9554 were shorter than that of strain 17,874 by eight and five amino acids, respectively. The study was able to demonstrate that the deletion of 16 amino acids from the loop of 17,874, led to a drop in the percentage of heptamers from 70 to 20%, whereas a complete (46 residues) loop deletion led to the exclusive formation of hexamers by the resulting m1del46 protein. Another study conducted by Tombola et al. showed that the loop region could influence the average channel conductance and propensity for the toxin to enter artificial lipid bilayers [19]. However, we noted a relative scarcity of articles pertaining to the above-mentioned ‘loop region’ found in vacA. Its role in the mechanism of vacA toxicity or in disease pathogenesis cannot be confirmed and remains ambiguous. Over the years, there was no further evaluation of this particular region until in 2009 when the d region, which occupies a similar locus, was defined.

We found that this particular region, besides occupying the same base pair regions as the deletion (d) region, also shares similar characteristics to intrinsic disorder regions. It is well documented that all the functional and structural peculiarities of IDPs/IDR are encoded in their amino acid sequences [22–25]. In addition, it has been recognized that there are significant differences between ordered protein/domains and IDP/IDR at the level of their amino acid sequences. Our study shows that there was a noticeable difference in amino acid composition within the region and it comprised mostly of disorder promoting residues. Intrinsic disorder in proteins manifests as a lack of stable tertiary structure and thus cannot be crystallized [26]. This causes the IDR to be flexible, a property shared by the identified polymorphic region. Similar findings have been described by Oguri et al., where the authors found intrinsically disordered proteins in *Salmonella enterica*, namely YciG, STM14_1829, and YmdF [27]. Another feature of the IDR is that it consists of repeated motifs of amino acids [28, 29]. Results from our study also revealed the presence of repeated amino acid sequences within the studied polymorphic region namely KDKP, ANDK and NTQV, and the number of repeats vary amongst strains. Intrinsic disorder in proteins can be present along the entire protein chain or in specific regions. A study by Hayashi et al. has demonstrated that IDR has been found in the C-terminus of the cagA toxin [30]. Like *H. pylori* CagA, many bacterial effectors are thought to contain IDRs, which are critical for their function and virulence [31, 32]. For example, in *Salmonella*, the flexibility provided by IDR promotes domain movement of virulence regulator protein ZirS [33] and E3 ubiquitin ligase Smurf2 [34]. Features that make IDRs suitable for use in effector proteins are: flexibility that facilitates interaction with multiple proteins; accessibility to post-translational modification target sequences by modification enzymes; rapid evolution of repeat motifs to confer augmentation of target binding or acquisition of new functions; and compactness [35]. The prevalence of IDRs (of over 40 residues) has also been found in simpler organisms such as prokaryotes, albeit at a relatively low prevalence (6–33%) [26]. Based on our findings, the presence of such a region within the vacA toxin is highly probable.

The identification of the polymorphic region brings new insight on the vacA structure and its possible implication with disease. It is well established that the VacA gene expresses polymorphism in the s-, i-, m- and c- regions, respectively, with each region showing distinct allelic diversity. The s-region, m-region and the c-regions exist as 2 types (s1, s2, m1, m2, c1 and c2), while the i–region has 3 allelic types (i1, i2 and i3). The locus of d-region of the vacA gene was also identified as being polymorphic and classified into 2 genotypes, d1 and d2 by Ogiwara et al. The d1 and d2 genotypes differed by the presence or absence of 81 base pairs respectively. However, in their study, short base pair deletions (9 to 23 bp) were also identified and classed as d1 genotypes. The d1 genotypes were considered to consist of approximately 367 to 379 base pairs [17]. We suggest that the presence of short base pair deletions should not be grouped as one genotype. In addition, some strains might possess more base pair deletions that are not restricted to 9–23 bp or 81 bp. In the eventuality of such occurrence, it would be problematic to define the d-genotype of those strains, as they would neither be d1 nor d2 genotype. Our study shows that there are many variations that could exist within the polymorphic region. We were able to identify 3 distinct genotypes namely K, E or Q whereby each of them exhibit allelic diversity. We found that the K-type has 19 different subtypes, while both the E-type and the Q-type have 5 subtypes each.

Several studies have attempted to determine the association between the d-region and disease outcome [16, 17, 36–38]. In a study by Ogiwara et al. [17], the d-region genotype was found to be significantly associated with neutrophil infiltration and gastric atrophy in both the antrum and the corpus in Western countries. A 2013 study by Lafiti-Navid et al. [38], determined the genotypic frequency of 138 *H. pylori* isolates and studied the allelic profiles between 2 Iranian populations with high and low incidence rates of GC respectively. The study found that the frequency of vacA d1/i1 was significantly higher among HP isolates from high incidence areas of GC, while d2/i2 genotypes were more prevalent in isolates from low incidence areas of gastric cancer. In 2014, Basiri et al. [37], showed that the frequency of allele d1 was significantly higher in HP isolates from patients with gastric adenocarcinoma (66.6%) and peptic ulcer disease (71.4%) than in those with gastritis (27.4%). Similarly, a study by Bakhti et al. [16], showed significant association between *vacA* d1 allele and gastric adenocarcinoma but not peptic ulcer disease (PUD), although the d1-type of vacA was higher in patients with PUD than that in controls. In 2017, Abdi et al. [36], found that the vacA i1 and d1 genotypes were significantly linked to an increase risk of gastric cancer where both cardia and non-cardia gastric cancer patients were entered into the analysis. Furthermore, d1 was significantly linked to the risk of diffused type adenocarcinoma, (OR 7.71). A parallel can thus be drawn between the reported literature and our study findings. Our results suggest that the vacA K-type was more associated with cancer and MALT while the vacA E- and Q-types were more prevalent in non-cancer cases. A similar result was found with other genotypes of vacA s1i1m1c1n1-K. In addition, we found that when the KDKP motif occurred twice within strains, its prevalence was higher among cancer patients than DU patients. Interestingly, strains without any KDKP motifs were more frequent in DU patients. Based on these findings, we assume that strains harboring the K-type are therefore more virulent than the E- or Q- types.

It is well established that s1i1m1 strains are more virulent and more likely associated with gastric cancer than the s2i2m2 strains [39–41]. However, the same cannot be concluded about the d genotype, as there are only few studies that have attempted to find a correlation between the d genotype and the other regions. The few existing results are conflicting [36, 38]. In our study, we found that most of the cancer patients who expressed the K-type also expressed the s1, m1, i1, c1 and n1 genotypes. These genotypes (s1,i1,m1,n1,c1,K) were found to be more prevalent in CA (gastric cancer and MALT) than CG (chronic gastritis and functional dyspepsia) cases. However, further studies are needed to shed more light on the synergism among the different vacA regions and the newly defined VacA K-, E- and Q- types as well as their impacts on disease. Whilst previous studies have clearly described the role of *vacA* s1, i1 and m1 in vacuolating activity [14, 42, 43], the mechanism by which vacA K-type causes pathogenicity is yet to be elucidated. Nonetheless, we hypothesize that vacA K-type contributes to the virulence of those strains.

In clinical practice, the i1 genotype has been found to be a good predictor of gastric diseases [40, 42, 44, 45]. Similarly, this region could also be used as a marker to screen patients for the risk of developing gastric carcinoma. Until now, we have struggled to find appropriate treatment measures and eradication methods of HP. Understanding the mechanism governing the association between virulence factors and disease can help for the future treatment of refractory HP infection. However, further studies are needed to investigate this region and its relationship to the cag PAI to understand the virulence of HP.

Our study has used bioinformatics analysis to predict the properties of the region found between the i- and m-regions of the vacA toxin. In our study, we compared strains using a combination of bioinformatics tools and crossed check each strain manually, thus making our comparison more accurate. However, our results are based on theoretical assumptions and we reckon that experimental research from laboratories would go a long way to help confirm our results and hypothesis.

## Conclusion

We found that the region defining d1 or d2 genotypes exists as multiple variants and while this particular region forms a loop conformation, it shares similar properties to intrinsically disordered proteins. We believe that, classifying this region as d-genotypes may not be suitable. Understanding the structure and nature of this region is primordial to determine its function and eventually its impact on the virulence of the vacA toxin.

## Methods

### HP strains and genome sequences

A search on *Helicobacter pylori* was performed in the NCBI nucleotide database (https://www.ncbi.nlm.nih.gov/) up to January 2019. Results obtained were filtered by taxon and a total of 178,492 sequence data on the *Helicobacter Pylori* (HP) genome was retrieved.

The search for more sequence data was broadened through the Dryad database, which yielded an additional 401 sequences. (https://datadryad.org/bitstream/handle/10255/dryad.134968/BIGSdb_gene-by-gene_alignment.fasta.gz?sequence=1).

The inclusion criteria consisted of sequences with complete information on strain name and vacA gene sequences. Results with inadequate data and duplicates were not included. Only human strains were considered and strains from animal origin as well as cultivated strains were excluded (e.g. 26,695–1, 26,695-1CH, 26,695-1CL, 26,695-1MET, HP87P7, HP87P7tlpDRI). The origin of the strains was also taken into account to avoid duplication. A total of 1259 HP strains were retrieved and included in the study. Further information about the cagA status of the selected strains were searched from the NCBI nucleotide database and only those with complete data about their cagA-EPIYA status were included in the study. The search yielded a total of 782 cagA positive strains. Information about the corresponding host diseases of the retrieved strains, their geographical characteristics and strain type were obtained from available sequence annotations and published references. The data on host diseases were then divided into 6 major groups namely: duodenal ulcer (DU), gastric ulcer (GU), chronic gastritis & functional dyspepsia (CG/FD), atrophic gastritis & intestinal metaplasia (AG/IM), gastric adenocarcinoma & MALT lymphoma (CA/MALT) and healthy control or volunteer (VOL). Patients diagnosed with gastrointestinal stromal tumor (GIST) and esophageal gastritis were excluded. Among the 1259 HP strains, 22 peptic ulcer patients were excluded in our statistical analysis since our patient groups consisted of DU and GU as separate entities. The data for the strains included in this study is available in the Dryad repository at:https://datadryad.org/stash/share/K1gZY8Um_sPzVB-GMR5jaJfs8XgtGMcXt2ogXgCeaRM.

### Bioinformatics analysis of the HP VacA amino acid sequences

The Perl programming language (ActivePerl version 5.26.1) was used to process the data obtained to build a database for the strains and their corresponding information. The sequences retrieved were subjected to multiple sequence alignment using BioEdit software version 7.0 (http://www.mbio.ncsu.edu/BioEdit/bioedit.html). Subsequently, each amino acid sequences of VacA from the 1259 strains were individually compared as well as using Bioedit and then analyzed using Lasergene version 7 (http://www.dnastar.com/products/lasergene.php), and BLAST (http://blast.ncbi.nlm.nih.gov/Blast.cgi). Sequence logos of motifs found within the sequences were constructed using Weblogo 2.8.2 (University of California, Berkeley). (http://weblogo.berkeley.edu).

### Prediction of the secondary structure and physicochemical properties of the “d-region”

PredictProtein software (Technical University of Munich, July 2013) was used to predict the properties of the “d-region”. Using the software we were able to predict the secondary structure, solvent accessibility, protein disorder tendency and flexibility of the region. PredictProtein uses PROFsec to predict secondary structure of elements, i.e. helix (H; includes alpha-, pi- and 3_10-helix), strand (beta-) and loop (L). PROFacc was used to predict solvent accessibility of protein residues, which are grouped into 2 states: buried or exposed. To predict protein disorder, the software uses Meta-Disorder predictor, which is a combination of several orthogonal methods that capture many types of disorder. All these properties helped in identifying the probable nature of the “d-region”.

### Statistical analysis

Statistical analysis was performed using SAS version 9.4 (http://www.sas.com) and the significance threshold was set at *P <* .01. Simple frequencies were used to analyze the distribution of strain types and occurrence of different motifs.

The Mantel-Haenszel test, Pearson Chi-square test and Fischer’s Exact tests were used to determine association between categorical variables where appropriate. The Cochran-Armitage test for trend was used to verify the association between the number of motif repeats and disease outcome. Odds ratios (OR) were used to determine the association between vacA genotypes and various clinical outcomes.

## Supplementary Information

**Additional file 1.**

**Additional file 2.**

## Data Availability

Data available in supplementary materials.

## Abbreviations

AG: Atrophic Gastritis
CA: Gastric Adenocarcinoma
CagA: Cytotoxin-associated gene A
CG: Chronic Gastritis
DU: Duodenal Ulcer
FD: Functional Dyspepsia
GIST: Gastrointestinal Stromal Tumor
GU: Gastric Ulcer
HP: Helicobacter pylori
IDP: Intrinsically Disordered Protein
IDR: Intrinsically Disordered Region
IM: Intestinal Metaplasia
MALT: Mucosa Associated Lymphoid Tissue
NCA: Non Gastric Cancer
OMP: Outer Membrane Protein
PU: Peptic Ulcer
VacA: Vacuolating cytotoxin A
VOL: Volunteer and Healthy Control
