# Supplementary material for: New genotypes of Helicobacter Pylori VacA d-region identified from global strains
Source: BMC Mol Cell Biol. 2021 Jan 7;22:4. doi: 10.1186/s12860-020-00338-2 (PMC7791883; doi:10.1186/s12860-020-00338-2)
Supplement: Supplementary file 1 — Additional file 1. [file 12860_2020_338_MOESM1_ESM.docx]

| **locus_strain_subtype** | **Sequences** |
| --- | --- |
| AF049642_F36_K1GA2SV | YKDKPNNTT--SQSGAKSDKNESAKNDKQES-----------SQNNSNTQVINPPN-SGQ |
| AB190969_OK109_K1GA2V | YKDKPNNTT--SQSGAKNDKNENAKNDKQDS----------------NTQVINPPN-SGQ |
| LC420353_Nias9_EA2SV | YESQTKDNP-------KNDKNESAKNDKQES-----------SQNNSNTQVINPPN-SGQ |
| LC420360_Medan67_K1GA2V | YKDKPNNTN--SQSGAKNDKNESAKNDKQDS----------------NTQVINPPN-SGQ |
| AF049627_F44_K1GA2V | YKDKPNNTN--SQSGAKNDKNESAKNDKQDS----------------NTQVINPPN-SGQ |
| NC_020509_OK310_K1GA2V | YKDKPNNTN--SQSGAKSDKNESAKNDKQDS----------------NTQVINPPN-SGQ |
| NZ_AP017349_F70_K1GA3V | YKDKPNNTN--SQSSAKNDKQESAKNDKQESVKNDKQDS--------NTQVINPPN-SGQ |
| NZ_CP011487_PNG84A_K1GA2V | YKDKPNNTN--SQSGAKNDKNESAKNDKQDS----------------NTQVINPPN-SGQ |
| NZ_JRAC01000009_Manado-1_K1GA2V | YKDKPNNTN--SQSGAKSDKNESAKNDKQDS----------------NTQVINPPN-SGQ |
| NZ_LQNA02000019_UM303S_K1GA2V | YKDKPNNTT--SQSGAKNDKNESAKSDKQDS----------------NTQVINPPN-SGQ |
| NZ_LQNB02000013_UM303R_K1GA2V | YKDKPNNTT--SQSGAKNDKNESAKSDKQDS----------------NTQVINPPN-SGQ |
| NC_017355_v225d_K1GA2SV | YKDKPNNTA--SQSGAKNDKNESAKNDKQGS-----------SQDNSNTQVINPPN-SGQ |
| NC_010698_Shi470_K1TA1SV | YKDKP-----------DNTTQNSAKNDKQKS-----------SQDNSNTQVINPPD-SGQ |
| NC_017359_Sat464_K1TA1SV | YKDKP-----------DNTTQNSAKNDKQKS-----------SQDNSNTQVINPPN-SGQ |
| NZ_MJMZ01000007_S468A_K1GA2V | YKDKPNNTN--SQSGAKNDKNESAKNDKQDS----------------NTQVINPPN-SGQ |
| AB190970_OK111_K1GA2SV | YKDKPNNTN--SQSGAKNDKNESAKNDKQES-----------SQNNSNTQVINPPN-SGQ |
| NZ_JPXD01000001_YN4-84_K1GA2SV | YKDKPNNTT--SQSGAKNDKNESAKNDKQES-----------SQNNSNTQVINPPN-SGQ |
| NC_017367_F57_K1GA2SV | YKDKPNNTN--SQSGAKNDKNESAKNDKQES-----------SQNNSNTQVINPPN-SGQ |
| NZ_AP017359_MKM1_K1GA2SV | YKDKPNNTN--SQSGAKNDKNESAKNDKQES-----------SQNNSNTQVINPPN-SGQ |
| NZ_AP017362_MKM6_K1GA2SV | YKDKPNNTT--SQSGAKNDKNESAKNDKQES-----------SQNNSNTQVINPPN-SGQ |
| NZ_AP017335_F211_K1GA2SV | YKDKPNNIN--SQSGAKSDKNESAKNDKQES-----------SQNNSNTQVINPPN-SGQ |
| NZ_AP017356_MKF10_K1GA2SV | YKDKPNNTN--SQSGAKNDKNESAKNDKQES-----------SQNNSNTQVINPPN-SGQ |
| LC185413_07-223_K1GA2SV | YKDKPNNTN--SQSGAKNDKNESAKNDKQES-----------SQNNSNTQVINPPN-SGQ |
| AF071096_F71_K1GA2SV | YKDKPNNTN--SQSGAKNDKNESAKNDKQES-----------SQNNSNTQVINPPN-SGQ |
| NZ_AP017338_F24_K1GA2SV | YKDKPNNTN--SQSGAKNDKNESAKNDKQES-----------SQNNSNTQVINPPN-SGQ |
| NC_017365_F30_K1GA2SV | YKDKPNNTN--SQSGAKNDKNESAKNDKQES-----------SQNNSNTQVINPPN-SGQ |
| NZ_AP017330_F17_K1GA2SV | YKDKPNNTN--SQSGAKNDKNESAKNDKQES-----------SQNNSNTQVINPPN-SGQ |
| LC185422_10-456_K1GA2SV | YKNKTDNTT--SQSGAKNDKNESAKNDKQES-----------SQNNSNTQVINPPN-SGQ |
| AB190985_OK194_K1GA2SV | YKDKPNNTN--SQSGAKNDKNESAKNDKQES-----------SQNNSNTQVINPPN-SGQ |
| LC185398_01-540_K1GA2SV | YKDKPNNTN--SQSGAKNDKNESAKNDKQES-----------SQNNSNTQVINPPN-SGQ |
| NZ_MVTU01000018_HP15051_K1GA2SV | YKDKPNNTN--SQSGAKNDKNESAKNDKQES-----------SQNNSNTQVINPPN-SGQ |
| AB190963_F26_K1GA2SV | YKDKPNNTN--SQSGAKNDKNESAKNDKQES-----------SQNNSNTQVINPPN-SGQ |
| LC185424_13-330_K1GA2SV | YKDKPNNTN--SQSGAKSDKNESAKNDKQES-----------SQNNSNTQVINPPN-SGQ |
| LC185405_02-729_K1GA2SV | YKDKPNNTN--SQSGAKSDKNESAKNDKQES-----------SQNNSNTQVINPPN-SGQ |
| AF049648_F68_K1GA2SV | YKDKPNNTN--SQSGAKSDKNESAKNDKQES-----------SQNNSNTQVINPPN-SGQ |
| AB190977_OK158_K1GA2SV | YKDKPNNTN--SQSGAKNDKNESTKNDKQES-----------SQNNSNTQVINPPN-SGQ |
| LC185396_01-383_K1GA2SV | YKDKPNNTN--SQSGAKNDKNESAKNDKQES-----------SQNNSNTQVINPPN-SGQ |
| LC185415_10-252_K1GA3SV | YKDKSNNTN--SQSGAKNDKNESAKNDKNESAKNDKQES---SQNNSNTQVINPPN-SGQ |
| NC_017375_83_K1GA2SV | YKDKPNNTN--SQSGAKNDKNESAKNDKQES-----------SQNNSNTQVINPPN-SGQ |
| NZ_AKNJ01000009_CPY1124_K1GA2SV | YKDKPNNTN--SQSGAKNDKNESAKNDKQES-----------SQNNSNTQVINPPN-SGQ |
| LC185401_01-633_K1GA2SV | YKDKSNNTN--SQSGAKNDKNESAKNDKQES-----------SQNNSNTQVINPPN-SGQ |
| LC185393_01-365_K1GA2SV | YKDKPNNTT--SQSGAKNDKNESAKNDKQES-----------SQNNSNTQVINPPN-SGQ |
| NZ_AKNO01000004_CPY6261_K1GA2SV | YKDKPNNTN--SQSGAKNDKNESAKNDKQES-----------SQNNSNTQVINPPN-SGQ |
| NZ_AP017329_F13_K1GA2SV | YKDKPNNTN--SQSGAKNDKNESAKNDKQES-----------SQNNSNTQVINPPN-SGQ |
| AF049626_F42_K1GA2SV | YKDKPNNTN--SQSGAKSDKNESAKNDKQES-----------SQNNSNTQVINPPN-SGQ |
| AF049641_F33_K1GA2SV | YKDKPNNTN--SQSGAKNDKNESAKNDKQES-----------SQNNSNTQVINPPN-SGQ |
| LC420359_Medan56_K1GA2SV | YKDKPNNTN--SQSGAKNDKNESAKNDKQES-----------SQNNSNTQVINPPN-SGQ |
| LC420357_Medan49_K1GA2SV | YKDKPNNTN--SQSGAKNDKNESAKNDKQES-----------SQNNSNTQVINPPN-SGQ |
| LC420362_Medan73_K1GA2SV | YKDKPNNTN--SQSGAKNDKNESAKNDKQES-----------SQNNSNTQVINPPN-SGQ |
| LC420363_Medan75_K1GA2SV | YKDKPNNTN--SQSGAKNDKNESAKNDKQES-----------SQNNSNTQVINPPN-SGQ |
| NZ_MVWY01000003_HP11055_K1GA2SV | YKDKPNNTN--SQSGAKNDKNESAKNDKQES-----------SQNNSNTQVINPPN-SGQ |
| LC185426_97-474_K1GA2SV | YKDKPNNTN--SQSGAKSDKNESAKNDKQES-----------SQNNSNTQVINPPN-SGQ |
| 694_98_10_K1GA2SV | YKNKTDNTT--SQSGAKNDKNESAKNDKQES-----------SQNNSNTQVINPPN-SGQ |
| NZ_ABSX01000030_98-10_K1GA2SV | YKNKTDNTT--SQSGAKNDKNESAKNDKQES-----------SQNNSNTQVINPPN-SGQ |
| NZ_MVVA01000003_HP14051_K1GA2SV | YKDKPNNTN--SQSGAKSDKNESAKNDKQES-----------SQNNSNTQVINPPN-SGQ |
| LC185394_01-375_K1GA2SV | YKDKPNNTN--SQSGAKNDKNESAKNDKQES-----------SQNNSNTQVINPPN-SGQ |
| LC185397_01-487_K1GA2SV | YKDKPNNTN--SQSGAKSDKNESAKNDKQES-----------SQNNSNTQVINPPN-SGQ |
| AF049652_F73_K1GA2SV | YKDKPNNTN--SQSGAKSDKNESAKNDKQES-----------SQNNSSTQVINPPN-SGQ |
| AF049625_F35_K1GA2SV | YKDKPNNTN--SQSGAKNDKNESTKNDKQES-----------SQNNSNTQVINPPN-SGQ |
| AB190967_OK101_K1GA2SV | YKDKPNNTN--SQSGAKNDKNESAKNDKQES-----------SQNNSNTQVINPPN-SGQ |
| AB190978_OK159_K1GA2SV | YKDKPNNTN--SQSGTKNDKNESAKNDKQES-----------SQNNSNTQVINPPN-SGQ |
| NZ_MVVF01000010_HP14031_K1GA2SV | YKDKPNNTN--SQSGAKNDKNESTKNDKQES-----------SQNNSNTQVINPPN-SGQ |
| 4456_Yangon173_K1GA2SV | YKDKPNNTN--SQSGAKNDKNESAKNDKQES-----------SQNNSNTQVINPPN-SGQ |
| NZ_AKNQ01000002_CPY6311_K1GA2SV | YKDKPNNTN--SQSGAKNDKNESAKNDKQES-----------SQNNSNTQVINPPN-SGQ |
| AF361700_CHN3554a_K1GA2SV | YKDKPNNTN--SQSGAKNDKNESAKNDKQES-----------SQNNSNTQVINPPN-SGQ |
| AF361701_CHN4611a_K1GA2SV | YKDKPNNTN--SQSGAKNDKNESAKNDKQES-----------SQNNSNTQVINPPN-SGQ |
| NZ_JDVJ01000022_wls-5-17_K1GA2SV | YKDKPNNTN--SQSGAKNDKNESAKNDKQES-----------SQNNSNTQVINPPN-SGQ |
| NZ_JDVM01000044_wls-5-14_K1GA2SV | YKDKPNNTN--SQSGAKNDKNESAKNDKQES-----------SQNNSNTQVINPPN-SGQ |
| NZ_JDVN01000020_wls-5-13_K1GA2SV | YKDKPNNTN--SQSGAKNDKNESAKNDKQES-----------SQNNSNTQVINPPN-SGQ |
| NZ_JDVO01000045_wls-5-11_K1GA2SV | YKDKPNNTN--SQSGAKNDKNESAKNDKQES-----------SQNNSNTQVINPPN-SGQ |
| NZ_JDVT01000101_wls-5-6_K1GA2SV | YKDKPNNTN--SQSGAKNDKNESAKNDKQES-----------SQNNSNTQVINPPN-SGQ |
| NZ_JDVU01000042_wls-5-5_K1GA2SV | YKDKPNNTN--SQSGAKNDKNESAKNDKQES-----------SQNNSNTQVINPPN-SGQ |
| NZ_JDVL01000048_wls-5-15_K1GA2SV | YKDKPNNTN--SQSGAKNDKNESAKNDKQES-----------SQNNSNTQVINPPN-SGQ |
| NZ_AUPD01000009_wls-5-3_K1GA2SV | YKDKPNNTN--SQSGAKNDKNESAKNDKQES-----------SQNNSNTQVINPPN-SGQ |
| NZ_JCKD01000017_wls-5-2_K1GA2SV | YKDKPNNTN--SQSGAKNDKNESAKNDKQES-----------SQNNSNTQVINPPN-SGQ |
| NZ_JCKE01000013_wls-5-1_K1GA2SV | YKDKPNNTN--SQSGAKNDKNESAKNDKQES-----------SQNNSNTQVINPPN-SGQ |
| NZ_JDVI01000026_wls-5-18_K1GA2SV | YKDKPNNTN--SQSGAKNDKNESAKNDKQES-----------SQNNSNTQVINPPN-SGQ |
| NZ_JDVP01000031_wls-5-10_K1GA2SV | YKDKPNNTN--SQSGAKNDKNESAKNDKQES-----------SQNNSNTQVINPPN-SGQ |
| NZ_JDVQ01000036_wls-5-9_K1GA2SV | YKDKPNNTN--SQSGAKNDKNESAKNDKQES-----------SQNNSNTQVINPPN-SGQ |
| NZ_JDVR01000055_wls-5-8_K1GA2SV | YKDKPNNTN--SQSGAKNDKNESAKNDKQES-----------SQNNSNTQVINPPN-SGQ |
| NZ_JDVS01000100_wls-5-7_K1GA2SV | YKDKPNNTN--SQSGAKNDKNESAKNDKQES-----------SQNNSNTQVINPPN-SGQ |
| NZ_JDVK01000075_wls-5-16_K1GA2SV | YKDKPNNTN--SQSGAKNDKNESAKNDKQES-----------SQNNSNTQVINPPN-SGQ |
| NZ_JDVV01000047_wls-5-4_K1GA2SV | YKDKPNNTN--SQSGAKNDKNESAKNDKQES-----------SQNNSNTQVINPPN-SGQ |
| HQ287753_191.9_K1GA2V | YKDKPNNTN--SQSGAKSDKNESAKNDKQDS----------------NTQVINPPN-SGQ |
| HQ287752_501.9_K1GA2V | YKDKPNNTN--SQSGAKNDKNESAKNDKQDS----------------NTQVINPPN-SGQ |
| NZ_MVWK01000018_HP12069_K1GA1V | YKDKPNNTN--SQSGAKSDKNESAKKTTS--------K-------IVTTQVINPPN-SGQ |
| NZ_MVUG01000015_HP15027_K1GA2V | YKDKPNNTN--SQSGAKNDKNESAKNDKQDS----------------NTQVINPPN-SGQ |
| GQ331979_MZ12_K1GA2V | YKDKPNNTN--SQSGAKNDKNESAKNDKQDS----------------NTQVINPPN-SGQ |
| NZ_LFKJ01000019_UM291_K1GA2V | YKDKPNNTN--SQSGAKNDKNESAKNDKQDS----------------NTQVINPPN-SGQ |
| LC185399_01-543_K1GA2V | YKDKPNNTN--SQSGAKNDKNESAKNDKQDS----------------NTQVINPPN-SGQ |
| LC185403_02-331_K1GA2V | YKDKSNNTN--SQSGAKNDKNESTKNDKQDS----------------NTQVINPPN-SGQ |
| LC185406_02-767_K1GA2V | YKDKPNNTN--SQSGAKNDKNESAKNDKQGS----------------NTQVINPPN-SGQ |
| LC185402_01-643_K1GA2V | YKDKPNNTN--SQSGAKNDKNESAKNDKQDS----------------NTQVINPPN-SGQ |
| 4451_Yangon222_K1GA2V | YKDKPNNTT--SQSGAKNDKNESAKNDKQDS----------------NTQVINPPN-SGQ |
| GQ331977_MZ1_K1GA2V | YKDKPNNTT--SQSGAKNDKNESAKNDKQDS----------------NTQVINPPN-SGQ |
| LC185388_00-37_K1GA2V | YKDKPNNTN--SQSGAKSDKNESAKNDKQDS----------------NTQVINPPN-SGQ |
| LC185419_10-442_K1GA2V | YKDKPNNTN--SQSGAKNDKNESAKNDKQDS----------------NTQVINPPN-SGQ |
| NZ_LEOV01000041_UM520_K1GA2V | YKDKPNNTN--SQSGAKNDKNESAKNDKQDS----------------NTQVINPPN-SGQ |
| NZ_AP014712_ML3_K1GA2V | YKDKPNNTN--SQSGAKNDKNESAKNDKQDS----------------NTQVINPPN-SGQ |
| LC185392_01-301_K1GA2V | YKDKPNNTN--SQSGAKSDKNESAKNDKQDS----------------NTQVINPPN-SGQ |
| LC185400_01-617_K1GA2V | YKDKPNNTN--SQSGAKSDKNESAKNDKQDS----------------NTQVINPPN-SGQ |
| AF049631_F52_K1GA2V | YKDKPNNTN--SQSGAKNDKNESAKNDKQDS----------------NTQVINPPN-SGQ |
| NC_017366_F32_K1GA2V | YKDKPNNTN--SQSGAKNDKNESAKNDKQDS----------------NTQVINPPN-SGQ |
| LC420356_Medan37_K1GA2V | YKDKPNNTN--SQSGAKNDKNESAKNDKQDS----------------NTQVINPPN-SGQ |
| LC420358_Medan40_K1GA2V | YKDKPNNTT--SQSGAKNDKNESAKNDKQDS----------------NTQVINPPN-SGQ |
| LC420364_Padang42_K1GA2V | YKDKPNNTN--SQSGAKNDKNESAKNDKQDS----------------NTQVINPPN-SGQ |
| NZ_MVTX01000007_HP15040_K1GA2V | YKDKPNNTN--SQSGAKSDKNESAKNDKQDS----------------NTQVINPPN-SGQ |
| 693_8A3_K1GA2V | YKDKPNNTN--SQSGAKNDKNESAKNDKQDS----------------NTQVINPPN-SGQ |
| NZ_CADC01000005_BCS100H1_K1GA2V | YKDKPNNTN--SQSGAKNDKNESAKNDKQDS----------------NTQVINPPN-SGQ |
| NZ_MVUP01000010_HP15011_K1GA2V | YKDKPNNTN--SQSGAKNDKNESAKNDKQDS----------------NTQVINPPN-SGQ |
| NZ_MVWX01000006_HP11059_K1GA2V | YKDKPNNTN--SQSGAKNDKNESAKNDKQDS----------------NTQVINPPN-SGQ |
| NZ_MVUV01000022_HP14069_K1GA2V | YKDKPNNTN--SQSGAKNDKNESAKNDKQDS----------------NTQVINPPN-SGQ |
| NZ_LJNZ02000005_UM229S_K1GA2V | YKDKPNNTN--SQSGAKNDKNESAKNDKQDS----------------NTQVINPPN-SGQ |
| NZ_LJXJ02000003_UM229R_K1GA2V | YKDKPNNTN--SQSGAKNDKNESAKNDKQDS----------------NTQVINPPN-SGQ |
| NZ_LJXM02000006_UM171R_K1GA2V | YKDKPNNTN--SQSGAKNDKNESAKNDKQDS----------------NTQVINPPN-SGQ |
| NZ_MJNA01000009_H30_K1GA2V | YKDKPNNTN--SQSGAKNDKNESAKNDKQDS----------------NTQVINPPN-SGQ |
| NZ_MJNB01000004_H30A_K1GA2V | YKDKPNNTN--SQSGAKNDKNESAKNDKQDS----------------NTQVINPPN-SGQ |
| NZ_MJGH01000008_178_K1GA2V | YKDKPNNTN--SQSGAKNDKNESAKNDKQDS----------------NTQVINPPN-SGQ |
| NZ_MJMT01000013_178A_K1GA2V | YKDKPNNTN--SQSGAKNDKNESAKNDKQDS----------------NTQVINPPN-SGQ |
| NZ_JH791774_HLJHP193_K1GA2V | YKDKPNNTN--SQSGAKNDKNESAKNDKQDS----------------NTQVINPPN-SGQ |
| AF361702_CHN5038c_K1GA2V | YKDKPNNTN--SQSGAKNDKNESAKNDKQDS----------------NTQVINPPN-SGQ |
| NZ_CP034071_Hpbs1_K1GA2V | YKDKPNNTN--SQSGAKNDKNESAKNDKQDS----------------NTQVINPPN-SGQ |
| AB190971_OK118_K1GA2V | YKDKPNNTN--SQSGAKNDKNESAKNDKQDS----------------NTQVINPPN-SGQ |
| LC185425_14-200_K1GA2V | YKDKPNNTN--SQSGAKNDKNESAKNDKQDS----------------NTQVINPPN-SGQ |
| NZ_LFKK01000018_UM352_K1GA2V | YKDKPNNTN--SQSGAKSDKNESAKNDKQDS----------------NTQVINPPN-SGQ |
| NZ_AUSM01000026_UM065_K1GA2V | FKDKPNNTN--SQSGAKNDKNESAKNDKQDS----------------NTQVINPPN-SGQ |
| LC185423_11-9_K1GA2V | YKNKTDNTT--SQSGAKNDKNESAKNDKQDS----------------NTQVINPPN-SGQ |
| AF049633_F56_K1GA2V | YKDKPNNTN--SQSGAKSDKNESAKNDKQDS----------------NTQVINPPN-SGQ |
| LC185414_09-294_K1GA2V | YKDKPNNTT--SQSGAKNDKNESTKNDKQDS----------------NTQVINPPN-SGQ |
| NZ_AP017358_MKF8_K1GA2V | YKDKSNNTN--SQSGAKNDKNESAKNDKQDS----------------NTQVINPPN-SGQ |
| LC185420_10-447_K1GA2V | YKDKPNNTN--SQSGAKSDKNESAKNDKQDS----------------NTQVINPPN-SGQ |
| NZ_AP017331_F18_K1GA2V | YKDKPNNTT--SQSGAKSDKNESAKNDKQDS----------------NTQVINPPN-SGQ |
| LC185421_10-453_K1GA2V | YKDKPNNTN--SQSGAKSDKNESAKNDKQDS----------------NTQVINPPN-SGQ |
| LC185404_02-425_K1GA2V | YKDNLNNTN--SQSGAKNDKNESAKNDKQDS----------------NTQVINPPN-SGQ |
| NZ_CP023448_HPJP26_K1GA2V | YKDKPNNTN--SQSGAKNDKNESAKNDKQDS----------------NTQVINPPN-SGQ |
| LC185412_05-423_K1GA2V | YKDKPNNTN--SQSGAKNDKNESAKNDKQDS----------------NTQVINPPN-SGQ |
| NZ_CP011483_DU15_K1GA2V | YKDKPNNTN--SQSGAKNDKNESAKNDKQDS----------------NTQVVNPPN-SGQ |
| NZ_CP025474_H-137_K1GA2V | YKDKPNNTN--SQSGAKNDKNESAKNDKQDS----------------NTQVINPPN-SGQ |
| NZ_AP017334_F210_K1GA2V | YKDKPNNTN--SQSGAKNDKNESTKNDKQDS----------------NTQVINPPN-SGQ |
| NZ_AP017336_F21_K1GA2V | YKDKPNNTN--SQSGAKNDKNESAKNDKQDS----------------NTQVINPPN-SGQ |
| LC185418_10-416_K1GA2V | YKDKPNNTN--SQSGAKNDKNESAKNDKQDS----------------NTQVINPPN-SGQ |
| AF050328_CHN5060d_K1GA2V | YKDKPNNTN--SQSGAKNDKNESAKNDKQDS----------------NTQVINPPN-SGQ |
| NC_017368_F16_K1GA2V | YKDKPNNTN--SQSGAKNDKNESAKNDKQDS----------------NTQVINPPN-SGQ |
| LC185417_10-358_K1GA2V | YKDKPNNTN--SQSGAKNDKNESAKNDKQDS----------------NTQVINPPN-SGQ |
| NZ_AP017350_F72_K1GA2V | YKDKPNNTN--SQSGAKNDKNESAKNDKQDS----------------NTQVINPPN-SGQ |
| RJGE01000015_ZH58_K1GA2V | YKDKPNNTN--SQSGAKSDKNESTKNDKQDS----------------NTQVINPPN-SGQ |
| AB190959_F15_K1GA2V | YKDKPNNTN--SQSGAKNDKNESAKNDKQDS----------------NTQVINPPN-SGQ |
| NZ_AP017355_F94_K1GA2V | YKDKPNNTN--SQSGAKSDKNESAKNDKQDS----------------NTQVINPPN-SGQ |
| NZ_AP017341_F38_K1GA2V | YKDKPNNTN--SQSGAKSDKNESAKNDKQDS----------------NTQVINPPN-SGQ |
| NZ_AP017360_MKM5_K1GA2V | YKDKPNNTN--SQSGAKSDKNESAKNDKQDS----------------NTQVINPPN-SGQ |
| AF049647_F64_K1GA2V | YKDKPNNTN--SQSGAKNDKNESAKNDKQDS----------------NTQVINPPN-SGQ |
| AF049649_F69_K1GA2V | YKDKPNNTN--SQSGAKSDKNESAKNDKQDS----------------NTQVINPPN-SGQ |
| NZ_MVUD01000015_HP15032_K1GA2V | YKDKPNNTN--SQSGAKNDKNESAKNDKQDS----------------NTQVINPPN-SGQ |
| NZ_AP017345_F55_K1GA2V | YKDKPNNTN--SQSGAKSDKNESAKNDKQDS----------------NTQVINPPN-SGQ |
| AF049645_F61_K1GA2V | YKDKPNNTN--SQSGAKSDKNESAKNDKQDS----------------NTQVINPPN-SGQ |
| LC185391_01-23_K1GA2V | YKDKPNNTN--SQSGTKSDKNESAKNDKQDS----------------NTQVINPPN-SGQ |
| NC_017382_51_K1GA2V | YKDKPNNTN--SQSGAKNDKNESAKNDKQDS----------------NTQVINPPN-SGQ |
| NZ_AKNK01000001_CPY1313_K1GA2V | YKDKPNNTN--SQSGAKSDKNESAKNDKQDS----------------NTQVINPPN-SGQ |
| NZ_AKNP01000001_CPY6271_K1GA2V | YKDKPNNTN--SQSGAKNDKNESAKNDKQDS----------------NTQVINPPN-SGQ |
| LC185416_10-354_K1GA2V | YKDKPNNTN--SQSGAKNDKNESAKNDKQDS----------------NTQVINPPN-SGQ |
| LC185395_01-381_K1GA2V | YKDKPNNTN--SQSGAKSDKNESAKNDKQDS----------------NTQVINPPN-SGQ |
| AF049636_F65_K1GA2V | YKDKPNNTN--SQSGAKSDKNESAKNDKQDS----------------NTQVINPPN-SGQ |
| AF049628_F45_K1GA2V | YKDKPNNTN--SQSGAKSDKNESAKNDKQDS----------------NTQVINPPN-SGQ |
| NZ_AP017354_F90_K1GA2V | YKDKPNNTN--SQSGAKSDKNESAKNDKQDS----------------NTQVINPPN-SGQ |
| NZ_QBQM01000039_TN2GF4_K1GA2V | YKDKPNNTN--SQSGAKSDKNESAKNDKQDS----------------NTQVINPPN-SGQ |
| LC185408_03-2_K1GA2V | YKDKPNNTN--SQSGAKNDKNESAKNDKQDS----------------NTQVINPPN-SGQ |
| LC185410_04-140_K1GA2V | YKDKPNNTN--SQSGAKNDKNESAKNDKQDS----------------NTQVINPPN-SGQ |
| LC185411_04-303_K1GA2V | YKDKPNNTN--SQSGAKNDKNESAKNDKQDS----------------NTQVINPPN-SGQ |
| NZ_AP017352_F78_K1GA2V | YKDKPNNTN--SQSGAKNDKNESAKNDKQDS----------------NTQVINPPN-SGQ |
| NZ_AP017337_F23_K1GA2V | YKDKPNNTN--SQSGAKNDKNESAKNDKQDS----------------NTQVINPPN-SGQ |
| NC_017360_35A_K1GA2V | YKDKPNNTN--SQSGAKNDKNESAKNDKQDS----------------NTQVINPPN-SGQ |
| NZ_AKNN01000005_CPY6081_K1GA2V | YKDKPNNTN--SQSGAKSDKNESAKNDKQDS----------------NTQVINPPN-SGQ |
| LC185387_00-255_K1GA2V | YKDKPNNTN--SQSGAKNDKNESAKNDKQDS----------------NTQVINPPN-SGQ |
| LC185407_03-132_K1GA2SV | YKDKPNNTN--SQSGAKNDKNESAKNDKQES-----------SQNNSNTQVINPPNSSGQ |
| NZ_AKNM01000005_CPY3281_K1GA2V | YKDKTDNTT--SQSGAKNDKNESAKNDKQDS----------------NTQVINPPN-SGQ |
| AF049644_F43_K1GA2V | YKDKPNNTN--SQSGAKNDKNESAKNDKQDS----------------NTQVINPPN-SGQ |
| AF049629_F47_K1GA2V | YKDNLNNTN--SQSGAKSDKNESAKNDKQDS----------------NTQVINPPN-SGQ |
| NZ_AP017357_MKF3_K1GA2V | YKDKPNNTN--SQSGAKSDKNESAKNDKQDS----------------NTQVINPPN-SGQ |
| NZ_MVWM01000010_HP12064_K1GA2V | YKDKPNNTN--SQSGAKSDKNESAKNDKQDS----------------NTQVINPPN-SGQ |
| NZ_CP011482_L7_K1GA2V | YKDKPNNTN--SQSGAKNDKNESAKNDKQDS----------------NTQVINPPN-SGQ |
| RJHJ01000018_ZH96_K1GA1SV | YKDKPNNTN--SQSGTKNDKQES-------------------SQNNSNTQVINPPN-SGQ |
| CP003419_XZ274_K1GA2V | YKDKPNNTN--SQSGAKSDKNESAKNDKQDS----------------NTQVINPPN-SGQ |
| NZ_MVRZ01000023_HPJ165_K1GA2SV | YKDKPKDTPS--QNNPKNDKNESAKNDKQES-----------SQNNSNTQVINPPD-SPP |
| NZ_CP011485_ausabrJ05_K1N2SV2 | YKDKSEDTPS--QNSANNSQQNSANNSQQNSAQSNNGSSVINPPDSAQTSVINPPD-SPP |
| LC420361_Medan68_K1GA2V | YKDKPNNTN--SQSGAKNDKNESAKNDKQDS----------------NTQVINPPN-SGQ |
| NC_017739_Shi417_QGA2SV | YKNQTDNTT--SQSSAKNDKNESAKNDKQKS-----------SQDNSNTQVINPPN-SGQ |
| NZ_MJMW01000024_S380A_K1GA2SV | YKDKPNNTN--SQSGAKNDKNESAKNDKQES-----------SQNNSNTQVINPPN-SGQ |
| NZ_AKHR02000049_FD577_K1GA2V | YKDKPNNTN--SQSGAKNDKNESAKNDKQDS----------------NTQVINPPN-SGQ |
| NZ_AKHV02000095_GC26_K1GA2V | YKDKPNNTN--SQSGAKNDKNESAKNDKQDS----------------NTQVINPPN-SGQ |
| NZ_MVUU01000004_HP15002_K1GA2V | YKDKPNNTN--SQSGAKNDKNESAKNDKQDS----------------NTQVINPPN-SGQ |
| NZ_MKLV01000007_428_K1GA2V | YKDKPNNTN--SQSGAKNDKNESAKNDKQDS----------------NTQVINPPN-SGQ |
| AF191639_ch2_K1GA2SV | YKDKPNNTN--SQSGAKNDKNESAKNDKQES-----------SQNNSNTQVINPPN-SGQ |
| NZ_LJXL02000018_UM171S_K1GA2V | YKDKPNNTN--SQSGAKNDKNESAKNDKQDS----------------NTQVINPPN-SGQ |
| LC420369_Kolaka96_E | YENKTKDTP-------------------------------------------------AQ |
| LC420367_Kolaka79_E | YENKTKDTP-------------------------------------------------AQ |
| LC420368_Kolaka94_E | YENKTKDTP-------------------------------------------------AQ |
| NZ_QBRB01000064_GC43-HL_K2TNSV | YKDKPNDKP--SNTTQNN-----ANNNQQNS-----------AQNNSNTQVINPPN-STQ |
| NZ_MVYF01000023_HP03054_K2TNSV | YKDKPKDKP--SNTTQNN-----ANNNQQNS-----------AQNNSNTQVINPPN-SAQ |
| NZ_MVYH01000015_HP01330_K2TNSV | YKDKPKDKP--SNTTQNN-----ANNNQQNS-----------AQNNSNTQVINPPN-SAQ |
| LC420354_Medan36_K1GA2SV | YKDKPNNTN--SQSGAKNDKNESAKNDKQES-----------SQNNSNTQVINPPN-SGQ |
| 4463_NP05_278_K1GA2SV | YKDKPNNTP--SQSGAKNDKNESAKNDKQES-----------SQNNSNTQVINPPN-STQ |
| 4457_Yangon159_K1GA2V | YKDKPNNTP--SQSGAKNDKNESAKNDKQDS----------------NTQVINPPN-SGQ |
| RJEC01000013_ZH04_K1GA2SV | YKDKPNNTP--SQSGAKNDKNESAKNDKQES-----------SQNNSNTQVINPPN-SAQ |
| RJEE01000012_ZH06_K1GA2SV | YKDKPNNTP--SQSGAKNDKNESAKNDKQES-----------SQNNSNTQVINPPN-SAQ |
| RJEF01000011_ZH07_K1GA2SV | YKDKPNNTP--SQSGAKNDKNESAKNDKQES-----------SQNNSNTQVINPPN-SAQ |
| RJGC01000023_ZH56_K1GA2SV | YKDKPNNTP--SQSGAKNDKNESAKNDKQES-----------SQNNSNTQVINPPN-SAQ |
| NZ_PHMR01000018_KH7_K1GA2SV | YKDKPNNTP--SQSGAKNDKNESAKNDKQES-----------SQNNSNTQVINPPN-SAQ |
| GQ331974_PG218_K1GA2SV | YKDKPNNTP--SQSGAKNDKNESAKNDKQES-----------SQNNSNTQVINPPN-SGQ |
| LC187586_BH65_K1GA2V | YKDKPNNTP--SQSGTKNDKNESAKNDKQDS----------------NTQVINPPN-SAQ |
| LC187587_BH42_K1GA2SV | YKDKPNNTP--SQSGAKNDKNESAKNDKQES-----------SQNNSNTQVINPPN-STQ |
| NZ_AONJ01000011_NAK7_K1GA2V | YKDKPNNTP--SQSGAKNDKNESAKNDKQDS----------------NTQVINPPN-SGQ |
| NZ_MBJY01000038_22352_QGA2SV | YKNQTNNTP--SQSGAKNDKNESAKNDKQES-----------SQNNSNTQVINPPN-NTQ |
| 4546_Nic11_A_K2TNSV | YKDKPKDKP--SNTTQNN-----ANNNQQNS-----------AQNNSNTQVINPPN-SAQ |
| NZ_MBKC01000032_1077_K2TNSV | YKDKPKDKP--SNTTQNN-----ANNNQQNS-----------AQNNSNTQVINPPN-SAQ |
| NZ_QEGD01000019_VCT187-B122_K2TNSV | YKDKPKDKP--SNTTQNN-----ANNNQQNS-----------AQNNSNTQVINPPN-STQ |
| NZ_AKPA01000005_HpH-9_K1GA2SV | YKDKPNNTP--SQSGTKNDKNESAKNDKKES-----------SQNNSNTQVINPPN-SAQ |
| RJEJ01000016_ZH11_K1GA2SV | YKDKPNNTP--SQSGAKNDKNESAKNDKQES-----------SQNNSNTQVINPPN-SAQ |
| RJHU01000013_ZH108_QGA2SV | YKNQTNNTP--SQSGAKNDKNESAKNDKQES-----------SQNNSNTQVINPPS-SAQ |
| NZ_AKOQ01000009_HpH-45_K1GA2V | YKDKPNSTP--SQSGTKNDKNESAKNDKQQS-------------SNSNTEVINPPN-SVQ |
| 3663_3738_QGA2SV | YKNQTNNTP--SQSGAKNDKNESAKNDKQNS-----------AQNNSNTQVINPPN-SAQ |
| NC_011333_G27_K1GA2SV | YKDKPNNTP--SQSGTKNDKNESAKNDKQES-----------SQNNSNTQVINPPN-STQ |
| NZ_QEGL01000017_ECF139-B065_K1GA2SV | YKDKPNNTP--SQSGAKNDKNESAKNDKQES-----------SQNNSNTQVINPPN-SAQ |
| 8771_2011_41_K1GA2SV | YKDKPNNTP--SQSGTKNDKNESAKNDKQEI----------SQNNNSNTQVINPPN-STQ |
| NZ_MILH01000307_MG2011-41_K1GA2SV | YKDKPNNTP--SQSGTKNDKNESAKNDKQEI----------SQNNNSNTQVINPPN-STQ |
| 6290_Nic25_A_QGA2SV | YKNQTNNTP--SQSGTKNDKNESAKNDKQEI----------SQNNNSNTEVINPPN-NTQ |
| NC_014555_PeCan4_K2TNSV | YKDKPKDKP--SNTTQNN-----ANNNQQNS-----------AQNNSNTQVINPPN-SAQ |
| NC_017378_Puno120_K2TNSV | YKDKPKDKP--SNTTQNN-----ANNNQQNS-----------AQNNSNTQVINPPN-NTQ |
| NZ_QBRQ01000011_B29_K2TNSV | YKDKPKDKP--SNTTQNN-----ANNNQQNS-----------AQNNNNTQVINPPN-NTQ |
| NZ_QBRN01000053_B35_K1TNSV | YKDKPKEKP--SNTTQNN-----ANNNQQNS-----------AQNNSNTQVINPPN-SAQ |
| NZ_MBII01000020_22377_K2TNSV | YKDKPNDKP--SNTTQNN-----ANNNQQNS-----------AQNNSNTQVINPPN-SAQ |
| 8762_ms176_K2TNSV | YKDKPKDKP--SNTTQNN-----ANNNQQNS-----------AQNNSNTQVINPPN-SAQ |
| NZ_MILL01000035_MGms176_K2TNSV | YKDKPKDKP--SNTTQNN-----ANNNQQNS-----------AQNNSNTQVINPPN-SAQ |
| NZ_QBQJ01000001_GC69-HL_K2TNSV | YKDKPKDKP--SNTTQNN-----ANNNQQNS-----------TQNNSNTQVINPPN-SAQ |
| NZ_KB636380_GAM121Aii_K1GA1SV | YKDKPNSTT--SQSGTKNDKQEI------------------SQNNNNNTEVINPPN-NTQ |
| 639_Gambia94_24_K1GA1SV | YKDKPNNTP--SQSGTKNDKQEI------------------SQNNNSNTEVINPPN-NTQ |
| NC_017371_Gambia94/24_K1GA1SV | YKDKPNNTP--SQSGTKNDKQEI------------------SQNNNSNTEVINPPN-NTQ |
| NZ_KB644460_GAM120Ai_K1GA1SV | YKDKPNSTT--SQSGTKNDKQQI------------------SQNNNSNTEVINPPN-NTQ |
| NZ_AKOJ01000004_HpH-29_K1GA1SV | YKDKPNSTT--SQSGTKNDKQKI------------------SQNNNSNTEVINPPN-NTQ |
| NZ_CP007603_J166_K1GA1SV | YKDKPNSTT--SQSGTKNDKQEI------------------SQNNNSNTEVINPPN-NTQ |
| NC_017374_2017_K1GA1SV | YKDKPNSTT--SQSGTKNDKQEI------------------SQNNNSNTEVINPPN-NTQ |
| NC_017381_2018_K1GA1SV | YKDKPNSTT--SQSGTKNDKQEI------------------SQNNNSNTEVINPPN-NTQ |
| AF191643_AFN4769_K1GA1SV | YKDKPNSTT--SQSGTKNDKQEI------------------SQNNNSNTEVINPPN-NTQ |
| NZ_LR134517_NCTC13345_K1GA1SV | YKDKPNNTT--SQSGAKNDKQKI------------------SQNNNSNTEVINPPN-NTQ |
| NZ_QBQK01000046_3699_K1GA1SV | YKNKPNSTT--SQSGTKNDKQEI------------------SQNNNSNTEVINPPN-NTQ |
| NZ_AKOL01000008_HpH-36_K1GA1SV | YKDEPNSTT--SQSGTKNDKQEI------------------SQNNNSNTEVINPPN-NTQ |
| NZ_KB641801_GAM270ASi_K1GA1SV | YKDKPNSTT--SQSGAKNDKQQI------------------SQNNNSNTEVINPPN-NTQ |
| NZ_KB642119_GAM93Bi_K1GA1SV | YKDKPNSTT--SQSGTKNDKQEI------------------SQNNNSNTEVINPPN-NTQ |
| NZ_MVUB01000005_HP15034_K1GA1SV | YKDKPNSTT--SQSGTKNDKQEI------------------SQNNNSNTEVINPPN-NTQ |
| NZ_MBHK01000007_3125_K1GA1SV | YKDKPNNTT--SQSGTKNDKQQI------------------SQNNNSNTEVINPPN-NTQ |
| NZ_QBQN01000032_3824_K1GA1SV | YKDKPNSTT--SQSGTKNDKQEI------------------SQNNNSNTEVINPPN-NTQ |
| NZ_KB641843_GAM112Ai_K1GA1SV | YKDKPNNTT--PQSGTKNDKQEI------------------SQNNNSNTEVINPPN-NTQ |
| NZ_KB642143_GAM42Ai_K1GA1SV | YKDKPNNTT--PQSGTKNDKQEI------------------SQNNNSNTEVINPPN-NTQ |
| NZ_KB637030_GAM105Ai_K1A1SV | YKDKPNSTT--SPSGTKNDKQEI------------------SQNNNSNTEVINPPN-NTQ |
| NZ_KB641975_GAM80Ai_K1GA1SV | YKDKPNSTT--SQSGTKNDKQQI------------------SQNNNSNTEVINPPN-NTQ |
| NZ_KB636250_GAM246Ai_K1GA1SV | YKDKPNSTT--SQSGTKNDKQEI------------------SQNNNSNTEVINPPN-NTQ |
| 4565_Nic20_C_K1GA1SV | YKDKPNNTT--SQSGTKNDKQEI------------------SQNNNSNTEVINPPN-NTQ |
| NZ_AKOP01000010_HpH-44_K1GA1SV | YKDKPNNTT--SQSGTKNDKQEI------------------SQNNNSNTEVINPPN-NTQ |
| NZ_AKOB01000006_HpA-5_K1GA1SV | YKDKPNNTT--SQSGTKNDKQEI------------------SQNNNSNTEVINPPN-NTQ |
| NZ_AKOU01000005_HpA-16_K1GA1SV | YKDKPNNTT--SQSGTKNDKQEI------------------SQNNNSNTEVINPPN-NTQ |
| NZ_AKOX01000008_HpH-3_K1GA1SV | YKDKPNSTT--SQSGTKNDKQEI------------------SQNNNSNTEVINPPN-NTQ |
| NZ_AKOF01000005_HpH-16_K1GA1SV | YKDKPNSTT--SQSGAKNDKQQI------------------SQNNNSNTEVINPPN-NTQ |
| NZ_AKQF01000006_HpH-5b_K1GA1SV | YKDKPNSTT--SQSGTKNDKQKI------------------SQNNNSNTEVINPPN-NTQ |
| NZ_MTWS01000016_SV397_2_K1GA1SV | YKDKPNSTT--SQSGTKNDKQEI------------------SQNNNSNTEVINPPN-NTQ |
| NZ_MVYO01000016_HP01102_K1GA1SV | YKDKPNSTT--PQSGAKNDKQEI------------------SQNNNSNTEVINPPN-NTQ |
| NZ_AKOK01000006_HpH-30_K1GA1SV | YKDKPNSTT--SQSGTKNDKQEI------------------SQNNNSNTEVINPPN-NTQ |
| NZ_MVXV01000002_HP06045_K1GA1SV | YKDKPNNTT--SQSGAKNDKQEI------------------SQNNNSNTEVINPPN-SGQ |
| NZ_MTWK01000004_PZ5006_3A3_K1GA1SV | YKDKPNSTT--SQSGTKNDKQQI------------------SQNNNSNTEVINPPN-NTQ |
| NZ_AKPL01000004_HpP-4_K1GA1SV | YKDKPNSTT--SQSGTKNDKQAI------------------SENNNSNTEVINPPN-NTQ |
| NZ_AKQD01000004_HpP-4c_K1GA1SV | YKDKPNSTT--SQSGTKNDKQAI------------------SENNNSNTEVINPPN-NTQ |
| NZ_AKQE01000005_HpP-4d_K1GA1SV | YKDKPNSTT--SQSGTKNDKQAI------------------SENNNSNTEVINPPN-NTQ |
| NZ_MVTR01000007_HP15059_K1GA1SV | YKDKPNSTT--SQSGTKNDKQEI------------------SQNNNSNTEVINPPN-NTQ |
| NZ_AKOS01000004_HpA-8_K1GA1SV | YKDKPNSTT--SQSGTKNDKQEI------------------SQNNNSNTEVINPPN-NTQ |
| AKPO01000006_HpP-13_K1GA1SV | YKDKPNSTT--SQSGAKNDKQEI------------------SQNNNSNTEVINPPN-NTQ |
| NZ_AKQI01000006_HpP-13b_K1GA1SV | YKDKPNSTT--SQSGAKNDKQEI------------------SQNNNSNTEVINPPN-NTQ |
| RJHI01000003_ZH5_K1GA1SV | YKDKPNSTT--SQSGTKNDKQEI------------------SQNNNSNTEVINPPN-NTQ |
| NZ_MTWJ01000026_PZ5005_3A3_K1GA1SV | YKDKPNSTT--SQSGTKNDKQEI------------------SQNNNSNTEVINPPN-NTQ |
| NZ_CP011484_CC33C_K1GA1V | YKDKPNSTT--SQSSTKNDKQQS---------------------SNSNTEVINPPN-NTQ |
| NZ_CBNP010000003_SA252A_K1GA1SV | YKDKPNNTT--SQSGTKNDKQEI------------------SQNNNSNTEVINPPN-NTQ |
| 1424_SA227A_K1GA1SV | YKDKPNGTT--SQSGTKNDKQKI------------------SQNNNSNTEVINPPN-NTQ |
| NZ_CBNL010000021_SA227C_K1GA1SV | YKDKPNGTT--SQSGTKNDKQKI------------------SQNNNSNTEVINPPN-NTQ |
| NZ_MVVI01000003_HP13072_K1GA1SV | YKDKPNSTT--SQSGTKNDKQEI------------------SQNNNSNTEVINPPN-NTQ |
| NZ_MVTG01000017_HP99216_K1GA1SV | YKDKPNSTT--SQSGTKNDKQEI------------------SQNNNSNTEVINPPN-NTQ |
| NZ_MVXT01000002_HP06059_K1GA1SV | YKDKPNSTT--SQSGTKNDKQEI------------------SQNNNSNTEVINPPN-NTQ |
| NZ_AKPK01000005_HpP-3_K1GA1SV | YKDKPNSTT--SQSGAKNDKQEI------------------SQNNNSNTEVINPPN-NTQ |
| NZ_AKQC01000004_HpP-3b_K1GA1SV | YKDKPNSTT--SQSGAKNDKQEI------------------SQNNNSNTEVINPPN-NTQ |
| 1455_SA46C_K1GA1SV | YKDKPNSTT--SQSGTKNDKQEI------------------SQNNNSNTEVINPPN-NTQ |
| NZ_CBNV010000013_SA46A_K1GA1SV | YKDKPNSTT--SQSGTKNDKQEI------------------SQNNNSNTEVINPPN-NTQ |
| NZ_AKOM01000004_HpH-41_K1GA1SV | YKDKPNSTT--SQSGTKNDKQKI------------------SQNNNSNTEVINPPN-NTQ |
| NZ_MVXK01000015_HP09046_K1GA1SV | YKDKPNSTT--SQSGTKNDKQQI------------------SQNNNSNTEVINPPN-NTQ |
| NZ_MVWC01000012_HP13011_K1GA1SV | YKDKPNSTT--SQSGTKNDKQEI------------------SQNNNSNTEVINPPN-NTQ |
| NZ_QBQQ01000028_3755_K1GA1SV | YKDKPNSTT--SQSGTKNDKQEI------------------SQNNNSNTEVINPPN-NTQ |
| NZ_QBQP01000176_3770_K1GA1SV | YKDKPNSTT--SQSGTKNDKQKI------------------SQNNNSNTEVINPPN-NTQ |
| NZ_CBQL010000004_SA170C_K1GA1SV | YKDKPNSTT--SQSGTKNDKQEI------------------SQNNNSNTEVINPPN-NTQ |
| NZ_CBPZ010000002_SA146A_K1GA1SV | YKDKPNSTT--SQSGTKNDKQEI------------------SQNNNSNTEVINPPN-NTQ |
| NZ_MVTC01000002_HP99330_K1GA1SV | YKDKPNSTT--SQSGTKNDKQEI------------------SQNNNSNTEVINPPN-NTQ |
| NZ_CBNB010000004_SA45A_K1GA1SV | YKDKPNSTT--SQSGTKNDKQEI------------------SQNNNSNTEIINPPN-NTQ |
| NZ_CBPQ010000003_SA30A_K1GA1SV | YKDKPNSTT--SQSGTKNDKQEI------------------SQNNNSNTEVINPPN-NTQ |
| NZ_CBQB010000006_SA30C_K1GA1SV | YKDKPNSTT--SQSGTKNDKQEI------------------SQNNNSNTEVINPPN-NTQ |
| NZ_CBOL010000004_SA158A_K1GA1SV | YKDKPNSTT--SQSGTKNDKQEI------------------SQNNNSNTEVINPPN-NTQ |
| NZ_CBOQ010000005_SA210C_K1GA1SV | YKDKPNSTT--SQSGTKNDKQEI------------------SQNNNSNTEVINPPN-NTQ |
| NZ_CBOW010000005_SA163C_K1GA1SV | YKDKPNSTT--SQSGTKNDKQEI------------------SQNNNSNTEVINPPN-NTQ |
| NZ_CBPL010000004_SA300C_K1GA1SV | YKDKPNSTT--SQSGTKNDKQEI------------------SQNNNSNTEVINPPN-NTQ |
| NZ_CBPM010000007_SA31C_K1GA1SV | YKDKPNSTT--SQSGTKNDKQEI------------------SQNNNSNTEVINPPN-NTQ |
| NZ_CBPY010000003_SA158C_K1GA1SV | YKDKPNSTT--SQSGTKNDKQEI------------------SQNNNSNTEVINPPN-NTQ |
| NZ_CBNQ010000022_SA162C_K1GA1SV | YKDKPNSTT--SQSGTKNDKQEI------------------SQNNNSNTEVINPPN-NTQ |
| NZ_CBOS010000022_SA162A_K1GA1SV | YKDKPNSTT--SQSGTKNDKQEI------------------SQNNNSNTEVINPPN-NTQ |
| NZ_CBNE010000015_SA35A_K1GA1SV | YKDKPNSTT--SQSGTKNDKQEI------------------SQNNNSNTEVINPPN-NTQ |
| NZ_CBNU010000015_SA35C_K1GA1SV | YKDKPNSTT--SQSGTKNDKQEI------------------SQNNNSNTEVINPPN-NTQ |
| NZ_CBNF010000002_SA157A_K1GA1SV | YKDKPNSTT--SQSGTKNDKQEI------------------SQNNNSNTEVINPPN-NTQ |
| NZ_KB642274_HP116Bi_K1GA1SV | YKDKPNSTT--SQSGTKNDKQEI------------------SQNNNSNTEVINPPN-NTQ |
| NZ_AKPI01000003_HpP-1_K2TNSV | YKDKPKDKP--SNTTQNN-----ANNNQQNS-----------AQNNSNTQVINPPN-NTQ |
| NZ_AKQA01000006_HpP-1b_K2TNSV | YKDKPKDKP--SNTTQNN-----ANNNQQNS-----------AQNNSNTQVINPPN-NTQ |
| NZ_AKQK01000004_HpP-25c_K2TNSV | YKDKPKDKP--SNTTQNN-----ANNNQQNS-----------AQNNSNTEVINPPN-NTQ |
| NZ_AKQL01000004_HpP-25d_K2TNSV | YKDKPKDKP--SNTTQNN-----ANNNQQNS-----------AQNNSNTEVINPPN-NTQ |
| AF191644_AFN4847_K1GA1SV | YKDKPNSTT--PQSGTKNDKQEI------------------SQNNNSNTEVINPPN-NTQ |
| AF191642_AFN4124_K1GA1SV | YKDKPNNTP--SQSGTKNDKQEI------------------SQNNNSNTEVINPPN-NTQ |
| NZ_KB635961_GAM250T_K1GA1SV | YKDKPNSTT--SQSGTKNDKQEI------------------SQNNNSNTEVINPPN-NTQ |
| NZ_KB635999_GAM250AFi_K1GA1SV | YKDKPNSTT--SQSGTKNDKQEI------------------SQNNNSNTEVINPPN-NTQ |
| NZ_KB636035_HP250BFiV_K1GA1SV | YKDKPNSTT--SQSGTKNDKQEI------------------SQNNNSNTEVINPPN-NTQ |
| NZ_KB636113_HP250ASii_K1GA1SV | YKDKPNSTT--SQSGTKNDKQEI------------------SQNNNSNTEVINPPN-NTQ |
| NZ_KB636170_HP250BFi_K1GA1SV | YKDKPNSTT--SQSGTKNDKQEI------------------SQNNNSNTEVINPPN-NTQ |
| NZ_KB636234_HP250ASi_K1GA1SV | YKDKPNSTT--SQSGTKNDKQEI------------------SQNNNSNTEVINPPN-NTQ |
| NZ_KB636441_GAM252Bi_K1GA1SV | YKDKPNSTT--SQSGTKNDKQEI------------------SQNNNSNTEVINPPN-NTQ |
| NZ_KB636470_HP250BFii_K1GA1SV | YKDKPNSTT--SQSGTKNDKQEI------------------SQNNNSNTEVINPPN-NTQ |
| NZ_KB636562_GAM252T_K1GA1SV | YKDKPNSTT--SQSGTKNDKQEI------------------SQNNNSNTEVINPPN-NTQ |
| NZ_KB642432_HP250AFii_K1GA1SV | YKDKPNSTT--SQSGTKNDKQEI------------------SQNNNSNTEVINPPN-NTQ |
| NZ_KB642581_HP250AFiii_K1GA1SV | YKDKPNSTT--SQSGTKNDKQEI------------------SQNNNSNTEVINPPN-NTQ |
| NZ_KB642745_HP250AFiV_K1GA1SV | YKDKPNSTT--SQSGTKNDKQEI------------------SQNNNSNTEVINPPN-NTQ |
| NZ_KB642919_HP250BFiii_K1GA1SV | YKDKPNSTT--SQSGTKNDKQEI------------------SQNNNSNTEVINPPN-NTQ |
| NZ_KB644294_HP250BSi_K1GA1SV | YKDKPNSTT--SQSGTKNDKQEI------------------SQNNNSNTEVINPPN-NTQ |
| NZ_KB636645_GAM260ASi_K1GA1SV | YKDKPNNTT--SQSGTKNDKQEI------------------SQNNNSNTEVINPPN-NTQ |
| NZ_KB642504_HP260AFii_K1GA1SV | YKDKPNNTT--SQSGTKNDKQEI------------------SQNNNSNTEVINPPN-NTQ |
| NZ_KB642663_GAM268Bii_K1GA1SV | YKDKPNNTT--SQSGTKNDKQEI------------------SQNNNSNTEVINPPN-NTQ |
| NZ_KB642719_HP260ASii_K1GA1SV | YKDKPNNTT--SQSGTKNDKQEI------------------SQNNNSNTEVINPPN-NTQ |
| NZ_KB644225_HP260AFi_K1GA1SV | YKDKPNNTT--SQSGTKNDKQEI------------------SQNNNSNTEVINPPN-NTQ |
| NZ_CBNH010000001_SA226A_K1GA2V | YKDKPNNTT--SQSGAKNDKNESAKNDKQQS-------------SNSNTEVINPPN-NTQ |
| 751_GAMchjs136i_K1GA1SV | YKDKPNSTT--SQSGAKNDKQQI------------------SQNNNSNTEVINPPN-NTQ |
| NZ_KB644675_GAMchJs136i_K1GA1SV | YKDKPNSTT--SQSGAKNDKQQI------------------SQNNNSNTEVINPPN-NTQ |
| 8692_2004_20_K1GA1SV | YKDKPNSTT--SQSGAKNDKQEI------------------SQNNNSNTEVINPPN-NTQ |
| NZ_MILQ01000038_MM2004-20_K1GA1SV | YKDKPNSTT--SQSGAKNDKQEI------------------SQNNNSNTEVINPPN-NTQ |
| NZ_AKOG01000006_HpH-24_K1GA1SV | YKDKPNSTT--PQSGTKNDKQEI------------------SQNNNSNTEVINPPN-SAQ |
| NZ_CP011330_J99_K1GA1SV | YKDKPNSTT--SQSGTKNDKKEI------------------SQNNNSNTEVINPPN-NTQ |
| NZ_CP027404_FDAARGOS_300_K1GA1SV | YKDKPNSTT--SQSGTKNDKKEI------------------SQNNNSNTEVINPPN-NTQ |
| NZ_MTLF01000013_HP725g_K1GA1SV | YKDKPNSTT--SQSGTKNDKKEI------------------SQNNNSNTEVINPPN-NTQ |
| NZ_MUHJ01000014_HP_PWs_K1GA1SV | YKDKPNSTT--SQSGTKNDKKEI------------------SQNNNSNTEVINPPN-NTQ |
| NZ_MWQM01000093_HP_106_K1GA1SV | YKDKPNSTT--SQSGTKNDKKEI------------------SQNNNSNTEVINPPN-NTQ |
| NZ_MWUG01000109_G4_K1GA1SV | YKDKPNSTT--SQSGTKNDKKEI------------------SQNNNSNTEVINPPN-NTQ |
| NZ_CBNT010000002_SA301C_K1GA1SV | YKDKPNNTT--SQSGTKNDKQEI------------------SQNNNSNTEVINPPN-NTQ |
| NZ_CBPH010000005_SA301A_K1GA1SV | YKDKPNNTT--SQSGTKNDKQEI------------------SQNNNSNTEVINPPN-NTQ |
| NZ_AKON01000007_HpH-42_K1GA1SV | YKDKPNSTT--SQSGTKNDKQQI------------------SQNNNSNTEVINPPN-NTQ |
| NZ_KB635862_HP260Bi_K1GA1SV | YKDKPNNTT--PQSGTKNDKQEI------------------SQNNNSNTEVINPPN-NTQ |
| NZ_KB636865_GAM260Bi_K1GA1SV | YKDKPNNTT--PQSGTKNDKQEI------------------SQNNNSNTEVINPPN-NTQ |
| NZ_QBQB01000199_462_K1GA1SV | YKDKPNSTT--SQSGTKNDKQEI------------------SQNNNSNTEVINPPN-NTQ |
| AF191645_AFNG114_K1GA1SV | YKDKPNSTT--SQSGTKNDKQEI------------------SQNNNSNTEVINPPN-NTQ |
| NZ_KB642830_GAM210Bi_K1GA1SV | YKDKPNSTT--SQSGTKNDKQEI------------------SQNNNSNTEVINPPN-NTQ |
| NZ_KB636083_GAM249T_K1GA1SV | YKDKPNSTT--SQSGAKNDKQEI------------------SQNNNSNTEVINPPN-NTQ |
| NZ_AOTX01000028_HpH-1_K1GA1SV | YKDKPNSTT--SQSGTKNDKQEI------------------SQNNNSNTEVINPPN-NTQ |
| 6283_Nic29_A_K1GA1SV | YKDKPNNTT--PQSGTKNDKQEI------------------SQNNNSNTEVINPPN-NTQ |
| RJGN01000010_ZH68_K1GA1SV | YKDKPNSTT--SQSGTKNDKQQI------------------SQNNNSNTEVINPPN-NTQ |
| NZ_LT635458_HE143/09_K1GA1SV | YKDKPNNTT--SQSGTKNDKQKI------------------SQNNNSNTEVINPPN-NTQ |
| NZ_LT635459_HE132/09_K1GA1SV | YKDKPNNTT--SQSGTKNDKQKI------------------SQNNNSNTEVINPPN-NTQ |
| NZ_LT635471_HE141/09_K1GA1SV | YKDKPNNTT--SQSGTKNDKQKI------------------SQNNNSNTEVINPPN-NTQ |
| NZ_LT635472_HE170/09_K1GA1SV | YKDKPNNTT--SQSGTKNDKQKI------------------SQNNNSNTEVINPPN-NTQ |
| NZ_LT635473_HE136/09_K1GA1SV | YKDKPNNTT--SQSGTKNDKQKI------------------SQNNNSNTEVINPPN-NTQ |
| NZ_LT635474_HE171/09_K1GA1SV | YKDKPNNTT--SQSGTKNDKQKI------------------SQNNNSNTEVINPPN-NTQ |
| NZ_LT635476_HE134/09_K1GA1SV | YKDKPNNTT--SQSGTKNDKQKI------------------SQNNNSNTEVINPPN-NTQ |
| NZ_LT635477_HE147/09_K1GA1SV | YKDKPNNTT--SQSGTKNDKQKI------------------SQNNNSNTEVINPPN-NTQ |
| NZ_LT635478_HE142/09_K1GA1SV | YKDKPNNTT--SQSGTKNDKQKI------------------SQNNNSNTEVINPPN-NTQ |
| NZ_LT837687_BCM-300_K1GA1SV | YKDKPNNTT--SQSGTKNDKQKI------------------SQNNNSNTEVINPPN-NTQ |
| NZ_KB642385_GAM114Ai_K1GA1SV | YKDKPNSTT--SQSGTKNDKQEI------------------SQNNNSNTEVINPPN-NTQ |
| NZ_CBPR010000002_SA45C_K1GA1SV | YKDKPNSTT--SQSGTKNDKQEI------------------SQNNNSNTEIINPPN-NTQ |
| NZ_AKPF01000003_HpH-21_K1GA1SV | YKDKPNSTT--SQSGTKNDKQEI------------------SQNNNSNTEVINPPN-NTQ |
| NZ_QBRK01000048_B41_K1GA1SV | YKDKPNSTT--SQSGTKNDKQEI------------------SQNNNSNTEVINPPN-NTQ |
| NZ_KB644569_GAM119Bi_K1GA1SV | YKDKPNSTT--SQSGTKNDKQQI------------------SQNNNSNTEVINPPN-NTQ |
| NZ_AKPT01000006_HpP-26_K1GA1SV | YKDKPNSTT--SQSGTKNDKQEI------------------SQNNNSNTEVINPPN-NTQ |
| NZ_KE698743_GAM117Ai_K1GA1SV | YKDKPNSTT--SQSGTKNDKQQI------------------SSNNNSNTEVINPPN-NTQ |
| NZ_AKPN01000006_HpP-11_K1GA1SV | YKDKPNNTT--SQSGAKNDKQKI------------------SQNNNSNTEVINPPN-NTQ |
| NZ_AKPG01000006_HpH-23_K1GA1SV | YKDKPNSTT--SQSGTKNDKQEI------------------SQNNNSNTEVINPPN-NTQ |
| NZ_AKOR01000004_HpA-6_K1GA1SV | YKDKPNSTT--SQSGTKNDKQEI------------------SQNNNSNTEVINPPN-NTQ |
| NZ_AKOY01000004_HpH-4_K1GA1SV | YKDKPNSTT--SQSGTKNDKQEI------------------SQNNNSNTEVINPPN-NTQ |
| NZ_MVXN01000006_HP08072_K1GA1SV | YKDKPNSTT--SQSGTKNDKQEI------------------SQNNNSNTEVINPPN-NTQ |
| NZ_MVYS01000018_HP00152_K2TNSV | YKDKPKDKP--SNTTQNN-----ANNNQQNS-----------AQNNSNTQVINPPN-SAQ |
| 3629_SSR23_K2TNSV | YKDKPKDKP--SNTTQNN-----ANNNQQNS-----------AQNNSNTQVINPPN-SAQ |
| NZ_MVYK01000018_HP01306_K2TNSV | YKDKPKDKP--SNTTQNN-----ANNNQQNS-----------AQNNSNTQVINPPN-SAQ |
| NZ_MVYC01000017_HP04041_K2TNSV | YKDKPKDKP--SNTTQNN-----ANNNQQNS-----------AQNNSNTQVINPPN-SAQ |
| NZ_MVYQ01000002_HP00248_K2TNSV | YKDKPKDKP--SNTTQNN-----ANNNQQNS-----------AQNNSNTQVINPPN-SAQ |
| HPAF001358_ATCC43526_K2TNSV | YKDKPKDKP--SNTTQNN-----ANNNQQNS-----------AQNNNNTQVINPPN-SAQ |
| NZ_CP028325_FDAARGOS_298_K2TNSV | YKDKPKDKP--SNTTQNN-----ANNNQQNS-----------AQNNNNTQVINPPN-SAQ |
| NZ_LS483488_NCTC11637_K2TNSV | YKDKPKDKP--SNTTQNN-----ANNNQQNS-----------AQNNNNTQVINPPN-SAQ |
| 3640_CHA_185_K2TNSV | YKDKPKDKP--SNTTQNN-----ANNNQQNS-----------AQNNSNTQVINPPN-SAQ |
| RJGU01000003_ZH78_K2TA1SV | YKDKPKDKP--SNTTQNN-----AKNDKQNS-----------AQNNNNTSVINPPN-SAQ |
| NZ_AKNS01000005_NQ4200_K1GANSV | YKDKPNNTP--SQSGTKNDKNESAKNNQQNS-----------TQNNSNTQVINPPN-SAQ |
| NZ_QBQG01000047_31235_K2TNSV | YKDKPKDKP-------SNTTQNNANNNQQNS-----------TQNNSNTQVINPPN-SAQ |
| NZ_CADI01000010_NQ392_K2TNSV | YKDKPKDKP-------SNTTQNNANNNQQNS-----------AQNNSNTQVINPPN-SAQ |
| 8700_2006_407_K2TNSV | YKDKPKDKP-------SNTTQNNANNNQQNS-----------AQNNSNTYVINPPN-SAQ |
| NZ_MILF01000056_MG2006-407_K2TNSV | YKDKPKDKP-------SNTTQNNANNNQQNS-----------AQNNSNTYVINPPN-SAQ |
| 8698_2006_52_K2TNSV | YKDKPKDKP-------SNTTQNNANNNQQNS-----------AQNNSNTQVINPPN-STQ |
| NZ_MIKS01000003_MC2006-52_K2TNSV | YKDKPKDKP-------SNTTQNNANNNQQNS-----------AQNNSNTQVINPPN-STQ |
| 8715_ms1080_K2TNSV | YKDKPKDKP-------SNTTQNNANNNQQNS-----------AQNNSNTQVINPPN-SAQ |
| NZ_MIKY01000036_MCms1080_K2TNSV | YKDKPKDKP-------SNTTQNNANNNQQNS-----------AQNNSNTQVINPPN-SAQ |
| NZ_JSXV01000026_1846/05_K2TNSV | YKDKPKDKP-------SNTTQNNANNNQQNS-----------AQNNSNTQVINPPN-SAQ |
| NZ_QDJE01000014_B659-C2_K2TNSV | YKDKPKDKP-------SNTTQNNANNNQQNS-----------AQNNSNTQVINPPN-NTQ |
| NZ_QDJF01000016_B659-A1_K2TNSV | YKDKPKDKP-------SNTTQNNANNNQQNS-----------AQNNSNTQVINPPN-NTQ |
| 4532_Nic04_A_K3TNSV | YKDKPKDKPKDKP---SNTTQNNANNNQQNS-----------AQNNSNTQVINPPN-SAQ |
| NC_017362_Lithuania75_K1GA2V | YKDKPNNTP--SQSGAKNDKNESAKNDKQQS-----------S--NSNTEVINPPN-SAQ |
| NZ_QBPG01000018_9:1_single_QGA2SV | YKNQTNNTP--SQSGAKNDKNESAKNDKQES-----------SQNNSNTQVINPPN-STQ |
| NZ_QEHA01000020_B362_QGA2SV | YKNQTNNTP--SQSGTKNDKNESAKNDKQES-----------SQNNSNTQVINPPN-SAQ |
| 8683_ms167_K1GA2SV | YKDKPNNTP--SQSGAKNDKNESAKNDKQES-----------SQNNSNTQVINPPN-SAQ |
| NZ_MILK01000006_MGms167_K1GA2SV | YKDKPNNTP--SQSGAKNDKNESAKNDKQES-----------SQNNSNTQVINPPN-SAQ |
| HPU05676_60190(ATCC49_K1GA1SV | YKDKPNNTP--SQSGAKNDKQES-------------------SQNNSNTQVINPPN-STQ |
| NZ_MVXW01000016_HP06038_K1GA2V | YKDKPNNTT--PQSGTKNDKNESAKNDKQQS-----------S--NSNTQVINPPN-SAQ |
| NZ_QEHE01000018_B314_K1GA2SV | YKDKPNNTP--SQSGAKNDKNESAKNDKQES-----------SQNNSNTQVINPPN-STQ |
| NZ_MBHV01000021_26100_K1GA2SV | YKDKPNNTP--SQSGAKNDKQESAKNDKQES-----------SQNNSNTQVINPPN-STQ |
| NZ_MUOT01000027_CC26100_K1GA2SV | YKDKPNNTP--SQSGAKNDKQESAKNDKQES-----------SQNNSNTQVINPPN-STQ |
| RJFB01000020_ZH28_K1GA2SV | YKDKPNNTP--SQSGTKNDKNESAKNDKQES-----------SQNNSNTQVINPPN-STQ |
| NZ_MVVT01000021_HP13028_K1GA2SV | YKDKPNNTP--SQSGAKNDKNESAKNDKQES-----------SQNNSNTQVINPPN-SAQ |
| RJGI01000001_ZH62_K1GA2SV | YKDKPNNTP--SQSGAKNDKNESAKNDKQES-----------SQNNSNTQVINPPN-NTQ |
| 8722_ms1054_K1A2SV | YKDKPNNAP--SQSGTKNDKNESAKNDKQNS-----------TQNNSNTQVINPPN-SAQ |
| NZ_MIKU01000059_MCms1054_K1A2SV | YKDKPNNAP--SQSGTKNDKNESAKNDKQNS-----------TQNNSNTQVINPPN-SAQ |
| RJIY01000004_ZH139_QGA2SV | YKNQTNNTP--SQSGTKNDKNESAKNDKQES-----------SQNNSNTQVINPPN-SAQ |
| AF071095_F37_K1GA2SV | YKDKPNNTP--SQSGTKNDKNESAKNDKQES-----------AQNNSNTQVINPPN-SAQ |
| 8718_2003_84_K1GA2SV | YKDKPNNTP--SQSGAKNDKNESAKNDKQES-----------SQNNSNTQVINPPN-SAQ |
| NZ_MILX01000059_MU2003-84_K1GA2SV | YKDKPNNTP--SQSGAKNDKNESAKNDKQES-----------SQNNSNTQVINPPN-SAQ |
| NZ_CADE01000007_NQ315_K1GA1SV | YKDKPNNTP--SQSGTKNDKQES-------------------SQNNSNTQVINPPN-SAQ |
| NZ_MTWU01000002_SV380_1_K1GA2SV | YKDKPNNTP--SQSGAKNDKNESAKNDKQES-----------SQNNSNTQVINPPN-NTQ |
| NC_017379_Puno135_K1TA2SV | YKDKPNNTTQNS---AKNDKQNSAKNDKQKS-----------SQDNSNTQVINPPD-SGQ |
| RJFQ01000010_ZH43_K1GA1SV | YKDKPNSTT--SQSGAKNDKQEI------------------SQNNNSNTEVINPPN-NTQ |
| RJIZ01000020_ZH140_K1GA2V | YKDKPNNTN--SQSGAKSDKNESAKNDKQQS-----------S--NSNTEVINPPN-NTQ |
| NZ_AMOW01000004_R046Wa_K1GA2V | YKDKPNNTT--SQSGAKNDKNESAKNDKQQS-----------S--NSNTEVINPPN-SAQ |
| NZ_MBHU01000007_A037_QGA2SV | YKNQTNNTP--SQSGAKNDKNESAKNDKQNS-----------AQNNSNTQVINPPN-SAQ |
| NZ_MBGQ01000008_22317_K1GA1SV | YKDKPNNTP--SQSGTKNDKQES-------------------AQNNSNTQVINPPN-SAQ |
| NZ_MSYO01000016_PZ5009-3A2_K1GA1SV | YKDKPNNTP--SQSGTKNDKQES-------------------SQNNSNTQVINPPN-SAQ |
| 8697_ms1078_K1GA2V | YKDKPNNTT--PQSGTKNDKNESAKNDKQQS-----------S--NSNTEVINPPN-SAQ |
| NZ_MIKX01000057_MCms1078_K1GA2V | YKDKPNNTT--PQSGTKNDKNESAKNDKQQS-----------S--NSNTEVINPPN-SAQ |
| NZ_RPFU01000002_1088_K2TNSV | YKDKPKDKP--SNTTQNN-----ANNNQQNS-----------AQNNSSTQVINPPN-SAQ |
| NZ_MBJL01000248_2006_K1GA2V | YKDKPNNTT--SQSGAKNDKNESAKNDKQQS-----------S--NSNTEVINPPN-SAQ |
| NZ_MTWP01000004_SV340_2_QGA2SV | YKNQTNNTP--SQSGAKNDKNESAKNDKQES-----------SQNNSNTQVINPPN-SAQ |
| NZ_MBGX01000004_22151_QGA2SV | YKNQTNNTP--SQSGAKNDKNESAKNDKQES-----------SQNNSNTQVINPPN-NTQ |
| NZ_AKNT01000004_NQ4228_QGA2SV | YKNQTNNTP--SQSGAKNDKNESAKNDKQES-----------SQNNSNTQVINPPN-SAQ |
| 8734_22367_ve_QGA2SV | YKNQTNNTP--SQSGAKNDKNESAKNDKQES-----------SQNNSNTQVINPPN-NTQ |
| NZ_MBIM01000006_22367_QGA2SV | YKNQTNNTP--SQSGAKNDKNESAKNDKQES-----------SQNNSNTQVINPPN-NTQ |
| NZ_MUOZ01000053_CG22367_QGA2SV | YKNQTNNTP--SQSGAKNDKNESAKNDKQES-----------SQNNSNTQVINPPN-NTQ |
| NZ_ASYU01000056_PZ5056_QGA2SV | YKNQTNNTP--SQSGAKNDKNESAKNDKQES-----------SQNNSNTQVINPPN-SAQ |
| NZ_MBGT01000006_22312_K1GA1SV | YKDKPNNTP--SQSGAKNDKQES-------------------SQNNSNTQVINPPN-STQ |
| NZ_MUOG01000080_CA22312_K1GA1SV | YKDKPNNTP--SQSGAKNDKQES-------------------SQNNSNTQVINPPN-STQ |
| NZ_MBIK01000211_22370_QGA2SV | YKNQTNNTP--SQSGAKNDKNESAKNDKQES-----------SQNNSNTQVINPPN-NTQ |
| NZ_MUPA01000061_CG22370_QGA2SV | YKNQTNNTP--SQSGAKNDKNESAKNDKQES-----------SQNNSNTQVINPPN-NTQ |
| 8773_22388_QGA2SV | YKNQTNNTP--SQSGAKNDKNESAKNDKQES-----------SQNNSNTQVINPPN-SAQ |
| NZ_MUPQ01000420_CM22388_QGA2SV | YKNQTNNTP--SQSGAKNDKNESAKNDKQES-----------SQNNSNTQVINPPN-SAQ |
| NZ_MBHL01000007_3120_QGA2V | YKNQTNNTP--SQSGAKNDKNESAKNDKQQS-----------S--NSNTEVINPPN-SAQ |
| NZ_MVTK01000020_HP98123_K1GA2V | YKDKPNNTP--SQSGAKNDKNESAKNDKQQS-------------SNSNTEVINPPN-NTQ |
| NZ_MVUR01000022_HP15005_K1GA2V | YKDKPNNTT--PQSGAKNDKNESAKNDKQQS-------------SNSNTEVINPPN-NTQ |
| NZ_PHLY01000016_KH27_K1GA2V | YKDKPNNTT--PQSGTKNDKNESAKNDKQQS-------------SNSNTEVINPPN-NTQ |
| RJGV01000002_ZH79_K1GA1SV | YKDKPNSTT--SQSGAKNDKQEI------------------SQNNNSNTEVINPPN-SAQ |
| NZ_MVXG01000003_HP11013_K1GA1SV | YKDKPNNTT--SQSGAKNDKQQI------------------SQNNNSNTEVINPPN-NTQ |
| NZ_MVXJ01000003_HP11004_K1GA1SV | YKDKPNNTT--SQSGAKNDKQQI------------------SQNNNSNTEVINPPN-NTQ |
| RJED01000020_ZH05_K1GA2SV | YKDKPNNTP--SQSGAKNDKQESAKNDKQES-----------SQNNSNTQVINPPN-STQ |
| NZ_LIXH01000030_59_K1GA2SV | YKDKPNNTP--SQSGAKNDKQESAKNDKQES-----------SQNNSNTQVINPPN-STQ |
| NZ_AWUL01000013_CG-IMSS-2012_K1GA2V | YKDKPNSTP--SQSGAKNDKNESTKNDKQQS-----------S--NSNTEVINPPN-SAQ |
| 8723_ms44_K1GA2SV | YKDKPNNTP--SQSGAKNDKNESAKNDKQES-----------SQNNSNTQVINPPN-NTQ |
| NZ_MILP01000107_MGms44_K1GA2SV | YKDKPNNTP--SQSGAKNDKNESAKNDKQES-----------SQNNSNTQVINPPN-NTQ |
| NZ_MVWQ01000010_HP12053_K1GA1SV | YKDKPNNTP--SQSGTKNDKQES-------------------SQNNSNTQVINPPN-SAQ |
| NZ_QDJR01000018_B373_K1GA1SV | YKDKPNNTT--PQSGTKNDKQES-------------------SQNNSNTQVINPPN-SAQ |
| NZ_QEHG01000024_B274_K1GA2SV | YKDKPNNTP--SQSGAKNDKNESAKNDKQES-----------NQNNSNTQVINPPN-STQ |
| 8731_2005_126_K1GA2SV | YKDKPNNTP--SQSGAKNDKNESAKNDKQES-----------SQNNSNTQVINPPN-SAQ |
| NZ_MILT01000053_MM2005-126_K1GA2SV | YKDKPNNTP--SQSGAKNDKNESAKNDKQES-----------SQNNSNTQVINPPN-SAQ |
| RJFY01000019_ZH52_K1GA2SV | YKDKPNNTP--SQSGAKNDKNESAKNDKQES-----------SQNNSNTQVINPPN-SAQ |
| NZ_MBGP01000006_3046_K2TNSV | YKDKPKDKP--SNTTQNN------ANNNQQNS----------AQNNSNTQVINPPN-SVQ |
| NZ_MBKH01000230_3045_K2TNSV | YKDKPKDKP--SNTTQNN------ANNNQQNS----------AQNNSNTQVINPPN-SAQ |
| 8711_22023_K2TNSV | YKDKPKDKP--SNTTQNN------ANNNQQNS----------AQNNSNTQVINPPN-NAQ |
| NZ_MBHP01000007_22331_K2TNSV | YKDKPKDKP--SNTTQNN------ANNNQQNS----------AQNNSNTQVINPPN-NAQ |
| NZ_MUOU01000052_CG22023_K2TNSV | YKDKPKDKP--SNTTQNN------ANNNQQNS----------AQNNSNTQVINPPN-NAQ |
| NZ_MBGN01000031_3056_K2TNSV | YKDKPKDKP--SNTTQNN------ANNNQQNS----------AQNNSNTQVINPPN-SAQ |
| NZ_MBJZ01000010_22347_K2TNSV | YKDKPKDKP--SNTTQNN------ANNNQQNS----------AQNNSNTQVINPPN-STQ |
| 4556_Nic16_A_K2TNSV | YKDKPKDKP--SNTTQNN------ANNNQQNS----------AQNNSNTQVINPPN-NTQ |
| NZ_MBID01000109_22393_K2TNSV | YKDKPKDKP--SNTTQNN------ANNNQQNS----------AQNNSNTQVINPPN-NTQ |
| NZ_MUOM01000192_CA22393_K2TNSV | YKDKPKDKP--SNTTQNN------ANNNQQNS----------AQNNSNTQVINPPN-NTQ |
| NZ_CBPV010000002_SA156C_K2TNSV | YKDKPKDKP--SNTTQNN------ANNNQQNS----------AQNNSNTQVINPPN-SGQ |
| 4528_Nic01_A_K2TNSV | YKDKPKDKP--SNTTQNN------ANNNQQNS----------AQNNSNTQVINPPN-SAQ |
| NZ_QEHH01000020_B126_K2TNSV | YKDKPKDKP--SNTTQNN------ANNNQQNS----------AQNNSNTQVINPPN-NTQ |
| 8681_2012_26_K2TA1SV | YKDKHKDKP--SNTTQNN------AKNDKQES----------TQNNSNTQVINPPN-SAQ |
| NZ_MILW01000012_MM2012-26_K2TA1SV | YKDKHKDKP--SNTTQNN------AKNDKQES----------TQNNSNTQVINPPN-SAQ |
| NZ_QDJT01000021_B335_K2TNSV | YKDKPKDKP--SNTTQNN------ANNNQQNS----------AQNNSNTQVINPPN-SAQ |
| 8703_ms15_K2TNSV | YKDKPKDKP--SNTTQNN------ANNNQQNS----------AQNNSNTQVINPPN-SAQ |
| NZ_MILJ01000079_MGms15_K2TNSV | YKDKPKDKP--SNTTQNN------ANNNQQNS----------AQNNSNTQVINPPN-SAQ |
| 6292_Nic27_A_K2TNSV | YKDKPKDKP--SNTTQNN------ANNNQQNS----------AQNNSNTQVINPPN-SAQ |
| NZ_MBJB01000046_2036_K2TNSV | YKDKPKDKP--SNTTQNN------ANNNQQNS----------AQNNSNTQVINPPN-SAQ |
| NZ_MBIY01000198_3026_K2TNSV | YKDKPKDKP--SNTTQNN------ANNNQQNS----------AQNNSNTQVINPPN-STQ |
| 8688_ms1063_K2TNSV | YKDKPKDKP--SNTTQNN------ANNNQQNS----------AQNNSNTQVINPPN-SAQ |
| NZ_MIKW01000056_MCms1063_K2TNSV | YKDKPKDKP--SNTTQNN------ANNNQQNS----------AQNNSNTQVINPPN-SAQ |
| NZ_MBJE01000006_2061_K2TNSV | YKDKPKDKP--SNTTQNN------ANNNQQNS----------AQNNSNTQVINPPN-SAQ |
| NZ_MBJK01000002_1102_K2TNSV | YKDKPKDKP--SNTTQNN------ANNNQQNS----------AQNNSNTQVINPPN-SAQ |
| NZ_RPFS01000014_1002_K2TNSV | YKDKPKDKP--SNTTQNN------ANNNQQNS----------AQNNSNTQVINPPN-SAQ |
| NZ_MBIA01000025_24004_K2TA1SV | YKDKHKDKP--SNTTQNN------AKNDKQES----------TQNNSNTQVINPPN-STQ |
| NZ_MUON01000059_CA24004_K2TA1SV | YKDKHKDKP--SNTTQNN------AKNDKQES----------TQNNSNTQVINPPN-STQ |
| NZ_MBHX01000046_26084_K2TNSV | YKDKPKDKP--SNTTQNN------ANNNQQNS----------AQNNSNTQVINPPN-SAQ |
| NZ_MUOR01000021_CC26084_K2TNSV | YKDKPKDKP--SNTTQNN------ANNNQQNS----------AQNNSNTQVINPPN-SAQ |
| NZ_MBJC01000012_2040_K2TNSV | YKDKPKDKP--SNTTQNN------ANNNQQNS----------AQNNSNTQVINPPN-SAQ |
| NZ_MBJJ01000007_2027_K2TNSV | YKDKPKDKP--SNTTQNN------ANNNQQNS----------AQNNSNTQVINPPN-SAQ |
| 8720_22046_ve_K2TNSV | YKDKPKDKP--SNTTQNN------ANNNQQNS----------AQNNSNTQVINPPN-SAQ |
| NZ_MBKB01000048_22046_K2TNSV | YKDKPKDKP--SNTTQNN------ANNNQQNS----------AQNNSNTQVINPPN-SAQ |
| NZ_MUPH01000105_CM22046_K2TNSV | YKDKPKDKP--SNTTQNN------ANNNQQNS----------AQNNSNTQVINPPN-SAQ |
| NZ_MBKG01000018_2029_K2TNSV | YKDKPKDKP--SNTTQNN------ANNNQQNS----------AQNNSNTQVINPPN-NTQ |
| 8745_2011_145_K2TNSV | YKDKPKDKP--SNTTQNN------ANNNQQNS----------AQNNSNTQVINPPN-SAQ |
| NZ_MIKT01000123_MC2011-145_K2TNSV | YKDKPKDKP--SNTTQNN------ANNNQQNS----------AQNNSNTQVINPPN-SAQ |
| NZ_AKNU01000004_NQ4099_K2TA1SV | YKDKHKDKP--SNTTQNN------AKNDKQES----------TQNNSNTQVINPPN-SAQ |
| 8693_2005_98_K2TNSV | YKDKPKDKP--SNTTQNN------ANNNQQNS----------AQNNSNTQVINPPN-SAQ |
| NZ_MILD01000081_MG2005-98_K2TNSV | YKDKPKDKP--SNTTQNN------ANNNQQNS----------AQNNSNTQVINPPN-SAQ |
| NZ_AKNR01000007_NQ4216_K2TNSV | YKDKPKDKP--SNTTQNN------ANNNQQNS----------AQNNSNTQVINPPN-SAQ |
| NZ_QEHC01000017_B345_K2TNSV | YKDKPKDKP--SNTTQNN------ANNNQQNS----------AQNNSNTQVINPPN-SAQ |
| RJEY01000032_ZH25_K2TNSV | YKDKPKDKP--SNTTQNN------ANNNQQNS----------AQNNSNTQVINPPN-SAQ |
| NZ_MBIZ01000036_3029_K2TNSV | YKDKPKDKP--SNTTQNN------ANNNQQNS----------AQNNSNTQVINPPN-SAQ |
| NZ_MBIX01000003_3004_K2TNSV | YKDKPKDKP--SNTTQNN------ANNNQQNS----------AQNNSNTQVINPPN-SAQ |
| NZ_MBJV01000039_26083_K2TNSV | YKDKPKDKP--SNTTQNN------ANNNQQNS----------AQNNSNTQVINPPN-SAQ |
| NZ_UGHQ01000001_NCTC13338_K1GA1SV | YKDKPNNTT--SQSGAKNGKQEI------------------SQNNNSNTEVINPPN-NTQ |
| RJHB01000013_ZH86_K1GA1SV | YKDKPNNTT--SQSGAKNDKQQI------------------SQNNNSNTEVINPPN-NTQ |
| NZ_AKPE01000004_HpH-19_K2TA1SV | YKDKPKDKP--SNTTQNN------AKNDKQES----------TQNNSNTQVINPPN-STQ |
| NZ_MBJI01000007_2025_K2TNSV | YKDKPKDKP--SNTTQNN------ANNNQQNS----------AQNNSNTQVINPPN-SAQ |
| 4562_Nic19_A_K2TA1SV | YKDKPKDKP--SNTTQNN------AKNDKQES----------SQNNSNTQVINPPN-SAQ |
| 8701_2006_103_K2TNSV | YKDKPKDKP--SNTTQNN------ANNNQQNS----------TQNNSNTQVINPPN-SAQ |
| NZ_MILS01000011_MM2006-103_K2TNSV | YKDKPKDKP--SNTTQNN------ANNNQQNS----------TQNNSNTQVINPPN-SAQ |
| 6289_Nic24_A_K2TNSV | YKDKPKDKP--SNTTQNN------ANNNQQNS----------AQNNSNTQVINPPN-NTQ |
| 6293_Nic28_A_K2TNSV | YKDKPKDKP--SNTTQNN------ANNNQQNS----------AQNNSNTQVINPPN-NTQ |
| 4552_Nic14_A_K2TNSV | YKDKPKDKP--SNTTQNN------ANNNQQNS----------AQNNSNTQVINPPN-SAQ |
| NZ_RPFR01000012_1071_K2TNSV | YKDKPKDKP--SNTTQNN------ANNNQQNS----------AQNNSNTQVINPPN-STQ |
| NZ_MTWL01000002_PZ5016_3A3_K2TNSV | YKDKPKDKP--SNTTQNN------ANNNQQNS----------AQNNSNTQVINPPN-SVQ |
| 4548_Nic12_A_K2TNSV | YKDKPKDKP--SNTTQNN------ANNNQQNS----------AQNNSNTQVINPPN-SAQ |
| NZ_AKPH01000005_HpH-34_K2TNSV | YKDKPKDKP--SNTTQNN------ANNNQQNS----------AQNNSNTQVINPPN-STQ |
| 8713_ms931_K1GA1SV | YKDKPNNTP--SQSGTKNDKQEI------------------SQNNNSNTEVINPPN-NTQ |
| NZ_MIKZ01000063_MCms931_K1GA1SV | YKDKPNNTP--SQSGTKNDKQEI------------------SQNNNSNTEVINPPN-NTQ |
| 6288_Nic23_A_K1GA2SV | YKDKPNNTP--SQSGTKNDKQESTKNDKQEI----------SQNNNSNTEVINPPN-NTQ |
| NZ_MBIJ01000100_22371_K1GA1SV | YKDKPNNTT--SQSGTKNDKQQI------------------SQNNNSNTEVINPPN-NTQ |
| NZ_MUPB01000015_CG22371_K1GA1SV | YKDKPNNTT--SQSGTKNDKQQI------------------SQNNNSNTEVINPPN-NTQ |
| NZ_MBJT01000024_1061_K1GA1SV | YKDKPNNTT--SQSGTKNDKQQI------------------SQNNNSNTEVINPPN-NTQ |
| 8721_2006_479_K1GA1SV | YKDKPNNTT--SQSGTKNDKQEI------------------SQNSNSNTEVINPPN-NTQ |
| NZ_MILG01000056_MG2006-479_K1GA1SV | YKDKPNNTT--SQSGTKNDKQEI------------------SQNSNSNTEVINPPN-NTQ |
| 4538_Nic07_A_K1GA1SV | YKDKPNNTT--PQSGTKNDKQEI------------------SQNNNSNTEVINPPN-NTQ |
| 4566_Nic21_C_K1GA1SV | YKDKPNNTT--SQSGTKNDKQEI------------------SQNNNSNTEVINPPN-NTQ |
| NZ_CBKY010000006_HPARG63_K1GA2V | YKDKPNNTT--SQSGAKNDKNESAKNDKQQS-----------S--NSNTEVINPPN-NTQ |
| NZ_AKPJ01000001_HpP-2_K1GA2SV | YKDKPNNTS--SQSGTKNGKNESAKNDKQES-----------SQNNSNTQVINPPN-SAQ |
| 8732_2003_103_K1GA1SV | YKDKPNNTP--SQSGTKNDKQEI------------------SQNNNSNTQVINPPN-SAQ |
| NZ_MIKR01000134_MM2003-103_K1GA1SV | YKDKPNNTP--SQSGTKNDKQEI------------------SQNNNSNTQVINPPN-SAQ |
| NZ_MBIH01000019_22378_K1GA2V | YKDKPNNTP--SQSGTKNDKNESAKNDKQQS-----------S--NSNTEVINPPN-SAQ |
| NZ_MUPC01000161_CG22378_K1GA2V | YKDKPNNTP--SQSGTKNDKNESAKNDKQQS-----------S--NSNTEVINPPN-SAQ |
| NZ_AMOR01000004_R030b_K1GA2V | YKDKPNNTT--SQSGAKNDKNESAKNDKQQS-----------S--NSNTEVINPPN-NTQ |
| NZ_AKPD01000011_HpH-18_K1GA2V | YKDKPNNTP--SQSGAKNDKNESAKNDKQQS-----------S--NSNTEVINPPN-STQ |
| 8760_22315_ve_K1GA2V | YKDKPNNTT--SQSGAKNDKNESAKNDKQQS-----------S--NSNTEVINPPN-SAQ |
| NZ_MBHA01000006_22087_K1GA2V | YKDKPNNTT--SQSGAKNDKNESAKNDKQQS-----------S--NSNTEVINPPN-SAQ |
| NZ_MUPI01000073_CM22315_K1GA2V | YKDKPNNTT--SQSGAKNDKNESAKNDKQQS-----------S--NSNTEVINPPN-SAQ |
| NZ_MBIB01000047_22402_K1GA2V | YKDKPNNTP--SQSGAKNDKNESAKNDKQQS-----------S--NSNTEVINPPN-SAQ |
| NZ_MUOQ01000009_CC22402_K1GA2V | YKDKPNNTP--SQSGAKNDKNESAKNDKQQS-----------S--NSNTEVINPPN-SAQ |
| 8749_2004_2_K1GA2SV | YKDKPNNTP--SQSGAKNDKNESAKNDKQES-----------SQNNSNTQVINPHN-NTQ |
| NZ_MILY01000017_MU2004-2_K1GA2SV | YKDKPNNTP--SQSGAKNDKNESAKNDKQES-----------SQNNSNTQVINPHN-NTQ |
| 8737_2006_480_K1GA2V | YKDKPNNTT--SQSGAKNDKNESAKNDKQQS-----------S--NSNTEVINPPN-NTQ |
| NZ_MILU01000102_MM2006-480_K1GA2V | YKDKPNNTT--SQSGAKNDKNESAKNDKQQS-----------S--NSNTEVINPPN-NTQ |
| NZ_MVSW01000003_HP99648_K1GA2V | YKDKPNNTP--SQSGAKNDKNESAKNDKQDS----------------NTQVINPPN-SGQ |
| 8705_ms23_K1GA1SV | YKDKPNNTT--SQSGAKNDKQES-------------------SQNNSNTQVINPPN-SVQ |
| NZ_MILO01000042_MGms23_K1GA1SV | YKDKPNNTT--SQSGAKNDKQES-------------------SQNNSNTQVINPPN-SVQ |
| NC_017742_PeCan18_K1GA2SV | YKDKPNNTP--SQSGAKNDKNESAKNDKQES-----------SQNNSNTQVINPPN-STQ |
| NZ_QEGM01000018_CRM21_K1GA1SV | YKDKPNNTT--SQSGAKNDKQES-------------------SQNNSNTQVINPPN-SAQ |
| 6284_Nic30_A_K1GA2SV | YKDKPNNTT--PQSGAKNDKNESAKNDKQES-----------SQNNSNTQVINPPN-SAQ |
| NZ_MVXQ01000015_HP08031_K1GA2SV | YKDKPNNTP--SQNGAKNDKNESAKNDKQES-----------SQNNSNTQVINPPN-STQ |
| NZ_CBMY010000015_SA216A_K1GA2SV | YKDKPNNTP--SQSGAKNDKNESAKNDKQES-----------SQNNSNTQVINPPN-SAQ |
| NZ_CBNC010000010_SA216C_K1GA2SV | YKDKPNNTP--SQSGAKNDKNESAKNDKQES-----------SQNNSNTQVINPPN-SAQ |
| NZ_CBNO010000004_SA215C_K1GA2SV | YKDKPNNTP--SQSGAKNDKNESAKNDKQES-----------SQNNSNTQVINPPN-SAQ |
| NZ_CBNS010000014_SA214C_K1GA2SV | YKDKPNNTP--SQSGAKNDKNESAKNDKQES-----------SQNNSNTQVINPPN-SAQ |
| NZ_CBNM010000002_SA161C_K1GA2SV | YKDKPNNTP--SQSGAKNDKNESAKNDKQES-----------SQNNSNTQVINPPN-SAQ |
| NZ_CBPE010000005_SA161A_K1GA2SV | YKDKPNNTP--SQSGAKNDKNESAKNDKQES-----------SQNNSNTQVINPPN-SAQ |
| NZ_MBJW01000023_24012_K1GA2SV | YKDKPNNTP--SQSGAKNDKNESAKNDKQES-----------SQNNSNTQVINPPN-NTQ |
| NZ_MTWM01000012_PZ5019_3A3_QGA2SV | YKNQTNNTP--SQSGAKNDKNESAKNDKQES-----------SQNNSNTQVINPPN-SAQ |
| AB190965_F80_K1GA2SV | YKDKPNNTP--SQSGTKNDKNESAKNDKQNS-----------AQNNSNTQVINPPN-SAQ |
| AB190966_F92_K1GA2SV | YKDKPNNTP--SQSGTKNDKNESAKNDKQNS-----------AQNNSNTQVINPPN-SAQ |
| NZ_AP014523_NY40_QGA2SV | YKNQTNNTP--SQSGAKNDKNESAKNDKQES-----------SQNNSNTQVINPPN-NTQ |
| NZ_JSXT01000040_1198/04_QGA2SV | YKNQTNNTP--SQSGAKNDKNESAKNDKQES-----------SQNNSNTQVINPPN-SAQ |
| NZ_QEGR01000015_B630_K1GA2V | YKDKPNNTT--SQSGAKNDKNESAKNDKQQS-----------S--NSNTEVINPPN-STQ |
| NZ_MBJM01000006_2007_K1GA2V | YKDKPNNTP--SQSGAKNDKNESAKNDKQQS-----------S--NSNTEVINPPN-SAQ |
| NZ_UGJP01000002_NCTC13094_K1GA2SV | YKDKPNNTT--PQSGTKNDKNESAKNDKQES-----------SQNNSNTQVINPPN-STQ |
| NZ_MBIG01000006_22385_K1GA2V | YKDKPNNTP--SQSGAKNDKNESAKNDKQQS-----------S--NSNTEVINPPN-SAQ |
| NZ_MUPD01000137_CG22385_K1GA2V | YKDKPNNTP--SQSGAKNDKNESAKNDKQQS-----------S--NSNTEVINPPN-SAQ |
| NZ_MBHN01000027_22336_QGA2SV | YKNQTNNTP--SQSGAKNDKNESAKNDKQES-----------SQNNSNTQVINPPN-SAQ |
| NZ_MBHO01000001_22335_QGA2SV | YKNQTNNTP--SQSGAKNDKNESAKNDKQES-----------SQNNSNTQVINPPN-SAQ |
| NZ_MUOI01000088_CA22335_QGA2SV | YKNQTNNTP--SQSGAKNDKNESAKNDKQES-----------SQNNSNTQVINPPN-SAQ |
| 8726_22366_ve_QGA2SV | YKNQTNNTP--SQSGAKNDKNESAKNDKQES-----------SQNNSNTQVINPPN-SAQ |
| NZ_MBGY01000008_22095_QGA2SV | YKNQTNNTP--SQSGAKNDKNESAKNDKQES-----------SQNNSNTQVINPPN-SAQ |
| NZ_MUOY01000104_CG22366_QGA2SV | YKNQTNNTP--SQSGAKNDKNESAKNDKQES-----------SQNNSNTQVINPPN-SAQ |
| NZ_MBIS01000002_22346_QGA2SV | YKNQTNNTP--SQSGAKNDKNESAKNDKQES-----------SQNNSNTQVINPPN-NTQ |
| NZ_MUOK01000107_CA22339_QGA2SV | YKNQTNNTP--SQSGAKNDKNESAKNDKQES-----------SQNNSNTQVINPPN-NTQ |
| 8709_22341_ve_QGA2SV | YKNQTNNTP--SQSGAKNDKNESAKNDKQES-----------SQNNSNTQVINPPN-SAQ |
| NZ_MUPK01000086_CM22341_QGA2SV | YKNQTNNTP--SQSGAKNDKNESAKNDKQES-----------SQNNSNTQVINPPN-SAQ |
| NZ_MBIL01000022_22368_QGA2SV | YKNQTNNTP--SQSGAKNDKNESAKNDKQES-----------SQNNSNTQVINPPN-NTQ |
| NZ_MUPP01000031_CM22368_QGA2SV | YKNQTNNTP--SQSGAKNDKNESAKNDKQES-----------SQNNSNTQVINPPN-NTQ |
| NZ_MTWN01000019_PZ5033_3A2_QGA2SV | YKNQTNNTP--SQSGAKNDKNESAKNDKQES-----------SQNNSNTQVINPPN-SAQ |
| NC_014560_SJM180_K1GA2SV | YKDKPNNTP--SQSGTKNDKNESAKNDKQES-----------SQNNSNTQVINPPN-NTQ |
| NZ_MBHF01000009_22019_K1GA1SV | YKDKPNNTT--PQSG--------TKNDKQES-----------SQNNSNTQVINPPN-SAQ |
| NZ_MUPL01000007_CM22346_K1GA1SV | YKDKPNNTT--PQSG--------TKNDKQES-----------SQNNSNTQVINPPN-SAQ |
| 4534_Nic05_A_QGA2SV | YKNQTNNTP--SQSGAKNDKNESAKNDKQES-----------SQNNSNTQVINPPN-SAQ |
| 4536_Nic06_A_QGA2SV | YKNQTNNTP--SQSGAKNDKNESAKNDKQES-----------SQNNSNTQVINPPN-SAQ |
| NZ_MBIE01000005_22390_K1GA2V | YKDKPNNTP--SQSGAKNDKNESAKNDKQQS-----------S--NSNTEVINPPN-SAQ |
| NZ_MUOB01000003_CM22390_K1GA2V | YKDKPNNTP--SQSGAKNDKNESAKNDKQQS-----------S--NSNTEVINPPN-SAQ |
| NZ_ASYV01000171_PZ5080_QA2SV | YKSQTNNTPPPSQSGAKNDKNESAKNDKQES-----------SQNNSNTQVINPPN-SAQ |
| NZ_MBHY01000007_26024_QGA2SV | YKNQTNNTP--SQSGAKNDKNESAKNDKQES-----------SQNNSNTQVINPPN-SAQ |
| NZ_MUOO01000173_CA26024_QGA2SV | YKNQTNNTP--SQSGAKNDKNESAKNDKQES-----------SQNNSNTQVINPPN-SAQ |
| NZ_MBHM01000006_3118_K1GA2V | YKDKPNNTT--SQSGAKNDKNESAKNDKQQS-----------S--NSNTEVINPPN-SAQ |
| NZ_MBJA01000027_3033_K1GA2V | YKDKPNNTT--SQSGAKNDKNESAKNDKQQS-----------S--NSNTEVINPPN-SAQ |
| 8756_22019_ve_K1GA2V | YKDKPNNTP--SQSGAKNDKNESAKNDKQQS-----------S--NSNTEVINPPN-SAQ |
| NZ_MBIU01000007_22341_K1GA2V | YKDKPNNTP--SQSGAKNDKNESAKNDKQQS-----------S--NSNTEVINPPN-SAQ |
| NZ_MUOC01000010_CA22019_K1GA2V | YKDKPNNTP--SQSGAKNDKNESAKNDKQQS-----------S--NSNTEVINPPN-SAQ |
| NZ_MBJN01000004_2010_K1GA2V | YKDKPNNTP--SQSGTKNDKNESAKNDKQQS-----------S--NSNTEVINPPN-SAQ |
| NZ_QBQR01000066_GC52-HL_QGA2SV | YKNQTNNTP--SQSGAKNDKNESAKNDKQES-----------SQNNSNTQVINPPN-SAQ |
| NZ_QDJP01000022_B464A_K1GA2SV | YKDKPNNTP--SQSGTKNDKNESAKNDKQES-----------SQNNSNTQVINPPN-SAQ |
| NZ_MBJF01000007_2065_K1GA2SV | YKDKPNNTP--SQSGAKNDKNESAKNDKQES-----------SQNNSNTQVINPPN-SAQ |
| 8716_ms13_K1GA1SV | YKDKPNNTT--PQSGTKNDKQES-------------------SQNNSNTQVINPPN-SAQ |
| NZ_MILI01000029_MGms13_K1GA1SV | YKDKPNNTT--PQSGTKNDKQES-------------------SQNNSNTQVINPPN-SAQ |
| NZ_MBKD01000014_1086_QGA2SV | YKNQTNNTP--SQSGAKNDKNESAKNDKQES-----------SQNNSNTQVINPPN-NTQ |
| NZ_MUPM01000092_CM22347_K1GA2SV | YKDKPNNTT--SQSGAKNDKNESAKNDKQES-----------SQNNSNTQVINPPN-SAQ |
| 8695_2005_72_QGA2SV | YKNQTNNTP--SQSGAKNDKNESAKNDKQES-----------SQNNSNTQVINPPN-SAQ |
| NZ_MILR01000028_MM2005-72_QGA2SV | YKNQTNNTP--SQSGAKNDKNESAKNDKQES-----------SQNNSNTQVINPPN-SAQ |
| NZ_MBGV01000030_22308_K1GA2V | YKDKPNNTP--SQSGAKNDKNESAKNDKQQS-----------S--NSNTEVINPPN-SAQ |
| NC_017063_ELS37_QGA2SV | YKNQTNNTP--SQSGAKNDKNESAKNDKQES-----------SQNNSNTQVINPPN-STQ |
| 8684_ms1055_QGA2SV | YKNQTNNTP--SQSGAKNDKNESAKNDKQES-----------SQNNSNTQVINPPN-STQ |
| NZ_MIKV01000030_MCms1055_QGA2SV | YKNQTNNTP--SQSGAKNDKNESAKNDKQES-----------SQNNSNTQVINPPN-STQ |
| NZ_RPFT01000013_1057_QGA2SV | YKNQTNNTP--SQSGAKNDKNESAKNDKQES-----------SQNNSNTQVINPPN-SAQ |
| NZ_MBIO01000012_22362_K1GA2SV | YKDKPNNTP--SQSGAKNDKNESAKNDKQES-----------SQNNSNTQVINPPN-SAQ |
| NZ_MUOL01000069_CA22362_K1GA2SV | YKDKPNNTP--SQSGAKNDKNESAKNDKQES-----------SQNNSNTQVINPPN-SAQ |
| NZ_MBGS01000006_22315_QGA1SV | YKNQTNNTP--SQSGAKNDKQES-------------------SQNNSNTQVINPPN-SAQ |
| NZ_RPFQ01000017_22345_QGA1SV | YKNQTNNTP--SQSGAKNDKQES-------------------SQNNSNTQVINPPN-SAQ |
| NZ_MBHD01000006_22021_QGA2SV | YKNQTNNTP--SQSGAKNDKQESAKNDKQES-----------SQNNSNTQVINPPN-SAQ |
| NZ_MUPG01000144_CM22021_QGA2SV | YKNQTNNTP--SQSGAKNDKQESAKNDKQES-----------SQNNSNTQVINPPN-SAQ |
| NZ_MBGU01000058_22311_QGA2SV | YKNQTNNTP--SQSGAKNDKNESAKNDKQES-----------SQNNSNTQVINPPN-SAQ |
| NZ_MUOF01000136_CA22311_QGA2SV | YKNQTNNTP--SQSGAKNDKNESAKNDKQES-----------SQNNSNTQVINPPN-SAQ |
| NZ_MBIT01000036_22343_QGA2SV | YKNQTNNTP--SQSGAKNDKNESAKNDKQES-----------SQNNSNTQVINPPN-SAQ |
| NZ_MVWF01000023_HP13005_K1GA2V | YKDKPNNTT--SQSGAKNDKNESAKNDKQQS-----------S--NSNTEVINPPN-NTQ |
| NZ_MVXO01000004_HP08061_K1GA2V | YKDKPNNTT--SQSGAKNDKNESAKNDKQQS-----------S--NSNTEVINPPN-NTQ |
| NZ_MVXR01000011_HP07036_K1GA2V | YKDKPNNTT--SQSGAKNDKNESAKNDKQQS-----------S--NSNTEVINPPN-NTQ |
| NZ_QDJO01000016_B491_K1GA1SV | YKDKPNSTP--SQSGTKNDKQQI------------------SQNNNSNTEVINPPN-SAQ |
| 6291_Nic26_A_K1GA1SV | YKDKPNNTT--PQSGTKNDKQEI------------------SQNNNSNTEVINPPN-STQ |
| 8694_ms203_K1GA1SV | YKDKPNNTP--SQSGTKNDKQEI------------------SQNNNSNTQVINPPN-SAQ |
| NZ_MILN01000079_MGms203_K1GA1SV | YKDKPNNTP--SQSGTKNDKQEI------------------SQNNNSNTQVINPPN-SAQ |
| NZ_MVTF01000002_HP99244_K1GA2SV | YKDKPNNTP--SQSGAKNDKNESAKNDKQES-----------SQNNSNTQVINPPN-SAQ |
| NZ_MTWT01000005_SV449_1_K1GA2SV | YKDKPNNTP--SQSGAKNDKNESAKNDKQES-----------SQNNSNTQVINPPN-NTQ |
| NZ_MBGL01000078_3096_QGA2SV | YKNQTNNTP--SQSGAKNDKNESAKNDKQES-----------SQNNSNTQVINPPN-SAQ |
| NZ_MBJP01000076_1081_K1GA1SV | YKDKPNNTP--SQSGAKNDKQES-------------------SQNNSNTQVINPPN-NTQ |
| NZ_MBJD01000006_2047_K1GA2SV | YKDKPNNTP--SQSGAKNDKNESAKNDKQES-----------SQNNSNTQVINPPN-NTQ |
| RJIJ01000036_ZH124_QGA2SV | YKNQTNNTP--SQSGAKNDKNESAKNDKQEN-----------SQNNSNTQVINPPN-SAQ |
| NZ_MVXB01000021_HP11043_K1GA2V | YKDKPNNTN--SQSGAKNDKNESAKNDKQDS----------------NTQVINPPN-SAQ |
| RJHY01000020_ZH112_K1GA2SV | YKDKPNNTT--PQSGAKNDKNESAKNDKQES-----------SQNNSNTQVINPPN-SAQ |
| RJEH01000002_ZH09_QGA2SV | YKNQTNNTP--SQSGAKNDKNESAKNDKQES-----------SQNNSNTQVINPPN-SAQ |
| RJFI01000003_ZH35_K1GA2SV | YKDKPNNTP--SQSGAKNDKNESTKNDKQES-----------SQNNSNTQVINPPN-SSQ |
| NZ_CBRI010000002_HP87hu_QGA2SV | YKNQTNNTP--SQSGAKNDKNESTKNDKQES-----------SQNNSNTQVINPPN-SAQ |
| RJHX01000024_ZH1111_K1GA2V | YKDKPNNTP--SQSGAKNDKNESAKNDKKD----------------SNTQVINSPN-SAQ |
| NC_011498_P12_K1GA2SV | YKDKPNNTP--SQSGAKNDKNESAKNDKQES-----------SQNNSNTQVINPPN-SAQ |
| RJFO01000013_ZH41_K1GA2SV | YKDKPNNTP--SQSGTKNDKNESAKNDKQES-----------SQNNSNTQVINPPN-SAQ |
| GQ331980_DL1_K1GA2SV | YKDKPNNTP--SQSGAKNDKNESAKNDKQES-----------SQNNSNTQVINPPN-SAQ |
| NZ_MVUH01000002_HP15026_K1GA2SV | YKDKPNNTP--SQSGAKNDKNESAKNDKQES-----------SQNNSNTQVINPPN-SAQ |
| 3628_SSR22_K1GA2SV | YKDKPNNTL--SQSGAKNDKNESAKNDKQES-----------SQNNSNTEVINPPN-SVQ |
| NZ_PHLX01000007_KH28_QGA2SV | YKNQTNNTP--SQSGTKNDKNESAKNDKQES-----------SQNNSNTQVINPPN-STQ |
| NZ_MVTV01000026_HP15050_K1GA2SV | YKDKPNNTP--SQSGAKNDKNESAKNDKQES-----------SQNNSNTQVINPPN-STQ |
| NZ_QBQZ01000014_GC65-HL_K1GA2SV | YKDKPNNTP--SQSGAKNDKNESAKNDKQES-----------SQNNSNTQVINPPN-SAQ |
| NZ_QBPN01000045_38:2_K1GA1SV | YKDKPNNTN--SQSG--------AKNDKQES-----------SQNNSNTQVINPPN-SAQ |
| NZ_MVUF01000024_HP15028_K2TNSV | YKDKPKDKP-------SNTTQNNANNNQQNS-----------AQNNSNTQVINPPN-SAQ |
| NZ_QBPW01000025_52_K2TNSV | YKDKPKDKP-------SNTTQNNANNNQQNS-----------AQNNSNTQVINPPN-SAQ |
| Z26883_pWS10,pWS16_K2TA1SV | YKDKPNDKP-------SNTTQNNAKNDKQES-----------SQNNSNTQVINPPN-SAQ |
| NC_000915_26695_K2TNSV | YKDKPKDKP-------SNTTQNNANNNQQNS-----------AQNNSNTQVINPPN-SAQ |
| NJFC02000001_FDAARGOS_299_K2TNSV | YKDKPKDKP-------SNTTQNNANNNQQNS-----------AQNNSNTQVINPPN-SAQ |
| NZ_AZBP01000022_SS1_K2TNSV | YKDKPKDKP-------SNTTQNNANNNQQNS-----------AQNNSNTQVINPPN-SAQ |
| NZ_CP026325_dRdM1_K2TNSV | YKDKPKDKP-------SNTTQNNANNNQQNS-----------AQNNSNTQVINPPN-SAQ |
| NZ_CP026515_dRdM2addM2_K2TNSV | YKDKPKDKP-------SNTTQNNANNNQQNS-----------AQNNSNTQVINPPN-SAQ |
| NZ_FLKJ01000039_BAC555-8_K2TNSV | YKDKPKDKP-------SNTTQNNANNNQQNS-----------AQNNSNTQVINPPN-SAQ |
| NZ_FLKK01000008_BAC555-2_K2TNSV | YKDKPKDKP-------SNTTQNNANNNQQNS-----------AQNNSNTQVINPPN-SAQ |
| NZ_MVWD01000016_HP13009_K2TNSV | YKDKPKDKP-------SNTTQNNTNNNQQNS-----------AQNNSNTQVINPPN-SAQ |
| RJIC01000002_ZH117_K2TNSV | YKDKPKDKP-------SNTTQNNANNNQQNS-----------AQNNNNTSVINPPN-STQ |
| NZ_QBQX01000052_SSR1_K2TA1SV | YKDKPKDKP-------SNTTQNNAKNDKQES-----------SQNNSNTQVINPPN-SAQ |
| RJEM01000004_ZH13_K2TNSV | YKDKPKDKP-------NNTTQNNANNNQQNS-----------AQNNSNTQVINPPN-SAQ |
| RJIE01000002_ZH119_K2TA1SV | YKDKPKDKP-------SNTTQNNAKNDKQES-----------AQNNSNTQVINPPN-SAQ |
| NZ_CP034314_HP42K_K2TNSV | YKDKPKDKP-------SNTTQNNANNNQQNS-----------AQNNNNTSVINPPN-SAQ |
| NZ_MVSM01000011_HPJ040_K2TNSV | YKDKPKDKP-------SNTTQNNANNNQQNS-----------AQNNSNTQVINPPN-SAQ |
| NZ_QBQS01000049_GC30-HL_K2TA1SV | YKDKPKDKP-------SNTTQNNAKNDKQES-----------SQNNNNTQVINPPN-SAQ |
| NZ_QBPZ01000011_638_K2TNSV | YKDKPKDKP-------SNTTQNNANNNQQNS-----------AQNNSNTQVINPPN-SAQ |
| NZ_QBPX01000034_50_K3TNSV | YKDKPKDKPKDKP---NNTTQNNANNNQQNS-----------AQNNSNTQVINPPN-SAQ |
| NZ_QBPS01000058_18:2_K2TNSV | YKDKPKDKP-------SNTTQNNANNNQQNS-----------AQNNNNTSVINPPN-SAQ |
| NZ_CCMU01000017_H3014_K2TNSV | YKDKPKDKP-------SNTTQNNANNNQQNS-----------AQNNSNTQVINPPN-SAQ |
| NZ_MVYG01000022_HP02140_K2TA1SV | YKDKPKDKP-------SNTTQNNAKNDKQES-----------SQNNSNTQVINPPN-SAQ |
| NZ_MVSN01000029_HPJ025_K2TNSV | YKDKPKDKP-------SNTTQNNANNNQQNS-----------AQNNNNTSVINPPN-SAQ |
| NZ_MVSO01000002_HPJ024_K2TNSV | YKDKPKDKP-------SNTTQNNANNNQQNS-----------AQNNNNTSVINPPN-SAQ |
| 3627_SSR20_K3TNSV | YKDKPKDKPKDKP---SNTTQNNANNNQQNS-----------AQNNSNTQVINPPN-SAQ |
| 3631_SSR40_K2TNSV | YKDKPKDKP-------SNTTQNNANNNQQNS-----------AQNNSNTQVINPPN-SAQ |
| NZ_AKPW01000005_HpP-62_K2TNSV | YKDKPKDKP-------SNTTQNNANNNQQNS-----------AQNNSNTQVINPPN-STQ |
| NZ_QBPT01000201_73_K2TNSV | YKDKPKDKP-------SNTTQNNANNNQQNS-----------AQNNSNTQVINPPN-SAQ |
| NZ_AMOS01000005_R32b_K2TNSV | YKDKPKDKP-------SNTTQNNANNNQQNS-----------TQNNNNTHVINPPN-SAQ |
| NZ_QBPP01000035_29:2_single_K2TNSV | YKDKPKDKP-------SNTTQNNANNNQQNS-----------AQNNSNTQVINPPN-SAQ |
| RJEP01000012_ZH16_K1GA2V | YKDKPNNTP--SQSGAKNDKNESAKNDKQDS----------------NTQVINPPN-SAQ |
| RJGM01000009_ZH67_K1TA1SV | YKDKPKGKP-------SNTTQNNAKNDKQES-----------AQNNSNTQVINPPN-SAQ |
| RJIB01000013_ZH116_QGA2SV | YKNQTNNTP--SQSGAKNNKNESAKNDKQES-----------SQNNSNTQVINPPN-STQ |
| HPU07145_NCTC11638_K1GA2SV | YKDKPNNTP--SQSGAKNDKNESAKNDKQES-----------SQNNSNTQVINPPN-SAQ |
| S72494_S72494_K1GA2SV | YKDKPNNTP--SQSGAKNDKNESAKNDKQES-----------SQNNSNTQVINPPN-SAQ |
| RJHK01000001_ZH97_K1GA2SV | YKDKPTYNTP-SQSGTKNDKNESAKNDKQES-----------GQNNSNTQVINPPN-SAQ |
| RJGQ01000022_ZH72_QGA2SV | YKNQTNNTP--SQSGAKNDKNESAKNDKQES-----------SQNNSNTQVINPPN-STQ |
| NC_008086_HPAG1_QGA2SV | YKNQTNNTP--SQSGAKNDKNESAKNDKQES-----------SQNNSNTQVINPPN-STQ |
| NZ_QBPJ01000020_55:2_QGA2SV | YKNQTNNTP--SQSGAKNDKNESAKNDKQES-----------SQNNSNTQVINPPN-SAQ |
| LC420366_Merauke37_EA2SV | YESKTKD-------TPKNDKNESAKNDKQES-----------AQNNSNTQVINPPN-SAQ |
| LC420374_Merauke21_EA2SV | YESKTKD-------TPKNDKNESAKNDKQES-----------AQNNSNTQVINPPN-SAQ |
| LC420373_Merauke12_EA2SV | YESKTKD-------TPKNDKNESAKNDKQES-----------AQNNSNTQVINPPN-SAQ |
| LC420376_Merauke3_EA2SV | YESKTKD-------TPKNDKNESTKNDKQES-----------AQNNSNTQVINPPN-SAQ |
| LC420375_Merauke27_EA2SV | YESKTKD-------TPKNDKNESAKNDKQES-----------AQNNSNTQVINPPN-SAQ |
| LC420380_Merauke8_EA2SV | YESKTKD-------TPKNDKNESAKNDKQES-----------AQNNSNTQVINPPN-SAQ |
| LC420377_Merauke5_EA2SV | YESKTKD-------TPKNDKNESAKNDKQES-----------AQNNSNTQVINPPN-SAQ |
| LC420378_Merauke7_EA2SV | YESKTKD-------TPKNDKNESTKNDKQES-----------AQNNSNTQVINPPN-SAQ |
| NZ_MVSL01000008_HPJ050_K2TNSV | YKDKPKDKP--SNTTQNN-----ANNNQQNS-----------AQNNNNTSVINPPN-SAQ |
| NZ_MVSU01000020_HPAS14_QGA2SV2 | YKNQTNNTP--SQSGAKNDKNESAKNDKQESSQNNSNTQVINPPDNSNTQVINPPN-STQ |
| AF071097_F79_K1GA2V | YKDKPNNTN--SQSVGKSDKNESAKNDKQDS----------------NTQVINPPN-SGQ |
| LC187585_BH10_K1GA2SV | YKDKPNNTP--SQSGAKNDKNESAKNDKQES-----------SQNNSNTQVINPPN-SAQ |
| GQ331983_L8_K1GA1SV | YKDKPNNTP--SQSGAKNDKQES-------------------SQNNSNTQVINPPN-STQ |
| NZ_PHMP01000010_KH9_K1GA1SV | YKDKPNNTP--SQSGAKNDKQES-------------------SQNNSNTQVINPPN-SAQ |
| NZ_PHME01000012_KH20_K1GA2SV | YKDKPNNTP--SQSGTKNDKNESAKNDKQES-----------SQNNSNTQVINPPN-SAQ |
| LC187582_BH95_K1GA2V | YKDKPNNTP--SQSGTKSDKNESAKNDKQDS----------------NTQVINPPN-SGQ |
| NZ_AONK01000048_UM018_K1GA2SV | YKDKPNNTP--SQSGAKNDKNESAKNDKQES-----------SQNNSNTQVINPPN-SAQ |
| NZ_PHMN01000009_KH11_K1GA2SV | YKDKPNNTP--SQSGAKNDKNESAKNDKQES-----------SQNNSNTQVINPPN-SAQ |
| NZ_LFCB01000021_UM158_K1GA2SV | YKDKPNNTP--SQSGAKNDKNESAKNDKQES-----------SQNNSNTQVINPPN-SAQ |
| GQ331975_PG225_K1GA3SV | YKDKPNNTP--SQSGAKNDKNESAKNDKNESAKNDKQES---SQNNSNTQVINPPN-SGQ |
| GQ331976_PG227_K1GA3SV | YKDKPNNTP--SQSGAKNDKNESAKNDKNESAKNDKQES---SQNNSNTQVINPPN-SAQ |
| NZ_PHLO01000022_KH37_K1GA1SV | YKDKPNNTP--SQSGAKNDKQES-------------------SQNNSNTQVINPPN-SAQ |
| LC187574_BH94_K1GA2V | YKDKPNNTP--SQSGTKNDKNESAKNDKQDS----------------NTQVINPPN-SGQ |
| NZ_PHMH01000021_KH17_K1GA2SV | YKDKPNNTP--SQSGAKNDKNESAKNDKQES-----------SQNNSNTQVINPPN-SAQ |
| NZ_LFBX01000009_UM122_K1GA2SV | YKDKPNNTP--SQSGAKNDKNESAKNDKQES-----------SQNNSNTQVINPPN-SAQ |
| NZ_MVTD01000002_HP99316_K1GA2SV | YKDKPNNTP--SQSGTKNDKNESAKNDKQES-----------SQNNSNTQVINPPN-SAQ |
| NZ_AJFA02000020_NAB47_K1GA1SV | YKDKPNNTP--SQSGAKNDKQES-------------------SQNNSNTQVINPPN-SAQ |
| NZ_PHMA01000021_KH25_K1GA2SV | YKDKPNNTP--SQSGAKNDKNESAKNDKQES-----------SQNNSNTQVINPPN-SAQ |
| NZ_AUSO01000022_UM084_K1GA2SV | YKDKPNNTP--SQSGAKNDKNESAKNDKQES-----------SQNNSNTQVINPPN-SGQ |
| NZ_LFKM01000008_UM411_K1GA2SV | YKDKPNNTP--SQSGTKNDKNESAKNDKQES-----------SQNNSNTQVINPPN-SAQ |
| NZ_CBQE010000027_SA222A_K1GA2SV | YKDKPNNTP--SQSGAKNDKNESAKNDKQES-----------SQNNSNTQVINPPN-SAQ |
| NZ_PHMK01000014_KH14_K1GA3SV | YKDKPNNTP--SQSGAKNDKNESAKNDKNESAKNDKQES---SQNNSNTQVINPPN-SAQ |
| NZ_PHMG01000026_KH18_K1GA2SV | IKDKPNNTP--SQSGAKNDKNESAKNDKQES-----------SQNNSNTQVINPPN-SAQ |
| NZ_AUSI01000043_UM037_K1GA2V | YKDKPNNTP--SQSGTKNDKNESAKNDKQDS----------------NTQVINPPN-SAQ |
| LC187580_BH133_K1GA2SV | YKDKPNNTP--SQSGTKNDKNESAKNDKQES-----------SQNNSNTQVINPPN-SGQ |
| GQ331978_MZ4_K1GA2SV | YKDKPNNTP--SQSGAKNDKNESAKNDKQES-----------AQNNSNTQVINPPN-SGQ |
| 4473_NP05_107_K1GA2SV | YKDKPNNTP--SQSGAKNDKNESAKNDKQES-----------SQNNSNTQVINPPN-SGQ |
| 4467_NP05_250_K1GA2SV | YKDKPNNTP--SQSGAKNDKNESAKNDKQES-----------SQNNSNTQVINPPN-SAQ |
| NZ_PHMB01000011_KH23_K1GA2SV | YKDKPNNTP--SQSGAKNDKNESTKNDKQES-----------SQNNSNTQVINPPN-SGQ |
| NZ_LFKH01000001_UM228_K1GA2SV | YKDKPNNTP--SQSGAKNDKNESAKNDKQES-----------SQNNSNTQVINPPN-SVQ |
| NZ_PHLJ01000013_KH43_K1GA2SV | YKDKPNNTP--SQSGAKNDKNESAKNDKQES-----------SQNNSNTQVINPPN-SGQ |
| 4453_Yangon190_K1GA2SV | YKDKPNNTP--SQSGAKNDKNESAKNDKQES-----------SQNNSNTQVINPPN-SAQ |
| NZ_PHMJ01000009_KH15_K1GA2SV | YKDKPNNTP--SQSGAKNDKNESAKNDKQES-----------SQNNSNTQVINPPN-STQ |
| LLVY01000008_UM400AS_K1GA1SV | YKDKPNNTP--SQSGAKNDKQES-------------------SQNNSNTQVINPPN-SGQ |
| NZ_LQNC02000033_UM400AR_K1GA1SV | YKDKPNNTP--SQSGAKNDKQES-------------------SQNNSNTQVINPPN-SGQ |
| NZ_MOEE01000018_UM400bM_K1GA1SV | YKDKPNNTP--SQSGAKNDKQES-------------------SQNNSNTQVINPPN-SGQ |
| NZ_MOEF01000017_UM400b_K1GA1SV | YKDKPNNTP--SQSGAKNDKQES-------------------SQNNSNTQVINPPN-SGQ |
| 4475_NP04_K1GA2SV | YKDKPNNTL--SQSGAKNDKNESAKNDKQES-----------SQNNSNTQVINPPN-SGQ |
| NZ_LFDR01000011_UM087_K1GA2SV | YKDKPNNTP--SQSGAKNDKNESAKNDKQES-----------SQNNSNTQVINPPN-SAQ |
| NZ_PHLR01000011_KH34_K1GA2SV | YKDKPNNIP--SQSGAKNDKNESAKNDKQES-----------SQNNSNTQVINPPN-SGQ |
| NZ_PHLU01000022_KH31_K1GA2SV | YKDKPNNTP--SQSGAKNDKNESAKNDKQES-----------SQNNSNTQVINPPN-STQ |
| NZ_LFIS01000012_UM152_K1GA2SV | YKDKPNNTP--SQSGAKNDKNESAKNDKQES-----------SQNNSNTQVINPPN-SAQ |
| NZ_PHLM01000010_KH39_K1GA2SV | YKDKPNNTP--SQSGAKNDKNESAKNDKQES-----------SQNNSNTQVINPPN-SAQ |
| 3658_3754_K1GA2SV | YKDKPNNTP--SQSGAKNDKNESAKNDKQES-----------SQNNSNTQVINPPN-SGQ |
| 4450_Yangon233_K1GA2SV | YKDKPNSTN--SQSGAKNDKNKSAKNDKQES-----------SQNNSNTQVINPPN-SGQ |
| LC187566_BH86_K1GA2SV | YKDKPNNTP--SQSGAKNDKNESAKNDKQES-----------SQNNSNTQVINPPN-SAQ |
| NZ_AUSS01000022_UM114_K1GA2SV | YKDKPNNTP--SQSGAKNDKNESAKNDKQES-----------SQNNSNTQVINPPN-SGQ |
| LC187581_BH82_K1GA2SV | YKDKPNNTP--SQSGAKNDKNESAKNDKQES-----------SQNNSNTQVINPPN-SGQ |
| NZ_MVSX01000021_HP99647_K1GA2V | YKDKPNNTP--SQSGAKNDKNESAKNDKQDS----------------NTQVINPPN-SAQ |
| LC420379_Kolaka82_K1GA2SV | YKDKPNNTL--SQSGAKNDKNESAKNDKQES-----------SQNNSNTQVINPPN-SGQ |
| LC187578_BH73_K1GA2SV | YKDKPNNTN--SQSGAKNDKNESAKNDKQES-----------SQNNSNTQVINPPN-SGQ |
| LC187565_BH52_K1GA2SV | YKDKPNNTP--SQSGAKNDKNESAKNDKQES-----------SQNNSNTQVINPPN-SAQ |
| NZ_PHLH01000020_KH45_K1GA2SV | YKDKPNNTP--SQSGAKNDKNESAKNDKQES-----------SQNNSNTQVINPPN-SAQ |
| NZ_PHLT01000018_KH32_K1GA2SV | YKDKPNNTP--SQSGAKNDKNESAKNDKQES-----------SQNNSNTQVINPPN-SGQ |
| NZ_LFIU01000001_UM408_K1GA2V | YKDKPNNTP--SQSGAKNDKNESAKNDKQDS----------------NTQVINPPN-SGQ |
| LC187564_BH130_K1GA3V | YKDKPNNTP--SQSGAKNDKNESAKNDKNESAKNDKQDS--------NTQVINPPN-SGQ |
| LC187567_BH115_K1GA2V | YKDKPNNTP--SQSGTKNDKNESAKNDKQDS----------------NTQVINPPN-SGQ |
| LC187572_BH120_K1GA2SV | YKDKPNSTN--SQSSAKNDKNESAKNDKQES-----------SQNNSNTQVINPPN-SGQ |
| LC187563_BH3_K1GA2SV | YKDKPNNTP--SQSGAKNDKNESAKNDKQES-----------SQNNSNTQVINPPN-SGQ |
| 4452_Yangon202_K1GA2SV | YKDKPNNTP--SQSGAKNDKNESAKNDKQES-----------SQNNSNTQVINPPN-SGQ |
| LC187560_BH49_K1GA2SV | YKDKPNNTP--SQSGAKNDKNESAKNDKQES-----------SQNNSNTQVINPPN-SAQ |
| 4476_Myanmar66_K1GA2V | YKDKPNNTP--SQSGAKNDKNESAKNDKQDS----------------NTQVINPPN-SGQ |
| 4458_Yangon142_K1GA2V | YKDKPNNTP--SQSGAKNDKNESTKNDKQDS----------------NTQVINPPN-SGQ |
| 4478_Myanmar51_K1GA2V | YKDKPNNTP--SQSGAKNDKNESAKNDKQDS----------------NTQVINPPN-SGQ |
| LC187559_BH13_K1GA2V | YKDKPNNTP--SQSGAKNDKNESAKNDKQDS----------------NTQVINPPN-SGQ |
| GQ331984_L1_K1GA2V | YKDKPNSTN--SQSGAKNDKNESAKNDKQDS----------------NTQVINPPN-SGQ |
| 4471_NP05_121_K1GA2SV | YKDKPNNTP--SQSGAKNDKNESAKNDKQES-----------AQNNSNTQVINPPN-STQ |
| LC187583_BH104_K1GA2V | YKDKPNNTP--SQSGAKNDKNESAKNDKQDS----------------NTQVINPPN-SAQ |
| NZ_LFLE01000040_UM147_K1GA2V | YKDKPNNTP--SQSGAKNDKNESAKNDKQDS----------------NTQVINPPN-SGQ |
| LC187570_BH72_K1GA2SV | YKDKPNNTP--SQSGAKNDKNESAKNDKQES-----------SQNNSNTQVINPPN-SGQ |
| LC187569_BH69_K1GA2V | YKDKPNNTP--SQSGAKNDKNESAKNDKQDS----------------NTQVINPPN-SGQ |
| LC187561_BH91_K1GA2V | YKDKPNNTT--SQSGAKNDKNESAKNDKQDS----------------NTQVINPPN-SGQ |
| RJGJ01000009_ZH63_K1GA2V | YKDKPNNTP--SQSGAKNDKNESAKNDKQDS----------------NTQVINPPN-SAQ |
| 4474_NP05_105_K1GA2V | YKDKPNNTN--SQSGAKNDKNESAKNDKQDS----------------NTQVINPPN-SGQ |
| NZ_MVTT01000019_HP15054_K1GA2V | YKDKPDNTN--SQSGAKNDKNESAKNDKQDS----------------NTQVINPPN-SGQ |
| NZ_LFKE01000027_UM202_K1GA2V | YKDKPNNTP--SQSGAKNDKNESAKNDKQDS----------------NTQVINPPN-SGQ |
| GQ331981_DL2_K1GA2V | YKDKPNNTP--SQSGAKNDKNESAKNDKQDS----------------NTQVINPPN-SGQ |
| NZ_AJGJ02000042_NAD1_K1GA2V | YKDKPNNTP--SQSGAKNDKNESAKNDKQDS----------------NTQVINPPN-SGQ |
| LC187577_BH112_K1GA1V | YKDKPNNTP--SQSGAKNDKQDS------------------------NTQVINPPN-SGQ |
| LC187584_BH16_K1GA2V | YKDKPNNTP--SQSGAKNDKNESAKNDKQDS----------------NTQVINPPN-SGQ |
| LC187573_BH119_K1GA2V | YKDKPNNNP--SQSGAKNDKNESAKNDKQDS----------------NTQVINPPN-SAQ |
| 4477_Myanmar52_K1GA2V | YKDKPNNTP--SQSGAKNDKNESAKNDKQDS----------------NTQVINPPN-SAQ |
| LC187575_BH107_K1GA2V | YKDKPNNTP--SQSGAKNDKNESAKNDKQDS----------------NTQVINPPN-SAQ |
| LC187568_BH14_K1GA2V | YKDKPNNTP--SQSGAKNDKNESAKNDKQDS----------------NTQVINPPN-SGQ |
| 4455_Yangon179_K1GA2V | YKDKPNNTP--SQSGTKNDKNESAKNDKQDS----------------NTQVINPPN-SGQ |
| LC187576_BH63_K1GA2V | YKDKPNNTP--SQSGAKNDKNESAKNDKQDS----------------NTQVINPPN-SGQ |
| 4484_Mandalay03_K1GA2V | YKDKPNSTN--SQSGAKNDKNESAKNDKQDS----------------NTQVINPPN-SGQ |
| LC187562_BH109_K1GA2V | YKDKPNSTN--SQSGAKNDKNESAKNDKQDS----------------NTQVINPPN-SGQ |
| NC_019563_Aklavik86_TNS | YKRPPNGATQ--NNANNNQNN---------------------NQNNN----NASNS-QNN |
| AF191641_AFN1156_K1GP | YKDKPNNTPS--QSN--------------------------------------PKN-DTQ |
| NZ_KB644619_GAMchJs114i_K1GP | YKDKPNNTPS--QNN--------------------------------------PKN-DTQ |
| 749_GAMchjs117Ai_EP | YESKTKDTPSQNN----------------------------------------PKN-DTQ |
| NZ_KB642376_GAMchJs106B_EP | YESKTKDTPSQNN----------------------------------------PKN-DTQ |
| NZ_CBMW010000003_SA303C_EP | YESKTKDTPSQNN----------------------------------------PKN-ETQ |
| AVNI01000002_SouthAfrica50_EP | YESKTKDTPSQNN----------------------------------------PKN-DTQ |
| NZ_CBQC010000007_SA251A_EP | YKGKNKDT-SQGG----------------------------------------TKN-DTQ |
| NZ_CP011486_K26A1_EP | YKGKNKDT-SQGG----------------------------------------TKN-DTQ |
| NZ_CBOK010000012_SA169C_EP | YKGKNKDT-SQGG----------------------------------------TKN-DTQ |
| NZ_CBQH010000012_SA169A_EP | YKGKNKDT-SQGG----------------------------------------TKN-DTQ |
| NZ_CBNN010000004_SA40A_EP | YKGKNKDT-SQGG----------------------------------------TKN-DTQ |
| NZ_CBPS010000012_SA174A_EP | YKGKNKDT-SQGG----------------------------------------TKN-DTQ |
| NZ_CBOI010000003_SA175C_EP | YKGKNKDT-SQGG----------------------------------------TKN-DTQ |
| NZ_CBPO010000003_SA175A_EP | YKGKNKDT-SQGG----------------------------------------TKN-DTQ |
| NZ_MVWP01000007_HP12054_EP | YKGKNKDT-SQGG----------------------------------------TKN-DTQ |
| NZ_CBMX010000001_SA233A_EP | YKGKNKDT-SQGG----------------------------------------TKN-DTQ |
| NZ_CBPD010000001_SA233C_EP | YKGKNKDT-SQGG----------------------------------------TKN-DTQ |
| NZ_CBOX010000007_SA144C_EP | YKGKNKDT-SQGG----------------------------------------TKN-DTQ |
| NZ_CBOZ010000002_SA251C_EP | YKGKNKDT-SQGG----------------------------------------TKN-DTQ |
| NZ_CBPK010000031_SA213C_EP | YKGKNKDT-SQGG----------------------------------------TKN-DTQ |
| NZ_CBNK010000001_SA194C_EP | YKGKNKDT-SQGG----------------------------------------TKN-DTQ |
| NZ_CBQI010000001_SA36C_EP | YKGKNKDT-SQGG----------------------------------------TKN-DTQ |
| NZ_CBQF010000015_SA160C_EP | YKGKNKDT-SQGG----------------------------------------TKN-DTQ |
| NZ_CBPG010000001_SA172C_EP | YKGKNKDT-SQGG----------------------------------------TKN-DTQ |
| NZ_CBOJ010000015_SA253A_EP | YKGKNKDT-SQGG----------------------------------------TKN-DTQ |
| NZ_CBNY010000041_SA34C_EP | YKGKNKDT-SQGG----------------------------------------TKN-DTQ |
| NZ_CBOH010000005_SA155C_EP | YKGKNKDT-SQGG----------------------------------------TKN-DTQ |
| NZ_CBOT010000009_SA47A_EP | YESKTKDNPSQNN----------------------------------------PKN-DTQ |
| NZ_CBOO010000001_SA37A_EP | YESKTKDNPSQNN----------------------------------------PKN-DTQ |
| NZ_CBOP010000040_SA37C_EP | YESKTKDNPSQNN----------------------------------------PKN-DTQ |
| NZ_MVYB01000012_HP04042_K1TNSV | YKDKPSNTTQNN--ANNNQQNS--------------------AQNNSNTQVINPPN-SVQ |
| LC187596_BH97_EP | YESKTKDTPSQNN----------------------------------------PKN-DTQ |
| NZ_CBPT010000020_SA221C_EP | YESKTKDTPSQNN----------------------------------------PKN-DTQ |
| NZ_CBQA010000002_SA221A_EP | YESKTKDTPSQNN----------------------------------------PKN-DTQ |
| 4564_Nic20_A_EP | YESKTKDTPSQNN----------------------------------------PKN-DTQ |
| RJIX01000034_ZH138_EP | YESKTKDTPSQNN----------------------------------------PKN-DTQ |
| RJFH01000010_ZH34_EP | YESKTKDNP-QNN----------------------------------------PKN-DAQ |
| NZ_QEGO01000012_B712A_EP | YESKTKDNPSQNN----------------------------------------PKN-DTQ |
| NZ_MBIC01000139_22395_EP | YESKTKDNHSQNN----------------------------------------PKN-DTQ |
| NZ_QEGJ01000012_JGF25_EP | YESKTKDTP-QNN----------------------------------------PKN-DTQ |
| RJIT01000005_ZH134_EP | YESKTKDTPSQNN----------------------------------------PKN-DAQ |
| NZ_MVXM01000008_HP08073_EP | YESKTKDNPSQNN----------------------------------------PKN-DTQ |
| NZ_JSXX01000062_173/00_EP | YESKTKDTPSQNN----------------------------------------PKN-DTQ |
| 3647_3800_EP | YESKTKETPSQNN----------------------------------------PKN-DAQ |
| NZ_PHLN01000005_KH38_EP | YESKTKDTPSQNN----------------------------------------PKN-DTQ |
| RJGX01000018_ZH82_EP | YESKTKDTPSQNN----------------------------------------PKN-DTQ |
| RJIF01000014_ZH120_EP | YESKTKDTPSQNN----------------------------------------PKN-DTQ |
| 8776_22384_EP | YESKTKDTPSQNN----------------------------------------PKN-DAQ |
| NZ_PHMS01000025_KH6_EP | YESKTKDNP-QNN----------------------------------------PKN-DAQ |
| RJFJ01000010_ZH36_EP | YESKTKDTP-QNN----------------------------------------PKN-DTQ |
| RJIH01000021_ZH122_EP | YESKTKDTP-QNN----------------------------------------PKN-DTQ |
| 4540_Nic08_C2_EP | YESKTKDTPSQNN----------------------------------------PKN-DTQ |
| NZ_QELC01000010_B319_EP | YESKTKDN-PQNN----------------------------------------PKN-DAQ |
| RJEB01000002_ZH3_EP | YESKTKDN-PQNN----------------------------------------PKN-DTQ |
| NZ_MVVZ01000021_HP13021_EP | YESKTKDN-PQNN----------------------------------------PKN-DTQ |
| NZ_MVUY01000012_HP14054_EP | YESKTKDTTSQNN----------------------------------------PKN-DTQ |
| NZ_QEGZ01000011_B366_EP | YESKTKDTPSQNN----------------------------------------PKN-DAQ |
| NZ_MBHT01000057_A039_EP | YESKTKDNPSQNN----------------------------------------PKN-DAQ |
| NZ_MBJX01000026_22389_EP | YESKTKDNPSQNN----------------------------------------PKN-DTQ |
| NZ_MUPE01000054_CG22389_EP | YESKTKDNPSQNN----------------------------------------PKN-DTQ |
| NZ_JSXY01000029_228/99_EP | YESKTKDNPSQNN----------------------------------------PKN-DAQ |
| NZ_MBHB01000011_22025_EP | YESKTKDNPSQNN----------------------------------------PKN-DTQ |
| NZ_MUPJ01000099_CM22331_EP | YESKTKDNPSQNN----------------------------------------PKN-DTQ |
| NZ_QDJQ01000063_B444A_EP | YESKTKDTPSQNN----------------------------------------PKN-DAQ |
| 8764_22093_EP | YESKTKDTPSQNN----------------------------------------PKN-DAQ |
| NZ_MBIV01000002_22339_EP | YESKTKDTPSQNN----------------------------------------PKN-DAQ |
| NZ_MUOP01000270_CC22093_EP | YESKTKDTPSQNN----------------------------------------PKN-DAQ |
| NC_019560_Aklavik117_EP | YESKTKDN---------------------------------------------PKN-DTQ |
| NZ_PHLQ01000010_KH35_EP | YESKTKDTP-QNN----------------------------------------PKN-DTQ |
| 3617_SSR5_EP | YESKTKDTPSQNN----------------------------------------PKN-DTQ |
| NZ_PHLV01000002_KH30_EP | YESKTKDTPSQNN----------------------------------------PKN-DTQ |
| LC187612_BH127_EP | YESKTKDTPSQNS----------------------------------------PKN-DTQ |
| RJEQ01000015_ZH17_EP | YESKTKDTPSQNN----------------------------------------PKN-DTQ |
| NZ_MVTM01000014_HP16008_EP | YESKTKDTP-QNN----------------------------------------PKN-DAQ |
| RJHM01000002_ZH99_EP | YESKTKDNPSQNN----------------------------------------PKN-DVQ |
| NZ_QDJI01000006_B657-A1_EP | YESKTKDNPSQNN----------------------------------------PKN-DAQ |
| NZ_PHMI01000024_KH16_EP | YESKTKDTP-QNN----------------------------------------PKN-DTQ |
| RJHD01000001_ZH88_EP | YESKTKDTPSQNN----------------------------------------PKN-DAQ |
| NZ_AONL01000042_UM054_EP | YESKTKDNPSQNN----------------------------------------PKN-DAQ |
| NZ_MVYD01000002_HP03218_EP | YESKTKDNPSQNN----------------------------------------PKN-DTQ |
| RJFN01000002_ZH40_EP | YESKTKDTPSQNN----------------------------------------PKN-DAQ |
| NZ_QDJM01000009_B508A-T2A_EP | YESKTKDNPSQNN----------------------------------------PKN-DTQ |
| LC187603_BH96_EP | YESKTKDTP-QNN----------------------------------------PKN-DTQ |
| NZ_PHLW01000004_KH29_EP | YESKTKDTP-QNN----------------------------------------PKN-DAQ |
| RJGK01000006_ZH65_EP | YESKTKDNPSQNN----------------------------------------PKN-DAQ |
| NZ_AKPU01000004_HpP-30_EP | YESKTKDNPSQNN----------------------------------------PKN-DTQ |
| RJHN01000004_ZH100_EP | YESKTKDNPSQNN----------------------------------------PKN-DTQ |
| NZ_MVWL01000020_HP12068_EP | YESKTKDTPSQNN----------------------------------------PKN-DTQ |
| NZ_QDJG01000015_B657-C1_EP | YESKTKDTPSQNN----------------------------------------PKN-DAQ |
| NZ_QDJH01000011_B657-A4_EP | YESKTKDTPSQNN----------------------------------------PKN-DAQ |
| RJFM01000012_ZH39_EP | YESKIKDTPSQNN----------------------------------------PKN-DTQ |
| RJEA01000003_ZH02_EP | YESKTKDTPSQNN----------------------------------------PKN-DTQ |
| NZ_QBRS01000028_B25_EP | YESKIKDTPSQNN----------------------------------------PKN-DTQ |
| NZ_CP012907_29CaP_EP | YESKTKDTPSQNN----------------------------------------PKN-DAQ |
| LC187600_BH84_EP | YESKTKDTPSQNN----------------------------------------PKN-DAQ |
| LC187599_BH114_EP | YESKTKDNPSQNN----------------------------------------PKN-DTQ |
| NZ_UGHN01000001_NCTC13207_EP | YESKTKDTPSQNN----------------------------------------PKN-DTQ |
| RJFT01000009_ZH47_EP | YESKTKDNPSQNN----------------------------------------PKN-DTQ |
| RJGH01000021_ZH61_EP | YESKTKDTPSQNN----------------------------------------PKN-DAQ |
| NZ_QDJL01000011_B508A-T4_EP | YESKTKDNPSQNN----------------------------------------PKN-DTQ |
| NZ_QDJN01000008_B508A-S1_EP | YESKTKDNPSQNN----------------------------------------PKN-DTQ |
| RJFR01000038_ZH45_EP | YESKTKDTHSQNN----------------------------------------PKN-DTQ |
| LC420372_Kolaka56_K1GA2 | YKDKPNNTN--SQSGAKNDKNESAKNDKQDS----------------NTQVINPPN-SEQ |
| NZ_LFKL01000019_UM370_K1GA2V | YKDKPNNTN--SQSGAKNDKNESAKNDKQDS----------------NTQVINPPN-SGQ |
| AF050319_CHN3295b_K1GA2SV | YKDKPNNTN--SQSGAKNDKNESAKNDKQES-----------SPNNSNTQVINPPN-SGQ |
| RJFU01000016_ZH48_K1GA2V | YKDKPNNTN--SQSGAKNDKNESAKNDKQDS----------------NTQVINPPN-SGQ |
| NZ_AUSQ01000029_UM077_K1GA2V | YKDKPNNTN--SQSGAKNDKNESAKNDKQDS----------------NTQVINPPN-SGQ |
| NZ_LFBY01000015_UM119_K1GA2V | YKDKPNNTN--SQSGAKNDKNESAKNDKQDS----------------NTQVINPPN-SGQ |
| AF050327_CHN5114a_K1GA2V | YKDKPNNTN--SQSGAKNDKNESAKNDKQDS----------------NTQVINPPN-SGQ |
| NZ_JAAA01000007_HLJ039_K1GA2V | YKDKPNNTN--SQSGAKNDKNESAKNDKQDS----------------NTQVINPPN-SGQ |
| AF050320_CHN5147c_K1GA2V | YKDKPNNTN--SQSGAKNDKNESAKNDKQDS----------------NTQVINPPN-SGQ |
| AF050326_CHN1811a_K1GA2V | YKDKPNNTN--SQSGAKNDKNESAKNDKQDS----------------NTQVINPPN-SGQ |
| NZ_MVWG01000002_HP12078_K1GA2SV | YKDKPNNTN--SQSGTKNDKNESAKNDKQES-----------SQNNSNTQVINPPN-SGQ |
| NZ_MVWS01000017_HP12036_K1GA2V | YKDKPNNTN--SQSGAKNDKNESAKNDKQDS----------------NTQVINPPN-SGQ |
| NZ_LJXP02000019_UM233S_K1GA2V | YKDKPNNTN--SQSGAKSDKNESAKNDKQDS----------------NTQVINPPN-SGQ |
| NZ_LJXQ02000015_UM233R_K1GA2V | YKDKPNNTN--SQSGAKNDKNESAKNDKQDS----------------NTQVINPPN-SGQ |
| NZ_LJXN02000018_UM137S_K1A2SV | YKDKPTQ------SGTKNDKNESAKNDKQES-----------SQNNSNTQVINPPN-SAQ |
| NZ_LJXO02000008_UM137R_K1GA2V | YKDKPNNTN--SQSGAKNDKNESAKNDKQDS----------------NTQVINPPN-SGQ |
| LC420371_Kolaka99_K1GA2SV | YKDKPNNTN--SQSGAKNDKNESAKNDKQES-----------SQNNSNTQVINPPN-SGQ |
| LC420355_Medan50_K1GA2SV | YKDKPNNTP--SQSGAKNDKNESAKNDKQES-----------SQNNSNTQVINPPN-SAQ |
| NZ_CP022409_G272_K1GA2SV | YKDKPNNTN--SQSGAKNDKNESAKNDKQDS-----------SQNNSNTQVINPPN-SGQ |
| NZ_LJXI02000018_UM276S_K1GA2V | YKDKPNNTN--SQSGAKNDKNESAKNDKQDS----------------NTQVINPPN-SGQ |
| NZ_LJXK02000019_UM276R_K1GA2V | YKDKPNNTN--SQSGAKNDKNESAKNDKQDS----------------NTQVINPPN-SGQ |
| NZ_JH791472_HLJHP271_K1GA2SV | YKDKPNNTN--SQSGAKNDKNESAKNDKQES-----------GQNNSNTQVINPPN-SGQ |
| NZ_MVVL01000013_HP13063_K1GA2V | YKDKPNNTN--SQSGAKSDKNESAKNDKQDS----------------NTQVINPPN-SGQ |
| NZ_JH791471_HLJHP256_K1GA2V | YKDKPNNTN--SQSGAKNDKNESAKNDKQDS----------------NTQVINPPN-SGQ |
| AB190988_OK210_K1GA2V | YKDKPNNTN--SQSGAKSDKNESAKNDKQDS----------------NTQVINPPN-SGQ |
| NZ_AUSR01000011_UM111_K1GA2V | YKDKPNNTN--SQSGAKNDKNESAKNDKQDS----------------NTQVINPPN-SGQ |
| NZ_CP031558_GD63_K1GA2V | YKDKPNNTN--SQSGAKNDKNESAKNDKQDS----------------NTQVINPPN-SGQ |
| NZ_MVUI01000017_HP15025_K1GA2V | YKDKPNNTN--SQSGAKNDKNESAKNDKQDS----------------NTQVINPPN-SGQ |
| NZ_MVUL01000003_HP15018_K1A2V | YKDKPN---S--QSGAKNDKNESAKNDKQDS----------------NTQVINPPN-SGQ |
| AB190972_OK129_K1GA2V | YKDKPNNTN--SQSGAKSDKNESAKNDKQDS----------------NTQVINPPN-SGQ |
| NZ_MJMU01000010_1177_K1GA2V | YKDKPNNTN--SQSGAKNDKNESAKNDKQDS----------------NTQVINPPN-SGQ |
| NZ_AMFG01000002_C333_K1GA2V | YKDKPNNTN--SQSGAKNDKNESAKNDKQDS----------------NTQVINPPN-SGQ |
| NZ_AONN01000028_UM034_K1GA2V | YKDKPNNTN--SQSGAKNDKNESAKNDKQDS----------------NTQVINPPN-SGQ |
| NZ_MVUM01000022_HP15015_K1GA2V | YKDKPNNTN--SQSGAKNDKNESAKNDKQDS----------------NTQVINPPN-SGQ |
| NZ_MVTW01000009_HP15044_K1GA2V | YKDKPNNTN--SQSGAKNDKNESAKNDKQDS----------------NTQVINPPN-SGQ |
| NZ_MVTN01000012_HP16004_K1GA2SV | YKDKPNNTN--SQSGAKNDKNESAKNDKQES-----------SQNNSNTQVINPPN-SGQ |
| NZ_MVTZ01000026_HP15036_K1GA2V | YKDKPNNTN--SQSGAKNDKNESTKNDKQDS----------------NTQVINPPN-SGQ |
| NZ_MVUK01000016_HP15020_K1GA2V | YKDKPNNTN--SQSGAKSDKNESAKNDKQDS----------------NTQVINPPN-SGQ |
| NZ_JPXC01000003_YN1-91_K1GA2SV | YKDKPNNTN--SQSGAKNDKNESAKNDKQES-----------SQNNSNTQVINPPN-SGQ |
| NZ_MVUX01000019_HP14056_K1GA2SV | YKDKPNNTN--SQSGAKSDKNESAKNDKQES-----------SQNNSNTQVINPPN-SGQ |
| NZ_AOTT01000005_CPY1662_K1GA2V | YKDKPNNTN--SQSGAKNDKNESAKNDKQDS----------------NTQVINPPN-SGQ |
| NZ_JH791474_HLJHP253_K1GA2V | YKDKPNNTN--SQSGAKNDKNESAKNDKQDS----------------NTQVINPPN-SGQ |
| NZ_LFKI01000005_UM246_K1GA2V | YKDKPNNTN--SQSGAKSDKNESAKNDKQES----------------NTQVINPPN-SGQ |
| NZ_MJIS01000006_241_K1GA2V | YKDKPNNTN--SQSGAKNDKNESAKNDKQDS----------------NTQVINPPN-SGQ |
| NZ_AONM01000014_UM007_K1GA2SV | YKDKPNNTN--SQSGAKNDKNESAKNDKQES-----------SQNNSNTQVINPPN-SGQ |
| NZ_LQND02000021_UM443S_K1GA2V | YKDKPNNTN--SQSGAKSDKNESAKNDKQDS----------------NTQVINPPN-SGQ |
| NZ_LQNE02000019_UM443R_K1GA2V | YKDKPNNTN--SQSGAKSDKNESAKNDKQDS----------------NTQVINPPN-SGQ |
| NC_021215_UM032_K1GA2V | YKDKPNNTN--SQSGAKNDKNESAKNDKQDS----------------NTQVINPPN-SGQ |
| NC_021218_UM066_K1GA2V | YKDKPNNTN--SQSGAKNDKNESAKNDKQDS----------------NTQVINPPN-SGQ |
| NZ_JQNY01000051_Taiwan-47_K1GA2V | YKDKPNNTN--SQSGAKNDKNESAKNDKQDS----------------NTQVINPPN-SGQ |
| NZ_MVUE01000022_HP15031_K1GA2V | YKDKPNNTN--SQSGAKNDKNESAKNDKQDS----------------NTQVINPPN-SGQ |
| NZ_MVUS01000003_HP15004_K1GA2V | YKDKPNNTN--SQSGAKNDKNESAKNDKQDS----------------NTQVINPPN-SGQ |
| NZ_AP014710_ML1_K1GA2SV | YKDKPNNTN--SQSGAKNDKNESAKNDKQDS-----------SQNNSNTQVINPPN-SGQ |
| NZ_LFJR01000012_UM163_K1GA2V | YKDKPNNTN--SQSGAKNDKNESAKNDKQDS----------------NTQVINPPN-SGQ |
| NZ_LLVW02000003_UM163S_K1GA2V | YKDKPNNTN--SQSGAKNDKNESAKNDKQDS----------------NTQVINPPN-SGQ |
| NZ_LLVX02000004_UM163R_K1GA2V | YKDKPNNTN--SQSGAKNDKNESAKNDKQDS----------------NTQVINPPN-SGQ |
| NZ_MVWA01000023_HP13013_K1GA2SV | YKDKPNNTN--SQSGAKNDKNESAKNDKQES-----------SQNNSNTQVINPPN-SGQ |
| NZ_AUSP01000020_UM085_K1GA2V | YKDKPNNTN--SQSGAKNDKNESAKNDKQDS----------------NTQVINPPN-SGQ |
| NZ_QDJU01000019_B247A_QGA2SV | YKNQTNNTP--SQSGAKNDKNESAKNDKQES-----------SQNNSNTQVINPPN-STQ |
| RJIN01000042_ZH128_K1GA1SV | YKDKPNNTP--SQSGAKNDKQES-------------------SQNNSNTQVINPPN-SAQ |
| NZ_LELJ01000007_UM045_K1GA2V | YKDKPNNTP--SQSGAKNEKNESAKNDKQDS----------------NTQVINPPN-SAQ |
| NZ_PHLZ01000033_KH26_K1GA2SV | YKDKPNNTP--SQSGAKNDKNESAKNDKQES-----------SQNNSNTQVINPPN-STQ |
| NZ_PHMW01000021_KH1_K1GA2SV | YKDKPNNTP--SQSGAKNDKNESTKNDKQES-----------SQNNSNTQVINPPN-SAQ |
| NC_017376_Santal49_K1GA2SV | YKDKPNNTP--SQSGAKNDKNESAKNDKQES-----------SQNNSNTQVINPPN-SAQ |
| LC187589_BH46_K1GA2V | YKDKPNNTP--SQSGAKNDKNESAKNDKQDS----------------NTQVINPPN-SGQ |
| 4472_NP05_112_K1GA2SV | YKDKPNNTP--SQSGAKNDKNESAKNDKQES-----------SQNNSNTQVINPPN-SGQ |
| 4482_Mandalay30_K1GA2V | YKDKPNNTN--SQSGAKSDKNESAKNDKQDS----------------NTQVINPPN-SGQ |
| LC187588_BH47_K1GA2V | YKDKPNNTP--SQSGAKNDKNESAKNDKQDS----------------NTQVINPPN-SGQ |
| NZ_QBPU01000070_66_QGA2SV | YKNQTNNTP--SQSGAKNDKNESAKNDKQES-----------SQNNSNTQVINPPN-SAQ |
| RJFE01000023_ZH31_K1GA2SV | YKDKPNNTP--SQSGAKNDKNESAKNDKQES-----------SQNNSNTQVINPPN-SAQ |
| NZ_QBQH01000001_30950_K1GA1SV | YKDKPNNTP--SQSGAKNDKQES-------------------SQNNSNTQVINPPN-STQ |
| NZ_MVVC01000013_HP14048_K1GA2SV | YKDKPNNTP--SQSGAKNDKNESAKNDKQES-----------SQNNSNTQVINPPN-SAQ |
| NZ_PHLI01000009_KH44_K1GA2SV | YKDKPNNTP--SQSGAKNDKNESAKNDKQES-----------SQNNSNTQVINPPN-SAQ |
| NZ_PHLS01000003_KH33_K1GA2SV | YKDKPNNTP--SQSGTKNDKNESAKNDKQES-----------SQNNSNTQVINPPN-SAQ |
| LC187591_BH53_K1GA2SV | YKDKPNNTP--SQSGAKNDKNESAKNDKQES-----------SQNNSNTQVINPPN-SGQ |
| NZ_MJGG01000003_132_K1GA2SV | YKDKPNNTP--SQSGAKNDKNESAKNDKQES-----------SQNNSNTQVINPPN-SGQ |
| NZ_MJMX01000001_132A_K1GA2SV | YKDKPNNTP--SQSGAKNDKNESAKNDKQES-----------SQNNSNTQVINPPN-SGQ |
| NZ_PHLP01000005_KH36_K1GA2V | YKDKPNNTP--SQSGAKNDKNESAKNDKQDS----------------NTQVINPPN-SGQ |
| NC_017358_Cuz20_EP | YESKTKDNP-QNN----------------------------------------PKN-DTQ |
| NC_017741_Shi112_EP | YESKTKDNP-QNN----------------------------------------PKN-DTQ |
| RJFW01000009_Zh50_K1GA1SV | YKDKPNNTP--SQSGTKNDKQEI------------------SQNNNSNTQVINPPN-SAQ |
| LC187593_BH34_K1GA2V | YKDKPNSTN--SQSGAKNDKNESAKNDKQDS----------------NTQVINPPN-SGQ |
| NZ_MVUT01000020_HP15003_EP | YESKNKDN-----------------------------------------PQNNPKN-DTQ |
| NZ_LFKG01000003_UM211_EP | YESKTKDN---------------------------------------------PKN-DTQ |
| AB190981_OK180_EP | YESKNKDN-----------------------------------------PQNNPKN-DTQ |
| AB190982_OK181_EP | YESKNKDN-----------------------------------------PQNNPKN-DTQ |
| AB190973_OK130_EP | YESKNKDN-----------------------------------------PQNNPKN-DTQ |
| AB190986_OK204_EP | YESKNKDN-----------------------------------------PQNNPKN-DTQ |
| NZ_ANIO01000025_D33_EP | YESKTKDNP-QNN----------------------------------------PKN-DTQ |
| NZ_MVVM01000018_HP13061_EP | YESKTKDNP-QNN----------------------------------------PKN-DTQ |
| LC187592_BH36_EP | YESKTKDTPSQNN----------------------------------------PKN-DTQ |
| NZ_PHMT01000006_KH4_K1GA2SV | YKDKPNNTP--SQSGAKNDKNESAKNDKQES-----------SQNNSNTQVINPPN-SAQ |
| LC187590_BH101_K1GA2V | YKDKPNNTP--SQSGAKNDKNESAKNNKQDS----------------NTQVINPPN-SAQ |
| NZ_MVVQ01000024_HP13033_K1TNSV | YKDKP--------SNTTQNN---ANNNQQNS-----------TQNNSNTQVINPPN-SAQ |
| NZ_MVXI01000018_HP11005_EP | YESKTKDTPSQNN----------------------------------------PKN-DTQ |
| NZ_QBQA01000047_518_K1TNSV | YKDKP--------SNTTQNN---ANNNQQNS-----------AQNNSNTQVINPPN-SAQ |
| 3630_SSR33_K1TNSV | YKDKP--------SNTTQNN---ANNNQQNS-----------AQNNSNTQVINPPN-SAQ |
| NZ_QBPR01000050_23:2_single_K2TNSV | YKDKPKDKP--SNTTQNN-----ANNNQQNS-----------AQNNSNTQVINPPN-SAQ |
| 3620_SSR9_EP | YESKTKDNPSQNN----------------------------------------PKN-DAQ |
| NZ_CCMW01000020_H3018_K2TNSV | YKDKPKDKP--SNTTQNN-----ANNNQQNS-----------AQNNSNTQVINPPN-SAQ |
| HPU95971_95-54(J128)_K2TNSV | YKDKPKDKP--SNTTQNN-----ANNNQQNS-----------AQNNSNTQVINPPN-SAQ |
| NZ_QBQD01000017_448_K2TNSV | YKDKPKDKP--SNTTQNN-----ANNNQQNS-----------TQNNNNTSVINPPN-SAQ |
| LC420370_Kolaka98_E | YENKTKDTP-------------------------------------------------AQ |
| RJGD01000015_ZH57_K1GA2V | YKDKPNNTL--SQSGAKNDKNESAKNDKQDS----------------NTQVINPPN-STQ |
| RJHG01000002_ZH93_K1GA2SV | YKDKPNNTP--SQSGAKNDKNESAKNDKQES-----------SQNNSNTQVINPPN-NTQ |
| NZ_MVXZ01000003_HP04086_EP | YESKTKDTPSQNN----------------------------------------PKN-DTQ |
| NZ_MVXU01000030_HP06058_EP | YESKTKDTPSQNS----------------------------------------PKN-DTQ |
| 3624_SSR14_EP | YESKTKDNP-QNN----------------------------------------PKN-DTQ |
| RJEN01000003_ZH14_EP | YESKTKDNP-QNN----------------------------------------PKN-DTQ |
| NZ_AMOQ01000004_R018c_K2TNSV | YKDKPKDKP--SNTTQNN-----ANNNQQNS-----------AQNNNNTSVINPPN-SAQ |
| NZ_AMOY01000008_R056a_K2TNSV | YKDKPKDKP--SNTTQNN-----ANNNQQNS-----------AQNNNNTSVINPPN-SAQ |
| NZ_LFIT01000014_UM300_K1GA1SV | YKDKPNNTPS-----QSG-----AKNDKQES-----------SQNNSNTQVINPPN-DTQ |
| NZ_AKOC01000008_HpA-9_EP | YESKTKDTPSQNN----------------------------------------PKN-DTQ |
| NZ_QBPK01000026_55:1_K1TNSV | YKDKPSNT-T-----QNN-----ANNNQQNS-----------AQNNNNTSVINPPN-SAQ |
| NZ_LFIR01000013_UM165_EP | YESKTKDNPQ-----NN------------------------------------PKN-DTQ |
| NZ_MVWJ01000019_HP12070_EP | YESKTKDTPSQNN----------------------------------------PKN-DTQ |
| RJGG01000009_ZH60_ENSV | YESKTKDTPSQNN----------ANNNQQNS-----------TQNNSNTQVINPPN-SAQ |
| NZ_MVVU01000030_HP13027_EA1SV | YESKTKDNPSQNN----------PKNDKQES-----------SQNNSNTQVINPPN-SAQ |
| NZ_MVUJ01000025_HP15022_EP | YESKTKDNPSQNN----------PKNDT-------------------------------Q |
| NZ_QBQE01000073_38185_K1GA2V | YKDKPNSTT--SQSGTKNDKNESAKNDKQES--NN-----------SNTEVINPPN-NTQ |
| NZ_CBOC010000001_SA168C_K1GA1SV | YKDKPNNTT--SQSGTKNDKQEI------------------SQNNNSNTEVINPPN-NTQ |
| NZ_CBPU010000002_SA168A_K1GA1SV | YKDKPNNTT--SQSGTKNDKQEI------------------SQNNNSNTEVINPPN-NTQ |
| NZ_KB644403_GAM115Ai_K1A1SV | YKDKPNST-L--QSGTKNDKQEI------------------SQNNNSNTEVINPPN-NTQ |
| NZ_KB642330_GAM265BSii_K1GP | YKDKPNST-P--S-------------------------------------QSNPKN-DTQ |
| NZ_KB644501_GAM118Bi_K1GP | YKDKPNNT-P--S-------------------------------------QSNPKN-DTQ |
| NZ_QBRO01000008_B31_K1GA1SV | YKDKPNSTT--SQSGAKNDKQEI------------------SQNNNSNTEVINPPN-NTQ |
| NZ_QEGP01000018_B679_K1GA1SV | YKDKPNNTT--SQSGAKNDKQKI------------------SQNNNSNTEVINPPN-NTQ |
| NZ_AKOZ01000004_HpH-6_K1GA1SV | YKDKPNSTT--SQSGTKNDKQEI------------------SQNNNSNTEVINPPN-NTQ |
| NZ_CBND010000028_SA220A_K1GA1SV | YKDKPNNTT--SQSGAKNDKQEI------------------SQNNNSNTEVINPPN-NTQ |
| NZ_CBPX010000026_SA220C_K1GA1SV | YKDKPNNTT--SQSGAKNDKQEI------------------SQNNNSNTEVINPPN-NTQ |
| NZ_MVXC01000019_HP11042_EP | YESKTKDTPSQNN----------------------------------------PKN-DAQ |
| NZ_MVVK01000024_HP13064_QGA2V | YKNQTNNTP--SQSGAKNDKNESAKNDKQQS--SN-----------SNTEVINPPN-SAQ |
| NZ_QBRJ01000026_B43_K2TA1SV | YKDKPKDKP--SNTTQNNAKNDKQES----------------SQNNSNTQVINPPN-SAQ |
| NZ_MVWN01000014_HP12060_K2TA1SV | YKDKPKDKP--SNTTQNNAKNDKQES----------------SQNNSNTQVINPPN-STQ |
| NZ_QBRM01000044_B37_K2TNSV | YKDKPKDKP--SNTTQNN-----ANNNQQNS-----------AQNNSNTQVINPPN-SAQ |
| NZ_QEGG01000021_MMV242_K2TNSV | YKDKPKDKP--SNTTQNN-----ANNNQQNS-----------AQNNSNTQVINPPN-SAQ |
| NZ_QBPM01000053_38:5_K2TNSV | YKDKPKDKP--SNTTQNN-----ANNNQQNS-----------AQNNSNTQVINPPN-SAQ |
| NZ_QBQW01000092_3774_K2TNSV | YKDKPKDKP--SNTTQNN-----ANNNQQNS-----------AQNNSNTQVINPPN-SAQ |
| RJGR01000002_ZH75_K2TNSV | YKDKPKDKP--SNTTQNN-----ANNNQQNS-----------AQNNSNTQVINPPN-SAQ |
| NZ_AOTW01000001_HpA-11_K2TNSV | YKDKPKDKP--SNTTQNN-----ANNNQQNS-----------AQNNSNTQVINPPN-SAQ |
| NZ_QEGU01000017_B497A_EP | YESKTKDTPSQNN----------------------------------------PKN-DTQ |
| NZ_QEGK01000015_JDX15_EP | YESKTKDNPSQNN----------------------------------------PKN-DTQ |
| NZ_QEGS01000017_B572A_K2TNSV | YKDKPKDKP--SNTTQNN-----ANNNQQNS-----------AQNNSNTQVINPPN-STQ |
| QBQT01000542_GIL237_A2SV | YK-----TPS--QSGAKNDKNESAKNDKQNS-----------AQNNSNTQVINPPN-SAQ |
| NZ_QEGY01000020_B368_K1GA2SV | YKDKPNNTP--SQSGAKNDKNESAKNDKQES-----------SQNNSNTQVINPPN-SAQ |
| NZ_CBKZ010000007_HPARG8G_K1GA2V | YKDKPNHTTS--QSGTKNDKNESAKNDKQQS-----------S--NSNTEVINPPN-NTQ |
| NZ_QBQI01000010_30908_K1GA1SV | YKDKPNNTP--SQSGAKNDKQES-------------------SQNNSNTQVINPPN-SAQ |
| NZ_MBIN01000002_22366_K1GA2V | YKDKPNNTT--SQSGAKNDKNESAKNDKQQS-----------S--NSNTQVINPPN-SAQ |
| NZ_MUOW01000013_CG22087_K1GA2V | YKDKPNNTT--SQSGAKNDKNESAKNDKQQS-----------S--NSNTQVINPPN-SAQ |
| NZ_MBIP01000009_22360_K1GA2V | YKDKPNNTT--SQSGAKNDKNESAKNDKQQS-----------S--NSNTEVINPPN-SAQ |
| NZ_MUPO01000050_CM22360_K1GA2V | YKDKPNNTT--SQSGAKNDKNESAKNDKQQS-----------S--NSNTEVINPPN-SAQ |
| RJHQ01000012_ZH103_QGA2SV | YKNQTNNTP--SQSGAKNDKNESAKNDKQES-----------SQNNSNTQVINPPN-SAQ |
| NZ_QEHF01000019_B297_QGA2SV | YKNQTNNTP--SQSGAKNDKNESAKNDKQES-----------SQNNSNTQVINPPN-SAQ |
| NZ_QEGQ01000017_B661A_QGA2SV | YKNQTNNTP--SQSGAKNDKNESAKNDKQES-----------SQNNSNTQVINPPN-SAQ |
| RJDZ01000020_ZH01_K1GA1SV | YKDKPNNTT--SQSGAKNDKQES-------------------SQNNSNTQVINPPN-SGQ |
| NZ_MILM01000098_MGms2_QGA2SV | YKNQTNNTP--SQSGAKNDKNESAKNDKQES-----------SQNNSNTQVINPPN-STQ |
| NZ_QEGH01000020_JSS185-B120_K1GA2SV | YKDKPNNTP--SQSGTKNDKNESAKNDKQES-----------SQNNSNTQVINPPN-SVQ |
| NZ_QEGX01000020_B400_K1GA2SV | YKDKPNNTP--SQSGAKNDKNESTKNDKQES-----------SQNNSNTQVINPPN-STQ |
| NZ_MVVP01000009_HP13050_EP | YESKTKDNPSQNN----------------------------------------PKN-DTQ |
| NZ_KB636795_GAM101Biv_K1GP | YKDKPNNTPSQNN----------------------------------------PKN-DTQ |
| 747_GAMchjs106B_EP | YESKTKDTPSQNN----------------------------------------PKN-DTQ |
| NZ_KB641695_GAM231Ai_EP | YESKTKDTPSQNN----------------------------------------PKN-DTQ |
| NZ_KB644762_GAMchJs117Ai_EP | YESKTKDTPSQNN----------------------------------------PKN-DTQ |
| NZ_KB635918_HP260BFii_K1GP | YKDKPNNTPSQSN----------------------------------------PKN-DTQ |
| NZ_KB636976_GAM260BSi_K1GP | YKDKPNNTPSQSN----------------------------------------PKN-DTQ |
| NZ_KB636666_GAM244Ai_K1GP | YKDKPNNTPSQNN----------------------------------------PKN-DTQ |
| NZ_KB642078_GAM71Ai_K1GP | YKDKPNNTPSQSN----------------------------------------PKN-DTQ |
| 748_GAMchjs114i_K1GP | YKDKPNNTPSQNN----------------------------------------PKN-DTQ |
| 750_GAMchjs124i_K1GP | YKDKPNNTPSQSN----------------------------------------PKN-DTQ |
| NZ_KB644722_GAMchJs124i_K1GP | YKDKPNNTPSQSN----------------------------------------PKN-DTQ |
| NZ_MVWW01000014_HP12002_EP | YESKTKDNPSQNN----------------------------------------PKN-DAQ |
| HPU29401_Tx30a_EP | YESKTKDNP-QNN----------------------------------------PKN-DAQ |
| NZ_PHML01000027_KH13_EP | YESKTKDTPSQNN----------------------------------------PKN-DTQ |
| NZ_PHMQ01000020_KH8_EP | YESKTKDNPSQNN----------------------------------------PKN-DTQ |
| NZ_MUOX01000054_CG22322_EP | YESKTKDNPSQNN----------------------------------------PKN-DTQ |
| NZ_LFKF01000021_UM209_EP | YESKTKDTPSQNN----------------------------------------PKN-DTQ |
| RJGW01000023_ZH80_EP | YESKTKDTPSQNN----------------------------------------PKN-DTQ |
| RJFS01000012_ZH46_EP | YESKTKDNPSQNN----------------------------------------PKN-DTQ |
| NZ_MBHE01000055_22020_EP | YESKTKDNPSQNN----------------------------------------PKN-DTQ |
| NZ_MUOD01000109_CA22020_EP | YESKTKDNPSQNN----------------------------------------PKN-DTQ |
| NZ_QDJS01000011_B355_EP | YESKTKDTPSQNN----------------------------------------PKN-DTQ |
| NZ_MVWZ01000003_HP11054_EP | YESKTKDNP-QNN----------------------------------------PKN-DAQ |
| NZ_AKOW01000002_HpA-27_EP | YESKTKDNPQND--TQ-----------------------------------NNPKN-DTQ |
| NZ_CCMT01000004_H3016_EP | YESKTKDTPSQNN----------------------------------------PKN-DTQ |
| NZ_AKPC01000004_HpH-11_EP | YESKTKDTPSQNN----------------------------------------PKN-DTQ |
| NZ_MVVJ01000006_HP13068_EP | YESKTKDNPSQNN----------------------------------------PKN-DTQ |
| NZ_MVTH01000002_HP98490_EP | YESKTKDNPSQNN----------------------------------------PKN-DTQ |
| 640_B45_EP | YESKTKDTPSQNN----------------------------------------PKN-DAQ |
| NZ_LIXG01000044_45_EP | YESKTKDNPSQNN----------------------------------------PKN-DTQ |
| NZ_JSUY01000040_1089/03_EP | YESKTKDNPSQNN----------------------------------------PKN-DTQ |
| NZ_JSUZ01000018_1152/04_EP | YESKTKDNPSQNN----------------------------------------PKN-DTQ |
| RJHV01000005_ZH109_EP | YESKTKDNPSQNN----------------------------------------PKN-DAQ |
| NZ_MVYL01000003_HP01234_EP | YESKTKDNPSQNN----------------------------------------PKN-DAQ |
| RJID01000020_ZH118_EP | YESKTKDTPFSNN----------------------------------------PKN-DTQ |
| NZ_KB636762_GAM254Ai_EP | YESKTKDTPSQSN----------------------------------------PKN-DTQ |
| 3615_SSR3_EP | YESKTKDTPSQNN----------------------------------------PKN-DTQ |
| RJEZ01000026_ZH26_EP | YESKTKDTPSQNN----------------------------------------PKN-DAQ |
| RJEV01000011_ZH22_EP | YESKTKDNP-QNN----------------------------------------PKN-DTQ |
| NZ_MVTO01000007_HP16001_EP | YESKTKDNPSQNN----------------------------------------PKN-DTQ |
| RJHW01000032_ZH1110_EP | YESKTKDTPSQNN----------------------------------------PKN-DTQ |
| 3669_SW21A_EP | YESKTKDTPSQNN----------------------------------------PKN-DTQ |
| RJFA01000015_ZH27_EP | YESKTKDTPSQNN----------------------------------------PKN-DTQ |
| NZ_MVTQ01000003_HP15060_EP | YESKTKDNPSQNN----------------------------------------PKN-DTQ |
| RJHL01000004_ZH98_EP | YESKTKDNP-QNN----------------------------------------PKN-DTQ |
| NZ_MVUO01000008_HP15012_EP | YESKTKDTPSQNN----------------------------------------PKN-DTQ |
| RJEU01000017_ZH21_EP | YESKTKDTPSQNN----------------------------------------PKN-DTQ |
| RJFZ01000022_ZH53_EP | YESKTKDTPSQNN----------------------------------------PKN-DTQ |
| NZ_MVWO01000024_HP12059_EP | YESKTKDNP-QNN----------------------------------------PKN-DTQ |
| RJFG01000012_ZH33_EP | YESKTKDNP-QNN----------------------------------------PKN-DTQ |
| RJIS01000004_ZH133_EP | YESKTKDNP-QNN----------------------------------------PKN-DTQ |
| RJIA01000023_ZH115_EP | YESKTKDNP-KDN----------------------------------------PKN-DTQ |
| 3622_SSR12_EP | YESKTKDNP-QNN----------------------------------------PKN-DTQ |
| LC187611_BH27_EP | YESKTKDTP-QNN----------------------------------------PKN-DTQ |
| LC187610_BH24_EP | YESKTKDTP-QNN----------------------------------------PKN-DTQ |
| NZ_LFCA01000009_UM139_EP | YESKTKDTS-QNN----------------------------------------PKN-DTQ |
| 8736_2003_107_EP | YESKTKDTPSQNN----------------------------------------PKN-DTQ |
| NZ_MILA01000026_MG2003-107_EP | YESKTKDTPSQNN----------------------------------------PKN-DTQ |
| AB190976_OK155_K1GA1V | YKDKPNNTN--SQSGAKNDKQDS------------------------NTQVINPPN-SGQ |
| 4461_NP05_EP | YESKTKDTPSQNN----------------------------------------PKN-DAQ |
| NZ_QBQC01000025_456_EP | YESKTKDNP-QNN----------------------------------------PKN-DTQ |
| 3625_SSR17_EP | YESKTKDTPSQNN----------------------------------------PKN-DTQ |
| 3618_SSR7_EP | YESKTKDNPSQNN----------------------------------------PKN-DTQ |
| 3614_SSR2_EP | YESKTKDTPSQNN----------------------------------------PKN-DTQ |
| NZ_MVXE01000017_HP11032_EP | YESKTKDTPSQNN----------------------------------------PKN-DTQ |
| 3616_SSR4_EP | YESKTKDN---------------------------------------------PKN-DTQ |
| NZ_MVSR01000007_HPJ013_EP | YESKTKDNPSQNN----------------------------------------PKN-DTQ |
| 3623_SSR13_EP | YESKTKDTPSQNN----------------------------------------PKN-DTQ |
| NZ_AWNG01000022_X47-2AL_EP | YESKTKDTPSQNN----------------------------------------PKN-DTQ |
| NZ_MVFB01000005_OND1954_EP | YESKTKDTPSQNN----------------------------------------PKN-DTQ |
| NZ_AMOX01000005_R055a_EP | YESKTKDTPSQNN----------------------------------------PKN-DTQ |
| NZ_MVUW01000011_HP14065_EP | YESKTKDNPSQNN----------------------------------------PKN-DTQ |
| AB190980_OK179_EP | YESKTKDNP-QNN----------------------------------------PKN-DTQ |
| AB190984_OK187_EP | YESKTKDNP-QNN----------------------------------------PKN-DTQ |
| NZ_CP006824_oki422_EP | YESKNKDNP-QNN----------------------------------------PKN-DTQ |
| AB190983_OK185_EP | YESKNKDNP-QNN----------------------------------------PKN-DTQ |
| AB190987_OK205_EP | YESKNKDNP-QNN----------------------------------------PKN-DTQ |
| AB190968_OK107_EP | YESKNKDNP-QNN----------------------------------------PKN-DTQ |
| AB190975_OK144_EP | YESKNKDNP-QNN----------------------------------------PKN-DTQ |
| NZ_CP006827_oki898_EP | YESKNKDNP-QNN----------------------------------------PKN-DTQ |
| NZ_CP006821_oki112_EP | YESKNKDNP-QNN----------------------------------------PKN-DTQ |
| AB190979_OK160_EP | YESKNKDNP-QNN----------------------------------------PKN-DTQ |
| AB190974_OK139_EP | YESKNKDNP-QNN----------------------------------------PKN-DTQ |
| NZ_CP006820_oki102_EP | YESKNKDNP-QNN----------------------------------------PKN-DTQ |
| LC187608_BH70_EP | YESKTKDTPSQNN----------------------------------------PKN-DTQ |
| RJGS01000021_ZH76_EP | YESKTKDNPSQNN----------------------------------------PKN-DTQ |
| RJEO01000027_ZH15_EP | YESKTKDTPSQNN----------------------------------------PKN-DTQ |
| NZ_MVSV01000014_HP99689_EP | YESKTKDTP-QNN----------------------------------------PKN-DAQ |
| RJHF01000031_ZH91_EP | YESKTKDTPSQNN----------------------------------------PKN-DTQ |
| NZ_MVXF01000021_HP11020_EP | YESKTKDTPSQNN----------------------------------------PKN-DTQ |
| RJGZ01000004_ZH84_EP | YESKTKDNPSQNN----------------------------------------PKN-DTQ |
| NC_017372_India7_EP | YESKTKDTPSQNN----------------------------------------PKN-ETQ |
| NZ_PHLK01000016_KH41_EP | YESKTKDNPSQNN----------------------------------------PKN-DTQ |
| RJGF01000005_ZH59_EP | YESKTKDTPSQNN----------------------------------------PKN-DTQ |
| NZ_PHMD01000012_KH21_EP | YESKTKDNPSQNN----------------------------------------PKN-DAQ |
| RJGA01000006_ZH54_EP | YESKTKDTPSQNN----------------------------------------PKN-DTQ |
| NZ_QBPV01000035_57_single_EP | YESKTKDTPQNS-----------------------------------------PKN-DAQ |
| NZ_AMYU01000001_A45_EP | YESKTKDNPKDN-----------------------------------------PKN-DTQ |
| NZ_CP006822_oki128_EP | YESKTKDNP-QNN----------------------------------------PKN-DTQ |
| NZ_CP006823_oki154_EP | YESKTKDNP-QNN-PQNN-------------------------------PKNNPKN-DTQ |
| NZ_CP006825_oki673_EP | YESKTKDNP-QNN-PQN-----------------------------------NPKN-DTQ |
| NZ_MVWV01000018_HP12014_EP | YKGKNKDTS-QGG----------------------------------------TKN-DTQ |
| NZ_MVXS01000019_HP07019_EP | YKGKNKDTS-QGG----------------------------------------TKN-DTQ |
| NZ_CBNA010000013_SA302C_EP | YKGKNKDTS-QGG----------------------------------------TKN-DTQ |
| NZ_CBOF010000014_SA302A_EP | YKGKNKDTS-QGG----------------------------------------TKN-DTQ |
| NZ_CBOY010000008_SA172A_EP | YKGKNKDTS-QGG----------------------------------------TKN-DTQ |
| NZ_MVXA01000018_HP11049_EP | YESKTKDNPSQNN----------------------------------------PKN-DAQ |
| NZ_MVXL01000002_HP08074_EP | YESKTKDNPSQNN----------------------------------------PKN-DAQ |
| NZ_MBGO01000017_3053_EP | YESKTKDTPSQNN----------------------------------------PKN-DAQ |
| NZ_KB641893_GAM201Ai_K1GP | YKDKPNNTPSQSN----------------------------------------PKN-DTQ |
| NZ_KB642543_GAM264Ai_K1GP | YKDKPNNTPSQSN----------------------------------------PKN-DTQ |
| NZ_KB636348_GAM239Bi_EP | YESKTKDTPSQNN----------------------------------------PKN-DTQ |
| NZ_KB641928_GAM83T_EP | YESKTKDTLSQNN----------------------------------------PKN-DTQ |
| NZ_MVUC01000028_HP15033_EP | YESKTKDKPSSNN----------------------------------------PKN-DAQ |
| RJHR01000032_ZH104_EP | YESKTKDTPSQNN----------------------------------------PKN-DAQ |
| NZ_AKPR01000002_HpP-23_EP | YESKTKDNP-QNN----------------------------------------PKN-DAQ |
| NZ_AKOH01000005_HpH-27_EP | YESKTKDNP-QNN----------------------------------------PKN-DAQ |
| LC187601_BH78_EP | YESKTKDTP-QNN----------------------------------------PKN-DTQ |
| LC187602_BH77_EP | YESKTKDTP-QNN----------------------------------------PKN-DTQ |
| RJEG01000002_ZH08_EP | YESKTKDTPSQNN----------------------------------------PKN-DTQ |
| RJER01000002_ZH18_EP | YESKTKDTPSQNN----------------------------------------PKN-DTQ |
| NZ_QDJV01000010_AAP164_EP | YESKTKDNPSQNN----------------------------------------PKN-DTQ |
| NZ_CBQD010000013_SA165A_EP | YESKTKDNPSQNN----------------------------------------PKN-DAQ |
| NZ_CBQJ010000015_SA165C_EP | YESKTKDNPSQNN----------------------------------------PKN-DAQ |
| NZ_CBQK010000019_SA164A_EP | YESKTKDNPSQNN----------------------------------------PKN-DAQ |
| NZ_CBQM010000015_SA164C_EP | YESKTKDNPSQNN----------------------------------------PKN-DAQ |
| NZ_AOTV01000004_UMB_G1_EP | YESKTKDNPSQNN----------------------------------------PKN-DTQ |
| NZ_QEGN01000010_BMG112_EP | YESKTKDTPSQNN----------------------------------------PKN-DAQ |
| LC187605_BH1101_EP | YESKTKDNPSQNN----------------------------------------PKN-DAQ |
| RJFC01000026_ZH29_EP | YESKTKDNPSQNN----------------------------------------PKN-DTQ |
| NZ_MVVH01000003_HP14021_EP | YESKTKDNPSQNN----------------------------------------PKN-DTQ |
| NZ_MVTE01000006_HP99255_EP | YESKTKDTPSQNN----------------------------------------PKN-DTQ |
| NZ_MVWR01000002_HP12038_EP | YESKTKDTPSQNN----------------------------------------PKN-DTQ |
| NZ_CP007604_BM013A_EP | YESKTKDTPSQNN----------------------------------------PKN-DTQ |
| RJIL01000023_ZH126_EP | YESKTKDTPSQNN----------------------------------------PKN-DAQ |
| NZ_QBRH01000015_B47_EP | YESKTKDTPSQNN----------------------------------------PKN-DTQ |
| NZ_LIXF01000002_22_EP | YESKTKDTPSQNN----------------------------------------PKN-DTQ |
| 3662_3697_EP | YESKTKDTPSQNN----------------------------------------PKN-DAQ |
| NZ_PHMM01000006_KH12_EP | YESKTKDTPSQNN----------------------------------------PKN-DTQ |
| NZ_MVVW01000010_HP13025_EP | YESKTKDTPSQNN----------------------------------------PKN-DTQ |
| NZ_PHMU01000023_KH3_EP | YESKTKDNP-QNN----------------------------------------PKN-DTQ |
| NZ_PHMV01000021_KH2_EP | YESKTKNNP-QNN----------------------------------------PKN-DTQ |
| NZ_MVVS01000011_HP13029_EP | YESKTKDNPSQNN----------------------------------------PKN-DTQ |
| NC_012973_B38_EP | YESKTKDTP-QNN----------------------------------------PKN-DAQ |
| RJFX01000001_ZH51_EP | YESKTKDTP-QNN----------------------------------------PKN-DTQ |
| NZ_MBGM01000006_3076_K1GP | YKDKPNNTPSQNN----------------------------------------PKN-DTQ |
| NZ_MVWH01000008_HP12077_EP | YESKTKDNHSQNN----------------------------------------PKN-DTQ |
| NZ_AMOV01000005_R038b_EP | YESKTKDTPSQNN----------------------------------------PKN-DTQ |
| NZ_CP012905_7C_EP | YESKTKDTPSQNN----------------------------------------PKN-DAQ |
| RJHH01000018_ZH94_EP | YESKTKDTPSQNN----------------------------------------PKN-DTQ |
| NZ_CBNG010000014_SA173C_EP | YESKTKDTPSQNN----------------------------------------PKN-ETQ |
| NZ_CBOU010000013_SA173A_EP | YESKTKDTPSQNN----------------------------------------PKN-ETQ |
| NZ_AKQJ01000003_HpP-15b_EP | YESKTKDNP-QNN----------------------------------------PKN-DAQ |
| NZ_QBQF01000040_36166_EP | YESKTKDTPSQNN----------------------------------------PKN-DAQ |
| NZ_AZBQ01000032_Iso6_EP | YESKTKDTPSQNN----------------------------------------PKN-DAQ |
| NZ_AZBR01000003_PMSS1_EP | YESKTKDTPSQNN----------------------------------------PKN-DAQ |
| NZ_AZBS01000027_Iso7_EP | YESKTKDTPSQNN----------------------------------------PKN-DAQ |
| NZ_QEHB01000016_B360_EP | YESKTKDNPSQNN----------------------------------------PKN-DVQ |
| NZ_QEGI01000016_JMM43_EP | YESKTKDNPSQNN----------------------------------------PKN-DAQ |
| NZ_JSXB01000030_655/99_EP | YESKTKDNPSQNN----------------------------------------PKN-DAQ |
| NZ_QBRU01000068_B23+S27R2:R48_EP | YESKTKDTPSQNN----------------------------------------SKN-DTQ |
| RJIP01000014_ZH130_EP | YESKTKDTPQNN-----------------------------------------PKN-DTQ |
| NC_022130_SouthAfrica20_EP | YESKTKDTPSQNN----------------------------------------PKN-ETQ |
| NZ_MVVN01000024_HP13056_EP | YESKTKDNPSQNN----------------------------------------PKN-DAQ |
| NZ_MVWE01000004_HP13007_EP | YESKTKDTHSQNN----------------------------------------PKN-DTQ |
| NZ_MVYA01000012_HP04057_EP | YESKTKDTP-QNN----------------------------------------PKN-DTQ |
| RJEI01000018_ZH10_EP | YESKTKDTPSQNN----------------------------------------PKN-DTQ |
| NZ_MVXX01000010_HP05044_EP | YESKTKDTPSQNN----------------------------------------PKN-DTQ |
| RJHZ01000014_ZH113_EP | YESKTKDNPSQNN----------------------------------------PKN-DTQ |
| RJES01000016_ZH19_EP | YESKTKDTPSQNN----------------------------------------PKN-DTQ |
| RJHA01000017_ZH85_EP | YESKTKDTPSQNN----------------------------------------PKN-DTQ |
| RJIR01000021_ZH132_EP | YESKTKDTPSQNN----------------------------------------PKN-DTQ |
| RJIV01000003_ZH136_EP | YESKTKDTPSQNN----------------------------------------PKN-DAQ |
| NZ_PHLL01000021_KH40_EP | YESKTKDNP-QNN----------------------------------------PKN-DTQ |
| RJGY01000002_ZH83_EP | YESKTKDTPSQNN----------------------------------------PKN-DTQ |
| RJGL01000014_ZH66_EP | YESKTKDNPSQNN----------------------------------------PKN-DTQ |
| NZ_AKOV01000001_HpA-26_EP | YESKTKDTPSQNN----------------------------------------PKN-DAQ |
| 4542_Nic09_A_EP | YESKTKDNPSQNN----------------------------------------PKN-DAQ |
| 4553_Nic14_C_EP | YESKTKDNPSQNN----------------------------------------PKN-DAQ |
| NZ_QDJD01000010_CRL122_EP | YESKTKDTP-QNS----------------------------------------PKN-DAQ |
| NZ_MBIF01000020_22386_EP | YESKTKDTPSQNN----------------------------------------PKN-DAQ |
| RJHS01000003_ZH106_EP | YESKTKDNPSQNN----------------------------------------PKN-DAQ |
| NZ_QDJK01000010_B529A_EP | YESKTKDNP-QNN----------------------------------------PKN-DAQ |
| 8747_22322_EP | YESKTKDNPSQNN----------------------------------------PKN-DTQ |
| NZ_JSXU01000006_207/99_EP | YESKTKDTPSQNN----------------------------------------PKN-DAQ |
| LC187606_BH80_EP | YESKTKDTPSQNN----------------------------------------PKN-DAQ |
| NZ_AKOO01000004_HpH-43_EP | YESKTKDNPSQNN----------------------------------------PKN-DAQ |
| NZ_QBQL01000042_565-99_EP | YESKIKDTP-QNN----------------------------------------PKN-DTQ |
| RJHP01000001_ZH102_EP | YESKTKDTPSQNN----------------------------------------PKN-DTQ |
| NZ_QBRP01000003_B30_EP | YESKTKDNPSQNN----------------------------------------PKN-DAQ |
| LC187597_BH45_EP | YESKTKDNPSQNN----------------------------------------PKN-DTQ |
| RJHO01000014_ZH101_EP | YESKTKDTP-QNN----------------------------------------PKN-DAQ |
| RJFD01000005_ZH30_EP | YENKTKDNPSQNN----------------------------------------PKN-DAQ |
| RJFF01000001_ZH32_EP | YENKTKDNPSQNN----------------------------------------PKN-DAQ |
| RJGO01000012_ZH69_EP | YENKTKDNPSQNN----------------------------------------PKN-DAQ |
| LC187607_BH76_EP | YESKTKDTPSQNN----------------------------------------PKN-DAQ |
| NZ_MVVD01000013_HP14039_EP | YESKTKDTPSQNN----------------------------------------PKN-DAQ |
| NZ_QBQV01000033_3843_EP | YESKTKDNPSQNN----------------------------------------PKN-DAQ |
| 4554_Nic15_A_EP | YESKTKDNPSQNN----------------------------------------PKN-DAQ |
| NZ_MBHI01000027_3136_EP | YESKTKDTPSQNN----------------------------------------PKN-DAQ |

Supplementary Table 1: The high polymorphism in the “d-region” after BioEdit alignment of the 1259 strains included in the study.
