# Supplementary material for: New genotypes of Helicobacter Pylori VacA d-region identified from global strains
Source: BMC Mol Cell Biol. 2021 Jan 7;22:4. doi: 10.1186/s12860-020-00338-2 (PMC7791883; doi:10.1186/s12860-020-00338-2)
Supplement: Supplementary file 2 — Additional file 2. [file 12860_2020_338_MOESM2_ESM.docx]

| **Locus** | **hp_type** | **CA/NCA*** | **Disease** | **Continent** | **Country** | **W_EAST**** | **CagA EPIYA** | **VacA KQE_type** | **VacA Subtype** | **VacA simcn** | **Pubmed ID** |
| --- | --- | --- | --- | --- | --- | --- | --- | --- | --- | --- | --- |
| LC185387 | 00-255 | CA | CA | Asia | Japan | E | ABD | K | K1GA2V | s1i1m1c1n1 | 27833662 |
| LC185388 | 00-37 | CA | CA | Asia | Japan | E | ABD | K | K1GA2V | s1i1m1c1n1 | 27833662 |
| LC185391 | 01-23 | NCA | CG | Asia | Japan | E | ABD | K | K1GA2V | s1i1m1c1n1 | 27833662 |
| LC185392 | 01-301 | NCA | CG | Asia | Japan | E |  | K | K1GA2V | s1i1m1c1n1 | 27833662 |
| LC185393 | 01-365 | NCA | CG | Asia | Japan | E |  | K | K1GA2SV | s1i1m1c1n1 | 27833662 |
| LC185394 | 01-375 | NCA | CG | Asia | Japan | E | ABD | K | K1GA2SV | s1i1m1c1n1 | 27833662 |
| LC185395 | 01-381 | NCA | CG | Asia | Japan | E | ABD | K | K1GA2V | s1i1m1c1n1 | 27833662 |
| LC185396 | 01-383 | NCA | CG | Asia | Japan | E | ABD | K | K1GA2SV | s1i1m1c1n1 | 27833662 |
| LC185397 | 01-487 | NCA | CG | Asia | Japan | E | ABD | K | K1GA2SV | s1i1m1c1n1 | 27833662 |
| LC185398 | 01-540 | NCA | CG | Asia | Japan | E | ABD | K | K1GA2SV | s1i1m1c1n1 | 27833662 |
| LC185399 | 01-543 | CA | MALT | Asia | Japan | E | ABD | K | K1GA2V | s1i1m1c1n1 | 27833662 |
| LC185400 | 01-617 | NCA | CG | Asia | Japan | E | ABD | K | K1GA2V | s1i1m1c1n1 | 27833662 |
| LC185401 | 01-633 | NCA | CG | Asia | Japan | E | ABD | K | K1GA2SV | s1i1m1c1n1 | 27833662 |
| LC185402 | 01-643 | NCA | CG | Asia | Japan | E | ABD | K | K1GA2V | s1i1m1c1n1 | 27833662 |
| LC185403 | 02-331 | NCA | CG | Asia | Japan | E | ABD | K | K1GA2V | s1i1m1c1n1 | 27833662 |
| LC185404 | 02-425 | NCA | CG | Asia | Japan | E | ABD | K | K1GA2V | s1i1m1c1n1 | 27833662 |
| LC185405 | 02-729 | CA | CA | Asia | Japan | E |  | K | K1GA2SV | s1i1m1c1n1 | 27833662 |
| LC185406 | 02-767 | CA | MALT | Asia | Japan | E |  | K | K1GA2V | s1i1m1c1n1 | 27833662 |
| LC185407 | 03-132 | NCA | CG | Asia | Japan | E | ABD | K | K1GA2SV | s1i1m1c1n1 | 27833662 |
| LC185408 | 03-2 | NCA | CG | Asia | Japan | E |  | K | K1GA2V | s1i1m1c1n1 | 27833662 |
| LC185410 | 04-140 | CA | CA | Asia | Japan | E | ABD | K | K1GA2V | s1i1m1c1n1 | 27833662 |
| LC185411 | 04-303 | CA | MALT | Asia | Japan | E | ABD | K | K1GA2V | s1i1m1c1n1 | 27833662 |
| LC185412 | 05-423 | CA | CA | Asia | Japan | E |  | K | K1GA2V | s1i1m1c1n1 | 27833662 |
| LC185413 | 07-223 | CA | CA | Asia | Japan | E | ABD | K | K1GA2SV | s1i1m1c1n1 | 27833662 |
| LC185414 | 09-294 | CA | MALT | Asia | Japan | E | ABD | K | K1GA2V | s1i1m1c1n1 | 27833662 |
| LC185415 | 10-252 | CA | CA | Asia | Japan | E |  | K | K1GA3SV | s1i1m1c1n1 | 27833662 |
| LC185416 | 10-354 | CA | CA | Asia | Japan | E | ABD | K | K1GA2V | s1i1m1c1n1 | 27833662 |
| LC185417 | 10-358 | CA | CA | Asia | Japan | E | ABD | K | K1GA2V | s1i1m1c1n1 | 27833662 |
| LC185418 | 10-416 | CA | CA | Asia | Japan | E |  | K | K1GA2V | s1i1m1c1n1 | 27833662 |
| LC185419 | 10-442 | CA | MALT | Asia | Japan | E | ABD | K | K1GA2V | s1i1m1c1n1 | 27833662 |
| LC185420 | 10-447 | CA | CA | Asia | Japan | E | ABD | K | K1GA2V | s1i1m1c1n1 | 27833662 |
| LC185421 | 10-453 | CA | CA | Asia | Japan | E | ABD | K | K1GA2V | s1i1m1c1n1 | 27833662 |
| LC185422 | 10-456 | CA | MALT | Asia | Japan | E | ABD | K | K1GA2SV | s1i1m1c1n1 | 27833662 |
| NZ_RPFS01000014 | 1002 | CA | CA | America | Colombia | W | ABCC | K | K2TNSV | s1i1m1c1n1 |  |
| NZ_RPFT01000013 | 1057 | CA | CA | America | Colombia | W | ABCC | Q | QGA2SV | s1i1m1c1n1 | 28053669 |
| NZ_MBJT01000024 | 1061 | CA | CA | America | Colombia | W | ABCC | K | K1GA1SV | s1i1m1c1n1 | 28912838 |
| NZ_RPFR01000012 | 1071 | CA | CA | America | Colombia | W | ABCC | K | K2TNSV | s1i1m1c1n1 |  |
| NZ_MBKC01000032 | 1077 | CA | CA | America | Colombia | W | ABCC | K | K2TNSV | s1i1m1c2n2 | 28912838 |
| NZ_MBJP01000076 | 1081 | CA | CA | America | Colombia | W | ABC | K | K1GA1SV | s1i1m1c1n1 | 28912838 |
| NZ_MBKD01000014 | 1086 | CA | CA | America | Colombia | W | ABC | Q | QGA2SV | s1i1m1c1n1 | 28912838 |
| NZ_RPFU01000002 | 1088 | CA | CA | America | Colombia | W | ABC | K | K2TNSV | s1i1m1c1n1 | 28053669 |
| NZ_JSUY01000040 | 1089/03 | NCA | DU | America | Portugal | W | ABC | E | EP | s1i2m2c2n2 | 25657274 |
| LC185423 | 11-9 | CA | CA | Asia | Japan | E |  | K | K1GA2V | s1i1m1c1n1 | 27833662 |
| NZ_MBJK01000002 | 1102 | CA | CA | America | Colombia | W | ABCC | K | K2TNSV | s1i1m1c1n1 | 28912838 |
| NZ_JSUZ01000018 | 1152/04 | NCA | DU | America | Portugal | W | ABC | E | EP | s1i2m2c2n2 | 25657274 |
| NZ_MJMU01000010 | 1177 | CA | CA | Asia | Singapore | W | ABD | K | K1GA2V | s1i1m2c2n2 |  |
| NZ_JSXT01000040 | 1198/04 | NCA | DU | America | Portugal | W | ABCC | Q | QGA2SV | s1i1m1c1n1 | 25657274 |
| LC185424 | 13-330 | CA | MALT | Asia | Japan | E | ABD | K | K1GA2SV | s1i1m1c1n1 | 27833662 |
| NZ_MJGG01000003 | 132 | CA | CA | Asia | Singapore | W | ABC | K | K1GA2SV | s1i1m2c2n2 | 27583131 |
| NZ_MJMX01000001 | 132A | CA | CA | Asia | Singapore | W | ABC | K | K1GA2SV | s1i1m2c2n2 |  |
| LC185425 | 14-200 | CA | MALT | Asia | Japan | E | ABD | K | K1GA2V | s1i1m1c1n1 | 27833662 |
| NZ_JSXX01000062 | 173/00 | NCA | PU | America | Portugal | W |  | E | EP | s2i2m2c2n2 | 25657274 |
| NZ_MJGH01000008 | 178 | CA | CA | Asia | Singapore | W | ABD | K | K1GA2V | s1i1m1c1n1 | 27583131 |
| NZ_MJMT01000013 | 178A | CA | CA | Asia | Singapore | W | ABD | K | K1GA2V | s1i1m1c1n1 | 17057244 |
| NZ_JSXV01000026 | 1846/05 | NCA | DU | America | Portugal | W | ABCC | K | K2TNSV | s1i1m1c1n1 | 25657274 |
| NZ_QBPS01000058 | 18:2 | NCA | VOL | Europe | Sweden | W | ABC | K | K2TNSV | s1i1m1c1n1 | 19338650 |
| HQ287753 | 191.9 |  |  | Africa | Africa | W |  | K | K1GA2V | s1i1m1c1n1 | 20870762 |
| 8732 | 2003_103 |  |  |  |  |  |  | K | K1GA1SV | s1i1m1c1n1 |  |
| 8736 | 2003_107 |  |  |  |  |  |  | E | EP | s1i2m2c2n2 |  |
| 8718 | 2003_84 |  |  |  |  |  |  | K | K1GA2SV | s1i1m1c1n1 |  |
| 8749 | 2004_2 |  |  |  |  |  |  | K | K1GA2SV | s1i1m1c1n1 |  |
| 8692 | 2004_20 |  |  |  |  |  |  | K | K1GA1SV | s1i1m1c1n1 |  |
| 8731 | 2005_126 |  |  |  |  |  |  | K | K1GA2SV | s1i1m1c1n1 |  |
| 8695 | 2005_72 |  |  |  |  |  |  | Q | QGA2SV | s1i1m1c1n1 |  |
| 8693 | 2005_98 |  |  |  |  |  |  | K | K2TNSV | s1i1m1c1n1 |  |
| NZ_MBJL01000248 | 2006 | NCA | IM | America | Colombia | W | ABABC | K | K1GA2V | s1i1m1c1n1 | 28912838 |
| 8701 | 2006_103 |  |  |  |  |  |  | K | K2TNSV | s1i1m1c1n1 |  |
| 8700 | 2006_407 |  |  |  |  |  |  | K | K2TNSV | s1i1m1c1n1 |  |
| 8721 | 2006_479 |  |  |  |  |  |  | K | K1GA1SV | s1i1m1c1n1 |  |
| 8737 | 2006_480 |  |  |  |  |  |  | K | K1GA2V | s1i1m1c1n1 |  |
| 8698 | 2006_52 |  |  |  |  |  |  | K | K2TNSV | s1i1m1c1n1 |  |
| NZ_MBJM01000006 | 2007 | NCA | IM | America | Colombia | W | ABC | K | K1GA2V | s1i1m1c1n1 | 28912838 |
| NZ_MBJN01000004 | 2010 | NCA | IM | America | Colombia | W | ABC | K | K1GA2V | s1i1m1c1n1 | 28912838 |
| 8745 | 2011_145 |  |  |  |  |  |  | K | K2TNSV | s1i1m1c1n1 |  |
| 8771 | 2011_41 |  |  |  |  |  |  | K | K1GA2SV | s1i1m1c1n1 |  |
| 8681 | 2012_26 |  |  |  |  |  |  | K | K2TA1SV | s1i1m1c1n1 |  |
| NC_017374 | 2017 | NCA | DU | Europe | France | W | ABC | K | K1GA1SV | s1i1m1c1n1 | 21515762 |
| NC_017381 | 2018 | NCA | DU | Europe | France | W | ABC | K | K1GA1SV | s1i1m1c1n1 | 21515762 |
| NZ_MBJI01000007 | 2025 | NCA | CG | America | Colombia | W | ABC | K | K2TNSV | s1i1m1c1n1 | 28912838 |
| NZ_MBJJ01000007 | 2027 | NCA | IM | America | Colombia | W | ABC | K | K2TNSV | s1i1m1c1n1 | 28912838 |
| NZ_MBKG01000018 | 2029 | NCA | IM | America | Colombia | W | ABCC | K | K2TNSV | s1i1m1c1n1 |  |
| NZ_MBJB01000046 | 2036 | NCA | AG | America | Colombia | W | ABC | K | K2TNSV | s1i1m1c1n1 | 28912838 |
| NZ_MBJC01000012 | 2040 | NCA | AG | America | Colombia | W | ABC | K | K2TNSV | s1i1m1c1n1 | 28912838 |
| NZ_MBJD01000006 | 2047 | NCA | IM | America | Colombia | W | ABC | K | K1GA2SV | s1i1m1c1n1 | 28912838 |
| NZ_MBJE01000006 | 2061 | NCA | AG | America | Colombia | W | ABCC | K | K2TNSV | s1i1m1c1n1 | 28912838 |
| NZ_MBJF01000007 | 2065 | NCA | CG | America | Colombia | W | ABCC | K | K1GA2SV | s1i1m1c1n1 | 28912838 |
| NZ_JSXU01000006 | 207/99 | NCA | PU | America | Portugal | W |  | E | EP | s2i2m2c2n1 | 25657274 |
| NZ_LIXF01000002 | 22 |  |  | Asia | Kuwait | W | AB | E | EP | s2i2m2c2n2 |  |
| NZ_MBHF01000009 | 22019 | NCA | AG | America | Colombia | W | ABC | K | K1GA1SV | s1i1m1c1n1 | 28912838 |
| 8756 | 22019_ve |  |  |  |  |  |  | K | K1GA2V | s1i1m1c1n1 |  |
| NZ_MBHE01000055 | 22020 | NCA | IM | America | Colombia | W |  | E | EP | s2i2m2c2n2 | 28912838 |
| NZ_MBHD01000006 | 22021 | NCA | CG | America | Colombia | W | ABC | Q | QGA2SV | s1i1m1c1n1 | 28912838 |
| 8711 | 22023 | NCA | CG | America | Colombia | W |  | K | K2TNSV | s1i1m1c1n1 | 28912838 |
| NZ_MBHB01000011 | 22025 | NCA | IM | America | Colombia | W |  | E | EP | s2i2m2c2n2 | 28912838 |
| NZ_MBKB01000048 | 22046 | NCA | IM | America | Colombia | W | ABC | K | K2TNSV | s1i1m1c1n1 | 28912838 |
| 8720 | 22046_ve |  |  |  |  |  |  | K | K2TNSV | s1i1m1c1n1 |  |
| NZ_MBHA01000006 | 22087 | NCA | CG | America | Colombia | W | ABC | K | K1GA2V | s1i1m1c1n1 | 28912838 |
| 8764 | 22093 | CA | CA | America | Colombia | W | AC | E | EP | s2i2m2c2n2 | 28912838 |
| NZ_MBGY01000008 | 22095 | NCA | AG | America | Colombia | W | ABC | Q | QGA2SV | s1i1m1c1n1 | 28912838 |
| NZ_MBGX01000004 | 22151 | NCA | AG | America | Colombia | W | ABC | Q | QGA2SV | s1i1m1c1n1 | 28912838 |
| NZ_MBGV01000030 | 22308 | NCA | AG | America | Colombia | W | ABCC | K | K1GA2V | s1i1m1c1n1 | 28912838 |
| NZ_MBGU01000058 | 22311 | NCA | AG | America | Colombia | W | ABCC | Q | QGA2SV | s1i1m1c1n1 | 28912838 |
| NZ_MBGT01000006 | 22312 | NCA | IM | America | Colombia | W |  | K | K1GA1SV | s1i1m1c1n1 | 28912838 |
| NZ_MBGS01000006 | 22315 | NCA | AG | America | Colombia | W | ABC | Q | QGA1SV | s1i1m1c1n1 | 28912838 |
| 8760 | 22315_ve |  |  |  |  |  |  | K | K1GA2V | s1i1m1c1n1 |  |
| NZ_MBGQ01000008 | 22317 | NCA | CG | America | Colombia | W | ABCC | K | K1GA1SV | s1i1m1c1n1 | 28912838 |
| 8747 | 22322 | NCA | CG | America | Colombia | W | ABC | E | EP | s2i2m2c2n2 | 28912838 |
| NZ_MBHP01000007 | 22331 | NCA | IM | America | Colombia | W | ACC | K | K2TNSV | s1i1m1c1n1 | 28912838 |
| NZ_MBHO01000001 | 22335 | NCA | IM | America | Colombia | W | ABC | Q | QGA2SV | s1i1m1c1n1 | 28912838 |
| NZ_MBHN01000027 | 22336 | NCA | AG | America | Colombia | W | ABC | Q | QGA2SV | s1i1m1c1n1 | 28912838 |
| NZ_MBIV01000002 | 22339 | NCA | IM | America | Colombia | W |  | E | EP | s2i2m2c2n2 | 28912838 |
| NZ_MBIU01000007 | 22341 | NCA | IM | America | Colombia | W | ABCBC | K | K1GA2V | s1i1m1c1n1 | 28912838 |
| 8709 | 22341_ve |  |  |  |  |  |  | Q | QGA2SV | s1i1m1c1n1 |  |
| NZ_MBIT01000036 | 22343 | NCA | IM | America | Colombia | W | ABC | Q | QGA2SV | s1i1m1c1n1 | 28912838 |
| NZ_RPFQ01000017 | 22345 | NCA | IM | America | Colombia | W | ABC | Q | QGA1SV | s1i1m1c1n1 |  |
| NZ_MBIS01000002 | 22346 | NCA | IM | America | Colombia | W | ABC | Q | QGA2SV | s1i1m1c1n1 | 28912838 |
| NZ_MBJZ01000010 | 22347 | NCA | IM | America | Colombia | W | ABC | K | K2TNSV | s1i1m1c1n1 | 28912838 |
| NZ_MBJY01000038 | 22352 | NCA | IM | America | Colombia | W | ABC | Q | QGA2SV | s1i1m1c2n2 | 28912838 |
| NZ_MBIP01000009 | 22360 | NCA | AG | America | Colombia | W | ABC | K | K1GA2V | s1i1m2c2n2 | 28912838 |
| NZ_MBIO01000012 | 22362 | NCA | AG | America | Colombia | W | ABC | K | K1GA2SV | s1i1m1c1n1 | 28912838 |
| NZ_MBIN01000002 | 22366 | NCA | CG | America | Colombia | W | ABC | K | K1GA2V | s1i1m2c2n2 | 28912838 |
| 8726 | 22366_ve |  |  |  |  |  |  | Q | QGA2SV | s1i1m1c1n1 |  |
| NZ_MBIM01000006 | 22367 | NCA | CG | America | Colombia | W | ABC | Q | QGA2SV | s1i1m1c1n1 | 28912838 |
| 8734 | 22367_ve |  |  |  |  |  |  | Q | QGA2SV | s1i1m1c1n1 |  |
| NZ_MBIL01000022 | 22368 | NCA | IM | America | Colombia | W | ABBC | Q | QGA2SV | s1i1m1c1n1 | 28912838 |
| NZ_MBIK01000211 | 22370 | NCA | CG | America | Colombia | W | ABC | Q | QGA2SV | s1i1m1c1n1 | 28912838 |
| NZ_MBIJ01000100 | 22371 | NCA | CG | America | Colombia | W | ABC | K | K1GA1SV | s1i1m1c1n1 | 28912838 |
| NZ_MBII01000020 | 22377 | NCA | CG | America | Colombia | W | ABC | K | K2TNSV | s1i1m1c1n1 | 28912838 |
| NZ_MBIH01000019 | 22378 | NCA | CG | America | Colombia | W | ABCC | K | K1GA2V | s1i1m1c1n1 | 28912838 |
| 8776 | 22384 | NCA | CG | America | Colombia | W |  | E | EP | s2i1m2c2n2 |  |
| NZ_MBIG01000006 | 22385 | NCA | CG | America | Colombia | W | ABC | K | K1GA2V | s1i1m1c1n1 | 28912838 |
| NZ_MBIF01000020 | 22386 | NCA | IM | America | Colombia | W |  | E | EP | s2i2m2c2n2 | 28912838 |
| 8773 | 22388 | NCA | IM | America | Colombia | W | ABC | Q | QGA2SV | s1i1m1c1n1 |  |
| NZ_MBJX01000026 | 22389 | NCA | DU | America | Colombia | W |  | E | EP | s2i2m2c2n2 | 28912838 |
| NZ_MBIE01000005 | 22390 | NCA | DU | America | Colombia | W | ABC | K | K1GA2V | s1i1m1c1n1 | 28912838 |
| NZ_MBID01000109 | 22393 | NCA | AG | America | Colombia | W | ABC | K | K2TNSV | s1i1m1c1n1 | 28912838 |
| NZ_MBIC01000139 | 22395 | NCA | AG | America | Colombia | W |  | E | EP | s2i2m2c2n2 | 28912838 |
| NZ_MBIB01000047 | 22402 | CA | CA | America | Colombia | W | ABC | K | K1GA2V | s1i1m1c1n1 | 28912838 |
| NZ_JSXY01000029 | 228/99 | NCA | PU | America | Portugal | W |  | E | EP | s2i2m2c2n2 | 25657274 |
| NZ_QBPR01000050 | 23:2_single | NCA | VOL | Europe | Sweden | W | ABC | K | K2TNSV | s1i1m2c2n2 |  |
| NZ_MBIA01000025 | 24004 | NCA | CG | America | Colombia | W | ABCC | K | K2TA1SV | s1i1m1c1n1 | 28912838 |
| NZ_MBJW01000023 | 24012 | NCA | CG | America | Colombia | W | ABC | K | K1GA2SV | s1i1m1c1n1 | 28912838 |
| NZ_MJIS01000006 | 241 | CA | CA | Asia | Singapore | W | ABBD | K | K1GA2V | s1i1m2c2n2 |  |
| NZ_MBHY01000007 | 26024 | NCA | IM | America | Colombia | W | ABCC | Q | QGA2SV | s1i1m1c1n1 | 28912838 |
| NZ_MBJV01000039 | 26083 | CA | CA | America | Colombia | W | ABCC | K | K2TNSV | s1i1m1c1n1 | 28912838 |
| NZ_MBHX01000046 | 26084 | CA | CA | America | Colombia | W | ABCC | K | K2TNSV | s1i1m1c1n1 | 28912838 |
| NZ_MBHV01000021 | 26100 | CA | CA | America | Colombia | W | ABC | K | K1GA2SV | s1i1m1c1n1 | 28912838 |
| NC_000915 | 26695 | NCA | CG | Europe | England | W | ABC | K | K2TNSV | s1i1m1c1n1 | 9252185 |
| NZ_QBPP01000035 | 29:2_single | NCA | VOL | Europe | Sweden | W | ABCCC | K | K2TNSV | s1i1m1c1n1 |  |
| NZ_CP012907 | 29CaP | CA | CA | America | Mexico | W |  | E | EP | s2i2m2c2n2 | 26769924 |
| NZ_MBIX01000003 | 3004 | NCA | IM | America | Colombia | W | ABBCC | K | K2TNSV | s1i1m1c1n1 | 28912838 |
| NZ_MBIY01000198 | 3026 | NCA | IM | America | Colombia | W | ABC | K | K2TNSV | s1i1m1c1n1 | 28912838 |
| NZ_MBIZ01000036 | 3029 | NCA | CG | America | Colombia | W | ABCC | K | K2TNSV | s1i1m1c1n1 | 28912838 |
| NZ_MBJA01000027 | 3033 | NCA | CG | America | Colombia | W | ABC | K | K1GA2V | s1i1m1c1n1 | 28912838 |
| NZ_MBKH01000230 | 3045 | NCA | IM | America | Colombia | W | ABCC | K | K2TNSV | s1i1m1c1n1 | 28912838 |
| NZ_MBGP01000006 | 3046 | NCA | CG | America | Colombia | W | ABC | K | K2TNSV | s1i1m1c1n1 | 28912838 |
| NZ_MBGO01000017 | 3053 | NCA | AG | America | Colombia | W |  | E | EP | s2i2m2c2n1 | 28912838 |
| NZ_MBGN01000031 | 3056 | NCA | AG | America | Colombia | W | ABCC | K | K2TNSV | s1i1m1c1n1 | 28912838 |
| NZ_MBGM01000006 | 3076 | NCA | AG | America | Colombia | W |  | K | K1GP | s2i2m2c2n2 | 28912838 |
| NZ_QBQI01000010 | 30908 | NCA | VOL | Europe | Belgium | W | ABC | K | K1GA1SV | s1i1m2c2n2 |  |
| NZ_QBQH01000001 | 30950 | CA | CA | Europe | Belgium | W | ABC | K | K1GA1SV | s1i1m2c2n2 |  |
| NZ_MBGL01000078 | 3096 | NCA | AG | America | Colombia | W | ABC | Q | QGA2SV | s1i1m1c1n1 | 28912838 |
| NZ_MBHM01000006 | 3118 | NCA | AG | America | Colombia | W | ABC | K | K1GA2V | s1i1m1c1n1 | 28912838 |
| NZ_MBHL01000007 | 3120 | NCA | AG | America | Colombia | W | ABCC | Q | QGA2V | s1i1m1c1n1 | 28912838 |
| NZ_QBQG01000047 | 31235 | NCA | VOL | Europe | Belgium | W |  | K | K2TNSV | s1i1m1c1n1 |  |
| NZ_MBHK01000007 | 3125 | NCA | AG | America | Colombia | W | ABC | K | K1GA1SV | s1i1m1c1n1 | 28912838 |
| NZ_MBHI01000027 | 3136 | NCA | CG | America | Colombia | W |  | E | EP | s2i2m2c2n2 | 28912838 |
| NC_017360 | 35A |  |  | Asia | Japan | E | ABD | K | K1GA2V | s1i1m1c1n1 | 23555707 |
| NZ_QBQF01000040 | 36166 | NCA | VOL | Europe | Belgium | W |  | E | EP | s2i2m2c2n2 |  |
| 3662 | 3697 | NCA | CG | Europe | France | W |  | E | EP | s2i2m2c2n2 |  |
| NZ_QBQK01000046 | 3699 | NCA | CG | Europe | France | W | ABC | K | K1GA1SV | s1i1m1c1n1 |  |
| 3663 | 3738 |  |  | Europe | France | W |  | Q | QGA2SV | s1i1m1c1n1 |  |
| 3658 | 3754 |  |  | Europe | France | W |  | K | K1GA2SV | s1i1m1c1n1 |  |
| NZ_QBQQ01000028 | 3755 | NCA | CG | Europe | France | W | ABC | K | K1GA1SV | s1i1m1c1n1 |  |
| NZ_QBQP01000176 | 3770 | NCA | CG | Europe | France | W | ABB | K | K1GA1SV | s1i1m1c1n1 |  |
| NZ_QBQW01000092 | 3774 | NCA | PU | Europe | France | W | ABC | K | K2TNSV | s1i1m2c2n2 |  |
| 3647 | 3800 | NCA | CG | Europe | France | W |  | E | EP | s2i2m2c2n2 |  |
| NZ_QBQE01000073 | 38185 | CA | CA | Europe | Belgium | W | ABC | K | K1GA2V | s1i1m2c1n2 |  |
| NZ_QBQN01000032 | 3824 | NCA | CG | Europe | France | W | AB | K | K1GA1SV | s1i1m1c1n1 |  |
| NZ_QBQV01000033 | 3843 | NCA | PU | Europe | France | W |  | E | EP | s2i2m2c2n2 |  |
| NZ_QBPN01000045 | 38:2 | NCA | VOL | Europe | Sweden | W | ABC | K | K1GA1SV | s1i1m1c1n1 |  |
| NZ_QBPM01000053 | 38:5 | NCA | VOL | Europe | Sweden | W | ABC | K | K2TNSV | s1i1m2c2n2 |  |
| NZ_MKLV01000007 | 428 | CA | CA | Asia | Singapore | W | ABD | K | K1GA2V | s1i1m1c2n2 |  |
| NZ_QBQD01000017 | 448 | NCA | VOL | Europe | England | W | ABC | K | K2TNSV | s1i1m2c2n2 |  |
| NZ_LIXG01000044 | 45 |  |  | Asia | Kuwait | W | AB | E | EP | s1i2m2c2n2 | 27583131 |
| NZ_QBQC01000025 | 456 | NCA | VOL | Europe | England | W | ABC | E | EP | s1i2m2c2n2 |  |
| NZ_QBQB01000199 | 462 | NCA | VOL | Europe | England | W | ABC | K | K1GA1SV | s1i1m1c1n1 |  |
| NZ_QBPX01000034 | 50 | CA | CA | Europe | Sweden | W | ABCC | K | K3TNSV | s1i1m1c1n1 | 9753641 |
| HQ287752 | 501.9 |  |  | Africa | Africa | W |  | K | K1GA2V | s1i1m1c1n1 | 20870762 |
| NC_017382 | 51 | NCA | DU | Asia | Korea | E | ABD | K | K1GA2V | s1i1m1c1n1 | 27583131 |
| NZ_QBQA01000047 | 518 | NCA | VOL | Europe | England | W | ABC | K | K1TNSV | s1i1m2c2n2 |  |
| NZ_QBPW01000025 | 52 | CA | CA | Europe | Sweden | W | ADD | K | K2TNSV | s1i1m1c1n1 | 27583131 |
| NZ_QBPK01000026 | 55:1 | NCA | VOL | Europe | Sweden | W | ABC | K | K1TNSV | s1i2m2c2n2 | 19338650 |
| NZ_QBPJ01000020 | 55:2 | NCA | VOL | Europe | Sweden | W | ABCC | Q | QGA2SV | s1i1m1c1n1 |  |
| NZ_QBQL01000042 | 565-99 |  |  |  |  |  |  | E | EP | s2i2m2c2n2 |  |
| NZ_QBPV01000035 | 57_single | CA | CA | Europe | Sweden | W |  | E | EP | s2i2m2c2n1 |  |
| NZ_LIXH01000030 | 59 | CA | CA | Asia | Kuwait | W | ABC | K | K1GA2SV | s1i1m1c1n1 | 27583131 |
| HPU05676 | 60190 (ATCC 49503) |  |  | Europe | England | W |  | K | K1GA1SV | s1i1m1c1n1 |  |
| NZ_QBPZ01000011 | 638 | NCA | PU | Europe | England | W | ABC | K | K2TNSV | s1i1m1c1n1 |  |
| NZ_JSXB01000030 | 655/99 | NCA | PU | America | Portugal | W |  | E | EP | s2i2m2c2n2 | 25657274 |
| NZ_QBPU01000070 | 66 | CA | CA | Europe | Sweden | W | ABCC | Q | QGA2SV | s1i1m2c2n1 | 27583131 |
| NZ_QBPT01000201 | 73 | CA | CA | Europe | Sweden | W | ABC | K | K2TNSV | s1i1m1c1n1 | 27583131 |
| NZ_CP012905 | 7C | NCA | CG | America | Mexico | W |  | E | EP | s2i2m2c2n2 | 26744372 |
| NC_017375 | 83 |  |  | Asia | Nepal | W | ABD | K | K1GA2SV | s1i1m1c1n1 | 27583131 |
| 693 | 8A3 |  |  | Asia |  |  |  | K | K1GA2V | s1i1m1c1n1 | 21383187 |
| HPU95971 | 95-54 (J128) | NCA | PU |  |  |  |  | K | K2TNSV | s1i1m2c2n2 |  |
| LC185426 | 97-474 | CA | MALT | Asia | Japan | E |  | K | K1GA2SV | s1i1m1c1n1 | 27833662 |
| NZ_ABSX01000030 | 98-10 | CA | CA | Asia | Japan | E | ABD | K | K1GA2SV | s1i1m1c1n1 | 19123947 |
| 694 | 98_10 |  |  |  |  |  |  | K | K1GA2SV | s1i1m1c1n1 |  |
| NZ_QBPG01000018 | 9:1_single | NCA | VOL | Europe | Sweden | W |  | Q | QGA2SV | s1i1m1c1n1 |  |
| NZ_MBHU01000007 | A037 | NCA | PU | America | Colombia | W | ABC | Q | QGA2SV | s1i1m1c1n1 | 28912838 |
| NZ_MBHT01000057 | A039 | NCA | IM | America | Colombia | W |  | E | EP | s2i2m2c2n2 | 28912838 |
| NZ_AMYU01000001 | A45 | NCA | PU | Europe | Russia | W |  | E | EP | s2i2m2c2n2 |  |
| NZ_QDJV01000010 | AAP164 | NCA | CG | Europe | Spain | W |  | E | EP | s2i2m2c2n2 |  |
| AF191641 | AFN1156 |  |  | Africa | Kenya | W |  | K | K1GP | s2i2m2c2n2 |  |
| AF191642 | AFN4124 |  |  | Africa | Kenya | W |  | K | K1GA1SV | s1i1m1c1n1 |  |
| AF191643 | AFN4769 |  |  | Africa | Kenya | W |  | K | K1GA1SV | s1i1m1c1n1 |  |
| AF191644 | AFN4847 |  |  | Africa | Kenya | W |  | K | K1GA1SV | s1i1m1c1n1 |  |
| AF191645 | AFNG114 |  |  | Africa | Kenya | W |  | K | K1GA1SV | s1i1m1c1n1 |  |
| HPAF001358 | ATCC 43526 |  |  |  |  |  | ABCCC | K | K2TNSV | s1i1m1c1n1 |  |
| NC_019560 | Aklavik117 | NCA | CG | America | Canada | W |  | E | EP | s2i2m2c2n1 | 25883278 |
| NC_019563 | Aklavik86 | NCA | CG | America | Canada | W |  |  | TNS | s1i1m1c1n1 | 25883278 |
| NZ_QEHH01000020 | B126 |  |  | Europe | Spain | W | ABC | K | K2TNSV | s1i1m1c1n1 |  |
| NZ_QBRU01000068 | B23+S27R2:R48 | CA | MALT | Europe | France | W |  | E | EP | s2i2m2c2n2 |  |
| NZ_QDJU01000019 | B247A | CA | CA | Europe | Spain | W | ABC | Q | QGA2SV | s1i1m1c2n2 |  |
| NZ_QBRS01000028 | B25 | CA | MALT | Europe | France | W |  | E | EP | s2i2m2c2n2 |  |
| NZ_QEHG01000024 | B274 | NCA | CG | Europe | Spain | W | ABC | K | K1GA2SV | s1i1m1c1n1 |  |
| NZ_QBRQ01000011 | B29 | CA | MALT | Europe | France | W | ABC | K | K2TNSV | s1i1m1c1n1 |  |
| NZ_QEHF01000019 | B297 | NCA | CG | Europe | Spain | W | ABC | Q | QGA2SV | s1i1m2c2n2 |  |
| NZ_QBRP01000003 | B30 | CA | MALT | Europe | France | W |  | E | EP | s2i2m2c2n2 | 17346936 |
| NZ_QBRO01000008 | B31 | CA | MALT | Europe | France | W | ABC | K | K1GA1SV | s1i1m2c2n2 |  |
| NZ_QEHE01000018 | B314 |  |  | Europe | Spain | W |  | K | K1GA2SV | s1i1m1c1n1 |  |
| NZ_QELC01000010 | B319 | NCA | CG | Europe | Spain | W |  | E | EP | s2i2m2c2n2 | 26991758 |
| NZ_QDJT01000021 | B335 | CA | CA | Europe | Spain | W | ABC | K | K2TNSV | s1i1m1c1n1 |  |
| NZ_QEHC01000017 | B345 | NCA | CG | Europe | Spain | W | ABC | K | K2TNSV | s1i1m1c1n1 |  |
| NZ_QBRN01000053 | B35 | CA | MALT | Europe | France | W | ABC | K | K1TNSV | s1i1m1c1n1 |  |
| NZ_QDJS01000011 | B355 | NCA | CG | Europe | Spain | W |  | E | EP | s2i2m2c2n2 | 26991758 |
| NZ_QEHB01000016 | B360 | NCA | CG | Europe | Spain | W |  | E | EP | s2i2m2c2n1 |  |
| NZ_QEHA01000020 | B362 | NCA | CG | Europe | Spain | W |  | Q | QGA2SV | s1i1m1c1n1 |  |
| NZ_QEGZ01000011 | B366 |  |  | Europe | Spain | W |  | E | EP | s2i2m2c2n2 |  |
| NZ_QEGY01000020 | B368 | NCA | CG | Europe | Spain | W | AB | K | K1GA2SV | s1i1m2c2n2 |  |
| NZ_QBRM01000044 | B37 | CA | MALT | Europe | France | W | ABC | K | K2TNSV | s1i1m2c2n2 |  |
| NZ_QDJR01000018 | B373 |  |  | Europe | Spain | W | ABC | K | K1GA1SV | s1i1m1c1n1 |  |
| NC_012973 | B38 | CA | MALT | Europe | France | W |  | E | EP | s2i2m2c2n2 | 23555707 |
| NZ_QEGX01000020 | B400 | NCA | CG | Europe | Spain | W | ABC | K | K1GA2SV | s1i1m2c2n2 |  |
| NZ_QBRK01000048 | B41 | CA | MALT | Europe | France | W | ABBC | K | K1GA1SV | s1i1m1c1n1 |  |
| NZ_QBRJ01000026 | B43 | CA | MALT | Europe | France | W | ABCC | K | K2TA1SV | s1i1m2c2n2 |  |
| NZ_QDJQ01000063 | B444A |  |  | Europe | Spain | W |  | E | EP | s2i2m2c2n2 |  |
| 640 | B45 | CA | MALT | Europe | France | W |  | E | EP | s1i2m2c2n2 | 22086490 |
| NZ_QDJP01000022 | B464A |  |  | Europe | Spain | W | AB | K | K1GA2SV | s1i1m1c1n1 |  |
| NZ_QBRH01000015 | B47 | CA | MALT | Europe | France | W |  | E | EP | s2i2m2c2n2 |  |
| NZ_QDJO01000016 | B491 | CA | CA | Europe | Spain | W | ABC | K | K1GA1SV | s1i1m1c1n1 | 26991758 |
| NZ_QEGU01000017 | B497A | NCA | CG | Europe | Spain | W | ABC | E | EP | s1i1m2c2n2 |  |
| NZ_QDJN01000008 | B508A-S1 | CA | CA | Europe | Spain | W |  | E | EP | s2i2m2c2n2 |  |
| NZ_QDJM01000009 | B508A-T2A | CA | CA | Europe | Spain | W |  | E | EP | s1i2m2c2n2 |  |
| NZ_QDJL01000011 | B508A-T4 | CA | CA | Europe | Spain | W |  | E | EP | s2i2m2c2n2 |  |
| NZ_QDJK01000010 | B529A | NCA | CG | Europe | Spain | W |  | E | EP | s2i2m2c2n2 |  |
| NZ_QEGS01000017 | B572A |  |  | Europe | Spain | W | ABCC | K | K2TNSV | s1i1m2c2n2 |  |
| NZ_QEGR01000015 | B630 | NCA | CG | Europe | Spain | W | ABC | K | K1GA2V | s1i1m1c1n1 |  |
| NZ_QDJI01000006 | B657-A1 | NCA | CG | Europe | Spain | W |  | E | EP | s2i2m2c2n1 |  |
| NZ_QDJH01000011 | B657-A4 | NCA | CG | Europe | Spain | W |  | E | EP | s2i2m2c2n2 |  |
| NZ_QDJG01000015 | B657-C1 | NCA | CG | Europe | Spain | W |  | E | EP | s2i2m2c2n2 |  |
| NZ_QDJF01000016 | B659-A1 | NCA | CG | Europe | Spain | W |  | K | K2TNSV | s1i1m1c1n1 |  |
| NZ_QDJE01000014 | B659-C2 | NCA | CG | Europe | Spain | W |  | K | K2TNSV | s1i1m1c1n1 |  |
| NZ_QEGQ01000017 | B661A | NCA | CG | Europe | Spain | W |  | Q | QGA2SV | s1i1m2c2n1 |  |
| NZ_QEGP01000018 | B679 | NCA | CG | Europe | Spain | W | ABC | K | K1GA1SV | s1i1m2c2n2 |  |
| NZ_QEGO01000012 | B712A | NCA | CG | Europe | Spain | W |  | E | EP | s2i2m2c2n2 |  |
| NZ_FLKK01000008 | BAC555-2 |  |  |  |  |  | ABC | K | K2TNSV | s1i1m1c1n1 |  |
| NZ_FLKJ01000039 | BAC555-8 |  |  |  |  |  | ABC | K | K2TNSV | s1i1m1c1n1 |  |
| NZ_LT837687 | BCM-300 |  |  |  |  |  | ABC | K | K1GA1SV | s1i1m1c1n1 |  |
| NZ_CADC01000005 | BCS100H1 | NCA | CG | America | USA | W |  | K | K1GA2V | s1i1m1c1n1 | 21383187 |
| LC187585 | BH10 |  |  | Asia | Bangladesh | W | ABC | K | K1GA2SV | s1i1m1c1n1 |  |
| LC187590 | BH101 |  |  | Asia | Bangladesh | W | ABC | K | K1GA2V | s1i1m2c2n2 |  |
| LC187583 | BH104 |  |  | Asia | Bangladesh | W | AB | K | K1GA2V | s1i1m1c1n1 |  |
| LC187575 | BH107 |  |  | Asia | Bangladesh | W | ABCC | K | K1GA2V | s1i1m1c1n1 |  |
| LC187562 | BH109 |  |  | Asia | Bangladesh | W | ABC | K | K1GA2V | s1i1m1c1n1 |  |
| LC187605 | BH1101 |  |  | Asia | Bangladesh | W |  | E | EP | s2i2m2c2n1 |  |
| LC187577 | BH112 |  |  | Asia | Bangladesh | W | ABCC | K | K1GA1V | s1i1m1c1n1 |  |
| LC187599 | BH114 |  |  | Asia | Bangladesh | W |  | E | EP | s2i2m2c2n2 |  |
| LC187567 | BH115 |  |  | Asia | Bangladesh | W | ABC | K | K1GA2V | s1i1m1c1n1 |  |
| LC187573 | BH119 |  |  | Asia | Bangladesh | W | ABC | K | K1GA2V | s1i1m1c1n1 |  |
| LC187572 | BH120 |  |  | Asia | Bangladesh | W | ABCC | K | K1GA2SV | s1i1m1c1n1 |  |
| LC187612 | BH127 |  |  | Asia | Bangladesh | W | ABC | E | EP | s1i2m2c2n2 |  |
| LC187559 | BH13 |  |  | Asia | Bangladesh | W | ABC | K | K1GA2V | s1i1m1c1n1 | 22162751 |
| LC187564 | BH130 |  |  | Asia | Bangladesh | W | ABC | K | K1GA3V | s1i1m1c1n1 |  |
| LC187580 | BH133 |  |  | Asia | Bangladesh | W | ABCC | K | K1GA2SV | s1i1m1c1n1 |  |
| LC187568 | BH14 |  |  | Asia | Bangladesh | W |  | K | K1GA2V | s1i1m1c1n1 |  |
| LC187584 | BH16 |  |  | Asia | Bangladesh | W | ABC | K | K1GA2V | s1i1m1c1n1 |  |
| LC187610 | BH24 |  |  | Asia | Bangladesh | W |  | E | EP | s1i2m2c2n2 |  |
| LC187611 | BH27 |  |  | Asia | Bangladesh | W | ABCCC | E | EP | s1i2m2c2n2 |  |
| LC187563 | BH3 |  |  | Asia | Bangladesh | W | ABCC | K | K1GA2SV | s1i1m1c1n1 |  |
| LC187593 | BH34 |  |  | Asia | Bangladesh | W | ABCC | K | K1GA2V | s1i1m2c2n2 |  |
| LC187592 | BH36 |  |  | Asia | Bangladesh | W | ABC | E | EP | s1i1m2c2n1 |  |
| LC187587 | BH42 |  |  | Asia | Bangladesh | W | ABC | K | K1GA2SV | s1i1m1c2n2 |  |
| LC187597 | BH45 |  |  | Asia | Bangladesh | W |  | E | EP | s2i2m2c2n2 |  |
| LC187589 | BH46 |  |  | Asia | Bangladesh | W | ABC | K | K1GA2V | s1i1m2c2n2 |  |
| LC187588 | BH47 |  |  | Asia | Bangladesh | W | ABC | K | K1GA2V | s1i1m1c2n2 |  |
| LC187560 | BH49 |  |  | Asia | Bangladesh | W | ABC | K | K1GA2SV | s1i1m1c1n1 |  |
| LC187565 | BH52 |  |  | Asia | Bangladesh | W | AB | K | K1GA2SV | s1i1m1c1n1 |  |
| LC187591 | BH53 |  |  | Asia | Bangladesh | W | ABCC | K | K1GA2SV | s1i1m2c2n2 |  |
| LC187576 | BH63 |  |  | Asia | Bangladesh | W | ABC | K | K1GA2V | s1i1m1c1n1 |  |
| LC187586 | BH65 |  |  | Asia | Bangladesh | W | ABCCC | K | K1GA2V | s1i1m1c2n2 |  |
| LC187569 | BH69 |  |  | Asia | Bangladesh | W | ABC | K | K1GA2V | s1i1m1c1n1 |  |
| LC187608 | BH70 |  |  | Asia | Bangladesh | W |  | E | EP | s1i2m2c2n2 |  |
| LC187570 | BH72 |  |  | Asia | Bangladesh | W | ABC | K | K1GA2SV | s1i1m1c1n1 |  |
| LC187578 | BH73 |  |  | Asia | Bangladesh | W | ABC | K | K1GA2SV | s1i1m1c1n1 |  |
| LC187607 | BH76 |  |  | Asia | Bangladesh | W |  | E | EP | s2i2m2c2n2 |  |
| LC187602 | BH77 |  |  | Asia | Bangladesh | W | ABC | E | EP | s2i2m2c2n2 |  |
| LC187601 | BH78 |  |  | Asia | Bangladesh | W | ABC | E | EP | s2i2m2c2n2 |  |
| LC187606 | BH80 |  |  | Asia | Bangladesh | W |  | E | EP | s2i2m2c2n2 |  |
| LC187581 | BH82 |  |  | Asia | Bangladesh | W |  | K | K1GA2SV | s1i1m1c1n1 |  |
| LC187600 | BH84 |  |  | Asia | Bangladesh | W | ABC | E | EP | s2i2m2c2n2 |  |
| LC187566 | BH86 |  |  | Asia | Bangladesh | W | ABC | K | K1GA2SV | s1i1m1c1n1 |  |
| LC187561 | BH91 |  |  | Asia | Bangladesh | W | ABC | K | K1GA2V | s1i1m1c1n1 |  |
| LC187574 | BH94 |  |  | Asia | Bangladesh | W | ABC | K | K1GA2V | s1i1m1c1n1 |  |
| LC187582 | BH95 |  |  | Asia | Bangladesh | W | ABC | K | K1GA2V | s1i1m1c1n1 |  |
| LC187603 | BH96 |  |  | Asia | Bangladesh | W |  | E | EP | s2i2m2c2n2 |  |
| LC187596 | BH97 |  |  | Asia | Bangladesh | W |  | E | EP | s2i2m2c2n2 |  |
| NZ_CP007604 | BM013A | NCA | VOL | Oceania | Australia | W |  | E | EP | s2i2m2c2n2 | 24924186 |
| NZ_QEGN01000010 | BMG112 | NCA | CG | Europe | Spain | W |  | E | EP | s2i2m2c2n2 |  |
| NZ_AMFG01000002 | C333 | CA | CA | Asia | China | E | ABD | K | K1GA2V | s1i1m2c2n2 |  |
| NZ_MUOC01000010 | CA22019 | NCA | AG | America | Colombia | W | ABC | K | K1GA2V | s1i1m1c1n1 | 28293542 |
| NZ_MUOD01000109 | CA22020 | NCA | AG | America | Colombia | W |  | E | EP | s2i2m2c2n2 | 28293542 |
| NZ_MUOF01000136 | CA22311 | NCA | AG | America | Colombia | W | ABCC | Q | QGA2SV | s1i1m1c1n1 | 28293542 |
| NZ_MUOG01000080 | CA22312 | NCA | AG | America | Colombia | W |  | K | K1GA1SV | s1i1m1c1n1 | 28293542 |
| NZ_MUOI01000088 | CA22335 | NCA | AG | America | Colombia | W | ABC | Q | QGA2SV | s1i1m1c1n1 | 28293542 |
| NZ_MUOK01000107 | CA22339 | NCA | AG | America | Colombia | W | ABC | Q | QGA2SV | s1i1m1c1n1 | 28293542 |
| NZ_MUOL01000069 | CA22362 | NCA | AG | America | Colombia | W | ABC | K | K1GA2SV | s1i1m1c1n1 | 28293542 |
| NZ_MUOM01000192 | CA22393 | NCA | AG | America | Colombia | W | ABC | K | K2TNSV | s1i1m1c1n1 | 28293542 |
| NZ_MUON01000059 | CA24004 | NCA | AG | America | Colombia | W | ABC | K | K2TA1SV | s1i1m1c1n1 | 28293542 |
| NZ_MUOO01000173 | CA26024 | NCA | AG | America | Colombia | W | ABC | Q | QGA2SV | s1i1m1c1n1 | 28293542 |
| NZ_MUOP01000270 | CC22093 | CA | CA | America | Colombia | W |  | E | EP | s2i2m2c2n2 | 28293542 |
| NZ_MUOQ01000009 | CC22402 | CA | CA | America | Colombia | W | ABC | K | K1GA2V | s1i1m1c1n1 | 28293542 |
| NZ_MUOR01000021 | CC26084 | CA | CA | America | Colombia | W | ABC | K | K2TNSV | s1i1m1c1n1 | 28293542 |
| NZ_MUOT01000027 | CC26100 | CA | CA | America | Colombia | W | ABC | K | K1GA2SV | s1i1m1c1n1 | 28293542 |
| NZ_CP011484 | CC33C |  |  | Africa | South Africa | W | ABC | K | K1GA1V | s1i1m1c1n1 | 20808891 |
| NZ_AWUL01000013 | CG-IMSS-2012 | CA | CA | America | Mexico | W |  | K | K1GA2V | s1i1m1c1n1 | 24459275 |
| NZ_MUOU01000052 | CG22023 | NCA | CG | America | Colombia | W |  | K | K2TNSV | s1i1m1c1n1 | 28293542 |
| NZ_MUOW01000013 | CG22087 | NCA | CG | America | Colombia | W | ABC | K | K1GA2V | s1i1m2c2n2 | 28293542 |
| NZ_MUOX01000054 | CG22322 | NCA | CG | America | Colombia | W |  | E | EP | s2i2m2c2n2 | 28293542 |
| NZ_MUOY01000104 | CG22366 | NCA | CG | America | Colombia | W | ABC | Q | QGA2SV | s1i1m1c1n1 | 28293542 |
| NZ_MUOZ01000053 | CG22367 | NCA | CG | America | Colombia | W | ABC | Q | QGA2SV | s1i1m1c1n1 | 28293542 |
| NZ_MUPA01000061 | CG22370 | NCA | CG | America | Colombia | W | ABC | Q | QGA2SV | s1i1m1c1n1 | 28293542 |
| NZ_MUPB01000015 | CG22371 | NCA | CG | America | Colombia | W | ABC | K | K1GA1SV | s1i1m1c1n1 | 28293542 |
| NZ_MUPC01000161 | CG22378 | NCA | CG | America | Colombia | W |  | K | K1GA2V | s1i1m1c1n1 | 28293542 |
| NZ_MUPD01000137 | CG22385 | NCA | CG | America | Colombia | W | ABC | K | K1GA2V | s1i1m1c1n1 | 28293542 |
| NZ_MUPE01000054 | CG22389 | NCA | CG | America | Colombia | W |  | E | EP | s2i2m2c2n2 | 28293542 |
| 3640 | CHA_185 |  |  |  |  |  |  | K | K2TNSV | s1i1m1c1n1 |  |
| AF050326 | CHN1811a |  |  |  |  |  |  | K | K1GA2V | s1i1m2c2n1 |  |
| AF050319 | CHN3295b |  |  |  |  |  |  | K | K1GA2SV | s1i1m2c2n2 |  |
| AF361700 | CHN3554a |  |  |  |  |  |  | K | K1GA2SV | s1i1m1c1n1 |  |
| AF361701 | CHN4611a |  |  |  |  |  |  | K | K1GA2SV | s1i1m1c1n1 |  |
| AF361702 | CHN5038c |  |  |  |  |  |  | K | K1GA2V | s1i1m1c1n1 |  |
| AF050328 | CHN5060d |  |  |  |  |  |  | K | K1GA2V | s1i1m1c1n1 |  |
| AF050327 | CHN5114a |  |  |  |  |  |  | K | K1GA2V | s1i1m2c2n2 |  |
| AF050320 | CHN5147c |  |  |  |  |  |  | K | K1GA2V | s1i1m2c2n1 |  |
| NZ_MUPG01000144 | CM22021 | NCA | IM | America | Colombia | W | ABC | Q | QGA2SV | s1i1m1c1n1 | 28293542 |
| NZ_MUPH01000105 | CM22046 | NCA | IM | America | Colombia | W | ABC | K | K2TNSV | s1i1m1c1n1 | 28293542 |
| NZ_MUPI01000073 | CM22315 | NCA | IM | America | Colombia | W | ABC | K | K1GA2V | s1i1m1c1n1 | 28293542 |
| NZ_MUPJ01000099 | CM22331 | NCA | IM | America | Colombia | W |  | E | EP | s2i2m2c2n2 | 28293542 |
| NZ_MUPK01000086 | CM22341 | NCA | IM | America | Colombia | W |  | Q | QGA2SV | s1i1m1c1n1 | 28293542 |
| NZ_MUPL01000007 | CM22346 | NCA | IM | America | Colombia | W | ABC | K | K1GA1SV | s1i1m1c1n1 | 28293542 |
| NZ_MUPM01000092 | CM22347 | NCA | IM | America | Colombia | W |  | K | K1GA2SV | s1i1m1c1n1 | 28293542 |
| NZ_MUPO01000050 | CM22360 | NCA | IM | America | Colombia | W | ABC | K | K1GA2V | s1i1m2c2n2 | 28293542 |
| NZ_MUPP01000031 | CM22368 | NCA | IM | America | Colombia | W | ABBC | Q | QGA2SV | s1i1m1c1n1 | 28293542 |
| NZ_MUPQ01000420 | CM22388 | NCA | IM | America | Colombia | W |  | Q | QGA2SV | s1i1m1c1n1 | 28293542 |
| NZ_MUOB01000003 | CM22390 | NCA | IM | America | Colombia | W | ABC | K | K1GA2V | s1i1m1c1n1 | 28293542 |
| NZ_AKNJ01000009 | CPY1124 | NCA | GU | Asia | Japan | E | ABD | K | K1GA2SV | s1i1m1c1n1 | 23661595 |
| NZ_AKNK01000001 | CPY1313 | NCA | DU | Asia | Japan | E | ABD | K | K1GA2V | s1i1m1c1n1 | 23661595 |
| NZ_AOTT01000005 | CPY1662 | NCA | DU | Asia | Japan | E | ABD | K | K1GA2V | s1i1m2c2n2 |  |
| NZ_AKNM01000005 | CPY3281 | NCA | DU | Asia | Japan | E | ABD | K | K1GA2V | s1i1m1c1n1 | 23661595 |
| NZ_AKNN01000005 | CPY6081 | CA | CA | Asia | Japan | E | ABD | K | K1GA2V | s1i1m1c1n1 | 23661595 |
| NZ_AKNO01000004 | CPY6261 | CA | CA | Asia | Japan | E | ABD | K | K1GA2SV | s1i1m1c1n1 | 23661595 |
| NZ_AKNP01000001 | CPY6271 | CA | CA | Asia | Japan | E | ABD | K | K1GA2V | s1i1m1c1n1 | 23661595 |
| NZ_AKNQ01000002 | CPY6311 | CA | CA | Asia | Japan | E | ABD | K | K1GA2SV | s1i1m1c1n1 | 23661595 |
| NZ_QDJD01000010 | CRL122 | NCA | CG | Europe | Spain | W |  | E | EP | s2i2m2c2n2 | 26991758 |
| NZ_QEGM01000018 | CRM21 | NCA | CG | Europe | Spain | W | ABC | K | K1GA1SV | s1i1m1c1n1 |  |
| NC_017358 | Cuz20 |  |  | America | Peru | W | ABD | E | EP | s1i2m2c2n1 |  |
| NZ_ANIO01000025 | D33 | CA | CA | Asia | China | E |  | E | EP | s1i2m2c2n2 | 24914326 |
| GQ331980 | DL1 |  |  | Asia | India | W |  | K | K1GA2SV | s1i1m1c1n1 |  |
| GQ331981 | DL2 |  |  | Asia | India | W |  | K | K1GA2V | s1i1m1c1n1 |  |
| NZ_CP011483 | DU15 |  |  | Asia | Korea | E | ABD | K | K1GA2V | s1i1m1c1n1 | 20808891 |
| NZ_QEGL01000017 | ECF139-B065 |  |  | Europe | Spain | W | ABC | K | K1GA2SV | s1i1m1c1n1 |  |
| NC_017063 | ELS37 | CA | CA | America | El Salvador | W | ABC | Q | QGA2SV | s1i1m1c1n1 |  |
| NZ_AP017329 | F13 | NCA | CG | Asia | Japan | E | ABD | K | K1GA2SV | s1i1m1c1n1 | 27604221 |
| AB190959 | F15 | NCA | CG | Asia | Japan | E | ABD | K | K1GA2V | s1i1m1c1n1 | 16081930 |
| NC_017368 | F16 | NCA | CG | Asia | Japan | E | ABD | K | K1GA2V | s1i1m1c1n1 | 21575176 |
| NZ_AP017330 | F17 | NCA | CG | Asia | Japan | E | AABD | K | K1GA2SV | s1i1m1c1n1 | 27604221 |
| NZ_AP017331 | F18 | NCA | CG | Asia | Japan | E | ABABD | K | K1GA2V | s1i1m1c1n1 | 27604221 |
| NZ_AP017336 | F21 | NCA | DU | Asia | Japan | E | ABD | K | K1GA2V | s1i1m1c1n1 | 9780260 |
| NZ_AP017334 | F210 |  |  | Asia | Japan | E | ABD | K | K1GA2V | s1i1m1c1n1 | 27604221 |
| NZ_AP017335 | F211 |  |  | Asia | Japan | E | ABD | K | K1GA2SV | s1i1m1c1n1 | 27604221 |
| NZ_AP017337 | F23 | NCA | DU | Asia | Japan | E | ABD | K | K1GA2V | s1i1m1c1n1 | 27604221 |
| NZ_AP017338 | F24 | NCA | DU | Asia | Japan | E | ABD | K | K1GA2SV | s1i1m1c1n1 | 27604221 |
| AB190963 | F26 | NCA | CG | Asia | Japan | E | ABABD | K | K1GA2SV | s1i1m1c1n1 | 16081930 |
| NC_017365 | F30 | NCA | DU | Asia | Japan | E | ABD | K | K1GA2SV | s1i1m1c1n1 | 21575176 |
| NC_017366 | F32 | CA | CA | Asia | Japan | E | ABD | K | K1GA2V | s1i1m1c1n1 | 21575176 |
| AF049641 | F33 | NCA | DU | Asia | Japan | E | ABD | K | K1GA2SV | s1i1m1c1n1 | 9780260 |
| AF049625 | F35 | NCA | DU | Asia | Japan | E | ABD | K | K1GA2SV | s1i1m1c1n1 | 9780260 |
| AF049642 | F36 | NCA | CG | Asia | Japan | E | ABD | K | K1GA2SV | s1i1m1c1n1 | 9780260 |
| AF071095 | F37 | NCA | CG | Asia | Japan | E | ABD | K | K1GA2SV | s1i1m1c1n1 | 9196179 |
| NZ_AP017341 | F38 | NCA | CG | Asia | Japan | E | ABD | K | K1GA2V | s1i1m1c1n1 | 9780260 |
| AF049626 | F42 |  |  | Asia | Japan | E |  | K | K1GA2SV | s1i1m1c1n1 | 9780260 |
| AF049644 | F43 | NCA | CG | Asia | Japan | E | ABD | K | K1GA2V | s1i1m1c1n1 | 9780260 |
| AF049627 | F44 | NCA | DU | Asia | Japan | E | AD | K | K1GA2V | s1i1m1c1n1 | 9780260 |
| AF049628 | F45 |  |  | Asia | Japan | E |  | K | K1GA2V | s1i1m1c1n1 | 9780260 |
| AF049629 | F47 | NCA | DU | Asia | Japan | E | ABD | K | K1GA2V | s1i1m1c1n1 | 9780260 |
| AF049631 | F52 | NCA | DU | Asia | Japan | E |  | K | K1GA2V | s1i1m1c1n1 | 9780260 |
| NZ_AP017345 | F55 | NCA | CG | Asia | Japan | E | ABDD | K | K1GA2V | s1i1m1c1n1 | 9780260 |
| AF049633 | F56 | NCA | CG | Asia | Japan | E | ABDABD | K | K1GA2V | s1i1m1c1n1 | 9780260 |
| NC_017367 | F57 | CA | CA | Asia | Japan | E | ABD | K | K1GA2SV | s1i1m1c1n1 | 9780260 |
| AF049645 | F61 |  |  | Asia | Japan | E |  | K | K1GA2V | s1i1m1c1n1 | 9780260 |
| AF049647 | F64 |  |  | Asia | Japan | E |  | K | K1GA2V | s1i1m1c1n1 | 9780260 |
| AF049636 | F65 | NCA | CG | Asia | Japan | E | ABD | K | K1GA2V | s1i1m1c1n1 | 9780260 |
| AF049648 | F68 |  |  | Asia | Japan | E |  | K | K1GA2SV | s1i1m1c1n1 | 9780260 |
| AF049649 | F69 |  |  | Asia | Japan | E |  | K | K1GA2V | s1i1m1c1n1 | 9780260 |
| NZ_AP017349 | F70 | NCA | PU | Asia | Japan | E | ABD | K | K1GA3V | s1i1m1c1n1 | 9780260 |
| AF071096 | F71 |  |  | Asia | Japan | E |  | K | K1GA2SV | s1i1m1c1n1 | 9196179 |
| NZ_AP017350 | F72 | NCA | PU | Asia | Japan | E | ABD | K | K1GA2V | s1i1m1c1n1 | 9780260 |
| AF049652 | F73 | NCA | DU | Asia | Japan | E | ABD | K | K1GA2SV | s1i1m1c1n1 | 9780260 |
| NZ_AP017352 | F78 |  |  | Asia | Japan | E | ABD | K | K1GA2V | s1i1m1c1n1 | 9780260 |
| AF071097 | F79 | NCA | GU | Asia | Japan | E | ABCC | K | K1GA2V | s1i1m1c1n1 | 9196179 |
| AB190965 | F80 | NCA | DU | Asia | Japan | E | ABCD | K | K1GA2SV | s1i1m1c1n1 | 9196179 |
| NZ_AP017354 | F90 |  |  | Asia | Japan | E | ABD | K | K1GA2V | s1i1m1c1n1 | 27604221 |
| AB190966 | F92 | NCA | CG | Asia | Japan | E | ABABD | K | K1GA2SV | s1i1m1c1n1 | 16081930 |
| NZ_AP017355 | F94 |  |  | Asia | Japan | E | ABDD | K | K1GA2V | s1i1m1c1n1 | 9780260 |
| NZ_AKHR02000049 | FD577 | NCA | FD | Asia | Malaysia | W | ABD | K | K1GA2V | s1i1m1c2n2 | 24051312 |
| NZ_CP028325 | FDAARGOS_298 | NCA | CG | America | USA | W | ABCCC | K | K2TNSV | s1i1m1c1n1 |  |
| NJFC02000001 | FDAARGOS_299 | NCA | CG | America | USA | W | ABC | K | K2TNSV | s1i1m1c1n1 |  |
| NZ_CP027404 | FDAARGOS_300 | NCA | DU | America | USA | W | AC | K | K1GA1SV | s1i1m1c1n1 |  |
| NC_011333 | G27 |  |  | Europe | Italy | W | ABCC | K | K1GA2SV | s1i1m1c1n1 | 9782498 |
| NZ_CP022409 | G272 | NCA | CG | Asia | China | E | ABD | K | K1GA2SV | s1i1m2c2n2 |  |
| NZ_MWUG01000109 | G4 |  |  | Africa | Morocco | W |  | K | K1GA1SV | s1i1m1c1n1 |  |
| NZ_KB636795 | GAM101Biv |  |  | Africa | Gambia | W |  | K | K1GP | s2i2m2c2n2 |  |
| NZ_KB637030 | GAM105Ai |  |  | Africa | Gambia | W | ABC | K | K1A1SV | s1i1m1c1n1 |  |
| NZ_KB641843 | GAM112Ai |  |  | Africa | Gambia | W | ABC | K | K1GA1SV | s1i1m1c1n1 |  |
| NZ_KB642385 | GAM114Ai |  |  | Africa | Gambia | W | ABC | K | K1GA1SV | s1i1m1c1n1 |  |
| NZ_KB644403 | GAM115Ai |  |  | Africa | Gambia | W | ABC | K | K1A1SV | s1i1m2c2n1 |  |
| NZ_KE698743 | GAM117Ai |  |  | Africa | Gambia | W | ABC | K | K1GA1SV | s1i1m1c1n1 |  |
| NZ_KB644501 | GAM118Bi |  |  | Africa | Gambia | W | ABC | K | K1GP | s1i1m2c2n1 |  |
| NZ_KB644569 | GAM119Bi |  |  | Africa | Gambia | W | ABC | K | K1GA1SV | s1i1m1c1n1 |  |
| NZ_KB644460 | GAM120Ai |  |  | Africa | Gambia | W | ABC | K | K1GA1SV | s1i1m1c1n1 |  |
| NZ_KB636380 | GAM121Aii |  |  | Africa | Gambia | W | ABC | K | K1GA1SV | s1i1m1c1n1 |  |
| NZ_KB641893 | GAM201Ai |  |  | Africa | Gambia | W |  | K | K1GP | s2i2m2c2n2 |  |
| NZ_KB642830 | GAM210Bi |  |  |  |  |  | ABC | K | K1GA1SV | s1i1m1c1n1 |  |
| NZ_KB641695 | GAM231Ai |  |  | Africa | Gambia | W |  | E | EP | s2i2m2c2n2 |  |
| NZ_KB636348 | GAM239Bi |  |  | Africa | Gambia | W |  | E | EP | s2i2m2c2n2 |  |
| NZ_KB636666 | GAM244Ai |  |  | Africa | Gambia | W |  | K | K1GP | s2i2m2c2n2 |  |
| NZ_KB636250 | GAM246Ai |  |  | Africa | Gambia | W | ABC | K | K1GA1SV | s1i1m1c1n1 |  |
| NZ_KB636083 | GAM249T |  |  | Africa | Gambia | W | ABC | K | K1GA1SV | s1i1m1c1n1 |  |
| NZ_KB635999 | GAM250AFi |  |  |  |  |  | ABC | K | K1GA1SV | s1i1m1c1n1 |  |
| NZ_KB635961 | GAM250T |  |  |  |  |  | ABC | K | K1GA1SV | s1i1m1c1n1 |  |
| NZ_KB636441 | GAM252Bi |  |  |  |  |  | ABC | K | K1GA1SV | s1i1m1c1n1 |  |
| NZ_KB636562 | GAM252T |  |  |  |  |  | ABC | K | K1GA1SV | s1i1m1c1n1 |  |
| NZ_KB636762 | GAM254Ai |  |  | Africa | Gambia | W | ABC | E | EP | s1i2m2c2n2 |  |
| NZ_KB636645 | GAM260ASi |  |  | Africa | Gambia | W | ABC | K | K1GA1SV | s1i1m1c1n1 |  |
| NZ_KB636976 | GAM260BSi |  |  | Africa | Gambia | W |  | K | K1GP | s2i2m2c2n2 |  |
| NZ_KB636865 | GAM260Bi |  |  | Africa | Gambia | W | ABC | K | K1GA1SV | s1i1m1c1n1 |  |
| NZ_KB642543 | GAM264Ai |  |  | Africa | Gambia | W |  | K | K1GP | s2i2m2c2n2 |  |
| NZ_KB642330 | GAM265BSii |  |  | Africa | Gambia | W |  | K | K1GP | s1i1m2c2n2 |  |
| NZ_KB642663 | GAM268Bii |  |  |  |  |  | ABC | K | K1GA1SV | s1i1m1c1n1 |  |
| NZ_KB641801 | GAM270ASi |  |  | Africa | Gambia | W | ABC | K | K1GA1SV | s1i1m1c1n1 |  |
| NZ_KB642143 | GAM42Ai |  |  |  |  |  | ABC | K | K1GA1SV | s1i1m1c1n1 |  |
| NZ_KB642078 | GAM71Ai |  |  | Africa | Gambia | W |  | K | K1GP | s2i2m2c2n2 |  |
| NZ_KB641975 | GAM80Ai |  |  | Africa | Gambia | W | ABC | K | K1GA1SV | s1i1m1c1n1 |  |
| NZ_KB641928 | GAM83T |  |  |  |  |  |  | E | EP | s2i2m2c2n2 |  |
| NZ_KB642119 | GAM93Bi |  |  |  |  |  | ABC | K | K1GA1SV | s1i1m1c1n1 |  |
| NZ_KB642376 | GAMchJs106B |  |  |  |  |  |  | E | EP | s2i2m2c2n2 |  |
| NZ_KB644619 | GAMchJs114i |  |  |  |  |  |  | K | K1GP | s2i2m2c2n2 |  |
| NZ_KB644762 | GAMchJs117Ai |  |  |  |  |  |  | E | EP | s2i2m2c2n2 |  |
| NZ_KB644722 | GAMchJs124i |  |  |  |  |  |  | K | K1GP | s2i2m2c2n2 |  |
| NZ_KB644675 | GAMchJs136i |  |  |  |  |  | ABC | K | K1GA1SV | s1i1m1c1n2 |  |
| 747 | GAMchjs106B |  |  | Africa | Gambia | W |  | E | EP | s2i2m2c2n2 |  |
| 748 | GAMchjs114i |  |  | Africa | Gambia | W |  | K | K1GP | s2i2m2c2n2 |  |
| 749 | GAMchjs117Ai |  |  | Africa | Gambia | W |  | E | EP | s2i2m2c2n2 |  |
| 750 | GAMchjs124i |  |  | Africa | Gambia | W |  | K | K1GP | s2i2m2c2n2 |  |
| 751 | GAMchjs136i |  |  | Africa | Gambia | W |  | K | K1GA1SV | s1i1m1c1n2 |  |
| NZ_AKHV02000095 | GC26 | CA | CA | Asia | Malaysia | W | ABD | K | K1GA2V | s1i1m1c2n2 | 24051312 |
| NZ_QBQS01000049 | GC30-HL | CA | CA | Europe | France | W | ABC | K | K2TA1SV | s1i1m1c1n1 |  |
| NZ_QBRB01000064 | GC43-HL | CA | CA | Europe | France | W | ABC | K | K2TNSV | s1i1m1c2n2 |  |
| NZ_QBQR01000066 | GC52-HL | CA | CA | Europe | France | W | ABC | Q | QGA2SV | s1i1m1c1n1 |  |
| NZ_QBQZ01000014 | GC65-HL | CA | CA | Europe | France | W | ABC | K | K1GA2SV | s1i1m1c1n1 |  |
| NZ_QBQJ01000001 | GC69-HL | CA | CA | Europe | France | W | AC | K | K2TNSV | s1i1m1c1n1 |  |
| NZ_CP031558 | GD63 | NCA | GU |  | Viet Nam | W | ABD | K | K1GA2V | s1i1m2c2n1 |  |
| QBQT01000542 | GIL237 | NCA | PU | Europe | France | W | AB |  | A2SV | s1i1m2c2n2 |  |
| NC_017371 | Gambia94/24 |  |  | Africa | Gambia | W | ABC | K | K1GA1SV | s1i1m1c1n1 | 10809702 |
| 639 | Gambia94_24 |  |  |  |  |  |  | K | K1GA1SV | s1i1m1c1n1 |  |
| NZ_CP025474 | H-137 |  |  | Asia | Korea | E | ABD | K | K1GA2V | s1i1m1c1n1 |  |
| NZ_MJNA01000009 | H30 | CA | CA | Asia | Singapore | W | ABD | K | K1GA2V | s1i1m1c1n1 |  |
| NZ_CCMU01000017 | H3014 |  |  | Europe | England | W | ABC | K | K2TNSV | s1i1m1c1n1 | 12748272 |
| NZ_CCMT01000004 | H3016 |  |  | Europe | England | W |  | E | EP | s1i1m2c2n2 | 12748272 |
| NZ_CCMW01000020 | H3018 |  |  | Europe | England | W |  | K | K2TNSV | s1i1m2c2n2 | 12748272 |
| NZ_MJNB01000004 | H30A | CA | CA | Asia | Singapore | W | ABD | K | K1GA2V | s1i1m1c1n1 |  |
| NZ_LT635459 | HE132/09 |  |  |  |  |  | ABC | K | K1GA1SV | s1i1m1c1n1 |  |
| NZ_LT635476 | HE134/09 |  |  |  |  |  | ABC | K | K1GA1SV | s1i1m1c1n1 |  |
| NZ_LT635473 | HE136/09 |  |  |  |  |  | ABC | K | K1GA1SV | s1i1m1c1n1 |  |
| NZ_LT635471 | HE141/09 |  |  |  |  |  | ABC | K | K1GA1SV | s1i1m1c1n1 |  |
| NZ_LT635478 | HE142/09 |  |  |  |  |  | ABC | K | K1GA1SV | s1i1m1c1n1 |  |
| NZ_LT635458 | HE143/09 |  |  |  |  |  | ABC | K | K1GA1SV | s1i1m1c1n1 |  |
| NZ_LT635477 | HE147/09 |  |  |  |  |  | ABC | K | K1GA1SV | s1i1m1c1n1 |  |
| NZ_LT635472 | HE170/09 |  |  |  |  |  | ABC | K | K1GA1SV | s1i1m1c1n1 |  |
| NZ_LT635474 | HE171/09 |  |  |  |  |  | ABC | K | K1GA1SV | s1i1m1c1n1 |  |
| NZ_JAAA01000007 | HLJ039 | CA | CA | Asia | China | E | ABD | K | K1GA2V | s1i1m2c2n1 | 24565107 |
| NZ_JH791774 | HLJHP193 | NCA | AG | Asia | China | E | ABD | K | K1GA2V | s1i1m1c1n1 | 23105058 |
| NZ_JH791474 | HLJHP253 |  |  | Asia | China | E | ABBD | K | K1GA2V | s1i1m2c2n2 |  |
| NZ_JH791471 | HLJHP256 | NCA | AG | Asia | China | E | ABD | K | K1GA2V | s1i1m2c2n2 | 23105058 |
| NZ_JH791472 | HLJHP271 | NCA | GU | Asia | China | E | ABD | K | K1GA2SV | s1i1m2c2n2 | 23105058 |
| NZ_MVYS01000018 | HP00152 |  |  | Oceania | Australia | W | ABC | K | K2TNSV | s1i1m1c1n1 |  |
| NZ_MVYQ01000002 | HP00248 |  |  | Oceania | Australia | W | ABC | K | K2TNSV | s1i1m1c1n1 |  |
| NZ_MVYO01000016 | HP01102 |  |  | Oceania | Australia | W | ABC | K | K1GA1SV | s1i1m1c1n1 |  |
| NZ_MVYL01000003 | HP01234 |  |  | Oceania | Australia | W |  | E | EP | s1i2m2c2n2 |  |
| NZ_MVYK01000018 | HP01306 |  |  | Oceania | Australia | W | ABC | K | K2TNSV | s1i1m1c1n1 |  |
| NZ_MVYH01000015 | HP01330 |  |  | Oceania | Australia | W | ABC | K | K2TNSV | s1i1m1c2n2 |  |
| NZ_MVYG01000022 | HP02140 |  |  | Oceania | Australia | W | ABC | K | K2TA1SV | s1i1m1c1n1 |  |
| NZ_MVYF01000023 | HP03054 |  |  | Oceania | Australia | W | ABC | K | K2TNSV | s1i1m1c2n2 |  |
| NZ_MVYD01000002 | HP03218 |  |  | Oceania | Australia | W |  | E | EP | s1i2m2c2n2 |  |
| NZ_MVYC01000017 | HP04041 |  |  | Oceania | Australia | W | ABC | K | K2TNSV | s1i1m1c1n1 |  |
| NZ_MVYB01000012 | HP04042 |  |  | Oceania | Australia | W |  | K | K1TNSV | s2i2m2c2n2 |  |
| NZ_MVYA01000012 | HP04057 |  |  | Oceania | Australia | W |  | E | EP | s2i2m2c2n2 |  |
| NZ_MVXZ01000003 | HP04086 |  |  | Oceania | Australia | W | ABC | E | EP | s1i1m2c2n2 |  |
| NZ_MVXX01000010 | HP05044 |  |  | Oceania | Australia | W |  | E | EP | s2i2m2c2n2 |  |
| NZ_MVXW01000016 | HP06038 |  |  | Oceania | Australia | W | ABC | K | K1GA2V | s1i1m1c1n1 |  |
| NZ_MVXV01000002 | HP06045 |  |  | Oceania | Australia | W | ABC | K | K1GA1SV | s1i1m1c1n1 |  |
| NZ_MVXU01000030 | HP06058 |  |  | Oceania | Australia | W |  | E | EP | s1i1m2c2n2 |  |
| NZ_MVXT01000002 | HP06059 |  |  | Oceania | Australia | W | ABB | K | K1GA1SV | s1i1m1c1n1 |  |
| NZ_MVXS01000019 | HP07019 |  |  | Oceania | Australia | W |  | E | EP | s2i2m2c2n2 |  |
| NZ_MVXR01000011 | HP07036 |  |  | Oceania | Australia | W | AB | K | K1GA2V | s1i1m1c1n1 |  |
| NZ_MVXQ01000015 | HP08031 |  |  | Oceania | Australia | W |  | K | K1GA2SV | s1i1m1c1n1 |  |
| NZ_MVXO01000004 | HP08061 |  |  | Oceania | Australia | W | ABC | K | K1GA2V | s1i1m1c1n1 |  |
| NZ_MVXN01000006 | HP08072 |  |  | Oceania | Australia | W |  | K | K1GA1SV | s1i1m1c1n1 |  |
| NZ_MVXM01000008 | HP08073 |  |  | Oceania | Australia | W |  | E | EP | s2i2m2c2n2 |  |
| NZ_MVXL01000002 | HP08074 |  |  | Oceania | Australia | W |  | E | EP | s2i2m2c2n2 |  |
| NZ_MVXK01000015 | HP09046 |  |  | Oceania | Australia | W | ABC | K | K1GA1SV | s1i1m1c1n1 |  |
| NZ_MVXJ01000003 | HP11004 |  |  | Oceania | Australia | W |  | K | K1GA1SV | s1i1m1c1n1 |  |
| NZ_MVXI01000018 | HP11005 |  |  | Oceania | Australia | W |  | E | EP | s1i1m2c2n2 |  |
| NZ_MVXG01000003 | HP11013 |  |  | Oceania | Australia | W | ABC | K | K1GA1SV | s1i1m1c1n1 |  |
| NZ_MVXF01000021 | HP11020 |  |  | Oceania | Australia | W | ABC | E | EP | s1i2m2c2n2 |  |
| NZ_MVXE01000017 | HP11032 |  |  | Oceania | Australia | W |  | E | EP | s1i2m2c2n2 |  |
| NZ_MVXC01000019 | HP11042 |  |  | Oceania | Australia | W |  | E | EP | s1i1m2c2n2 |  |
| NZ_MVXB01000021 | HP11043 |  |  | Oceania | Australia | W | ABCC | K | K1GA2V | s1i1m1c1n1 |  |
| NZ_MVXA01000018 | HP11049 |  |  | Oceania | Australia | W |  | E | EP | s2i2m2c2n2 |  |
| NZ_MVWZ01000003 | HP11054 |  |  | Oceania | Australia | W |  | E | EP | s2i1m2c2n2 |  |
| NZ_MVWY01000003 | HP11055 |  |  | Oceania | Australia | W | ABC | K | K1GA2SV | s1i1m1c1n1 |  |
| NZ_MVWX01000006 | HP11059 |  |  | Oceania | Australia | W | ABD | K | K1GA2V | s1i1m1c1n1 |  |
| NZ_KB642274 | HP116Bi |  |  | Africa | Gambia | W | ABC | K | K1GA1SV | s1i1m1c2n2 |  |
| NZ_MVWW01000014 | HP12002 |  |  | Oceania | Australia | W |  | E | EP | s2i2m2c2n2 |  |
| NZ_MVWV01000018 | HP12014 |  |  | Oceania | Australia | W |  | E | EP | s2i2m2c2n2 |  |
| NZ_MVWS01000017 | HP12036 |  |  | Oceania | Australia | W | ABD | K | K1GA2V | s1i1m2c2n2 |  |
| NZ_MVWR01000002 | HP12038 |  |  | Oceania | Australia | W |  | E | EP | s2i2m2c2n2 |  |
| NZ_MVWQ01000010 | HP12053 |  |  | Oceania | Australia | W | ABC | K | K1GA1SV | s1i1m1c1n1 |  |
| NZ_MVWP01000007 | HP12054 |  |  | Oceania | Australia | W |  | E | EP | s2i2m2c2n2 |  |
| NZ_MVWO01000024 | HP12059 |  |  | Oceania | Australia | W | AB | E | EP | s1i2m2c2n2 |  |
| NZ_MVWN01000014 | HP12060 |  |  | Oceania | Australia | W | ABCC | K | K2TA1SV | s1i1m2c2n2 |  |
| NZ_MVWM01000010 | HP12064 |  |  | Oceania | Australia | W | ABD | K | K1GA2V | s1i1m1c2n1 |  |
| NZ_MVWL01000020 | HP12068 |  |  | Oceania | Australia | W |  | E | EP | s2i2m2c2n2 |  |
| NZ_MVWK01000018 | HP12069 |  |  | Oceania | Australia | W | ABD | K | K1GA1V | s1i1m1c1n1 |  |
| NZ_MVWJ01000019 | HP12070 |  |  | Oceania | Australia | W |  | E | EP | s1i2m2c2n1 |  |
| NZ_MVWH01000008 | HP12077 |  |  | Oceania | Australia | W |  | E | EP | s2i2m2c2n2 |  |
| NZ_MVWG01000002 | HP12078 |  |  | Oceania | Australia | W | ABD | K | K1GA2SV | s1i1m2c2n1 |  |
| NZ_MVWF01000023 | HP13005 |  |  | Oceania | Australia | W |  | K | K1GA2V | s1i1m1c1n1 |  |
| NZ_MVWE01000004 | HP13007 |  |  | Oceania | Australia | W |  | E | EP | s2i2m2c2n2 |  |
| NZ_MVWD01000016 | HP13009 |  |  | Oceania | Australia | W | ABC | K | K2TNSV | s1i1m1c1n1 |  |
| NZ_MVWC01000012 | HP13011 |  |  | Oceania | Australia | W |  | K | K1GA1SV | s1i1m1c1n1 |  |
| NZ_MVWA01000023 | HP13013 |  |  | Oceania | Australia | W | ABD | K | K1GA2SV | s1i1m2c2n2 |  |
| NZ_MVVZ01000021 | HP13021 |  |  | Oceania | Australia | W |  | E | EP | s2i2m2c2n2 |  |
| NZ_MVVW01000010 | HP13025 |  |  | Oceania | Australia | W |  | E | EP | s1i2m2c2n2 |  |
| NZ_MVVU01000030 | HP13027 |  |  | Oceania | Australia | W |  | E | EA1SV | s1i2m2c2n2 |  |
| NZ_MVVT01000021 | HP13028 |  |  | Oceania | Australia | W | ABC | K | K1GA2SV | s1i1m1c1n1 |  |
| NZ_MVVS01000011 | HP13029 |  |  | Oceania | Australia | W |  | E | EP | s2i2m2c2n2 |  |
| NZ_MVVQ01000024 | HP13033 |  |  | Oceania | Australia | W |  | K | K1TNSV | s1i1m2c2n2 |  |
| NZ_MVVP01000009 | HP13050 |  |  | Oceania | Australia | W |  | E | EP | s2i2m2c2n2 |  |
| NZ_MVVN01000024 | HP13056 |  |  | Oceania | Australia | W |  | E | EP | s2i2m2c2n2 |  |
| NZ_MVVM01000018 | HP13061 |  |  | Oceania | Australia | W | ABC | E | EP | s1i2m2c2n2 |  |
| NZ_MVVL01000013 | HP13063 |  |  | Oceania | Australia | W | ABD | K | K1GA2V | s1i1m2c2n1 |  |
| NZ_MVVK01000024 | HP13064 |  |  | Oceania | Australia | W | ABC | Q | QGA2V | s1i1m2c2n2 |  |
| NZ_MVVJ01000006 | HP13068 |  |  | Oceania | Australia | W | AB | E | EP | s1i2m2c2n2 |  |
| NZ_MVVI01000003 | HP13072 |  |  | Oceania | Australia | W | ABCC | K | K1GA1SV | s1i1m1c1n1 |  |
| NZ_MVVH01000003 | HP14021 |  |  | Oceania | Australia | W |  | E | EP | s2i2m2c2n2 |  |
| NZ_MVVF01000010 | HP14031 |  |  | Oceania | Australia | W | ABD | K | K1GA2SV | s1i1m1c1n1 |  |
| NZ_MVVD01000013 | HP14039 |  |  | Oceania | Australia | W |  | E | EP | s2i2m2c2n2 |  |
| NZ_MVVC01000013 | HP14048 |  |  | Oceania | Australia | W | ABC | K | K1GA2SV | s1i1m2c2n2 |  |
| NZ_MVVA01000003 | HP14051 |  |  | Oceania | Australia | W | ABD | K | K1GA2SV | s1i1m1c1n1 |  |
| NZ_MVUY01000012 | HP14054 |  |  | Oceania | Australia | W |  | E | EP | s2i2m2c2n2 |  |
| NZ_MVUX01000019 | HP14056 |  |  | Oceania | Australia | W | ABD | K | K1GA2SV | s1i1m2c2n2 |  |
| NZ_MVUW01000011 | HP14065 |  |  | Oceania | Australia | W | ABC | E | EP | s1i2m2c2n2 |  |
| NZ_MVUV01000022 | HP14069 |  |  | Oceania | Australia | W | ABD | K | K1GA2V | s1i1m1c1n1 |  |
| NZ_MVUU01000004 | HP15002 |  |  | Oceania | Australia | W | ABD | K | K1GA2V | s1i1m1c2n2 |  |
| NZ_MVUT01000020 | HP15003 |  |  | Oceania | Australia | W | ABD | E | EP | s1i2m2c2n2 |  |
| NZ_MVUS01000003 | HP15004 |  |  | Oceania | Australia | W | ABD | K | K1GA2V | s1i1m2c2n2 |  |
| NZ_MVUR01000022 | HP15005 |  |  | Oceania | Australia | W |  | K | K1GA2V | s1i1m1c1n1 |  |
| NZ_MVUP01000010 | HP15011 |  |  | Oceania | Australia | W | ABD | K | K1GA2V | s1i1m1c1n1 |  |
| NZ_MVUO01000008 | HP15012 |  |  | Oceania | Australia | W | ABC | E | EP | s1i2m2c2n2 |  |
| NZ_MVUM01000022 | HP15015 |  |  | Oceania | Australia | W | AB | K | K1GA2V | s1i1m2c2n2 |  |
| NZ_MVUL01000003 | HP15018 |  |  | Oceania | Australia | W | ABD | K | K1A2V | s1i1m2c2n2 |  |
| NZ_MVUK01000016 | HP15020 |  |  | Oceania | Australia | W | ABD | K | K1GA2V | s1i1m2c2n2 |  |
| NZ_MVUJ01000025 | HP15022 |  |  | Oceania | Australia | W | ABC | E | EP | s1i2m2c2n2 |  |
| NZ_MVUI01000017 | HP15025 |  |  | Oceania | Australia | W | ABD | K | K1GA2V | s1i1m2c2n2 |  |
| NZ_MVUH01000002 | HP15026 |  |  | Oceania | Australia | W | ABC | K | K1GA2SV | s1i1m1c1n1 |  |
| NZ_MVUG01000015 | HP15027 |  |  | Oceania | Australia | W | ABD | K | K1GA2V | s1i1m1c1n1 |  |
| NZ_MVUF01000024 | HP15028 |  |  | Oceania | Australia | W | AC | K | K2TNSV | s1i1m1c1n1 |  |
| NZ_MVUE01000022 | HP15031 |  |  | Oceania | Australia | W | ABD | K | K1GA2V | s1i1m2c2n2 |  |
| NZ_MVUD01000015 | HP15032 |  |  | Oceania | Australia | W | ABD | K | K1GA2V | s1i1m1c1n1 |  |
| NZ_MVUC01000028 | HP15033 |  |  | Oceania | Australia | W |  | E | EP | s2i2m2c2n2 |  |
| NZ_MVUB01000005 | HP15034 |  |  | Oceania | Australia | W | ABC | K | K1GA1SV | s1i1m1c1n1 |  |
| NZ_MVTZ01000026 | HP15036 |  |  | Oceania | Australia | W |  | K | K1GA2V | s1i1m2c2n2 |  |
| NZ_MVTX01000007 | HP15040 |  |  | Oceania | Australia | W | ABD | K | K1GA2V | s1i1m1c1n1 |  |
| NZ_MVTW01000009 | HP15044 |  |  | Oceania | Australia | W | ABD | K | K1GA2V | s1i1m2c2n2 |  |
| NZ_MVTV01000026 | HP15050 |  |  | Oceania | Australia | W | ABC | K | K1GA2SV | s1i1m1c1n1 |  |
| NZ_MVTU01000018 | HP15051 |  |  | Oceania | Australia | W | ABD | K | K1GA2SV | s1i1m1c1n1 |  |
| NZ_MVTT01000019 | HP15054 |  |  | Oceania | Australia | W | ABC | K | K1GA2V | s1i1m1c1n1 |  |
| NZ_MVTR01000007 | HP15059 |  |  | Oceania | Australia | W | ABC | K | K1GA1SV | s1i1m1c1n1 |  |
| NZ_MVTQ01000003 | HP15060 |  |  | Oceania | Australia | W | ABC | E | EP | s1i1m2c2n2 |  |
| NZ_MVTO01000007 | HP16001 |  |  | Oceania | Australia | W | ABC | E | EP | s1i2m2c2n2 |  |
| NZ_MVTN01000012 | HP16004 |  |  | Oceania | Australia | W |  | K | K1GA2SV | s1i1m2c2n2 |  |
| NZ_MVTM01000014 | HP16008 |  |  | Oceania | Australia | W |  | E | EP | s1i2m2c2n2 |  |
| NZ_KB642745 | HP250AFiV |  |  |  |  |  | ABC | K | K1GA1SV | s1i1m1c1n1 |  |
| NZ_KB642432 | HP250AFii |  |  |  |  |  | ABC | K | K1GA1SV | s1i1m1c1n1 |  |
| NZ_KB642581 | HP250AFiii |  |  |  |  |  | ABC | K | K1GA1SV | s1i1m1c1n1 |  |
| NZ_KB636234 | HP250ASi |  |  |  |  |  | ABC | K | K1GA1SV | s1i1m1c1n1 |  |
| NZ_KB636113 | HP250ASii |  |  |  |  |  | ABC | K | K1GA1SV | s1i1m1c1n1 |  |
| NZ_KB636170 | HP250BFi |  |  |  |  |  | ABC | K | K1GA1SV | s1i1m1c1n1 |  |
| NZ_KB636035 | HP250BFiV |  |  |  |  |  | ABC | K | K1GA1SV | s1i1m1c1n1 |  |
| NZ_KB636470 | HP250BFii |  |  |  |  |  | ABC | K | K1GA1SV | s1i1m1c1n1 |  |
| NZ_KB642919 | HP250BFiii |  |  |  |  |  | ABC | K | K1GA1SV | s1i1m1c1n1 |  |
| NZ_KB644294 | HP250BSi |  |  |  |  |  | ABC | K | K1GA1SV | s1i1m1c1n1 |  |
| NZ_KB644225 | HP260AFi |  |  |  |  |  | ABC | K | K1GA1SV | s1i1m1c1n1 |  |
| NZ_KB642504 | HP260AFii |  |  |  |  |  | ABC | K | K1GA1SV | s1i1m1c1n1 |  |
| NZ_KB642719 | HP260ASii |  |  |  |  |  | ABC | K | K1GA1SV | s1i1m1c1n1 |  |
| NZ_KB635918 | HP260BFii |  |  |  |  |  |  | K | K1GP | s2i2m2c2n2 |  |
| NZ_KB635862 | HP260Bi |  |  |  |  |  | ABC | K | K1GA1SV | s1i1m1c1n1 |  |
| NZ_CP034314 | HP42K | NCA | CG | Europe | Belarus | W | ABC | K | K2TNSV | s1i1m1c1n1 |  |
| NZ_MTLF01000013 | HP725g |  |  | Africa | Morocco | W |  | K | K1GA1SV | s1i1m1c1n1 |  |
| NZ_CBRI010000002 | HP87hu |  |  | Europe | Germany | W | ABC | Q | QGA2SV | s1i1m1c1n1 | 23836820 |
| NZ_MVTK01000020 | HP98123 |  |  | Oceania | Australia | W | ABC | K | K1GA2V | s1i1m1c1n1 |  |
| NZ_MVTH01000002 | HP98490 |  |  | Oceania | Australia | W | ABC | E | EP | s1i1m2c2n2 |  |
| NZ_MVTG01000017 | HP99216 |  |  | Oceania | Australia | W | ABC | K | K1GA1SV | s1i1m1c1n1 |  |
| NZ_MVTF01000002 | HP99244 |  |  | Oceania | Australia | W | ABCC | K | K1GA2SV | s1i1m1c1n1 |  |
| NZ_MVTE01000006 | HP99255 |  |  | Oceania | Australia | W |  | E | EP | s2i2m2c2n2 |  |
| NZ_MVTD01000002 | HP99316 |  |  | Oceania | Australia | W | ABC | K | K1GA2SV | s1i1m1c1n1 |  |
| NZ_MVTC01000002 | HP99330 |  |  | Oceania | Australia | W | ABC | K | K1GA1SV | s1i1m1c1n1 |  |
| NZ_MVSX01000021 | HP99647 |  |  | Oceania | Australia | W | ABC | K | K1GA2V | s1i1m1c1n1 |  |
| NZ_MVSW01000003 | HP99648 |  |  | Oceania | Australia | W | ABC | K | K1GA2V | s1i1m1c1n1 |  |
| NZ_MVSV01000014 | HP99689 |  |  | Oceania | Australia | W |  | E | EP | s1i2m2c2n2 |  |
| NC_008086 | HPAG1 | NCA | AG | Europe | Sweden | W | ABBC | Q | QGA2SV | s1i1m1c1n1 | 16788065 |
| NZ_CBKY010000006 | HPARG63 |  |  |  |  |  | ABC | K | K1GA2V | s1i1m1c1n1 |  |
| NZ_CBKZ010000007 | HPARG8G |  |  |  |  |  | ABC | K | K1GA2V | s1i1m2c2n2 |  |
| NZ_MVSU01000020 | HPAS14 |  |  | Oceania | Australia | W |  | Q | QGA2SV2 | s1i1m1c1n1 |  |
| NZ_MVSR01000007 | HPJ013 |  |  | Oceania | Australia | W |  | E | EP | s1i2m2c2n2 |  |
| NZ_MVSO01000002 | HPJ024 |  |  | Oceania | Australia | W | ABC | K | K2TNSV | s1i1m1c1n1 |  |
| NZ_MVSN01000029 | HPJ025 |  |  | Oceania | Australia | W | ABC | K | K2TNSV | s1i1m1c1n1 |  |
| NZ_MVSM01000011 | HPJ040 |  |  | Oceania | Australia | W | ABC | K | K2TNSV | s1i1m1c1n1 |  |
| NZ_MVSL01000008 | HPJ050 |  |  | Oceania | Australia | W | ABC | K | K2TNSV | s1i1m1c1n1 |  |
| NZ_MVRZ01000023 | HPJ165 |  |  | Oceania | Australia | W | ABC | K | K1GA2SV | s1i1m1c1n1 |  |
| NZ_CP023448 | HPJP26 |  |  | America | USA | W | AABD | K | K1GA2V | s1i1m1c1n1 |  |
| NZ_MWQM01000093 | HP_106 |  |  | Africa | Morocco | W |  | K | K1GA1SV | s1i1m1c1n1 |  |
| NZ_MUHJ01000014 | HP_PWs |  |  | Africa | Morocco | W |  | K | K1GA1SV | s1i1m1c1n1 |  |
| NZ_AOTW01000001 | Hp A-11 |  |  | America | USA | W | ABC | K | K2TNSV | s1i1m2c2n2 |  |
| NZ_AKOU01000005 | Hp A-16 | NCA | CG | America | USA | W | ABC | K | K1GA1SV | s1i1m1c1n1 |  |
| NZ_AKOV01000001 | Hp A-26 | NCA | CG | America | USA | W |  | E | EP | s2i2m2c2n2 |  |
| NZ_AKOW01000002 | Hp A-27 | NCA | CG | America | USA | W | ABC | E | EP | s1i1m2c2n2 |  |
| NZ_AKOB01000006 | Hp A-5 | NCA | GU | America | USA | W | ABCC | K | K1GA1SV | s1i1m1c1n1 |  |
| NZ_AKOR01000004 | Hp A-6 | NCA | CG | America | USA | W | ABCC | K | K1GA1SV | s1i1m1c1n1 |  |
| NZ_AKOS01000004 | Hp A-8 | NCA | CG | America | USA | W | ABC | K | K1GA1SV | s1i1m1c1n1 |  |
| NZ_AKOC01000008 | Hp A-9 |  |  | America | USA | W | ABC | E | EP | s1i2m2c2n2 |  |
| NZ_AOTX01000028 | Hp H-1 |  |  | America | USA | W | AC | K | K1GA1SV | s1i1m1c1n1 |  |
| NZ_AKPC01000004 | Hp H-11 | NCA | CG | America | USA | W | AB | E | EP | s1i1m2c2n1 |  |
| NZ_AKOF01000005 | Hp H-16 | NCA | PU | America | USA | W | ABC | K | K1GA1SV | s1i1m1c1n1 |  |
| NZ_AKPD01000011 | Hp H-18 |  |  | America | USA | W | ABC | K | K1GA2V | s1i1m1c1n1 |  |
| NZ_AKPE01000004 | Hp H-19 | NCA | CG | America | USA | W | ABC | K | K2TA1SV | s1i1m1c1n1 |  |
| NZ_AKPF01000003 | Hp H-21 | NCA | CG | America | USA | W |  | K | K1GA1SV | s1i1m1c1n1 |  |
| NZ_AKPG01000006 | Hp H-23 | NCA | CG | America | USA | W | ABCC | K | K1GA1SV | s1i1m1c1n1 |  |
| NZ_AKOG01000006 | Hp H-24 | NCA | GU | America | USA | W | ABCC | K | K1GA1SV | s1i1m1c1n1 |  |
| NZ_AKOH01000005 | Hp H-27 | NCA | GU | America | USA | W |  | E | EP | s2i2m2c2n2 |  |
| NZ_AKOJ01000004 | Hp H-29 |  |  | America | USA | W | ABC | K | K1GA1SV | s1i1m1c1n1 |  |
| NZ_AKOX01000008 | Hp H-3 | NCA | CG | America | USA | W |  | K | K1GA1SV | s1i1m1c1n1 |  |
| NZ_AKOK01000006 | Hp H-30 | NCA | GU | America | USA | W | ABC | K | K1GA1SV | s1i1m1c1n1 |  |
| NZ_AKPH01000005 | Hp H-34 | NCA | CG | America | USA | W |  | K | K2TNSV | s1i1m1c1n1 |  |
| NZ_AKOL01000008 | Hp H-36 |  |  | America | USA | W | ABC | K | K1GA1SV | s1i1m1c1n1 |  |
| NZ_AKOY01000004 | Hp H-4 | NCA | CG | America | USA | W | ABC | K | K1GA1SV | s1i1m1c1n1 |  |
| NZ_AKOM01000004 | Hp H-41 | NCA | DU | America | USA | W | ABC | K | K1GA1SV | s1i1m1c1n1 |  |
| NZ_AKON01000007 | Hp H-42 |  |  | America | USA | W | ABC | K | K1GA1SV | s1i1m1c1n1 |  |
| NZ_AKOO01000004 | Hp H-43 | NCA | DU | America | USA | W |  | E | EP | s2i2m2c2n2 |  |
| NZ_AKOP01000010 | Hp H-44 |  |  | America | USA | W | ABC | K | K1GA1SV | s1i1m1c1n1 |  |
| NZ_AKOQ01000009 | Hp H-45 | NCA | DU | America | USA | W | ABCC | K | K1GA2V | s1i1m1c1n1 |  |
| NZ_AKQF01000006 | Hp H-5b |  |  | America | USA | W | ABCC | K | K1GA1SV | s1i1m1c1n1 |  |
| NZ_AKOZ01000004 | Hp H-6 | NCA | CG | America | USA | W | ACCC | K | K1GA1SV | s1i1m2c2n2 |  |
| NZ_AKPA01000005 | Hp H-9 | NCA | CG | America | USA | W | ABC | K | K1GA2SV | s1i1m1c1n1 |  |
| NZ_AKPI01000003 | Hp P-1 | NCA | CG | America | USA | W | ABC | K | K2TNSV | s1i1m1c1n1 |  |
| NZ_AKPN01000006 | Hp P-11 | NCA | CG | America | USA | W | ABC | K | K1GA1SV | s1i1m1c1n1 |  |
| AKPO01000006 | Hp P-13 | NCA | CG | America | USA | W | ABC | K | K1GA1SV | s1i1m1c1n1 |  |
| NZ_AKQI01000006 | Hp P-13b |  |  | America | USA | W | ABC | K | K1GA1SV | s1i1m1c1n1 |  |
| NZ_AKQJ01000003 | Hp P-15b |  |  | America | USA | W |  | E | EP | s2i2m2c2n2 |  |
| NZ_AKQA01000006 | Hp P-1b |  |  | America | USA | W | ABC | K | K2TNSV | s1i1m1c1n1 |  |
| NZ_AKPJ01000001 | Hp P-2 | NCA | CG | America | USA | W | ABC | K | K1GA2SV | s1i1m1c1n1 |  |
| NZ_AKPR01000002 | Hp P-23 | NCA | CG | America | USA | W |  | E | EP | s2i2m2c2n2 |  |
| NZ_AKQK01000004 | Hp P-25c |  |  | America | USA | W | ABC | K | K2TNSV | s1i1m1c1n1 |  |
| NZ_AKQL01000004 | Hp P-25d |  |  | America | USA | W | ABC | K | K2TNSV | s1i1m1c1n1 |  |
| NZ_AKPT01000006 | Hp P-26 | NCA | CG | America | USA | W | ABC | K | K1GA1SV | s1i1m1c1n1 |  |
| NZ_AKPK01000005 | Hp P-3 | NCA | CG | America | USA | W | ABC | K | K1GA1SV | s1i1m1c1n1 |  |
| NZ_AKPU01000004 | Hp P-30 | NCA | CG | America | USA | W |  | E | EP | s2i2m2c2n2 |  |
| NZ_AKQC01000004 | Hp P-3b |  |  | America | USA | W | ABC | K | K1GA1SV | s1i1m1c1n1 |  |
| NZ_AKPL01000004 | Hp P-4 | NCA | CG | America | USA | W | ABC | K | K1GA1SV | s1i1m1c1n1 |  |
| NZ_AKQD01000004 | Hp P-4c |  |  | America | USA | W | ABC | K | K1GA1SV | s1i1m1c1n1 |  |
| NZ_AKQE01000005 | Hp P-4d |  |  | America | USA | W | ABC | K | K1GA1SV | s1i1m1c1n1 |  |
| NZ_AKPW01000005 | Hp P-62 | NCA | CG | America | USA | W | ABC | K | K2TNSV | s1i1m1c1n1 |  |
| NZ_CP034071 | Hpbs1 | NCA | GU | Asia | China | E | ABD | K | K1GA2V | s1i1m1c1n1 |  |
| NC_017372 | India7 | NCA | PU | Asia | India | W | ABCC | E | EP | s1i2m2c2n2 |  |
| NZ_AZBQ01000032 | Iso6 |  |  | Europe | Germany | W | ABB | E | EP | s2i2m2c2n2 |  |
| NZ_AZBS01000027 | Iso7 |  |  | Europe | Germany | W |  | E | EP | s2i2m2c2n2 |  |
| NZ_CP007603 | J166 |  |  | America | USA | W | ABC | K | K1GA1SV | s1i1m1c1n1 | 9789049 |
| NZ_CP011330 | J99 | NCA | DU | America | USA | W | AC | K | K1GA1SV | s1i1m1c1n1 | 9923682 |
| NZ_QEGK01000015 | JDX15 | NCA | CG | Europe | Spain | W | ABC | E | EP | s1i1m2c2n2 |  |
| NZ_QEGJ01000012 | JGF25 |  |  | Europe | Spain | W |  | E | EP | s2i2m2c2n2 |  |
| NZ_QEGI01000016 | JMM43 |  |  | Europe | Spain | W |  | E | EP | s2i2m2c2n2 |  |
| NZ_QEGH01000020 | JSS185-B120 | NCA | CG | Europe | Spain | W | ABC | K | K1GA2SV | s1i1m2c2n2 |  |
| NZ_CP011486 | K26A1 |  |  | Africa | Angola | W |  | E | EP | s2i2m2c2n2 |  |
| NZ_PHMW01000021 | KH1 | NCA | IM | Asia | India | W | ABC | K | K1GA2SV | s1i1m2c2n2 |  |
| NZ_PHMN01000009 | KH11 | NCA | CG | Asia | India | W | ABC | K | K1GA2SV | s1i1m1c1n1 |  |
| NZ_PHMM01000006 | KH12 | NCA | CG | Asia | India | W |  | E | EP | s2i2m2c2n2 |  |
| NZ_PHML01000027 | KH13 | NCA | CG | Asia | India | W |  | E | EP | s2i2m2c2n2 |  |
| NZ_PHMK01000014 | KH14 | NCA | CG | Asia | India | W | ABCC | K | K1GA3SV | s1i1m1c1n1 |  |
| NZ_PHMJ01000009 | KH15 | NCA | CG | Asia | India | W | ABC | K | K1GA2SV | s1i1m1c1n1 |  |
| NZ_PHMI01000024 | KH16 | NCA | CG | Asia | India | W |  | E | EP | s2i2m2c2n2 |  |
| NZ_PHMH01000021 | KH17 | NCA | CG | Asia | India | W | ABCC | K | K1GA2SV | s1i1m1c1n1 |  |
| NZ_PHMG01000026 | KH18 | NCA | CG | Asia | India | W | ABC | K | K1GA2SV | s1i1m1c1n1 |  |
| NZ_PHMV01000021 | KH2 | NCA | GU | Asia | India | W |  | E | EP | s2i2m2c2n2 |  |
| NZ_PHME01000012 | KH20 | NCA | DU | Asia | India | W | ABC | K | K1GA2SV | s1i1m1c1n1 |  |
| NZ_PHMD01000012 | KH21 | NCA | DU | Asia | India | W | ABC | E | EP | s1i2m2c2n2 |  |
| NZ_PHMB01000011 | KH23 | NCA | DU | Asia | India | W | ABC | K | K1GA2SV | s1i1m1c1n1 |  |
| NZ_PHMA01000021 | KH25 | NCA | CG | Asia | India | W | ABC | K | K1GA2SV | s1i1m1c1n1 |  |
| NZ_PHLZ01000033 | KH26 | NCA | CG | Asia | India | W | ABC | K | K1GA2SV | s1i1m2c2n2 |  |
| NZ_PHLY01000016 | KH27 | NCA | CG | Asia | India | W | ABC | K | K1GA2V | s1i1m1c1n1 |  |
| NZ_PHLX01000007 | KH28 | NCA | CG | Asia | India | W | ABC | Q | QGA2SV | s1i1m1c1n1 |  |
| NZ_PHLW01000004 | KH29 | NCA | CG | Asia | India | W |  | E | EP | s2i2m2c2n2 |  |
| NZ_PHMU01000023 | KH3 | NCA | CG | Asia | India | W |  | E | EP | s2i2m2c2n2 |  |
| NZ_PHLV01000002 | KH30 | NCA | CG | Asia | India | W | ABC | E | EP | s1i2m2c2n2 |  |
| NZ_PHLU01000022 | KH31 | NCA | CG | Asia | India | W | ABCC | K | K1GA2SV | s1i1m1c1n1 |  |
| NZ_PHLT01000018 | KH32 | NCA | IM | Asia | India | W | ABC | K | K1GA2SV | s1i1m1c1n1 |  |
| NZ_PHLS01000003 | KH33 | NCA | DU | Asia | India | W | ABC | K | K1GA2SV | s1i1m2c2n2 |  |
| NZ_PHLR01000011 | KH34 | NCA | CG | Asia | India | W | ABC | K | K1GA2SV | s1i1m1c1n1 |  |
| NZ_PHLQ01000010 | KH35 | NCA | CG | Asia | India | W | AB | E | EP | s1i1m2c2n2 |  |
| NZ_PHLP01000005 | KH36 | NCA | CG | Asia | India | W | ABCC | K | K1GA2V | s1i1m2c2n2 |  |
| NZ_PHLO01000022 | KH37 | NCA | DU | Asia | India | W | ABCC | K | K1GA1SV | s1i1m1c1n1 |  |
| NZ_PHLN01000005 | KH38 | NCA | IM | Asia | India | W |  | E | EP | s2i2m2c2n2 |  |
| NZ_PHLM01000010 | KH39 | NCA | DU | Asia | India | W | ABB | K | K1GA2SV | s1i1m1c1n1 |  |
| NZ_PHMT01000006 | KH4 | NCA | DU | Asia | India | W | ABC | K | K1GA2SV | s1i1m2c2n2 |  |
| NZ_PHLL01000021 | KH40 | NCA | CG | Asia | India | W |  | E | EP | s2i2m2c2n2 |  |
| NZ_PHLK01000016 | KH41 | NCA | CG | Asia | India | W | ABC | E | EP | s1i2m2c2n2 |  |
| NZ_PHLJ01000013 | KH43 | NCA | CG | Asia | India | W | ABCC | K | K1GA2SV | s1i1m1c1n1 |  |
| NZ_PHLI01000009 | KH44 | NCA | CG | Asia | India | W | ABC | K | K1GA2SV | s1i1m2c2n2 |  |
| NZ_PHLH01000020 | KH45 | NCA | CG | Asia | India | W | ABC | K | K1GA2SV | s1i1m1c1n1 |  |
| NZ_PHMS01000025 | KH6 | NCA | CG | Asia | India | W |  | E | EP | s2i2m2c2n2 |  |
| NZ_PHMR01000018 | KH7 | NCA | CG | Asia | India | W | ABC | K | K1GA2SV | s1i1m1c2n1 |  |
| NZ_PHMQ01000020 | KH8 | NCA | GU | Asia | India | W |  | E | EP | s2i2m2c2n2 |  |
| NZ_PHMP01000010 | KH9 | NCA | DU | Asia | India | W | ABC | K | K1GA1SV | s1i1m1c1n1 |  |
| LC420372 | Kolaka56 |  |  | Asia | Indonesia | W |  | K | K1GA2 | s1i1m1c1n2 |  |
| LC420367 | Kolaka79 |  |  | Asia | Indonesia | W |  | E | E | s1i2m1c1n2 |  |
| LC420379 | Kolaka82 |  |  | Asia | Indonesia | W |  | K | K1GA2SV | s1i1m1c1n1 |  |
| LC420368 | Kolaka94 |  |  | Asia | Indonesia | W |  | E | E | s1i2m1c1n2 |  |
| LC420369 | Kolaka96 |  |  | Asia | Indonesia | W |  | E | E | s1i2m1c1n2 |  |
| LC420370 | Kolaka98 |  |  | Asia | Indonesia | W |  | E | E | s1i2m1c1n2 |  |
| LC420371 | Kolaka99 |  |  | Asia | Indonesia | W |  | K | K1GA2SV | s1i1m1c1n2 |  |
| GQ331984 | L1 |  |  | Asia | India | W |  | K | K1GA2V | s1i1m1c1n1 |  |
| NZ_CP011482 | L7 |  |  | Asia | India | W | ABBD | K | K1GA2V | s1i1m1c2n1 | 20808891 |
| GQ331983 | L8 | NCA | CG | Asia | India | W |  | K | K1GA1SV | s1i1m1c1n1 | 15814963 |
| NC_017362 | Lithuania75 |  |  | Europe | Lithuania | W | ABC | K | K1GA2V | s1i1m1c1n1 |  |
| NZ_MIKS01000003 | MC2006-52 | CA | CA | America | Mexico | W | AB | K | K2TNSV | s1i1m1c1n1 | 28293542 |
| NZ_MIKT01000123 | MC2011-145 | CA | CA | America | Mexico | W |  | K | K2TNSV | s1i1m1c1n1 | 28293542 |
| NZ_MIKU01000059 | MCms1054 | CA | CA | America | Mexico | W | ABC | K | K1A2SV | s1i1m1c1n1 | 28293542 |
| NZ_MIKV01000030 | MCms1055 | CA | CA | America | Mexico | W | ABC | Q | QGA2SV | s1i1m1c1n1 | 28293542 |
| NZ_MIKW01000056 | MCms1063 | CA | CA | America | Mexico | W | ABC | K | K2TNSV | s1i1m1c1n1 | 28293542 |
| NZ_MIKX01000057 | MCms1078 | CA | CA | America | Mexico | W | ABC | K | K1GA2V | s1i1m1c1n1 | 28293542 |
| NZ_MIKY01000036 | MCms1080 | CA | CA | America | Mexico | W | ABC | K | K2TNSV | s1i1m1c1n1 | 28293542 |
| NZ_MIKZ01000063 | MCms931 | CA | CA | America | Mexico | W | ABC | K | K1GA1SV | s1i1m1c1n1 | 28293542 |
| NZ_MILA01000026 | MG2003-107 | NCA | CG | America | Mexico | W | ABC | E | EP | s1i2m2c2n2 | 28293542 |
| NZ_MILD01000081 | MG2005-98 | NCA | CG | America | Mexico | W | ABC | K | K2TNSV | s1i1m1c1n1 | 28293542 |
| NZ_MILF01000056 | MG2006-407 | NCA | CG | America | Mexico | W | ABC | K | K2TNSV | s1i1m1c1n1 | 28293542 |
| NZ_MILG01000056 | MG2006-479 | NCA | CG | America | Mexico | W | ABC | K | K1GA1SV | s1i1m1c1n1 | 28293542 |
| NZ_MILH01000307 | MG2011-41 | NCA | CG | America | Mexico | W |  | K | K1GA2SV | s1i1m1c1n1 | 28293542 |
| NZ_MILI01000029 | MGms13 | NCA | CG | America | Mexico | W |  | K | K1GA1SV | s1i1m1c1n1 | 28293542 |
| NZ_MILJ01000079 | MGms15 | NCA | CG | America | Mexico | W | ABC | K | K2TNSV | s1i1m1c1n1 | 28293542 |
| NZ_MILK01000006 | MGms167 | NCA | CG | America | Mexico | W | ABC | K | K1GA2SV | s1i1m1c1n1 | 28293542 |
| NZ_MILL01000035 | MGms176 | NCA | CG | America | Mexico | W |  | K | K2TNSV | s1i1m1c1n1 | 28293542 |
| NZ_MILM01000098 | MGms2 | NCA | CG | America | Mexico | W | ABC | Q | QGA2SV | s1i1m2c2n2 | 28293542 |
| NZ_MILN01000079 | MGms203 | NCA | CG | America | Mexico | W |  | K | K1GA1SV | s1i1m1c1n1 | 28293542 |
| NZ_MILO01000042 | MGms23 | NCA | CG | America | Mexico | W | ABC | K | K1GA1SV | s1i1m1c1n1 | 28293542 |
| NZ_MILP01000107 | MGms44 | NCA | CG | America | Mexico | W | ABC | K | K1GA2SV | s1i1m1c1n1 | 28293542 |
| NZ_AP017356 | MKF10 |  |  | Asia | Japan | E | ABD | K | K1GA2SV | s1i1m1c1n1 | 27604221 |
| NZ_AP017357 | MKF3 |  |  | Asia | Japan | E | ABABD | K | K1GA2V | s1i1m1c1n1 | 27604221 |
| NZ_AP017358 | MKF8 |  |  | Asia | Japan | E | ABD | K | K1GA2V | s1i1m1c1n1 | 27604221 |
| NZ_AP017359 | MKM1 |  |  | Asia | Japan | E | ABD | K | K1GA2SV | s1i1m1c1n1 | 27604221 |
| NZ_AP017360 | MKM5 |  |  | Asia | Japan | E | AABD | K | K1GA2V | s1i1m1c1n1 | 27604221 |
| NZ_AP017362 | MKM6 |  |  | Asia | Japan | E | ABD | K | K1GA2SV | s1i1m1c1n1 | 27604221 |
| NZ_AP014710 | ML1 | CA | MALT | Asia | China | E | ABD | K | K1GA2SV | s1i1m2c2n2 | 25908123 |
| NZ_AP014712 | ML3 | CA | MALT | Asia | China | E |  | K | K1GA2V | s1i1m1c1n1 | 25908123 |
| NZ_MIKR01000134 | MM2003-103 | NCA | IM | America | Mexico | W | ABCBC | K | K1GA1SV | s1i1m1c1n1 | 28293542 |
| NZ_MILQ01000038 | MM2004-20 | NCA | IM | America | Mexico | W | ABC | K | K1GA1SV | s1i1m1c1n1 | 28293542 |
| NZ_MILT01000053 | MM2005-126 | NCA | IM | America | Mexico | W | ABC | K | K1GA2SV | s1i1m1c1n1 | 28293542 |
| NZ_MILR01000028 | MM2005-72 | NCA | IM | America | Mexico | W | ABC | Q | QGA2SV | s1i1m1c1n1 | 28293542 |
| NZ_MILS01000011 | MM2006-103 | NCA | IM | America | Mexico | W | ABC | K | K2TNSV | s1i1m1c1n1 | 28293542 |
| NZ_MILU01000102 | MM2006-480 | NCA | IM | America | Mexico | W | ABC | K | K1GA2V | s1i1m1c1n1 | 28293542 |
| NZ_MILW01000012 | MM2012-26 | NCA | IM | America | Mexico | W |  | K | K2TA1SV | s1i1m1c1n1 | 28293542 |
| NZ_QEGG01000021 | MMV242 |  |  | Europe | Spain | W | ABC | K | K2TNSV | s1i1m2c2n2 |  |
| NZ_MILX01000059 | MU2003-84 | NCA | IM | America | Mexico | W | AB | K | K1GA2SV | s1i1m1c1n1 | 28293542 |
| NZ_MILY01000017 | MU2004-2 | NCA | IM | America | Mexico | W | ABC | K | K1GA2SV | s1i1m1c1n1 | 28293542 |
| GQ331977 | MZ1 | NCA | PU | Asia | India | W | ABC | K | K1GA2V | s1i1m1c1n1 |  |
| GQ331979 | MZ12 |  |  | Asia | India | W | ABC | K | K1GA2V | s1i1m1c1n1 |  |
| GQ331978 | MZ4 |  |  | Asia | India | W | ABC | K | K1GA2SV | s1i1m1c1n1 |  |
| NZ_JRAC01000009 | Manado-1 | NCA | VOL | Asia | Indonesia | W | ABD | K | K1GA2V | s1i1m1c1n1 | 25299127 |
| 4484 | Mandalay03 |  |  | Asia | Myanmar | W |  | K | K1GA2V | s1i1m1c1n1 |  |
| 4482 | Mandalay30 |  |  | Asia | Myanmar | W |  | K | K1GA2V | s1i1m1c2n2 |  |
| LC420354 | Medan36 |  |  | Asia | Indonesia | W |  | K | K1GA2SV | s1i1m1c2n2 |  |
| LC420356 | Medan37 |  |  | Asia | Indonesia | W |  | K | K1GA2V | s1i1m1c1n1 |  |
| LC420358 | Medan40 |  |  | Asia | Indonesia | W |  | K | K1GA2V | s1i1m1c1n1 |  |
| LC420357 | Medan49 |  |  | Asia | Indonesia | W |  | K | K1GA2SV | s1i1m1c1n1 |  |
| LC420355 | Medan50 |  |  | Asia | Indonesia | W |  | K | K1GA2SV | s1i1m1c1n1 |  |
| LC420359 | Medan56 |  |  | Asia | Indonesia | W |  | K | K1GA2SV | s1i1m1c1n1 |  |
| LC420360 | Medan67 |  |  | Asia | Indonesia | W |  | K | K1GA2V | s1i1m1c1n1 |  |
| LC420361 | Medan68 |  |  | Asia | Indonesia | W |  | K | K1GA2V | s1i1m1c2n1 |  |
| LC420362 | Medan73 |  |  | Asia | Indonesia | W |  | K | K1GA2SV | s1i1m1c1n1 |  |
| LC420363 | Medan75 |  |  | Asia | Indonesia | W |  | K | K1GA2SV | s1i1m1c1n1 |  |
| LC420373 | Merauke12 |  |  | Asia | Indonesia | W |  | E | EA2SV | s1i1m1c1n1 |  |
| LC420374 | Merauke21 |  |  | Asia | Indonesia | W |  | E | EA2SV | s1i1m1c1n1 |  |
| LC420375 | Merauke27 |  |  | Asia | Indonesia | W |  | E | EA2SV | s1i1m1c1n1 |  |
| LC420376 | Merauke3 |  |  | Asia | Indonesia | W |  | E | EA2SV | s1i1m1c1n1 |  |
| LC420366 | Merauke37 |  |  | Asia | Indonesia | W |  | E | EA2SV | s1i1m1c1n1 |  |
| LC420377 | Merauke5 |  |  | Asia | Indonesia | W |  | E | EA2SV | s1i1m1c1n1 |  |
| LC420378 | Merauke7 |  |  | Asia | Indonesia | W |  | E | EA2SV | s1i1m1c1n1 |  |
| LC420380 | Merauke8 |  |  | Asia | Indonesia | W |  | E | EA2SV | s1i1m1c1n1 |  |
| 4478 | Myanmar51 |  |  | Asia | Myanmar | W |  | K | K1GA2V | s1i1m1c1n1 |  |
| 4477 | Myanmar52 |  |  | Asia | Myanmar | W |  | K | K1GA2V | s1i1m1c1n1 |  |
| 4476 | Myanmar66 |  |  | Asia | Myanmar | W |  | K | K1GA2V | s1i1m1c1n1 |  |
| NZ_AJFA02000020 | NAB47 | NCA | DU | Asia | India | W |  | K | K1GA1SV | s1i1m1c1n1 | 23045484 |
| NZ_AJGJ02000042 | NAD1 | NCA | DU | Asia | India | W | ABC | K | K1GA2V | s1i1m1c1n1 | 23045484 |
| NZ_AONJ01000011 | NAK7 |  |  | Asia | India | W | ABC | K | K1GA2V | s1i1m1c2n2 |  |
| NZ_LS483488 | NCTC 11637 | NCA | CG | Oceania | Australia | W | ABCCC | K | K2TNSV | s1i1m1c1n1 |  |
| HPU07145 | NCTC 11638 |  |  |  |  |  |  | K | K1GA2SV | s1i1m1c1n1 |  |
| NZ_UGJP01000002 | NCTC13094 |  |  |  |  |  | ABCC | K | K1GA2SV | s1i1m1c1n1 |  |
| NZ_UGHN01000001 | NCTC13207 |  |  |  |  |  |  | E | EP | s2i2m2c2n2 |  |
| NZ_UGHQ01000001 | NCTC13338 |  |  |  |  |  | ABC | K | K1GA1SV | s1i1m1c1n1 |  |
| NZ_LR134517 | NCTC13345 |  |  | Africa | Nigeria | W | ABC | K | K1GA1SV | s1i1m1c1n1 |  |
| 4475 | NP04 |  |  | Asia | Nepal | W |  | K | K1GA2SV | s1i1m1c1n1 |  |
| 4461 | NP05 |  |  | Asia | Nepal | W |  | E | EP | s1i2m2c2n2 |  |
| 4474 | NP05_105 |  |  |  |  |  |  | K | K1GA2V | s1i1m1c1n1 |  |
| 4473 | NP05_107 |  |  |  |  |  |  | K | K1GA2SV | s1i1m1c1n1 |  |
| 4472 | NP05_112 |  |  |  |  |  |  | K | K1GA2SV | s1i1m1c2n2 |  |
| 4471 | NP05_121 |  |  |  |  |  |  | K | K1GA2SV | s1i1m1c1n1 |  |
| 4467 | NP05_250 |  |  |  |  |  |  | K | K1GA2SV | s1i1m1c1n1 |  |
| 4463 | NP05_278 |  |  |  |  |  |  | K | K1GA2SV | s1i1m1c2n1 |  |
| NZ_CADE01000007 | NQ315 |  |  | America | Colombia | W |  | K | K1GA1SV | s1i1m1c1n1 | 21383187 |
| NZ_CADI01000010 | NQ392 |  |  | America | Colombia | W |  | K | K2TNSV | s1i1m1c1n1 | 21383187 |
| NZ_AKNU01000004 | NQ4099 | NCA | FD | America | Colombia | W | ABC | K | K2TA1SV | s1i1m1c1n1 | 23661595 |
| NZ_AKNS01000005 | NQ4200 | NCA | IM | America | Colombia | W | ABC | K | K1GANSV | s1i1m1c1n1 | 23661595 |
| NZ_AKNR01000007 | NQ4216 | NCA | FD | America | Colombia | W | ABCC | K | K2TNSV | s1i1m1c1n1 | 23661595 |
| NZ_AKNT01000004 | NQ4228 | NCA | IM | America | Colombia | W | ABC | Q | QGA2SV | s1i1m1c1n1 | 23661595 |
| NZ_AP014523 | NY40 |  |  | Asia | Japan | E | ABC | Q | QGA2SV | s1i1m1c1n1 |  |
| LC420353 | Nias9 |  |  | Asia | Indonesia | W |  | E | EA2SV | s1i1m1c1n1 |  |
| 4528 | Nic01_A | NCA | AG | America | Nicaragua | W |  | K | K2TNSV | s1i1m1c1n1 |  |
| 4532 | Nic04_A | NCA | AG | America | Nicaragua | W |  | K | K3TNSV | s1i1m1c1n1 |  |
| 4534 | Nic05_A | NCA | AG | America | Nicaragua | W |  | Q | QGA2SV | s1i1m1c1n1 |  |
| 4536 | Nic06_A | NCA | AG | America | Nicaragua | W |  | Q | QGA2SV | s1i1m1c1n1 |  |
| 4538 | Nic07_A | NCA | AG | America | Nicaragua | W |  | K | K1GA1SV | s1i1m1c1n1 |  |
| 4540 | Nic08_C2 | NCA | CG | America | Nicaragua | W |  | E | EP | s2i2m2c2n2 |  |
| 4542 | Nic09_A | NCA | AG | America | Nicaragua | W |  | E | EP | s2i2m2c2n2 |  |
| 4546 | Nic11_A | NCA | AG | America | Nicaragua | W |  | K | K2TNSV | s1i1m1c2n2 |  |
| 4548 | Nic12_A | NCA | AG | America | Nicaragua | W |  | K | K2TNSV | s1i1m1c1n1 |  |
| 4552 | Nic14_A | NCA | CG | America | Nicaragua | W |  | K | K2TNSV | s1i1m1c1n1 |  |
| 4553 | Nic14_C | NCA | CG | America | Nicaragua | W |  | E | EP | s2i2m2c2n2 |  |
| 4554 | Nic15_A | NCA | CG | America | Nicaragua | W |  | E | EP | s2i2m2c2n2 |  |
| 4556 | Nic16_A | NCA | CG | America | Nicaragua | W |  | K | K2TNSV | s1i1m1c1n1 |  |
| 4562 | Nic19_A | NCA | IM | America | Nicaragua | W |  | K | K2TA1SV | s1i1m1c1n1 |  |
| 4564 | Nic20_A | NCA | IM | America | Nicaragua | W |  | E | EP | s2i2m2c2n2 |  |
| 4565 | Nic20_C | NCA | IM | America | Nicaragua | W |  | K | K1GA1SV | s1i1m1c1n1 |  |
| 4566 | Nic21_C | NCA | IM | America | Nicaragua | W |  | K | K1GA1SV | s1i1m1c1n1 |  |
| 6288 | Nic23_A | NCA | IM | America | Nicaragua | W |  | K | K1GA2SV | s1i1m1c1n1 |  |
| 6289 | Nic24_A | NCA | CG | America | Nicaragua | W |  | K | K2TNSV | s1i1m1c1n1 |  |
| 6290 | Nic25_A | NCA | IM | America | Nicaragua | W |  | Q | QGA2SV | s1i1m1c1n1 |  |
| 6291 | Nic26_A | NCA | CG | America | Nicaragua | W |  | K | K1GA1SV | s1i1m1c1n1 |  |
| 6292 | Nic27_A | NCA | CG | America | Nicaragua | W |  | K | K2TNSV | s1i1m1c1n1 |  |
| 6293 | Nic28_A | NCA | CG | America | Nicaragua | W |  | K | K2TNSV | s1i1m1c1n1 |  |
| 6283 | Nic29_A | NCA | CG | America | Nicaragua | W |  | K | K1GA1SV | s1i1m1c1n1 |  |
| 6284 | Nic30_A | NCA | CG | America | Nicaragua | W |  | K | K1GA2SV | s1i1m1c1n1 |  |
| AB190967 | OK101 | NCA | CG | Asia | Japan | E | ABD | K | K1GA2SV | s1i1m1c1n1 | 16081930 |
| AB190968 | OK107 | NCA | CG | Asia | Japan | E | ABCCC | E | EP | s1i2m2c2n2 | 16081930 |
| AB190969 | OK109 | NCA | CG | Asia | Japan | E | ABD | K | K1GA2V | s1i1m1c1n1 | 16081930 |
| AB190970 | OK111 | NCA | CG | Asia | Japan | E | ABCC | K | K1GA2SV | s1i1m1c1n1 | 16081930 |
| AB190971 | OK118 | NCA | CG | Asia | Japan | E | ABD | K | K1GA2V | s1i1m1c1n1 | 16081930 |
| AB190972 | OK129 | NCA | CG | Asia | Japan | E | ABD | K | K1GA2V | s1i1m2c2n2 | 16081930 |
| AB190973 | OK130 | NCA | DU | Asia | Japan | E | ABC | E | EP | s1i2m2c2n1 | 16081930 |
| AB190974 | OK139 | NCA | CG | Asia | Japan | E | ABC | E | EP | s1i2m2c2n2 | 16081930 |
| AB190975 | OK144 | NCA | DU | Asia | Japan | E | ABC | E | EP | s1i2m2c2n2 | 16081930 |
| AB190976 | OK155 | NCA | CG | Asia | Japan | E | ABC | K | K1GA1V | s2i2m2c2n2 | 16081930 |
| AB190977 | OK158 | NCA | DU | Asia | Japan | E | ABD | K | K1GA2SV | s1i1m1c1n1 | 16081930 |
| AB190978 | OK159 | NCA | CG | Asia | Japan | E | ABD | K | K1GA2SV | s1i1m1c1n1 | 16081930 |
| AB190979 | OK160 | NCA | CG | Asia | Japan | E | ABC | E | EP | s1i2m2c2n2 | 16081930 |
| AB190980 | OK179 | NCA | DU | Asia | Japan | E |  | E | EP | s1i2m2c2n2 | 16081930 |
| AB190981 | OK180 | NCA | DU | Asia | Japan | E | ABC | E | EP | s1i2m2c2n2 | 16081930 |
| AB190982 | OK181 | NCA | AG | Asia | Japan | E | AB | E | EP | s1i2m2c2n2 | 16081930 |
| AB190983 | OK185 | NCA | DU | Asia | Japan | E | ABC | E | EP | s1i2m2c2n2 | 16081930 |
| AB190984 | OK187 | NCA | DU | Asia | Japan | E | ABC | E | EP | s1i2m2c2n2 | 16081930 |
| AB190985 | OK194 | NCA | CG | Asia | Japan | E | ABD | K | K1GA2SV | s1i1m1c1n1 | 16081930 |
| AB190986 | OK204 | NCA | DU | Asia | Japan | E | ABD | E | EP | s1i2m2c2n2 | 16081930 |
| AB190987 | OK205 | NCA | DU | Asia | Japan | E |  | E | EP | s1i2m2c2n2 | 16081930 |
| AB190988 | OK210 | NCA | DU | Asia | Japan | E | ABC | K | K1GA2V | s1i1m2c2n2 | 16081930 |
| NC_020509 | OK310 | CA | CA | Asia | Japan | E | ABCC | K | K1GA2V | s1i1m1c1n1 | 23505045 |
| NZ_MVFB01000005 | OND1954 |  |  | Oceania | Australia | W |  | E | EP | s1i2m2c2n2 |  |
| NC_011498 | P12 | NCA | DU | Europe | Germany | W | ABCC | K | K1GA2SV | s1i1m1c1n1 | 23782461 |
| GQ331974 | PG218 |  |  | Asia | India | W |  | K | K1GA2SV | s1i1m1c2n2 |  |
| GQ331975 | PG225 |  |  | Asia | India | W |  | K | K1GA3SV | s1i1m1c1n1 |  |
| GQ331976 | PG227 |  |  | Asia | India | W |  | K | K1GA3SV | s1i1m1c1n1 | 22879937 |
| NZ_AZBR01000003 | PMSS1 | NCA | DU | Europe | Switzerland | W | ABC | E | EP | s2i2m2c2n2 |  |
| NZ_CP011487 | PNG84A |  |  | Oceania | Papua New Guinea | W |  | K | K1GA2V | s1i1m1c1n1 |  |
| NZ_MTWJ01000026 | PZ5005_3A3 | NCA | CG | America | Colombia | W | ABC | K | K1GA1SV | s1i1m1c1n1 |  |
| NZ_MTWK01000004 | PZ5006_3A3 | NCA | CG | America | Colombia | W | ABC | K | K1GA1SV | s1i1m1c1n1 |  |
| NZ_MSYO01000016 | PZ5009-3A2 | NCA | CG | America | Colombia | W | ABC | K | K1GA1SV | s1i1m1c1n1 |  |
| NZ_MTWL01000002 | PZ5016_3A3 | NCA | AG | America | Colombia | W | ABC | K | K2TNSV | s1i1m1c1n1 |  |
| NZ_MTWM01000012 | PZ5019_3A3 | NCA | IM | America | Colombia | W | ABC | Q | QGA2SV | s1i1m1c1n1 |  |
| NZ_MTWN01000019 | PZ5033_3A2 | NCA | IM | America | Colombia | W |  | Q | QGA2SV | s1i1m1c1n1 |  |
| NZ_ASYU01000056 | PZ5056 | NCA | IM | America | Colombia | W | ABBCC | Q | QGA2SV | s1i1m1c1n1 | 24051318 |
| NZ_ASYV01000171 | PZ5080 | NCA | IM | America | Colombia | W | ABC | Q | QA2SV | s1i1m1c1n1 | 24051318 |
| LC420364 | Padang42 |  |  | Asia | Indonesia | W |  | K | K1GA2V | s1i1m1c1n1 |  |
| NC_017742 | PeCan18 | CA | CA | America | Peru | W | ABD | K | K1GA2SV | s1i1m1c1n1 |  |
| NC_014555 | PeCan4 | CA | CA | America | Peru | W | ABBC | K | K2TNSV | s1i1m1c1n1 |  |
| NC_017378 | Puno120 | NCA | CG | America | Peru | W | ABDC | K | K2TNSV | s1i1m1c1n1 |  |
| NC_017379 | Puno135 | NCA | CG | America | Peru | W | ABCC | K | K1TA2SV | s1i1m1c1n1 |  |
| NZ_AMOQ01000004 | R018c |  |  | America | Canada | W | AC | K | K2TNSV | s1i2m2c2n2 |  |
| NZ_AMOR01000004 | R030b | NCA | VOL | America | Canada | W | ABABC | K | K1GA2V | s1i1m1c1n1 | 23661595 |
| NZ_AMOV01000005 | R038b | NCA | VOL | America | Canada | W |  | E | EP | s2i2m2c2n2 | 23661595 |
| NZ_AMOW01000004 | R046Wa | NCA | VOL | America | Canada | W |  | K | K1GA2V | s1i1m1c1n1 | 23661595 |
| NZ_AMOX01000005 | R055a |  |  | America | Canada | W | ABC | E | EP | s1i2m2c2n2 |  |
| NZ_AMOY01000008 | R056a | NCA | VOL | America | Canada | W | AC | K | K2TNSV | s1i2m2c2n2 | 23661595 |
| NZ_AMOS01000005 | R32b | NCA | VOL | America | Canada | W | ABC | K | K2TNSV | s1i1m1c1n1 | 23661595 |
| NZ_MJMW01000024 | S380A | CA | CA | Asia | Singapore | W | ABD | K | K1GA2SV | s1i1m1c2n2 |  |
| NZ_MJMZ01000007 | S468A | CA | CA | Asia | Singapore | W | ABD | K | K1GA2V | s1i1m2c1n1 |  |
| S72494 | S72494 |  |  |  |  |  |  | K | K1GA2SV | s1i1m1c1n1 |  |
| NZ_CBOX010000007 | SA144C |  |  | Africa | South Africa | W |  | E | EP | s2i2m2c2n2 | 23898187 |
| NZ_CBPZ010000002 | SA146A |  |  | Africa | South Africa | W | ABC | K | K1GA1SV | s1i1m1c1n1 | 23898187 |
| NZ_CBOH010000005 | SA155C |  |  | Africa | South Africa | W |  | E | EP | s2i2m2c2n2 | 23898187 |
| NZ_CBPV010000002 | SA156C |  |  | Africa | South Africa | W | ABC | K | K2TNSV | s1i1m1c1n1 | 23898187 |
| NZ_CBNF010000002 | SA157A |  |  | Africa | South Africa | W | ABC | K | K1GA1SV | s1i1m1c1n1 | 23898187 |
| NZ_CBOL010000004 | SA158A |  |  | Africa | South Africa | W | ABC | K | K1GA1SV | s1i1m1c1n1 | 23898187 |
| NZ_CBPY010000003 | SA158C |  |  | Africa | South Africa | W | ABC | K | K1GA1SV | s1i1m1c1n1 | 23898187 |
| NZ_CBQF010000015 | SA160C |  |  | Africa | South Africa | W |  | E | EP | s2i2m2c2n2 | 23898187 |
| NZ_CBPE010000005 | SA161A |  |  | Africa | South Africa | W |  | K | K1GA2SV | s1i1m1c1n1 | 23898187 |
| NZ_CBNM010000002 | SA161C |  |  | Africa | South Africa | W | ABC | K | K1GA2SV | s1i1m1c1n1 | 23898187 |
| NZ_CBOS010000022 | SA162A |  |  | Africa | South Africa | W | ABC | K | K1GA1SV | s1i1m1c1n1 | 23898187 |
| NZ_CBNQ010000022 | SA162C |  |  | Africa | South Africa | W | ABC | K | K1GA1SV | s1i1m1c1n1 | 23898187 |
| NZ_CBOW010000005 | SA163C |  |  | Africa | South Africa | W | ABC | K | K1GA1SV | s1i1m1c1n1 | 23898187 |
| NZ_CBQK010000019 | SA164A |  |  | Africa | South Africa | W |  | E | EP | s2i2m2c2n2 | 23898187 |
| NZ_CBQM010000015 | SA164C |  |  | Africa | South Africa | W |  | E | EP | s2i2m2c2n2 | 23898187 |
| NZ_CBQD010000013 | SA165A |  |  | Africa | South Africa | W |  | E | EP | s2i2m2c2n2 | 23898187 |
| NZ_CBQJ010000015 | SA165C |  |  | Africa | South Africa | W |  | E | EP | s2i2m2c2n2 | 23898187 |
| NZ_CBPU010000002 | SA168A |  |  | Africa | South Africa | W | ABC | K | K1GA1SV | s1i1m2c2n2 | 23898187 |
| NZ_CBOC010000001 | SA168C |  |  | Africa | South Africa | W | ABCC | K | K1GA1SV | s1i1m2c2n2 | 23898187 |
| NZ_CBQH010000012 | SA169A |  |  | Africa | South Africa | W |  | E | EP | s2i2m2c2n2 | 23898187 |
| NZ_CBOK010000012 | SA169C |  |  | Africa | South Africa | W |  | E | EP | s2i2m2c2n2 | 23898187 |
| NZ_CBQL010000004 | SA170C |  |  | Africa | South Africa | W | ABC | K | K1GA1SV | s1i1m1c1n1 | 23898187 |
| NZ_CBOY010000008 | SA172A |  |  | Africa | South Africa | W |  | E | EP | s2i2m2c2n2 | 23898187 |
| NZ_CBPG010000001 | SA172C |  |  | Africa | South Africa | W |  | E | EP | s2i2m2c2n2 | 23898187 |
| NZ_CBOU010000013 | SA173A |  |  | Africa | South Africa | W |  | E | EP | s2i2m2c2n2 | 23898187 |
| NZ_CBNG010000014 | SA173C |  |  | Africa | South Africa | W |  | E | EP | s2i2m2c2n2 | 23898187 |
| NZ_CBPS010000012 | SA174A |  |  | Africa | South Africa | W |  | E | EP | s2i2m2c2n2 | 23898187 |
| NZ_CBPO010000003 | SA175A |  |  | Africa | South Africa | W |  | E | EP | s2i2m2c2n2 | 23898187 |
| NZ_CBOI010000003 | SA175C |  |  | Africa | South Africa | W |  | E | EP | s2i2m2c2n2 | 23898187 |
| NZ_CBNK010000001 | SA194C |  |  | Africa | South Africa | W |  | E | EP | s2i2m2c2n2 | 23898187 |
| NZ_CBOQ010000005 | SA210C |  |  | Africa | South Africa | W | ABC | K | K1GA1SV | s1i1m1c1n1 | 23898187 |
| NZ_CBPK010000031 | SA213C |  |  | Africa | South Africa | W |  | E | EP | s2i2m2c2n2 | 23898187 |
| NZ_CBNS010000014 | SA214C |  |  | Africa | South Africa | W |  | K | K1GA2SV | s1i1m1c1n1 | 23898187 |
| NZ_CBNO010000004 | SA215C |  |  | Africa | South Africa | W | ABC | K | K1GA2SV | s1i1m1c1n1 | 23898187 |
| NZ_CBMY010000015 | SA216A |  |  | Africa | South Africa | W | ABC | K | K1GA2SV | s1i1m1c1n1 | 23898187 |
| NZ_CBNC010000010 | SA216C |  |  | Africa | South Africa | W |  | K | K1GA2SV | s1i1m1c1n1 | 23898187 |
| NZ_CBND010000028 | SA220A |  |  | Africa | South Africa | W | ABC | K | K1GA1SV | s1i1m2c2n2 | 23898187 |
| NZ_CBPX010000026 | SA220C |  |  | Africa | South Africa | W | ABC | K | K1GA1SV | s1i1m2c2n2 | 23898187 |
| NZ_CBQA010000002 | SA221A |  |  | Africa | South Africa | W |  | E | EP | s2i2m2c2n2 | 23898187 |
| NZ_CBPT010000020 | SA221C |  |  | Africa | South Africa | W |  | E | EP | s2i2m2c2n2 | 23898187 |
| NZ_CBQE010000027 | SA222A |  |  | Africa | South Africa | W |  | K | K1GA2SV | s1i1m1c1n1 | 23898187 |
| NZ_CBNH010000001 | SA226A |  |  | Africa | South Africa | W | ABC | K | K1GA2V | s1i1m1c1n1 | 23898187 |
| 1424 | SA227A |  |  | Africa | South Africa | W |  | K | K1GA1SV | s1i1m1c1n1 | 23898187 |
| NZ_CBNL010000021 | SA227C |  |  | Africa | South Africa | W | ABC | K | K1GA1SV | s1i1m1c1n1 | 23898187 |
| NZ_CBMX010000001 | SA233A |  |  | Africa | South Africa | W |  | E | EP | s2i2m2c2n2 | 23898187 |
| NZ_CBPD010000001 | SA233C |  |  | Africa | South Africa | W |  | E | EP | s2i2m2c2n2 | 23898187 |
| NZ_CBQC010000007 | SA251A |  |  | Africa | South Africa | W |  | E | EP | s2i2m2c2n2 | 23898187 |
| NZ_CBOZ010000002 | SA251C |  |  | Africa | South Africa | W |  | E | EP | s2i2m2c2n2 | 23898187 |
| NZ_CBNP010000003 | SA252A |  |  | Africa | South Africa | W |  | K | K1GA1SV | s1i1m1c1n1 | 23898187 |
| NZ_CBOJ010000015 | SA253A |  |  | Africa | South Africa | W |  | E | EP | s2i2m2c2n2 | 23898187 |
| NZ_CBPL010000004 | SA300C |  |  | Africa | South Africa | W | ABC | K | K1GA1SV | s1i1m1c1n1 | 23898187 |
| NZ_CBPH010000005 | SA301A |  |  | Africa | South Africa | W | ABC | K | K1GA1SV | s1i1m1c1n2 | 23898187 |
| NZ_CBNT010000002 | SA301C |  |  | Africa | South Africa | W | ABC | K | K1GA1SV | s1i1m1c1n2 | 23898187 |
| NZ_CBOF010000014 | SA302A |  |  | Africa | South Africa | W |  | E | EP | s2i2m2c2n2 | 23898187 |
| NZ_CBNA010000013 | SA302C |  |  | Africa | South Africa | W |  | E | EP | s2i2m2c2n2 | 23898187 |
| NZ_CBMW010000003 | SA303C |  |  | Africa | South Africa | W |  | E | EP | s2i2m2c2n2 | 23898187 |
| NZ_CBPQ010000003 | SA30A |  |  | Africa | South Africa | W | ABC | K | K1GA1SV | s1i1m1c1n1 | 23898187 |
| NZ_CBQB010000006 | SA30C |  |  | Africa | South Africa | W | ABC | K | K1GA1SV | s1i1m1c1n1 | 23898187 |
| NZ_CBPM010000007 | SA31C |  |  | Africa | South Africa | W | ABC | K | K1GA1SV | s1i1m1c1n1 | 23898187 |
| NZ_CBNY010000041 | SA34C |  |  | Africa | South Africa | W |  | E | EP | s2i2m2c2n2 | 23898187 |
| NZ_CBNE010000015 | SA35A |  |  | Africa | South Africa | W |  | K | K1GA1SV | s1i1m1c1n1 | 23898187 |
| NZ_CBNU010000015 | SA35C |  |  | Africa | South Africa | W |  | K | K1GA1SV | s1i1m1c1n1 | 23898187 |
| NZ_CBQI010000001 | SA36C |  |  | Africa | South Africa | W |  | E | EP | s2i2m2c2n2 | 23898187 |
| NZ_CBOO010000001 | SA37A |  |  | Africa | South Africa | W |  | E | EP | s2i2m2c2n2 | 23898187 |
| NZ_CBOP010000040 | SA37C |  |  | Africa | South Africa | W |  | E | EP | s2i2m2c2n2 | 23898187 |
| NZ_CBNN010000004 | SA40A |  |  | Africa | South Africa | W |  | E | EP | s2i2m2c2n2 | 23898187 |
| NZ_CBNB010000004 | SA45A |  |  | Africa | South Africa | W | ABC | K | K1GA1SV | s1i1m1c1n1 | 23898187 |
| NZ_CBPR010000002 | SA45C |  |  | Africa | South Africa | W | ABC | K | K1GA1SV | s1i1m1c1n1 | 23898187 |
| NZ_CBNV010000013 | SA46A |  |  | Africa | South Africa | W | ABC | K | K1GA1SV | s1i1m1c1n1 | 23898187 |
| 1455 | SA46C |  |  | Africa | South Africa | W |  | K | K1GA1SV | s1i1m1c1n1 | 23898187 |
| NZ_CBOT010000009 | SA47A |  |  | Africa | South Africa | W |  | E | EP | s2i2m2c2n2 | 23898187 |
| NC_014560 | SJM180 | NCA | CG | America | Peru | W | ABC | K | K1GA2SV | s1i1m1c1n1 |  |
| NZ_AZBP01000022 | SS1 | NCA | CG | Asia | China | E | ABC | K | K2TNSV | s1i1m1c1n1 | 28223462 |
| NZ_QBQX01000052 | SSR1 | NCA | GU | Europe | Ireland | W | ABC | K | K2TA1SV | s1i1m1c1n1 |  |
| 3622 | SSR12 |  |  | Europe | Ireland | W |  | E | EP | s1i2m2c2n2 |  |
| 3623 | SSR13 |  |  | Europe | Ireland | W |  | E | EP | s1i2m2c2n2 |  |
| 3624 | SSR14 |  |  | Europe | Ireland | W |  | E | EP | s1i1m2c2n2 |  |
| 3625 | SSR17 |  |  | Europe | Ireland | W |  | E | EP | s1i2m2c2n2 |  |
| 3614 | SSR2 |  |  | Europe | Ireland | W |  | E | EP | s1i2m2c2n2 |  |
| 3627 | SSR20 |  |  | Europe | Ireland | W |  | K | K3TNSV | s1i1m1c1n1 |  |
| 3628 | SSR22 |  |  | Europe | Ireland | W |  | K | K1GA2SV | s1i1m1c1n1 |  |
| 3629 | SSR23 |  |  | Europe | Ireland | W |  | K | K2TNSV | s1i1m1c1n1 |  |
| 3615 | SSR3 |  |  | Europe | Ireland | W |  | E | EP | s1i2m2c2n1 |  |
| 3630 | SSR33 |  |  | Europe | Ireland | W |  | K | K1TNSV | s1i1m2c2n2 |  |
| 3616 | SSR4 |  |  | Europe | Ireland | W |  | E | EP | s1i2m2c2n2 |  |
| 3631 | SSR40 |  |  | Europe | Ireland | W |  | K | K2TNSV | s1i1m1c1n1 |  |
| 3617 | SSR5 |  |  | Europe | Ireland | W |  | E | EP | s1i2m2c2n2 |  |
| 3618 | SSR7 |  |  | Europe | Ireland | W |  | E | EP | s1i2m2c2n2 |  |
| 3620 | SSR9 |  |  | Europe | Ireland | W |  | E | EP | s1i1m2c2n2 |  |
| NZ_MTWP01000004 | SV340_2 | NCA | CG | America | Colombia | W |  | Q | QGA2SV | s1i1m1c1n1 |  |
| NZ_MTWU01000002 | SV380_1 | NCA | IM | America | Colombia | W | ABC | K | K1GA2SV | s1i1m1c1n1 |  |
| NZ_MTWS01000016 | SV397_2 | NCA | CG | America | Colombia | W | ABC | K | K1GA1SV | s1i1m1c1n1 |  |
| NZ_MTWT01000005 | SV449_1 | NCA | IM | America | Colombia | W | ABC | K | K1GA2SV | s1i1m1c1n1 |  |
| 3669 | SW21A |  |  | Europe | Wales | W |  | E | EP | s1i2m2c2n1 |  |
| NC_017376 | Santal49 | NCA | VOL | Asia | India | W | ABC | K | K1GA2SV | s1i1m2c2n2 |  |
| NC_017359 | Sat464 |  |  | America | Peru | W | ABD | K | K1TA1SV | s1i1m1c1n1 |  |
| NC_017741 | Shi112 | NCA | AG | America | Peru | W | ABD | E | EP | s1i2m2c2n1 | 21757722 |
| NC_017739 | Shi417 | NCA | AG | America | Peru | W | ABDC | Q | QGA2SV | s1i1m1c2n1 | 21757722 |
| NC_010698 | Shi470 | NCA | AG | America | Peru | W | ABD | K | K1TA1SV | s1i1m1c1n1 | 21124785 |
| NC_022130 | SouthAfrica20 |  |  | Africa | South Africa | W |  | E | EP | s2i2m2c2n2 | 24072860 |
| AVNI01000002 | SouthAfrica50 |  |  | Africa | South Africa | W |  | E | EP | s2i2m2c2n1 | 24072860 |
| NZ_QBQM01000039 | TN2GF4 | NCA | DU | Asia | Japan | E | ABD | K | K1GA2V | s1i1m1c1n1 | 17202133 |
| NZ_JQNY01000051 | Taiwan-47 |  |  | Asia | China | E | ABD | K | K1GA2V | s1i1m2c2n2 | 25299127 |
| HPU29401 | Tx30a |  |  | America | USA | W |  | E | EP | s2i2m2c2n2 | 7629077 |
| NZ_AONM01000014 | UM007 |  |  | Asia | Malaysia | W | ABD | K | K1GA2SV | s1i1m2c2n2 |  |
| NZ_AONK01000048 | UM018 |  |  | Asia | Malaysia | W | ABC | K | K1GA2SV | s1i1m1c1n1 |  |
| NC_021215 | UM032 | NCA | PU | Asia | Malaysia | W | ABD | K | K1GA2V | s1i1m2c2n2 | 26031894 |
| NZ_AONN01000028 | UM034 |  |  | Asia | Malaysia | W | ABD | K | K1GA2V | s1i1m2c2n2 |  |
| NZ_AUSI01000043 | UM037 | CA | CA | Asia | Malaysia | W | ABC | K | K1GA2V | s1i1m1c1n1 | 24051312 |
| NZ_LELJ01000007 | UM045 | NCA | CG | Asia | Malaysia | W | ABC | K | K1GA2V | s1i1m2c2n2 |  |
| NZ_AONL01000042 | UM054 |  |  | Asia | Malaysia | W |  | E | EP | s2i2m2c2n2 |  |
| NZ_AUSM01000026 | UM065 | NCA | PU | Asia | Malaysia | W | ABD | K | K1GA2V | s1i1m1c1n1 | 24051312 |
| NC_021218 | UM066 | NCA | PU | Asia | Malaysia | W | ABD | K | K1GA2V | s1i1m2c2n2 | 24051312 |
| NZ_AUSQ01000029 | UM077 | NCA | PU | Asia | Malaysia | W | ABD | K | K1GA2V | s1i1m2c2n2 | 24051312 |
| NZ_AUSO01000022 | UM084 | NCA | PU | Asia | Malaysia | W | ABC | K | K1GA2SV | s1i1m1c1n1 | 24051312 |
| NZ_AUSP01000020 | UM085 | NCA | FD | Asia | Malaysia | W | ABD | K | K1GA2V | s1i1m2c2n2 | 24051312 |
| NZ_LFDR01000011 | UM087 | NCA | CG | Asia | Malaysia | W | ABC | K | K1GA2SV | s1i1m1c1n1 |  |
| NZ_AUSR01000011 | UM111 | NCA | FD | Asia | Malaysia | W | ABD | K | K1GA2V | s1i1m2c2n2 | 24051312 |
| NZ_AUSS01000022 | UM114 | NCA | PU | Asia | Malaysia | W | ABC | K | K1GA2SV | s1i1m1c1n1 | 24051312 |
| NZ_LFBY01000015 | UM119 | NCA | CG | Asia | Malaysia | W | ABD | K | K1GA2V | s1i1m2c2n2 |  |
| NZ_LFBX01000009 | UM122 | NCA | CG | Asia | Malaysia | W | ABC | K | K1GA2SV | s1i1m1c1n1 |  |
| NZ_LJXO02000008 | UM137R | NCA | CG | Asia | Malaysia | W | ABD | K | K1GA2V | s1i1m2c2n2 |  |
| NZ_LJXN02000018 | UM137S | NCA | CG | Asia | Malaysia | W | ABD | K | K1A2SV | s1i1m2c2n2 |  |
| NZ_LFCA01000009 | UM139 | NCA | CG | Asia | Malaysia | W | AC | E | EP | s1i2m2c2n2 |  |
| NZ_LFLE01000040 | UM147 |  |  | Asia | Malaysia | W | ABCC | K | K1GA2V | s1i1m1c1n1 |  |
| NZ_LFIS01000012 | UM152 | NCA | CG | Asia | Malaysia | W | ABCC | K | K1GA2SV | s1i1m1c1n1 |  |
| NZ_LFCB01000021 | UM158 | NCA | CG | Asia | Malaysia | W | ABCC | K | K1GA2SV | s1i1m1c1n1 |  |
| NZ_LFJR01000012 | UM163 | NCA | CG | Asia | Malaysia | W | ABD | K | K1GA2V | s1i1m2c2n2 |  |
| NZ_LLVX02000004 | UM163R | NCA | CG | Asia | Malaysia | W | ABD | K | K1GA2V | s1i1m2c2n2 |  |
| NZ_LLVW02000003 | UM163S | NCA | CG | Asia | Malaysia | W | ABD | K | K1GA2V | s1i1m2c2n2 |  |
| NZ_LFIR01000013 | UM165 | NCA | CG | Asia | Malaysia | W | ABCC | E | EP | s1i2m2c2n2 |  |
| NZ_LJXM02000006 | UM171R | NCA | CG | Asia | Malaysia | W | ABD | K | K1GA2V | s1i1m1c1n1 |  |
| NZ_LJXL02000018 | UM171S | NCA | CG | Asia | Malaysia | W | ABD | K | K1GA2V | s1i1m1c2n2 |  |
| NZ_LFKE01000027 | UM202 | NCA | CG | Asia | Malaysia | W | ABC | K | K1GA2V | s1i1m1c1n1 |  |
| NZ_LFKF01000021 | UM209 | NCA | CG | Asia | Malaysia | W |  | E | EP | s2i2m2c2n2 |  |
| NZ_LFKG01000003 | UM211 | NCA | CG | Asia | Malaysia | W | ABC | E | EP | s1i1m2c2n2 |  |
| NZ_LFKH01000001 | UM228 | NCA | CG | Asia | Malaysia | W | ABCC | K | K1GA2SV | s1i1m1c1n1 |  |
| NZ_LJXJ02000003 | UM229R | NCA | CG | Asia | Malaysia | W | ABD | K | K1GA2V | s1i1m1c1n1 |  |
| NZ_LJNZ02000005 | UM229S | NCA | CG | Asia | Malaysia | W | ABD | K | K1GA2V | s1i1m1c1n1 |  |
| NZ_LJXQ02000015 | UM233R | NCA | CG | Asia | Malaysia | W | ABD | K | K1GA2V | s1i1m2c2n2 |  |
| NZ_LJXP02000019 | UM233S | NCA | CG | Asia | Malaysia | W | ABD | K | K1GA2V | s1i1m2c2n2 |  |
| NZ_LFKI01000005 | UM246 | NCA | CG | Asia | Malaysia | W | ABD | K | K1GA2V | s1i1m2c2n2 |  |
| NZ_LJXK02000019 | UM276R | NCA | CG | Asia | Malaysia | W | ABD | K | K1GA2V | s1i1m2c2n2 |  |
| NZ_LJXI02000018 | UM276S | NCA | CG | Asia | Malaysia | W | ABD | K | K1GA2V | s1i1m2c2n2 |  |
| NZ_LFKJ01000019 | UM291 | NCA | CG | Asia | Malaysia | W | ABD | K | K1GA2V | s1i1m1c1n1 |  |
| NZ_LFIT01000014 | UM300 | NCA | CG | Asia | Malaysia | W | AC | K | K1GA1SV | s1i2m2c2n2 |  |
| NZ_LQNB02000013 | UM303R | NCA | CG | Asia | Malaysia | W | ABD | K | K1GA2V | s1i1m1c1n1 |  |
| NZ_LQNA02000019 | UM303S | NCA | CG | Asia | Malaysia | W | ABD | K | K1GA2V | s1i1m1c1n1 |  |
| NZ_LFKK01000018 | UM352 | NCA | CG | Asia | Malaysia | W | ABD | K | K1GA2V | s1i1m1c1n1 |  |
| NZ_LFKL01000019 | UM370 | NCA | CG | Asia | Malaysia | W | ABD | K | K1GA2V | s1i1m2c2n1 |  |
| NZ_LQNC02000033 | UM400AR | NCA | CG | Asia | Malaysia | W | ABC | K | K1GA1SV | s1i1m1c1n1 |  |
| LLVY01000008 | UM400AS | NCA | CG | Asia | Malaysia | W | ABC | K | K1GA1SV | s1i1m1c1n1 |  |
| NZ_MOEF01000017 | UM400b | NCA | CG | Asia | Malaysia | W | ABC | K | K1GA1SV | s1i1m1c1n1 |  |
| NZ_MOEE01000018 | UM400bM | NCA | CG | Asia | Malaysia | W | ABC | K | K1GA1SV | s1i1m1c1n1 |  |
| NZ_LFIU01000001 | UM408 | NCA | CG | Asia | Malaysia | W | ABC | K | K1GA2V | s1i1m1c1n1 |  |
| NZ_LFKM01000008 | UM411 | NCA | CG | Asia | Malaysia | W | ABCC | K | K1GA2SV | s1i1m1c1n1 |  |
| NZ_LQNE02000019 | UM443R | NCA | CG | Asia | Malaysia | W | ABD | K | K1GA2V | s1i1m2c2n2 |  |
| NZ_LQND02000021 | UM443S | NCA | CG | Asia | Malaysia | W | ABD | K | K1GA2V | s1i1m2c2n2 |  |
| NZ_LEOV01000041 | UM520 | NCA | CG | Asia | Malaysia | W | ABD | K | K1GA2V | s1i1m1c1n1 |  |
| NZ_AOTV01000004 | UMB_G1 |  |  | America | Canada | W |  | E | EP | s2i2m2c2n2 |  |
| NZ_QEGD01000019 | VCT187-B122 |  |  | Europe | Spain | W | ABC | K | K2TNSV | s1i1m1c2n2 |  |
| NZ_AWNG01000022 | X47-2AL |  |  |  |  |  |  | E | EP | s1i2m2c2n2 | 24356847 |
| CP003419 | XZ274 | CA | CA | Asia | China | E | ABBD | K | K1GA2V | s1i1m1c2n1 | 22815458 |
| NZ_JPXC01000003 | YN1-91 | NCA | CG | Asia | China | E | ABD | K | K1GA2SV | s1i1m2c2n2 | 25799515 |
| NZ_JPXD01000001 | YN4-84 | NCA | CG | Asia | China | E | ABD | K | K1GA2SV | s1i1m1c1n1 | 25799515 |
| 4458 | Yangon142 |  |  | Asia | Myanmar | W | ABC | K | K1GA2V | s1i1m1c1n1 |  |
| 4457 | Yangon159 |  |  | Asia | Myanmar | W | ABD | K | K1GA2V | s1i1m1c2n1 |  |
| 4456 | Yangon173 |  |  | Asia | Myanmar | W |  | K | K1GA2SV | s1i1m1c1n1 |  |
| 4455 | Yangon179 |  |  | Asia | Myanmar | W | ABC | K | K1GA2V | s1i1m1c1n1 |  |
| 4453 | Yangon190 |  |  | Asia | Myanmar | W | ABC | K | K1GA2SV | s1i1m1c1n1 |  |
| 4452 | Yangon202 |  |  | Asia | Myanmar | W | ABC | K | K1GA2SV | s1i1m1c1n1 |  |
| 4451 | Yangon222 |  |  | Asia | Myanmar | W | ABBD | K | K1GA2V | s1i1m1c1n1 |  |
| 4450 | Yangon233 |  |  | Asia | Myanmar | W | ABC | K | K1GA2SV | s1i1m1c1n1 |  |
| RJDZ01000020 | ZH01 | NCA | CG | Europe | Switzerland | W |  | K | K1GA1SV | s1i1m2c2n2 |  |
| RJEA01000003 | ZH02 | NCA | CG | Europe | Switzerland | W |  | E | EP | s2i2m2c2n2 |  |
| RJEC01000013 | ZH04 | NCA | CG | Europe | Switzerland | W |  | K | K1GA2SV | s1i1m1c2n2 |  |
| RJED01000020 | ZH05 | NCA | CG | Europe | Switzerland | W | ABC | K | K1GA2SV | s1i1m1c1n1 |  |
| RJEE01000012 | ZH06 | NCA | CG | Europe | Switzerland | W |  | K | K1GA2SV | s1i1m1c2n2 |  |
| RJEF01000011 | ZH07 | NCA | CG | Europe | Switzerland | W |  | K | K1GA2SV | s1i1m1c2n2 |  |
| RJEG01000002 | ZH08 | NCA | CG | Europe | Switzerland | W |  | E | EP | s2i2m2c2n2 |  |
| RJEH01000002 | ZH09 | NCA | CG | Europe | Switzerland | W | ABC | Q | QGA2SV | s1i1m1c1n1 |  |
| RJEI01000018 | ZH10 | NCA | CG | Europe | Switzerland | W |  | E | EP | s2i2m2c2n2 |  |
| RJHN01000004 | ZH100 | NCA | CG | Europe | Switzerland | W |  | E | EP | s2i2m2c2n2 |  |
| RJHO01000014 | ZH101 | NCA | CG | Europe | Switzerland | W |  | E | EP | s2i2m2c2n2 |  |
| RJHP01000001 | ZH102 | NCA | CG | Europe | Switzerland | W |  | E | EP | s2i2m2c2n2 |  |
| RJHQ01000012 | ZH103 | NCA | CG | Europe | Switzerland | W |  | Q | QGA2SV | s1i1m2c2n2 |  |
| RJHR01000032 | ZH104 | NCA | CG | Europe | Switzerland | W |  | E | EP | s2i2m2c2n2 |  |
| RJHS01000003 | ZH106 | NCA | CG | Europe | Switzerland | W |  | E | EP | s2i2m2c2n2 |  |
| RJHU01000013 | ZH108 | NCA | CG | Europe | Switzerland | W | ABC | Q | QGA2SV | s1i1m1c1n1 |  |
| RJHV01000005 | ZH109 | NCA | CG | Europe | Switzerland | W | ABB | E | EP | s1i2m2c2n2 |  |
| RJEJ01000016 | ZH11 | NCA | CG | Europe | Switzerland | W |  | K | K1GA2SV | s1i1m1c1n1 |  |
| RJHW01000032 | ZH1110 | NCA | CG | Europe | Switzerland | W | ABC | E | EP | s1i2m2c2n2 |  |
| RJHX01000024 | ZH1111 | NCA | CG | Europe | Switzerland | W | ABC | K | K1GA2V | s1i1m1c1n1 |  |
| RJHY01000020 | ZH112 | NCA | CG | Europe | Switzerland | W | ABC | K | K1GA2SV | s1i1m1c1n1 |  |
| RJHZ01000014 | ZH113 | NCA | CG | Europe | Switzerland | W |  | E | EP | s2i2m2c2n2 |  |
| RJIA01000023 | ZH115 | NCA | CG | Europe | Switzerland | W | ABC | E | EP | s1i2m2c2n2 |  |
| RJIB01000013 | ZH116 | NCA | CG | Europe | Switzerland | W | ABC | Q | QGA2SV | s1i1m1c1n1 |  |
| RJIC01000002 | ZH117 | NCA | CG | Europe | Switzerland | W | ABC | K | K2TNSV | s1i1m1c1n1 |  |
| RJID01000020 | ZH118 | NCA | CG | Europe | Switzerland | W | ABC | E | EP | s1i2m2c2n2 |  |
| RJIE01000002 | ZH119 | NCA | CG | Europe | Switzerland | W | ABC | K | K2TA1SV | s1i1m1c1n1 |  |
| RJIF01000014 | ZH120 | NCA | CG | Europe | Switzerland | W |  | E | EP | s2i2m2c2n2 |  |
| RJIH01000021 | ZH122 | NCA | CG | Europe | Switzerland | W |  | E | EP | s2i2m2c2n2 |  |
| RJIJ01000036 | ZH124 | NCA | CG | Europe | Switzerland | W | ABC | Q | QGA2SV | s1i1m1c1n1 |  |
| RJIL01000023 | ZH126 | NCA | CG | Europe | Switzerland | W |  | E | EP | s2i2m2c2n2 |  |
| RJIN01000042 | ZH128 | NCA | CG | Europe | Switzerland | W |  | K | K1GA1SV | s1i1m1c2n2 |  |
| RJEM01000004 | ZH13 | NCA | CG | Europe | Switzerland | W | ABC | K | K2TNSV | s1i1m1c1n1 |  |
| RJIP01000014 | ZH130 | NCA | CG | Europe | Switzerland | W |  | E | EP | s2i2m2c2n2 |  |
| RJIR01000021 | ZH132 | NCA | CG | Europe | Switzerland | W |  | E | EP | s2i2m2c2n2 |  |
| RJIS01000004 | ZH133 | NCA | CG | Europe | Switzerland | W | ABC | E | EP | s1i2m2c2n2 |  |
| RJIT01000005 | ZH134 | NCA | CG | Europe | Switzerland | W |  | E | EP | s2i2m2c2n2 |  |
| RJIV01000003 | ZH136 | NCA | CG | Europe | Switzerland | W |  | E | EP | s2i2m2c2n2 |  |
| RJIX01000034 | ZH138 | NCA | CG | Europe | Switzerland | W |  | E | EP | s2i2m2c2n1 |  |
| RJIY01000004 | ZH139 | NCA | CG | Europe | Switzerland | W | ABC | Q | QGA2SV | s1i1m1c1n1 |  |
| RJEN01000003 | ZH14 | NCA | CG | Europe | Switzerland | W | ABC | E | EP | s1i1m2c2n2 |  |
| RJIZ01000020 | ZH140 | NCA | CG | Europe | Switzerland | W |  | K | K1GA2V | s1i1m1c1n1 |  |
| RJEO01000027 | ZH15 | NCA | CG | Europe | Switzerland | W |  | E | EP | s1i2m2c2n2 |  |
| RJEP01000012 | ZH16 | NCA | CG | Europe | Switzerland | W | ABC | K | K1GA2V | s1i1m1c1n1 |  |
| RJEQ01000015 | ZH17 | NCA | CG | Europe | Switzerland | W | ABC | E | EP | s1i2m2c2n2 |  |
| RJER01000002 | ZH18 | NCA | CG | Europe | Switzerland | W |  | E | EP | s2i2m2c2n2 |  |
| RJES01000016 | ZH19 | NCA | CG | Europe | Switzerland | W |  | E | EP | s2i2m2c2n2 |  |
| RJEU01000017 | ZH21 | NCA | CG | Europe | Switzerland | W | ABC | E | EP | s1i2m2c2n2 |  |
| RJEV01000011 | ZH22 | NCA | CG | Europe | Switzerland | W | ABC | E | EP | s1i2m2c2n1 |  |
| RJEY01000032 | ZH25 | NCA | CG | Europe | Switzerland | W | ABC | K | K2TNSV | s1i1m1c1n1 |  |
| RJEZ01000026 | ZH26 | NCA | CG | Europe | Switzerland | W | ABC | E | EP | s1i2m2c2n1 |  |
| RJFA01000015 | ZH27 | NCA | CG | Europe | Switzerland | W | ABC | E | EP | s1i2m2c2n1 |  |
| RJFB01000020 | ZH28 | NCA | CG | Europe | Switzerland | W | ABC | K | K1GA2SV | s1i1m1c1n1 |  |
| RJFC01000026 | ZH29 | NCA | CG | Europe | Switzerland | W |  | E | EP | s2i2m2c2n1 |  |
| RJEB01000002 | ZH3 | NCA | CG | Europe | Switzerland | W |  | E | EP | s2i2m2c2n2 |  |
| RJFD01000005 | ZH30 | NCA | CG | Europe | Switzerland | W |  | E | EP | s2i2m2c2n2 |  |
| RJFE01000023 | ZH31 | NCA | CG | Europe | Switzerland | W | ABCC | K | K1GA2SV | s1i1m2c2n1 |  |
| RJFF01000001 | ZH32 | NCA | CG | Europe | Switzerland | W |  | E | EP | s2i2m2c2n2 |  |
| RJFG01000012 | ZH33 | NCA | CG | Europe | Switzerland | W | ABC | E | EP | s1i2m2c2n2 |  |
| RJFH01000010 | ZH34 | NCA | CG | Europe | Switzerland | W |  | E | EP | s2i2m2c2n2 |  |
| RJFI01000003 | ZH35 | NCA | CG | Europe | Switzerland | W | ABC | K | K1GA2SV | s1i1m1c1n1 |  |
| RJFJ01000010 | ZH36 | NCA | CG | Europe | Switzerland | W |  | E | EP | s2i2m2c2n2 |  |
| RJFM01000012 | ZH39 | NCA | CG | Europe | Switzerland | W |  | E | EP | s2i2m2c2n2 |  |
| RJFN01000002 | ZH40 | NCA | CG | Europe | Switzerland | W | ABC | E | EP | s1i2m2c2n2 |  |
| RJFO01000013 | ZH41 | NCA | CG | Europe | Switzerland | W | ABC | K | K1GA2SV | s1i1m1c1n1 |  |
| RJFQ01000010 | ZH43 | NCA | CG | Europe | Switzerland | W | ABC | K | K1GA1SV | s1i1m1c1n2 |  |
| RJFR01000038 | ZH45 | NCA | CG | Europe | Switzerland | W |  | E | EP | s2i2m2c2n2 |  |
| RJFS01000012 | ZH46 | NCA | CG | Europe | Switzerland | W |  | E | EP | s2i2m2c2n2 |  |
| RJFT01000009 | ZH47 | NCA | CG | Europe | Switzerland | W |  | E | EP | s2i2m2c2n2 |  |
| RJFU01000016 | ZH48 | NCA | CG | Europe | Switzerland | W | ABD | K | K1GA2V | s1i1m2c2n2 |  |
| RJHI01000003 | ZH5 | NCA | CG | Europe | Switzerland | W | ABC | K | K1GA1SV | s1i1m1c1n1 |  |
| RJFX01000001 | ZH51 | NCA | CG | Europe | Switzerland | W |  | E | EP | s2i2m2c2n2 |  |
| RJFY01000019 | ZH52 | NCA | CG | Europe | Switzerland | W | ABC | K | K1GA2SV | s1i1m1c1n1 |  |
| RJFZ01000022 | ZH53 | NCA | CG | Europe | Switzerland | W | ABC | E | EP | s1i2m2c2n2 |  |
| RJGA01000006 | ZH54 | NCA | CG | Europe | Switzerland | W | ABC | E | EP | s1i2m2c2n2 |  |
| RJGC01000023 | ZH56 | NCA | CG | Europe | Switzerland | W |  | K | K1GA2SV | s1i1m1c2n2 |  |
| RJGD01000015 | ZH57 | NCA | CG | Europe | Switzerland | W |  | K | K1GA2V | s1i2m2c2n2 |  |
| RJGE01000015 | ZH58 | NCA | CG | Europe | Switzerland | W | ABD | K | K1GA2V | s1i1m1c1n1 |  |
| RJGF01000005 | ZH59 | NCA | CG | Europe | Switzerland | W |  | E | EP | s1i2m2c2n2 |  |
| RJGG01000009 | ZH60 | NCA | CG | Europe | Switzerland | W |  | E | ENSV | s1i2m2c2n2 |  |
| RJGH01000021 | ZH61 | NCA | CG | Europe | Switzerland | W |  | E | EP | s2i2m2c2n2 |  |
| RJGI01000001 | ZH62 | NCA | CG | Europe | Switzerland | W | ABC | K | K1GA2SV | s1i1m1c1n1 |  |
| RJGJ01000009 | ZH63 | NCA | CG | Europe | Switzerland | W | ABC | K | K1GA2V | s1i1m1c1n1 |  |
| RJGK01000006 | ZH65 | NCA | CG | Europe | Switzerland | W |  | E | EP | s2i2m2c2n1 |  |
| RJGL01000014 | ZH66 | NCA | CG | Europe | Switzerland | W |  | E | EP | s2i2m2c2n2 |  |
| RJGM01000009 | ZH67 | NCA | CG | Europe | Switzerland | W | ABC | K | K1TA1SV | s1i1m1c1n1 |  |
| RJGN01000010 | ZH68 | NCA | CG | Europe | Switzerland | W | ABC | K | K1GA1SV | s1i1m1c1n1 |  |
| RJGO01000012 | ZH69 | NCA | CG | Europe | Switzerland | W |  | E | EP | s2i2m2c2n2 |  |
| RJGQ01000022 | ZH72 | NCA | CG | Europe | Switzerland | W | ABC | Q | QGA2SV | s1i1m1c1n1 |  |
| RJGR01000002 | ZH75 | NCA | CG | Europe | Switzerland | W | ABC | K | K2TNSV | s1i1m2c2n2 |  |
| RJGS01000021 | ZH76 | NCA | CG | Europe | Switzerland | W | ABC | E | EP | s1i2m2c2n2 |  |
| RJGU01000003 | ZH78 | NCA | CG | Europe | Switzerland | W |  | K | K2TA1SV | s1i1m1c1n1 |  |
| RJGV01000002 | ZH79 | NCA | CG | Europe | Switzerland | W | ABC | K | K1GA1SV | s1i1m1c1n1 |  |
| RJGW01000023 | ZH80 | NCA | CG | Europe | Switzerland | W |  | E | EP | s2i2m2c2n2 |  |
| RJGX01000018 | ZH82 | NCA | CG | Europe | Switzerland | W |  | E | EP | s2i2m2c2n2 |  |
| RJGY01000002 | ZH83 | NCA | CG | Europe | Switzerland | W |  | E | EP | s2i2m2c2n2 |  |
| RJGZ01000004 | ZH84 | NCA | CG | Europe | Switzerland | W | ABC | E | EP | s1i2m2c2n2 |  |
| RJHA01000017 | ZH85 | NCA | CG | Europe | Switzerland | W |  | E | EP | s2i2m2c2n2 |  |
| RJHB01000013 | ZH86 | NCA | CG | Europe | Switzerland | W | ABC | K | K1GA1SV | s1i1m1c1n1 |  |
| RJHD01000001 | ZH88 | NCA | CG | Europe | Switzerland | W |  | E | EP | s1i2m2c2n2 |  |
| RJHF01000031 | ZH91 | NCA | CG | Europe | Switzerland | W | AB | E | EP | s1i2m2c2n2 |  |
| RJHG01000002 | ZH93 | NCA | CG | Europe | Switzerland | W |  | K | K1GA2SV | s1i2m2c2n2 |  |
| RJHH01000018 | ZH94 | NCA | CG | Europe | Switzerland | W |  | E | EP | s2i2m2c2n2 |  |
| RJHJ01000018 | ZH96 | NCA | CG | Europe | Switzerland | W | ABDD | K | K1GA1SV | s1i1m1c2n1 |  |
| RJHK01000001 | ZH97 | NCA | CG | Europe | Switzerland | W |  | K | K1GA2SV | s1i1m1c1n1 |  |
| RJHL01000004 | ZH98 | NCA | CG | Europe | Switzerland | W | ABD | E | EP | s1i2m2c2n2 |  |
| RJHM01000002 | ZH99 | NCA | CG | Europe | Switzerland | W | ABC | E | EP | s1i2m2c2n2 |  |
| RJFW01000009 | Zh50 | NCA | CG | Europe | Switzerland | W |  | K | K1GA1SV | s2i1m2c2n2 |  |
| NZ_CP011485 | ausabrJ05 |  |  | Oceania | Australia | W |  | K | K1N2SV2 | s1i1m1c1n1 |  |
| AF191639 | ch2 |  |  |  |  |  |  | K | K1GA2SV | s1i1m1c2n2 | 10816542 |
| NZ_CP026325 | dRdM1 |  |  |  |  |  | ABC | K | K2TNSV | s1i1m1c1n1 |  |
| NZ_CP026515 | dRdM2addM2 |  |  |  |  |  | ABC | K | K2TNSV | s1i1m1c1n1 |  |
| 8722 | ms1054 |  |  | America | Mexico | W |  | K | K1A2SV | s1i1m1c1n1 |  |
| 8684 | ms1055 | CA | CA | America | Mexico | W |  | Q | QGA2SV | s1i1m1c1n1 |  |
| 8688 | ms1063 |  |  | America | Mexico | W |  | K | K2TNSV | s1i1m1c1n1 |  |
| 8697 | ms1078 | CA | CA | America | Mexico | W |  | K | K1GA2V | s1i1m1c1n1 |  |
| 8715 | ms1080 | CA | CA | America | Mexico | W |  | K | K2TNSV | s1i1m1c1n1 |  |
| 8716 | ms13 | NCA | CG | America | Mexico | W |  | K | K1GA1SV | s1i1m1c1n1 |  |
| 8703 | ms15 | NCA | CG | America | Mexico | W |  | K | K2TNSV | s1i1m1c1n1 |  |
| 8683 | ms167 |  |  | America | Mexico | W |  | K | K1GA2SV | s1i1m1c1n1 |  |
| 8762 | ms176 | NCA | CG | America | Mexico | W |  | K | K2TNSV | s1i1m1c1n1 |  |
| 8694 | ms203 | NCA | CG | America | Mexico | W |  | K | K1GA1SV | s1i1m1c1n1 |  |
| 8705 | ms23 | NCA | CG | America | Mexico | W |  | K | K1GA1SV | s1i1m1c1n1 |  |
| 8723 | ms44 |  |  | America | Mexico | W |  | K | K1GA2SV | s1i1m1c1n1 |  |
| 8713 | ms931 | CA | CA | America | Mexico | W |  | K | K1GA1SV | s1i1m1c1n1 |  |
| NZ_CP006820 | oki102 | NCA | AG | Asia | Japan | E | ABC | E | EP | s1i2m2c2n2 | 24744331 |
| NZ_CP006821 | oki112 | NCA | AG | Asia | Japan | E | ABC | E | EP | s1i2m2c2n2 | 24744331 |
| NZ_CP006822 | oki128 | NCA | AG | Asia | Japan | E |  | E | EP | s2i2m2c2n2 | 24744331 |
| NZ_CP006823 | oki154 | NCA | DU | Asia | Japan | E |  | E | EP | s2i2m2c2n2 | 24744331 |
| NZ_CP006824 | oki422 | NCA | AG | Asia | Japan | E | ABC | E | EP | s1i2m2c2n2 | 24744331 |
| NZ_CP006825 | oki673 | NCA | GU | Asia | Japan | E |  | E | EP | s2i2m2c2n2 | 24744331 |
| NZ_CP006827 | oki898 | NCA | DU | Asia | Japan | E | ABC | E | EP | s1i2m2c2n2 | 24744331 |
| Z26883 | pWS10, pWS16 |  |  |  |  |  |  | K | K2TA1SV | s1i1m1c1n1 |  |
| NC_017355 | v225d | NCA | CG | America | Venezuela | W | ABDC | K | K1GA2SV | s1i1m1c1n1 | 20400544 |
| NZ_JCKE01000013 | wls-5-1 |  |  | Asia | China | E | ABD | K | K1GA2SV | s1i1m1c1n1 |  |
| NZ_JDVP01000031 | wls-5-10 |  |  | Asia | China | E | ABD | K | K1GA2SV | s1i1m1c1n1 |  |
| NZ_JDVO01000045 | wls-5-11 |  |  | Asia | China | E | ABD | K | K1GA2SV | s1i1m1c1n1 |  |
| NZ_JDVN01000020 | wls-5-13 |  |  | Asia | China | E | ABD | K | K1GA2SV | s1i1m1c1n1 |  |
| NZ_JDVM01000044 | wls-5-14 |  |  | Asia | China | E | ABD | K | K1GA2SV | s1i1m1c1n1 |  |
| NZ_JDVL01000048 | wls-5-15 |  |  | Asia | China | E | ABD | K | K1GA2SV | s1i1m1c1n1 |  |
| NZ_JDVK01000075 | wls-5-16 |  |  | Asia | China | E | ABD | K | K1GA2SV | s1i1m1c1n1 |  |
| NZ_JDVJ01000022 | wls-5-17 |  |  | Asia | China | E | ABD | K | K1GA2SV | s1i1m1c1n1 |  |
| NZ_JDVI01000026 | wls-5-18 |  |  | Asia | China | E | ABD | K | K1GA2SV | s1i1m1c1n1 |  |
| NZ_JCKD01000017 | wls-5-2 |  |  | Asia | China | E | ABD | K | K1GA2SV | s1i1m1c1n1 |  |
| NZ_AUPD01000009 | wls-5-3 | NCA | CG | Asia | China | E | ABD | K | K1GA2SV | s1i1m1c1n1 |  |
| NZ_JDVV01000047 | wls-5-4 |  |  | Asia | China | E | ABD | K | K1GA2SV | s1i1m1c1n1 |  |
| NZ_JDVU01000042 | wls-5-5 |  |  | Asia | China | E | ABD | K | K1GA2SV | s1i1m1c1n1 |  |
| NZ_JDVT01000101 | wls-5-6 |  |  | Asia | China | E | ABD | K | K1GA2SV | s1i1m1c1n1 |  |
| NZ_JDVS01000100 | wls-5-7 |  |  | Asia | China | E | ABD | K | K1GA2SV | s1i1m1c1n1 |  |
| NZ_JDVR01000055 | wls-5-8 |  |  | Asia | China | E | ABD | K | K1GA2SV | s1i1m1c1n1 |  |
| NZ_JDVQ01000036 | wls-5-9 |  |  | Asia | China | E | ABD | K | K1GA2SV | s1i1m1c1n1 |  |

Supplementary Table 2: Information about the 1259 strains included in the study.

**CA/NCA*:** Host disease were classified as either CA (Gastric adenocarcinoma and MALT) or NCA (Non-gastric cancer lesions: Chronic gastritis (CG), Duodenal ulcer (DU), Gastric ulcer (GU), Functional dyspepsia (FD), Peptic ulcer (PU), Atrophic gastritis (AG), Intestinal metaplasia (IM), Volunteer (VOL),

**W_EAST****: Hp strain types classified into either Western (W) or East-Asian (E)
